# Phthalic anhydride (PA): a valuable substrate in organic transformations

**DOI:** 10.1039/d3ra03378c

**Published:** 2023-08-15

**Authors:** Kobra Nikoofar, Mansoorehsadat Sadathosainy

**Affiliations:** a Department of Organic Chemistry, Faculty of Chemistry, Alzahra University P.O. Box 1993891176 Tehran Iran k.nikoofar@alzahra.ac.ir kobranikoofar@yahoo.com +98 2188041344 +98 2188041344

## Abstract

This review has been centralized on applications of phthalic anhydride (PA) as a valuable and significant heterocyclic substrate in two- and multicomponent organic reactions. The article has been subdivided into the following parts: (i) PA introduction by focusing on its characterization, synthesizing procedure, and multiple-aspect applications. In addition, the previous review articles based on PA have also been indicated; (ii) the applications of PA as a substrate have been subdivided into parts with a glance on the reaction components numbers; (iii) the applications of PA in esterification reactions; and (iv) some examples of PA in multistep synthesis. The review covers the corresponding literature up to the end of 2022. According to the abovementioned classifications, PA is a potent substrate to design a wide range of heterocyclic compounds that possess various kinds of properties and applications in chemistry, industry, and pharmaceuticals.

## Abstract 2

This review has been centralized on applications of phthalic anhydride (PA) as a valuable and significant heterocyclic substrate in two- and multicomponent organic reactions.

## Graphical abstract

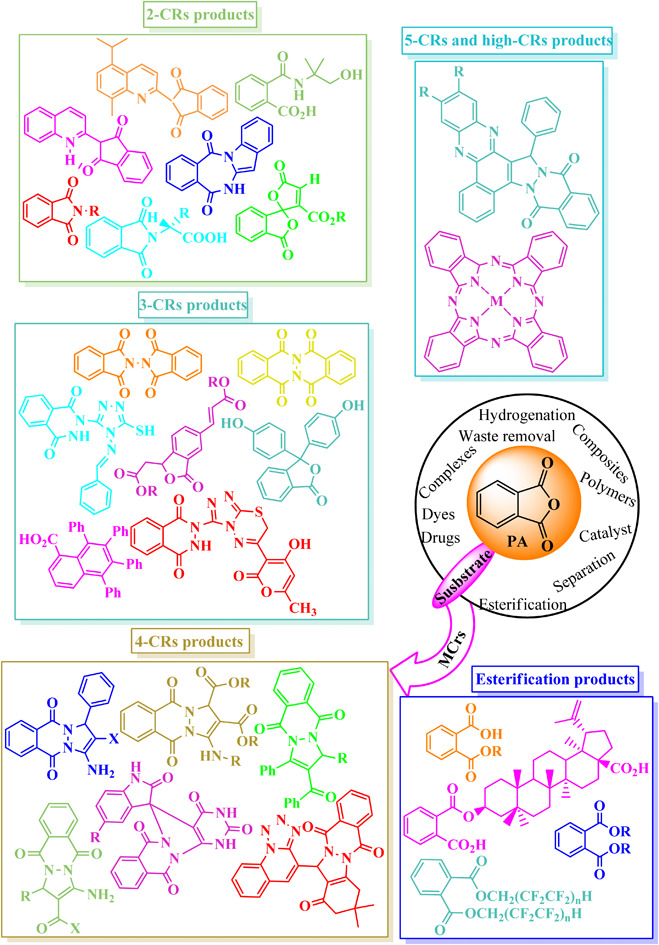

## 1. Introduction

2-Benzofuran-1,3-dione (isobenzofuran-1,3-dione, 1,3-isobenzofuranidone, 1,3-dioxophthalan, phthalandione, 1,3-phthalandione, 1,2-benzenedicarboxylic acid anhydride, phthalic acid anhydride, 1,2-benzenedicarboxylic anhydride, 1,3-dihydro-1,3-dioxoisobenzofuran) with the common/popular name of phthalic anhydride (PA) is a versatile organic compound with the formula C_6_H_4_(CO)_2_O. It is a white (or somehow colorless) powder (or flakes and sometimes needles) with a melting point of 131 °C, molar mass of 148.1 g mol^−1^, and some acidic odor. Actually, this fused bicycle with the LD_50_ = 1.530 mg kg^−1^ (oral rat) is the anhydride form of phthalic acid. It is soluble in ethanol, acetone, water, and benzene but sparingly in ether and chloroform.^1^ Contact with skin and eye may cause an allergic skin reaction and serious eye damage. Basketter and Kimber published a review article in 2016, in which the absence of both skin- and respiratory-sensitizing capacity of PA was confirmed based on interpreting predictive toxicology tests for skin sensitization.^2^

This white solid was first synthesized by Auguste Laurent in 1836. The first procedure involved liquid-phase mercury-catalyzed oxidation of naphthalene.^3^ In the modern process, vanadium pentoxide was used as the catalyst in a gas-phase reaction with naphthalene using molecular oxygen.^4^ Rindone group in 2010 prepared PA *via* the ozonation of naphthalene.^5^

An alternative synthetic procedure is also based on the oxidation of *o*-xylene by vanadium-based catalysts such as V_2_O_5_/TiO_2_ and anatase-supported vanadium oxide.^6–8^ In 2018, Co–Mn/H_3_PW_12_O_40_@TiO_2_ was also utilized as the catalyst for the selective vapor-phase oxidation of *o*-xylene to PA in the presence of green oxidant.^9^ Boger and Menegola also used extruded monoliths with high thermal conductivity as improved operated and economic catalysts to prepare PA from the oxidation of *o*-xylene.^10^ This technology was also studied in detail by Tronconi group in 2012.^11^ The air oxidation of *o*-xylene over mesoporous V-Mo-MCM-41 molecular sieves was also applied for PA synthesis by Selvaraj and Lee in 2005.^12^ PA can be also produced from phthalic acid.^13^ Nikolov's research group published a review article on the synthesis of PA from *o*-xylene in detail.^14^ Dias and Portela in 1997 issued a review article that discussed about various catalysts to gain PA from two main routes for its synthesis, which concerns the oxidation of *o*-xylene and naphthalene.^15^

Recently some green and novel protocols have been presented to obtain PA such as renewable production from biomass-derived furan and maleic anhydride.^16^ Deng group in 2021 also reported green production of PA from biobased furan and maleic anhydride by an acid resin catalyst.^17^ PA obtained renewably form 5-hydroxymethfurfural (HMF) using MoO_3_/Cu(NO_3_)_2_ as a catalyst.^18^ Sha group in 2016 designed a conceptual process to prepare PA from corn stover, an agricultural residue. Their techno-economic assessment contends energy integration alternatives as well as water consumption and life cycle greenhouse emissions.^19^ Ierapetritou and coworkers in 2015 accounted a novel route for PA production from hemicellulose solutions, in which they focused on synthetic process, technoeconomic analysis, and life cycle assessment (LCA) of the protocol.^20^

Kinney and Pincus in 1951 reported PA production from catalytic air oxidation of some substrates such as certain higher aromatics and coal tar fractions.^21^ The PA synthesis by the oxidation of tar oils was also represented by Shelmerdine's group in 1953.^22^

Prichard in 1956 obtained PA from the reaction of bromobenzene and carbon monoxide in the presence of sodium carbonate and nickel carbonyl.^23^

Zazhigalov and Kiziun in 2017 reported PA production by Diels–Alder reaction during *n*-pentane oxidation on vanadium phosphorus oxide (VPO) catalyst.^24^ Kim and Yang in 2000 studied the synthesis of maleic and phthalic anhydrides from the mixture of cyclopentene and 1-pentene *via* their selective oxidations under different reaction parameters.^25^

In 1919, Gibbs published the title “phthalic anhydride. I-introduction”, which discussed the different synthetic procedures of PA.^26^ Gibbs in 1920 also interpreted the title “history of the preparation and properties of pure phthalic anhydride”.^27^

The chemical engineering design of the PA preparation reactor is considered based on the computer model that simulates the operation of the reactor over a range of conditions that lead to both satisfactory mass transfer and heat transfer requirements.^28^ Wainwright and Foster in 1979, reviewed the “catalysts, kinetics, and reactor design in phthalic anhydride synthesis”.^29^

PA has been utilized in different fields of science and technology. In polymerization technologies, some examples such as preparation of ternary polymeric system including polysulfide (PS), diglycidylether of bisphenol A resin, and PA have been reported. PA plays a role in the curing reaction of this ternary polymer.^30^ The copolymerization of PA with epoxides catalyzed by amine-bis(phenolate)chromium(iii) complexes also performed in 2021.^31^ Amin's group in 2011 prepared two hyperbranched polyesteramides (HB1 and HB2) by the bulk reaction between PA and diisopropanolamine (DiPA) or diethanolamine (DEA), respectively. The effects of various solutions of HB1 and HB2 on the properties (such as measurements of water of consistency, setting times, bulk density, apparent porosity, and compressive strength for the cement pastes) of ordinary Portland cement (OPC) and Portland limestone cement (PLC) were studied.^32^ Duchateau's group constructed a partially renewable polyester *via* the catalytic ring-opening copolymerization of limonene oxide and PA.^33^

Pooley and coworkers in 2005 copolymerized PA as an electrophilic monomer with aziridine or 2-methylaziridine as nucleophilic monomers in the absence of initiator under various experimental conditions.^34^

PA was utilized as a bridge for the grafting of chitosan biopolymer onto wool fabric to prepare antibacterial agents.^35^ It also played a role in the chemical modification of chitosan to obtain bacteria inhibitors.^36^ In 2020, a novel biodegradable diblock/triblock poly(ester-bicarbonate)s was prepared from cyclohexene oxide (CHO), propylene oxide (PO), PA, and CO_2_ in a one-pot/one-step protocol.^37^ The surface modification of silk fiber using PA to graft the polysaccharide chitosan has been reported in 2009, which lead to the dyeing ability of the grafted silk. The grafted samples possess antibacterial potential.^38^

PA is also a critical substrate to prepare a series of new optically active and thermally stable polyamides (PAs).^39^ It plays the role of a chain end group in the fluorescent PMMAs polymers. These polymers can detect the intermacromolecular reaction in reactive polymer blends at a low concentration using fluorescence-gel permeation chromatography.^40^

It has also utilized as a key substrate for the multistep preparation of novel optically active polyamides derived from 5-(3-methyl-2-phthalimidylpentanoylamino)isophthalic acid.^41^ PA was also utilized for the preparation of chiral polyesters through enantioselective terpolymerization with racemic and meso-epoxides and also sequence-controlled block copolymers.^42^

PA was also applied as a modifier in poly(butylene succinate-*co*-butylene adipate) (PBSA)/cellulose nanocrystal (CNC) composites. The modified PBSA/CNC composites demonstrated elevated mechanical properties, fast crystallization, and improved hydrophobicity.^43^ PA has been added in the preparation procedure of poly(butylenes succinate)/cellulose nanocrystals (PBS/CNC) composite *via* melt blending, which yielded higher crystallinity and smaller crystals.^44^

To obtain phthalylated cellulosic compounds with higher DS (degree of substitution), the chemical modification of sugarcane bagasse cellulose has been reported with PA in the 1-butyl-3-methyl imidazolium chloride solvent and 4-dimethylaminopyridine (DAMP) catalyst.^45^

PA is also significant in different fields of biological and pharmaceutical applications. It is a key substrate in the synthesis of symmetrical novel organoselenocyanates and diselenides dye stuffs, which was evaluated for the antitumor properties.^46^ In 2012, *N*-benzoyl 3-nitro-phthalimide, which possesses anxiolytic activity in mice model, was prepared from PA as the substrate.^47^

Bis[aqua-1,8-(1,2-dicarboxamido benzene)3,6-diazaoctane copper(ii)/nickel(ii)] tetrachloride, as two binuclear complex, was synthesized by a two-component one-pot metal template condensation between PA and 1,8-diamino-3,6-diazaoctane. The complexes are able to bind to calf thymus (CT)-DNA under physiological pH.^48^ Lepoittevin *et al.* in 2021 investigated the reactivity of PA as a chemical respiratory sensitizer toward reconstructed human epidermis (RHE).^49^

Spanedd and Bourel-Bonnet published a review article about the potential of cyclic anhydrides such as PA in bioconjugation to functionalize the biomolecules and carriers. The pH-dependent stability and reactivity, as well as the physical properties, can be tuned by the structure of the cyclic anhydride used. Thus, their application in smart delivery systems has become very important.^50^

Alheety in 2021 prepared new complexes of PA (as ligand) with some cations (such as Co(ii), Ni(ii), Cu(ii), Mn(ii), and Zn(ii)) in 1 : 1 molar ratio. The microbicide activity studies of the synthesized complexes against four types of bacteria (*E. coli*, *S. epidermidis*, *K. pneumoniae*, and *S. aureus*) was also reported. The complexes also demonstrated stability on laser beams for 10–30 s.^51^

Marzouk's group in 2016 designed and manufactured some new phthalazinones containing benzyl moiety *via* a multistep reaction started from PA. Some of the heterocycles demonstrated antitumor activity.^52^ Yamaguchi and coworkers in 1998 constructed some 2-[2-(l-imidazolyl)alkyl]-1(2*H*)-phthalazinones as novel antiasthmatic agents with dual activities of thromboxane A2 synthetase inhibition and bronchodilation.^53^ Costa Silva group in 2021 modified Chicha gum with PA to obtain a new biologically active material that demonstrated excellent inhibitory effect against *P. aeruginosa* and *K. pneumoniae* species (rating 100% inhibition) and could also inhibit *Escherichia coli* growth.^54^ Bold *et al.* in 2000 synthesized new anilinophthalazines as potent and orally well absorbed inhibitors of the VEGF receptor tyrosine kinases useful as antagonists of tumor-driven angiogenesis. The key substrate for the preparation procedure starts from PA.^55^

PA also has a special role in sensors. In 2016, phthaloylchitosan (PHCS) was synthesized simply and cost-effectively using chitosan and PA by microwave irradiation, which was applied to determine tyrosine with high sensitivity and good selectivity through carbon nanotube film-coated glassy carbon electrode.^56^ Zhang *et al.* in 2022 reported a novel phthalic anhydride-based room-temperature phosphorescence (RTP) emitter with the lifetime longer than one second.^57^

A novel fluorescent probe IMPD, based on imidazo[1,2-*a*]pyridine that contained PA moiety in its structure, was designed and synthesized by Huang's research group in 2021. The probe could detect hydrazine *via* its maleimide as the recognition group. In addition, the probe IMPD could dye the HepG2 cell with blue color in the presence of hydrazine.^58^

Another notable application of PA is in the dye industry. It utilized to prepare the nanosized copper phthalocyanin blue (CuPc) pigments.^59^ The phenol precipitation and dye bleaching capabilities of phthalic anhydride-modified horseradish peroxidase C (PA-HRP) were also investigated in 2000.^60^

Polymeric surfactants have been synthesized by the reaction of maleic anhydride (MA), polyethylene glycol (PEG), and PA, which exhibit excellent surface-active properties (including surface tension, low-foaming, solubilization, and dispersant properties) in disperse dye systems.^61^

PA also plays a role in separation and waste removal. In 2016, the Gurgel group prepared chemically-modified sugarcane bagasse, named as carboxylate-functionalized sugarcane bagasse (SPA), *via* the reaction of PA with sugarcane bagasse. The SPA adsorbent was used to remove Co^2+^, Cu^2+^, and Ni^2+^ from aqueous solution in mono- and multicomponent systems in the batch mode.^62^

Another aspect of the PA usage is in the field of catalyst and protecting group. It was utilized as a part of the co-catalytic system (in combination with Zn(OTf)_2_) to promote Beckmann rearrangement.^63^ The efficient and clean oxidation of sulfides to sulfones (not the probable sulfoxide) with urea–hydrogen peroxide in the presence of PA in ethyl acetate was also performed in 2018.^64^ PA also worked as a remarkable protective group in the synthesis of a dipeptide (β-alanine-l-histidine) *via* two procedures, which are solution phase peptide synthesis (SPS) and solid phase peptide synthesis (SPPS).^65^

Liu *et al.* in 2017 utilized PA as a low-temperature activator in the H_2_O_2_ bleaching system for cotton fabric. The performance of the H_2_O_2_/PA bleaching system was investigated by measuring the CIE whiteness index (WI) of the bleached cotton fabric, H_2_O_2_ decomposition rate, and bursting strength, respectively.^66^

Maldas and Kokta in 1990 considered the performance of PA as a coupling agent in wood fiber-filled polystyrene composites. Its presence evaluated the mechanical properties of the composite materials.^67^

Duan in 2019 interpreted PA-promoted ring-opening cationic polymerization of cyclohexene oxide catalyzed by dinuclear chromium complex supported by piperazine-bridged [ONSO] ligand to obtain atactic poly(cyclohexeneoxide) polymer. The formation of carbocation species by interaction between PA and bimetallic chromium complexes is the real initiator center.^68^

The hydrogenation of PA is another important reaction that has been evaluated extensively. Liquid phase selective hydrogenation of PA to phthalide (an important industrial intermediate for pharmaceuticals, fine chemicals, and organic synthesis) was reported in 2015 in the presence of Au/FeO_*x*_–TiO_2_ catalyst.^69,70^ Liquid phase hydrogenation of PA to phthalide over Au/TiO_2_ catalysts (with different gold loadings) was reported in 2009.^71^ Different catalytic systems were reported for PA hydrogenation to phthalide, such as acid-tolerant intermetallic cobalt–nickel silicides as noble metal like catalysts,^72^ CoSi_*x*_/CNTs,^73^ hydrophobic activated carbon supported Ni-based acid-resistant catalyst,^74^ and Al_2_O_3_-supported NiCu alloy.^75^

PA is applicable in some other fields as well. Al-Sawaad and Alwaaly research group in 2021 reported bisthioureaphthalatonickel(ii) complex (PTUNi) *via* the reaction of NiCl_2_·6H_2_O with thiourea (2 mol) and PA (1 mol). The complex was evaluated as a corrosion inhibitor for carbon steel alloy (C1010) against a corrosive medium of 0.1 M hydrochloric acid at 298 K.^76^ Fouda group in 2013 also reported anhydride derivatives (such as PA) as corrosion inhibitors for carbon steel in hydrochloric acid solutions.^77^ In 2022, glycerol/PA novel nanocomposite was introduced for microwave absorbing applications.^78^

Velayutham and coworkers in 2009 demonstrated the synthesis and characterization of polyurethane (PUR) coatings derived from polyols synthesized with glycerol, PA, and oleic acid. The utilized polyols were designated as Alk28, Alk40, and Alk65, in which 28%, 40%, and 65% of oleic acid was present, respectively. The coatings obtained from polyol Alk28, with the lowest percentage of oleic acid content, exhibited the best overall physicochemical properties, followed by Alk40. PUR from polyol with the highest percentage of oleic acid content, Pualk65 coatings, were softer and their anticorrosive properties were less satisfactory.^79^ Son in 1975 studied the role of PA in cure retardation of rubbers.^80^

Based on the importance of PA in diverse fields of science and technology, many review articles have been written. Dubey and coworkers in 1996 published a review article about the importance of phthalic anhydride as a petrochemical agent.^81^ In 2002, a review article entitled “Some new applications of phthalic anhydride in organic synthesis” was published by the Iordache research group.^82^ In 2021, Elgharbawy reported a mini-article entitled “A review on phthalic anhydride industry and uses”.^83^ In 2016, Basketter and Kimber discussed about PA as a chemical allergen in detail. They claimed that it displays a differentiated behavior, whereas most respiratory sensitizers are known also to give rise to delayed skin reactions; evidence for PA suggests that it only causes immediate type allergy.^84^ Siddiqui and Javed research group study the computational, spectroscopic, Hirshfeld surface, electronic state, and molecular docking properties (with 21 different protein receptors) of PA.^85^ Hong *et al.* in 2017 investigated the antiinflammatory effect of titrated extract of *Centella asiatica* in PA.^86^ In 1977, a patient with occupational asthma caused by PA was reported.^87^ Yanagimoto in 1956 discussed about the reaction between urea and PA under pressure.^88^ The Martin group in 2015 published a book-chapter entitled “Anhydride-based multicomponent reactions”, which contains some reactions of PA.^89^

## 2. Applications of PA in two-component reactions

**Scheme 1:**
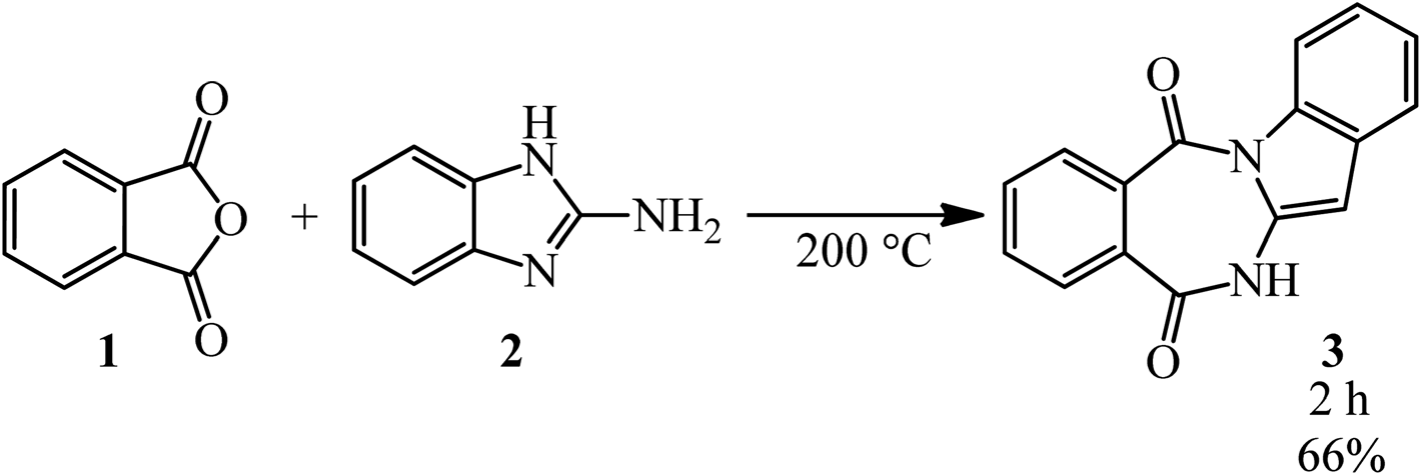
Synthesis of 6*H*-benzimidazo[1,2-*b*][2,4]benzodiazepine-7,12-dione.

Katritzky and Yates in 1976 prepared 6*H*-benzimidazo[1,2-*b*][2,4]benzodiazepine-7,12-dione (3) *via* the catalyst-free reaction of PA (1) and 2-aminobenzimidazole (2) (Scheme 1).^90^

Gitis group in 2000 reported the reaction of PA with 2-methylimidazole that formed amide.^91^ The authors also examined the reaction of maleic anhydride that leads to the formation of molecular complexes.

**Scheme 2:**
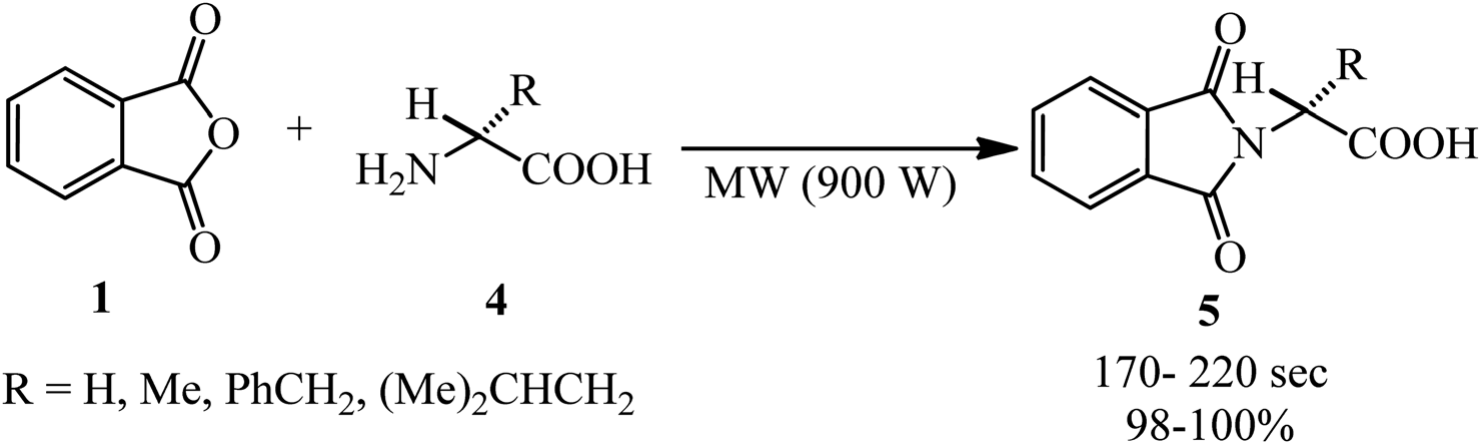
Synthesis of phthalimide derivatives.

Hajipour *et al.* in 2000 interpreted the phthloyation microwave-assisted reaction of PA (1) with amino acids (4) under solvent-free conditions to obtain phthalimide derivatives without racemization (5) (Scheme 2).^92^ The substrates were mixed with silica gel and irradiated with microwave (900 W) for an appropriate time.

Billman and Harting in 1948 obtained the phthalyl derivatives of different kinds of amino acids (5) *via* the reaction of PA (1) with (4) at 180–185 °C within 15 min with 30–92% yield.^93^ Zeng research group in 2004 also reported the *N*-phthaloylation of amino acids (Gly, Ala, Phe, Val, 10 mmol) with PA or phthalic acid (11 mmol) with amino acids at 130–135 °C under pressure (about 40 mmHg) for 15–30 min with 79.4–90.5% yield.^94^ Kidd and King in 1948 also reported the preparation of phthalyl-l-glutamic acid.^95^ Leite *et al.* in 2014 demonstrated the microwave-assisted synthesis of some phthaloyl amino acids from the reaction of PA (1) and amino acids (4, Gly, Ala, Val, Glu, Phe, Ile, and Trp) in a 1 : 1 molar ratio in TEA or 4-DMAP (0.5 mL) and DMF (three drops) within 2 min by 31.9–96%. The products presented antioral inflammatory activity comparable to thalidomide. Most of the compounds effectively suppressed nitric oxide production in murine cells stimulated with lipopolysaccharide.^96^ Ökten and coworkers in 2022 reported the facile, expeditious, and cost-effective preparation of *N*-phthaloyl (*S*)-amino acids and evaluated their *in silico* activities against *Staphylococcus aureus*.^97^

In 1958, the reaction of PA with different amino acids by refluxing in nonpolar solvents (such as benzene and toluene) in the presence of triethylamine was performed. By separating the water formed in the reaction, the phthalimide derivatives were prepared in good yields and without racemization. Phthaloylation without racemization may also be carried out in *N*,*N*-dimethylformamide medium.^98^

Homsi and Kasideh in 2015 obtained *N*-phthalimide amino acids from PA and amino acids (Gly, Ala, Phe, Val, Leu, and Asp) in refluxing glacial AcOH for 2 h with 66.8–95.8% yield. The synthesized compounds, which were purified through recrystallization from ethanol, were screened for their antimicrobial activity against four microorganisms, namely, *Streptococcus epidermidis*, *Escherichia coli*, *Mycobacterium tuberculosis*, and *Candida albicans*.^99^

**Scheme 3:**
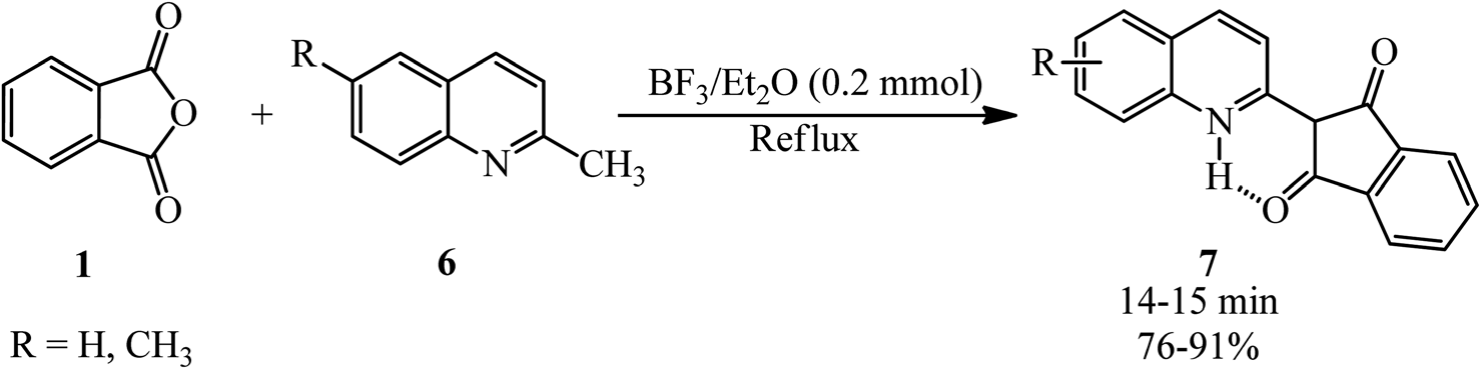
Synthesis of quinophthalones.

Safari *et al.* in 2009 achieved quinophthalone pigments (7) through the reaction of PA (1) and 2-methylquinolines (6) in the presence of BF_3_/Et_2_O as the catalyst under solvent-free and reflux conditions (Scheme 3).^100^

Loghmani-Khouzani in 2004 also gained some quinophthalones (7) *via* the microwave-assisted (700 W) reaction of PA (1) and 2-methylquinolines (6) in a 1 : 1 molar ratio in the presence of silica gel (silica gel 60, 230–240 mesh Merck, 300 mg) within 2 min by 85–97%.^101^

**Scheme 4:**
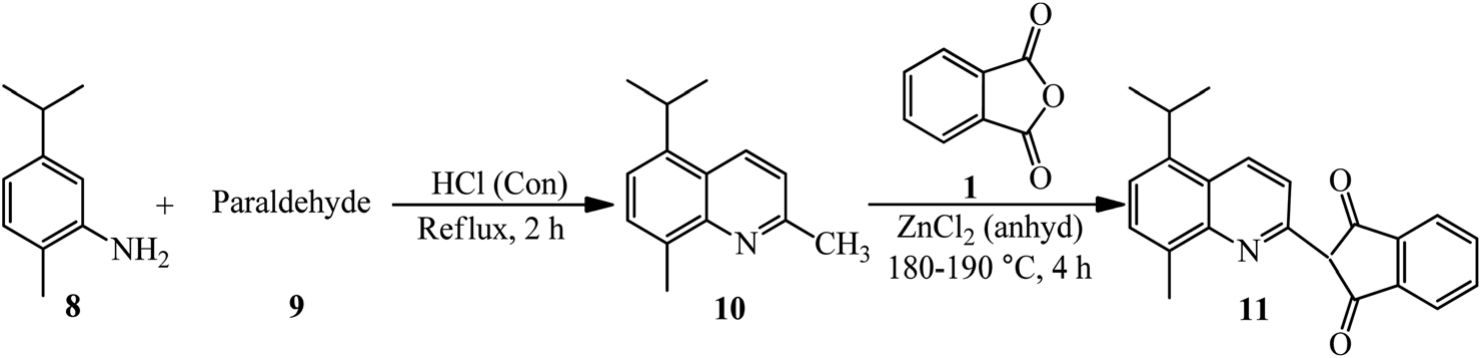
Synthesis of methyl-isopropyl-quinoline yellow.

Phillips and Goss in 1926 obtained methyl-isopropyl-quinoline yellow (11) through the reaction of PA with methylisopropyl-quinaldine (10). The product (10) was also achieved from 2-amino-*p*-cymene (8) and paraldehyde (9) (Scheme 4).^102^

**Scheme 5:**
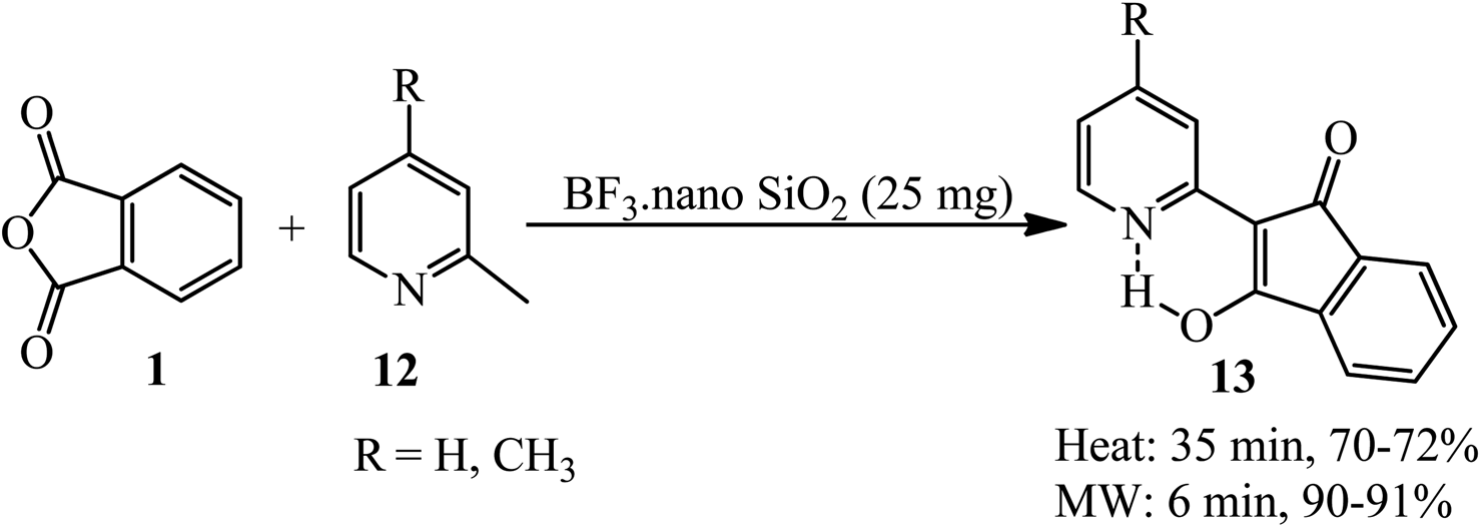
Synthesis of pyrophthalones.

Safari *et al.* in 2012 performed simple regioselective one-pot solvent-free reaction of 2-methylpyridines (12) and PA (1) in the presence of BF_3_·nano SiO_2_ (as a solid supported catalyst) at conventional heating and also under microwave irradiation to prepare the corresponding pyrophthalones (13) (Scheme 5).^103^

**Scheme 6:**
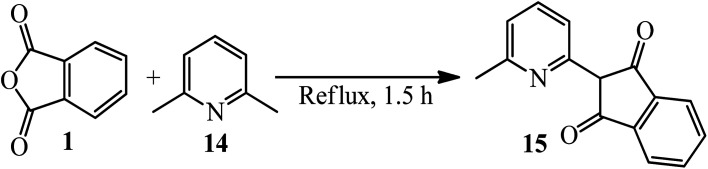
Synthesis of (6-methyl-2-pyridyl)-l,3-indanedione.

Cook and Martin in 1954 acquired orange crystals of 2-(6-methyl-2-pyridyl)-l,3-indanedione (15) *via* the reaction of PA (1) and 2,6-dimethylpyridine (14) in equimolar ratio under reflux conditions within 1.5 h. Their attempts to perform the condensation of both methyl groups through using 2 equimolar of PA were not successful, and only the mono-condensation product (15) as orange crystals was isolated (Scheme 6).^104^ The authors discussed about the chelating tendency of the obtained product with some bivalent-metal ions at 30 °C in a 75% (v/v) dioxane–water solution.

**Scheme 7:**
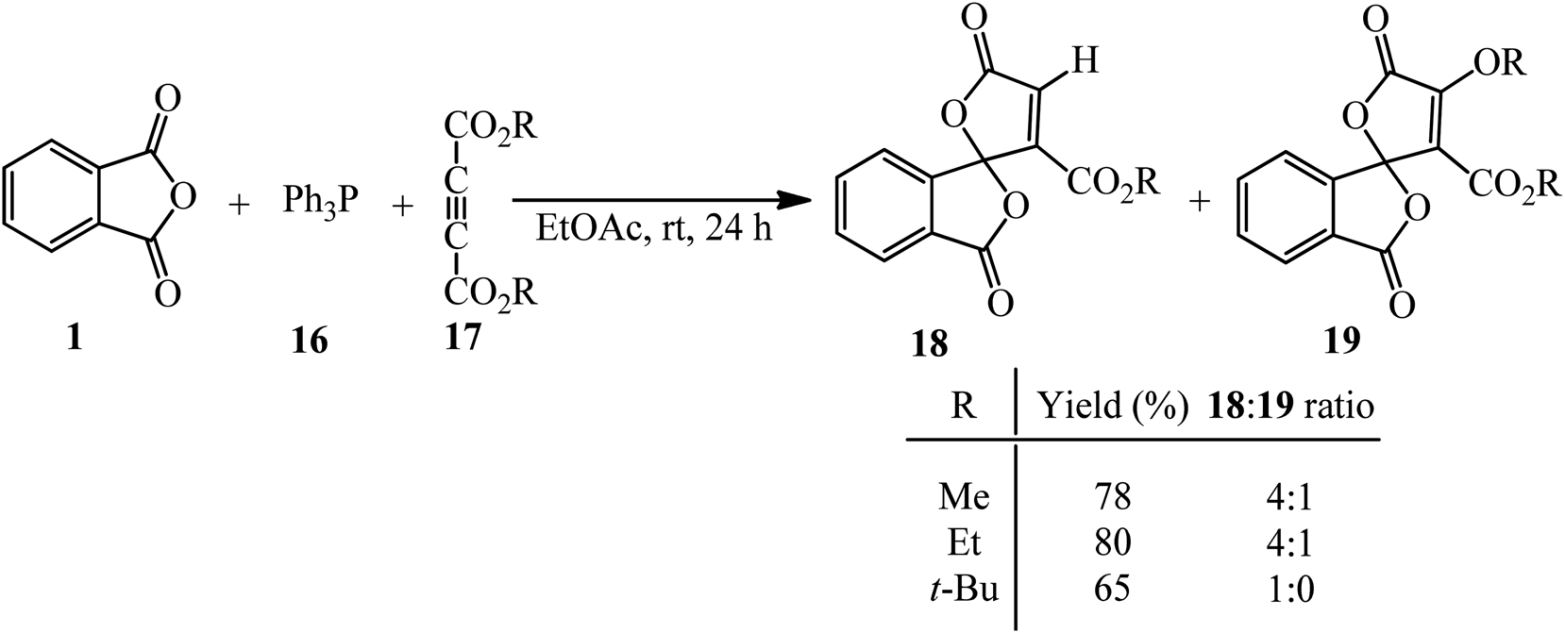
Synthesis of spirocyclic compounds based on PA.

**Scheme 8:**
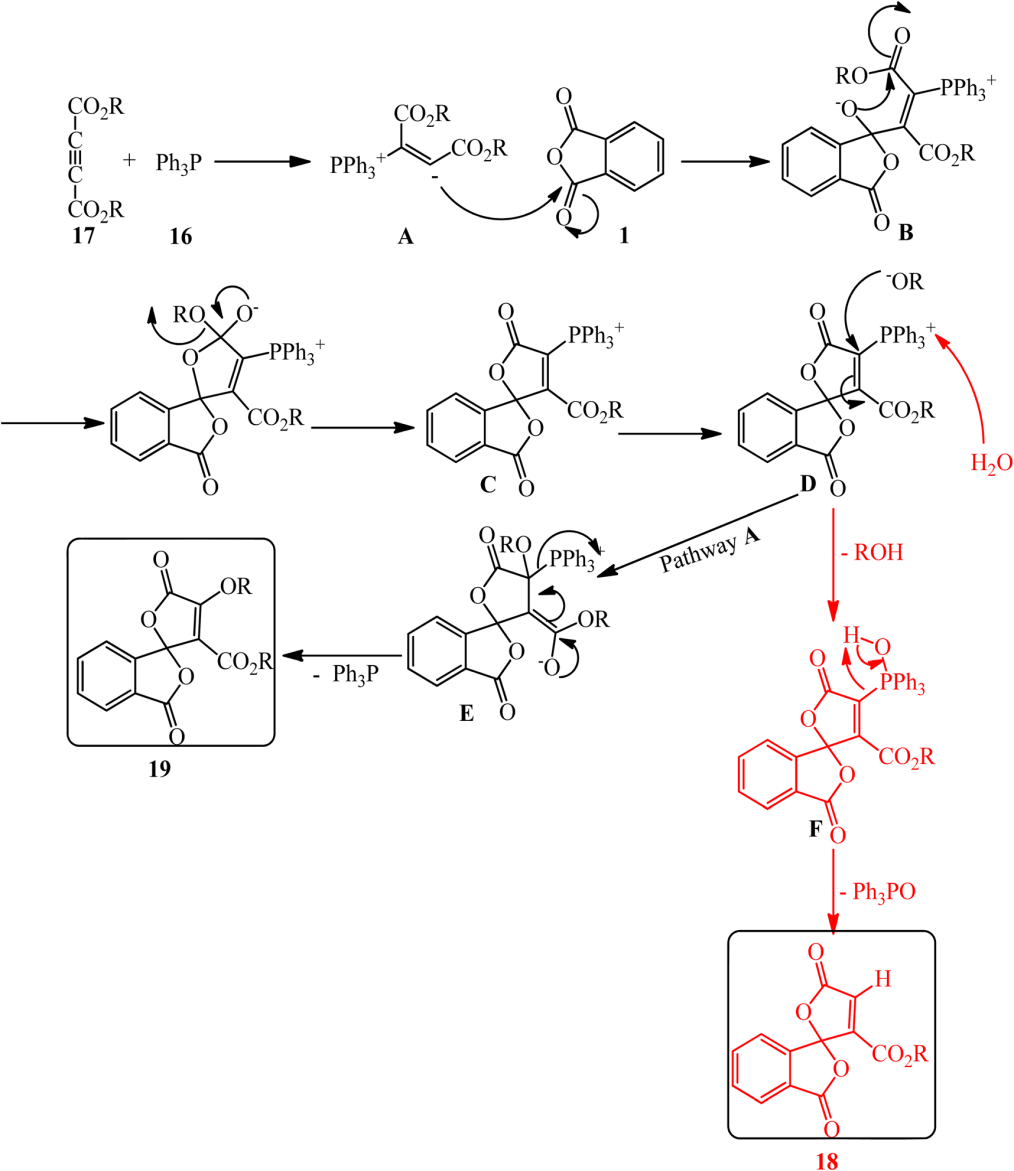
Proposed mechanism of PA-based spirocyclic compounds synthesis.

Bayat *et al.* in 2010 reacted PA (1) and dialkyl acetylenedicarboxylates (17) to afford the novel spirocyclic compounds (18, 19) at room temperature *via* the promoting effect of triphenylphosphine (16) (Scheme 7).^105^ Although Ph_3_P has been utilized in 1 mmol amount, similar to the other two substrates, but according to the proposed mechanism (Scheme 8) and also the products structure, its role is just that of a promoting agent. According to the mechanism, the first step is the formation of 1,3-dipolar intermediate (A), which attacked PA (1) to obtain zwitterionic intermediate (B), which cyclized to the spiro intermediate (D). Water attack to the positively charged phosphorus ion of (D) formed (F), followed by a proton transfer and loss of Ph_3_PO, which led to product (18). In pathway A, intermediate (D) was attacked by the alkoxy anion, and the subsequent loss of Ph_3_P gave compound (19).

**Scheme 9:**
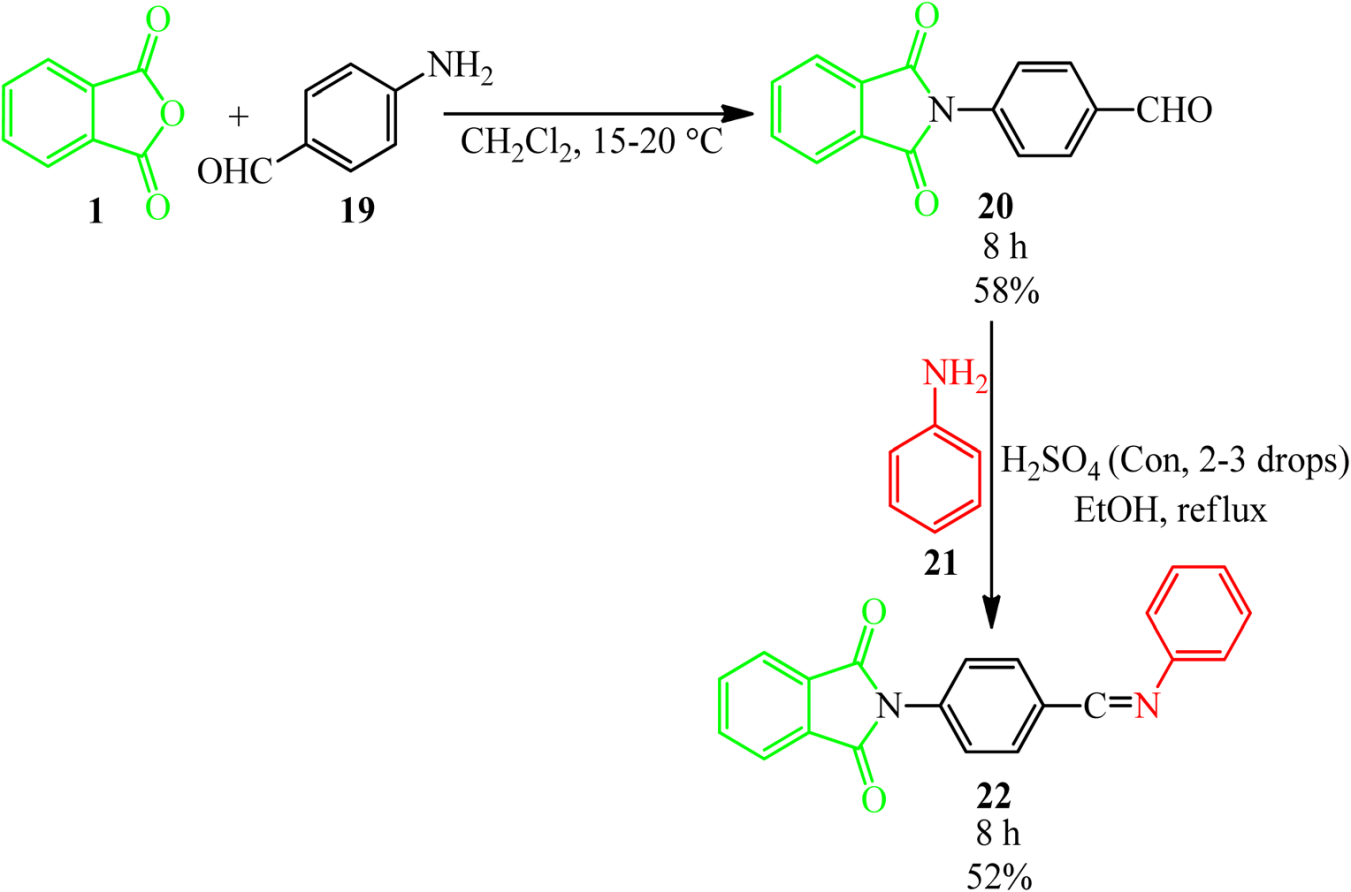
Synthesis of 4-(1,3-dioxoisoindolin-2-yl)benzaldehyde.

Sharma's research group in 2012 synthesized some Schiff bases possessing imide moieties through multistep reactions initiated from the reaction of PA (1) and 4-aminobenzaldehyde (19) in dichloromethane to obtain 4-(1,3-dioxoisoindolin-2-yl)benzaldehyde (20). In the second step (20), reacted with anilines (21) in the presence of glacial acetic acid to obtain the Schiff bases (22), which demonstrated analgesic and antiinflammatory activities (Scheme 9).^106^

**Scheme 10:**
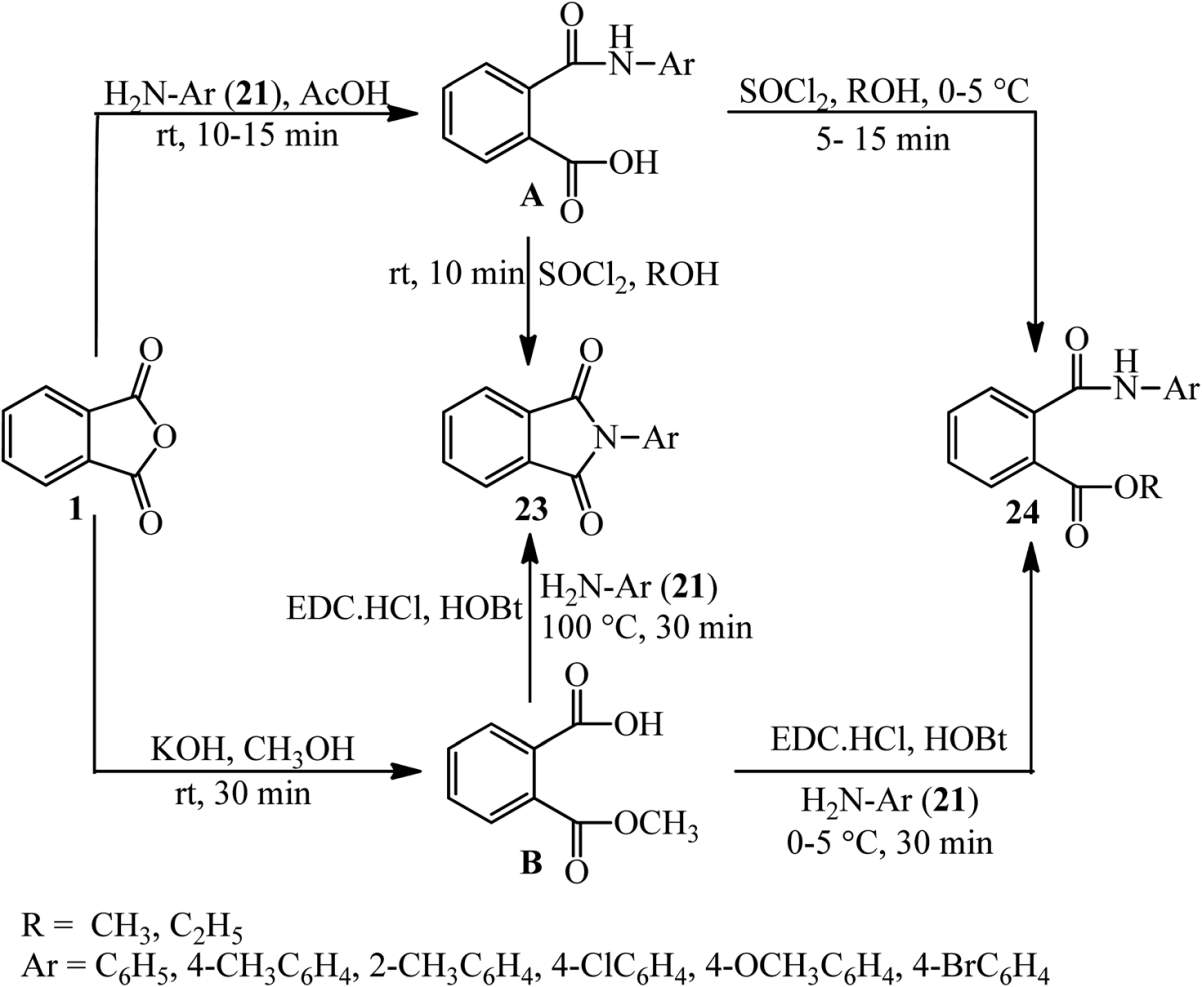
Cyclization of amidic acid esters.

Kumar *et al.* in 2014 described the reaction of PA (1) with amines (21) to obtain monoacid monoamides (A), which get the corresponding cyclic imide derivatives (23) in the presence of SOCl_2_. The PA also reacted with KOH to obtain monomethyl ester (B), which reacted with amines in the presence of 1-ethyl-3-(3-dimethylaminopropyl)-carbodiimid hydrochlorid (DEC·HCl) and *N*-hydroxy benzotriazole (HOBt) to get the cyclic imide derivatives (24) (Scheme 10).^107^

**Scheme 11:**
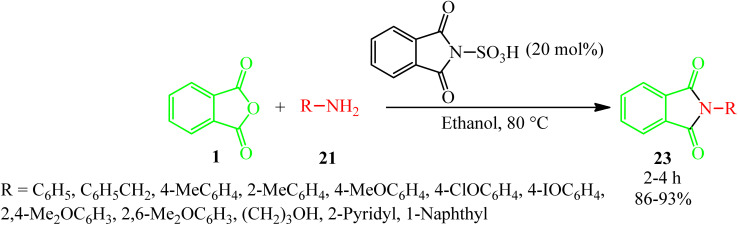
Synthesis of isoindoline-1,3-diones.

Habibi and Mohammadkhani Pordanjani in 2017 developed an efficient and easy catalytic protocol for the preparation of various isoindoline-1,3-diones (23) *via* the reaction of PA (1) with aromatic, aliphatic, and benzylic amines (21), using a catalytic amount of phthalimide *N*-sulfonic acid in ethanol at 80 °C (Scheme 11).^108^

Chia group in 2019 prepared the isoindoline-1,3-diones *via* the reaction of anilines and PA in the presence of “water extract of onion peel ash” (WEOPA) (2 mL) as a green catalytic-solvent system at 80 °C within 8 min by 64–92%.^109^ The WEOPA was recycled and reused for 5 runs without significant activity loss.

Aliabadi's group in 2014 obtained a series of phthalimides (23) through the reaction of PA (1) and anilines (21) in toluene solvent (reflux, 24 h) in the presence of Et_3_N with 21–80% yield. The antiepileptic activity of the products was investigated using two experimental models, namely, maximal electroshock (MES) and pentylenetetrazole (PTZ), and the obtained results were compared with diazepam as the reference drug. The neurotoxicity of the compounds was also evaluated using the rotarod model. The presence of *para*-methoxy substituent in the product showed anticonvulsant activity in the MES (maximal electroshock) model. None of the tested compounds demonstrated acceptable protection in subcutaneous PTZ (pentylenetetrazole) model.^110^

Patel's group in 2022 achieved some new classes of isoindoline-1,3-diones (23) *via* the reaction of PA (1) and the primary amino-containing compounds (21) in refluxing glacial acetic acid as the solvent-catalyst within 3 h with 62–78% yield. The quantum chemistry-based investigations of the products as antimycobacterial agents (toward the *H37Rv* strain by a dual read-out assay method) was also performed. Computational studies such as density functional theory (DFT) study, molecular docking, and dynamic simulation studies illustrated the reactivity and stability of the synthesized compounds as *InhA* inhibitors.^111^

Hamdi *et al.* investigated some phthalimides *via* the reaction of PA (1) and amines (21) (aromatic, aliphatic, and benzylic) in 1 : 1.1 molar ratio in the presence of *p*-TSA (50 mg) in refluxing toluene within 3 h with 29–88% yield. The synthesized compounds were screened for their antimicrobial activities against Gram-positive bacterial strains (*Micrococcus luteus*, *Listeria monocytogenes*, *Staphylococcus aureus*, and *Bacillus cereus*), a Gram-negative bacterial strain (*Salmonella typhimurium*), and a fungus (*Candida albicans*). The cytotoxicity studies of the phthalimides were conducted in two human cancer cell lines, namely, *MDA-MB-231* and *MCF-7*.^112^

Al-Mousawi in 2010 achieved 2-phenylisoindole-1,3-dione (23) *via* the reaction of PA (1) and aniline (21) under solvent-free conditions for 30 min in a focused microwave oven at 160 °C with 96% yield.^113^

Singh Bisht and Rajat Bisht in 2021 reported the reaction of PA with some amines (such as urea, glycine, aniline, and sulphanilic acid) to yield various phthalimide derivatives (23) using domestic microwave with 70.7–80.21% yield. All synthesized derivatives were subjected to DDPH scavenging activity, which showed good to high antioxidant potential (69.56%) in the presence of ascorbic acid as the standard.^114^

**Scheme 12:**
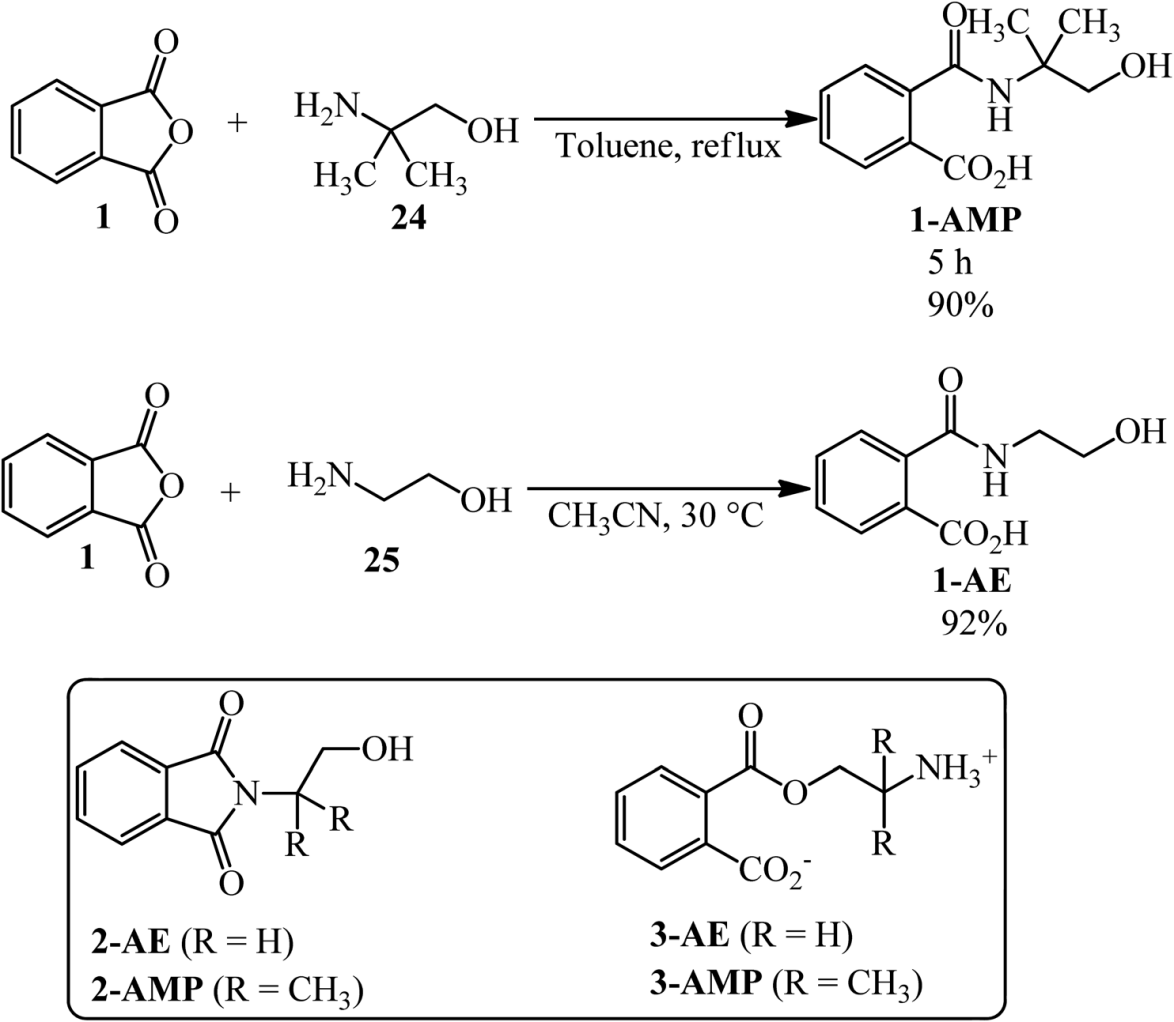
Reaction of PA with 2-amino-2-methyl-1-propanol (AMP) and 2-aminoethanol (AE).

Wicks and Chen in 1979 mixed equimolar amounts of PA with two kinds of alcohols. First, the reaction of PA (1) and 2-amino-2-methyl-1-propanol (AMP) (24) yielded amide 1-AMP, imide 2-AMP, and/or ester 3-AMP. Amide 1-AMP undergoes an acyl shift to ester 3-AMP, apparently representing the first example of an *N*- to *O*-acyl shift in the absence of strong acid. Second, the reaction of PA (1) and 2-aminoethanol (AE) (25) yielded only amide 1-AE or imide 2-AE. No acyl shift of amide 1-AE to ester 3-AE was detected in this case (Scheme 12).^115^

**Scheme 13:**
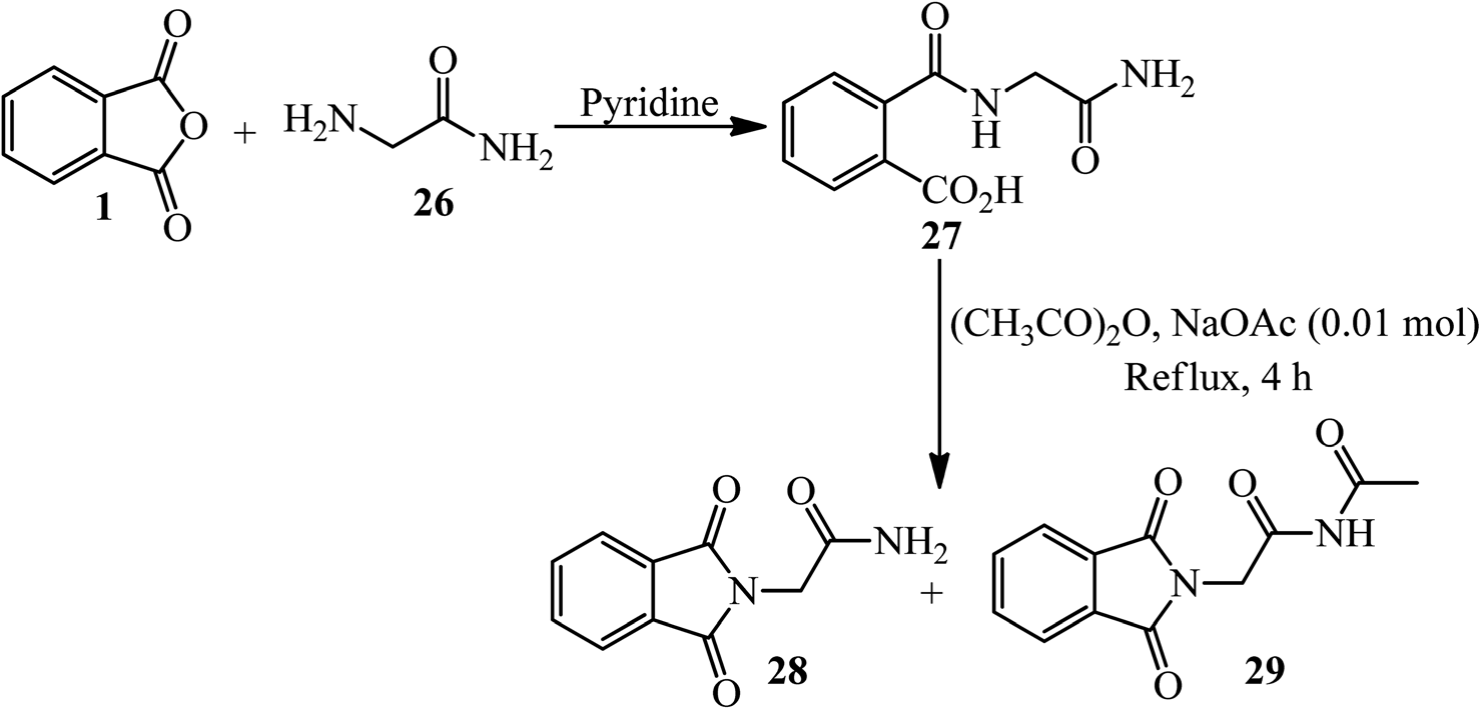
Synthesis of imides.

Hassanzadeh's group in 2017 supplied cyclic imides. In the first step, PA (1) reacted with glycinamide (26) in freshly distilled and dried pyridine under reflux conditions to yield the corresponding amic acid (27) within 5 h. The amic acid underwent ring closure with acetic anhydride and anhydrous sodium acetate to form imides (28, 29), which were isolated *via* column chromatography (Scheme 13).^116^

**Scheme 14:**
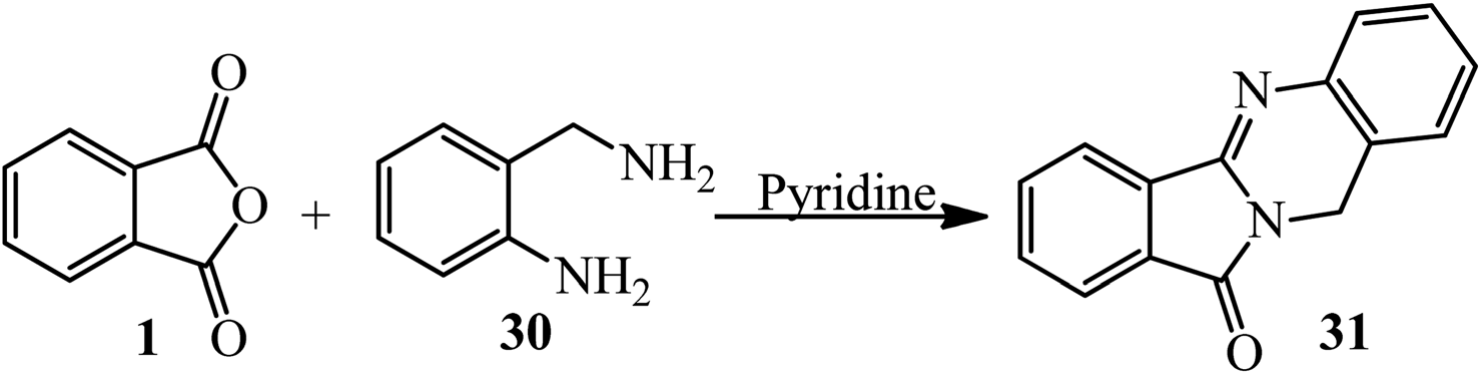
Synthesis of cyclic imides.

In another procedure, the reaction of PA and 2-amino-benzylamine (30) in pyridine within 5 h under reflux conditions gave the corresponding cyclic imide (31) (Scheme 14).^116^ The imides were screened for their antimicrobial activities against three types of bacteria and one type of fungi. Phthalimide derived from benzylamine exhibited remarkable antimicrobial activity against *E. coli*.^109^

**Scheme 15:**
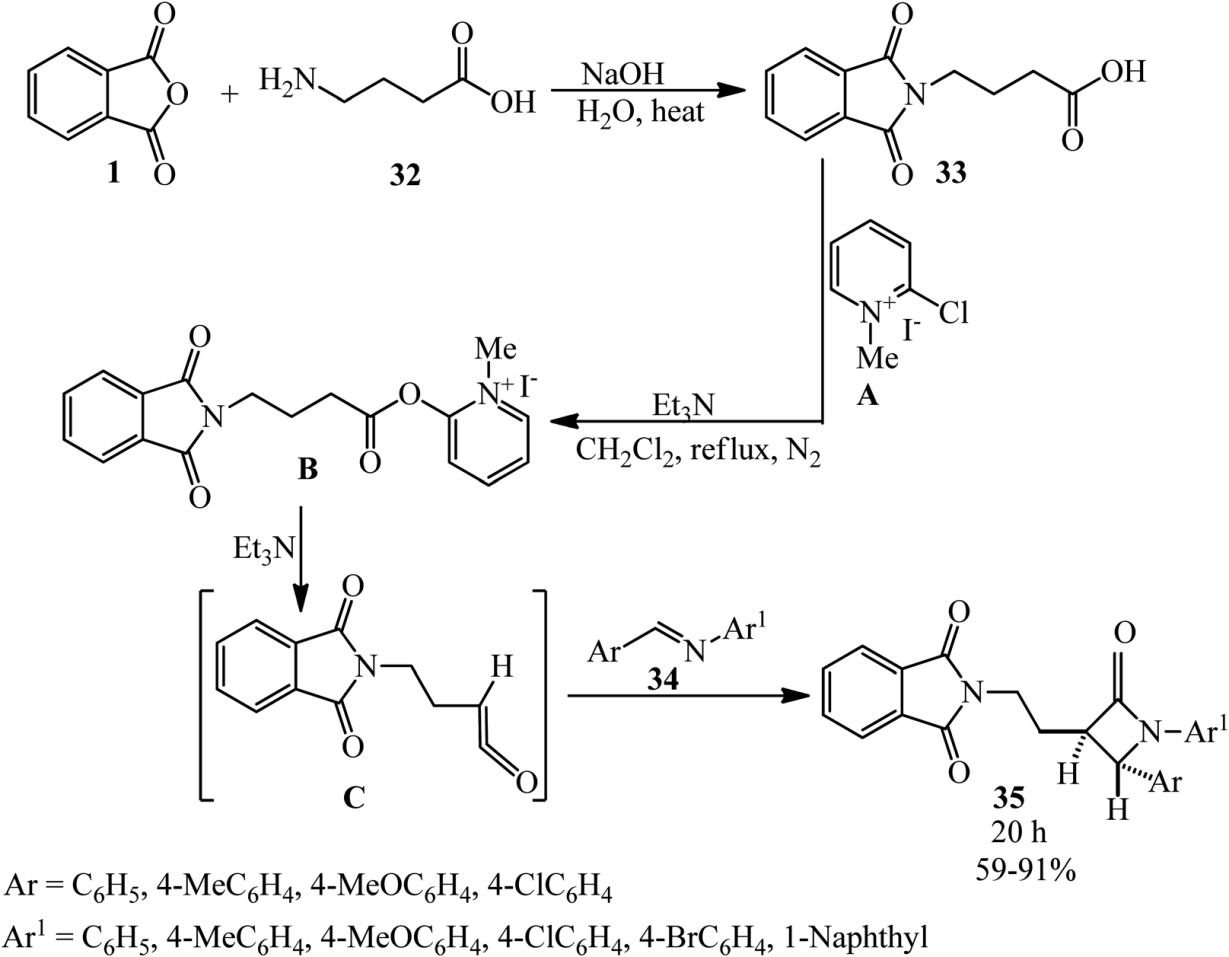
Stereoselective synthesis of 1,4-diaryl-3-[2-(2*H*-isoindol-2-yl)ethyl]azetidine-2-ones.

Islami *et al.* in 2015 obtained 4-(2*H*-isoindol-2-yl)butanoic acid (33) by the reaction of PA (1) with 4-aminobutanoic acid (32), which was followed by the reaction with Mukaiyama's reagent (2-chloro-1-methylpyridinium iodide, A) in the presence of triethylamine in dichloromethane. In the second step, novel diaryl-3-[2-(2*H*-isoindol-2-yl)ethyl]azetidin-2-ones (35) was synthesized *via* the reaction of 4-(2*H*-isoindol-2-yl)butanoic acid (33) with aromatic imines (34). The reaction proceeded through the *in situ* generation of a novel ketene containing an isoindole ring (C) and the subsequent electrocyclic reaction of a zwitterionic intermediate. The reaction was found to be highly stereoselective, and the *trans*-isomers of (E) were obtained as the only products (Scheme 15).^117^

**Scheme 16:**
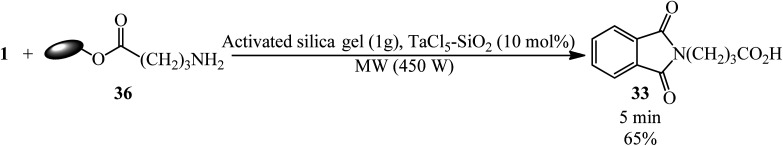
Synthesis of 4-(1,3-2,3-dihydro-1*H*-2-isoidolyl)butanoic acid.

Chandrasekhar and coworkers in 2009 identified (33) as an *N*-alkyl imide of PA from a microwave-assisted (450 W) procedure of polymer bound γ-amino butyric acid (36) (1 mmol) and PA (1.25 mmol) in the presence of activated silica gel and TaCl_5_–SiO_2_ (Scheme 16).^118^ The organic product was isolated from the resin–silica mixture using TFA.

**Scheme 17:**
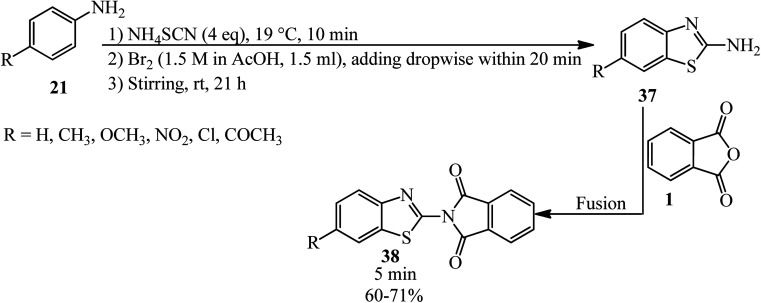
Synthesis of 2-(6-substituted benzo[*d*]thiazol-2-yl)isoindoline-1,3-diones.

Pawar's group in 2014 synthesized 2-(6-substituted benzo[*d*]thiazol-2-yl)isoindoline-1,3-diones (38) from the heat-assisted reaction of PA and 6-substituted 2-amino benzothiazoles (37) within 5 min (Scheme 17).^119^ The 2-amino benzothiazoles (37) were prepared as using a previously reported method.^120^ The antiinflammatory activity of the synthesized compounds was determined using carrageenan-induced rat paw edema method (a mechanistic model for *in vivo COX-2* inhibition). Compounds containing the Cl and COCH_3_ substituents showed antiinflammatory activity comparable to the reference drug diclofenac at 100 mg kg^−1^ doses.

**Scheme 18:**
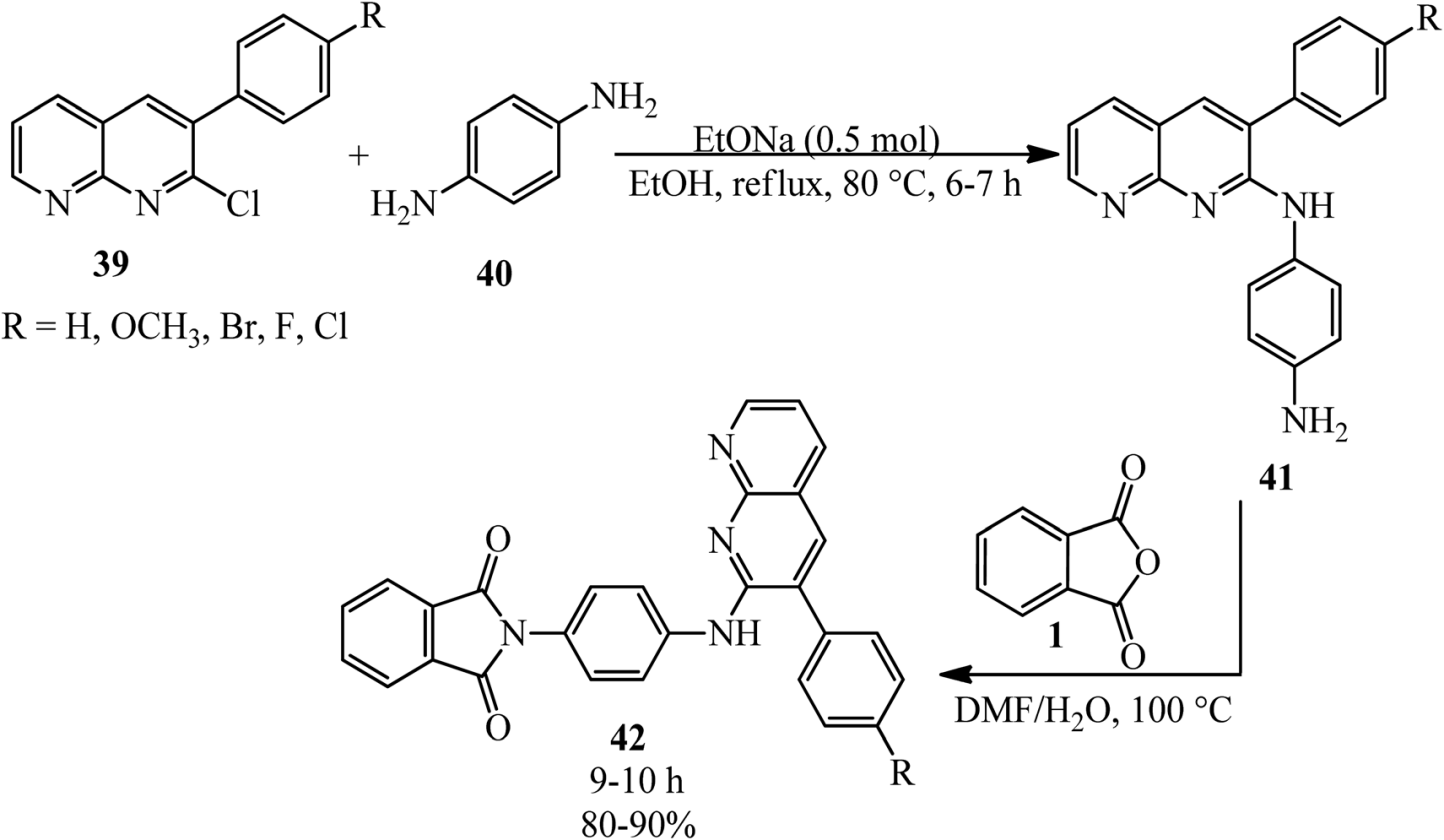
2-(4-((3-Aryl-1,8-naphthyridin-2-yl)amino)phenyl)isoindoline-1,3-diones synthesis.

2-(4-((3-Aryl-1,8-naphthyridin-2-yl)amino)phenyl)isoindoline-1,3-diones (42) was obtained by the treatment of substituted 3-aryl-1,8-naphthyridines (41) with PA in refluxing *N*,*N*-dimethylformamide containing 5% (v/v) water by Sakram group in 2018 (Scheme 18).^121^ Some of the products demonstrated moderate to good antimicrobial activity compared with the streptomycin reference.

**Scheme 19:**
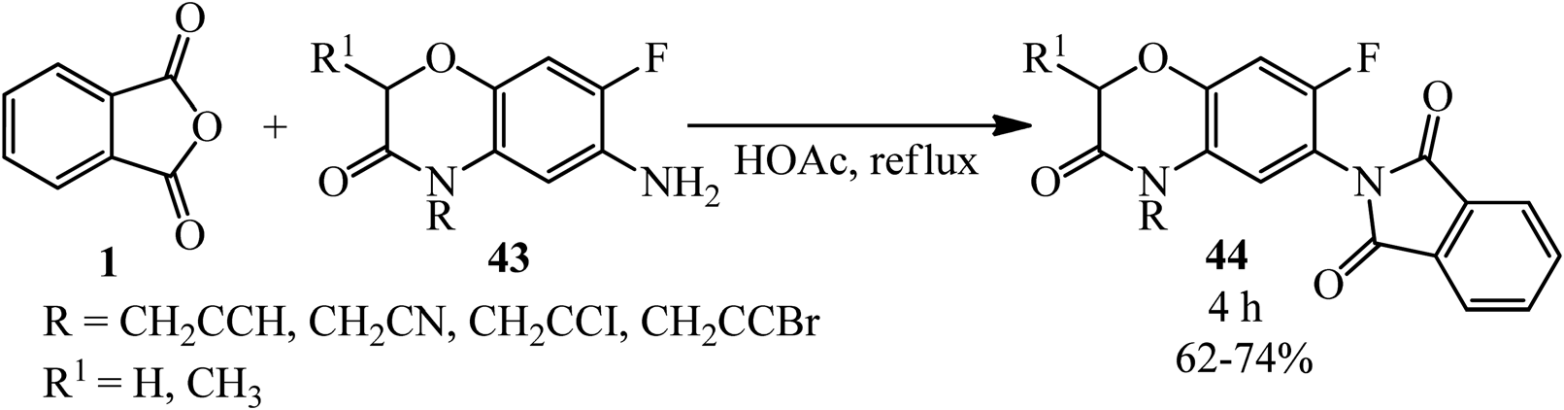
Synthesis of 2-(7-fluoro-3-oxo-3,4-dihydro-2*H*-benzo[*b*][1,4]oxazin-6-yl)isoindoline-1,3-diones.

The target compounds 2-(7-fluoro-3-oxo-3,4-dihydro-2*H*-benzo[*b*][1,4]oxazin-6-yl)isoindoline-1,3-diones (44) were constructed by Huang's group in 2005 *via* the reaction of PA (1) with 6-amino-7-fluoro-4-substituted-2*H*-3(4*H*)-benzo[*b*][1,4]oxazinones (43) in refluxing acetic acid within 4 h (Scheme 19).^122^ The products demonstrated protoporphyrinogen oxidase (protox) inhibitory properties. The preliminary bioassay data displayed that some of them possessed promising herbicidal activities comparable to that of the lead compound *B2055* (flumioxazin and its iodo analogue).

**Scheme 20:**
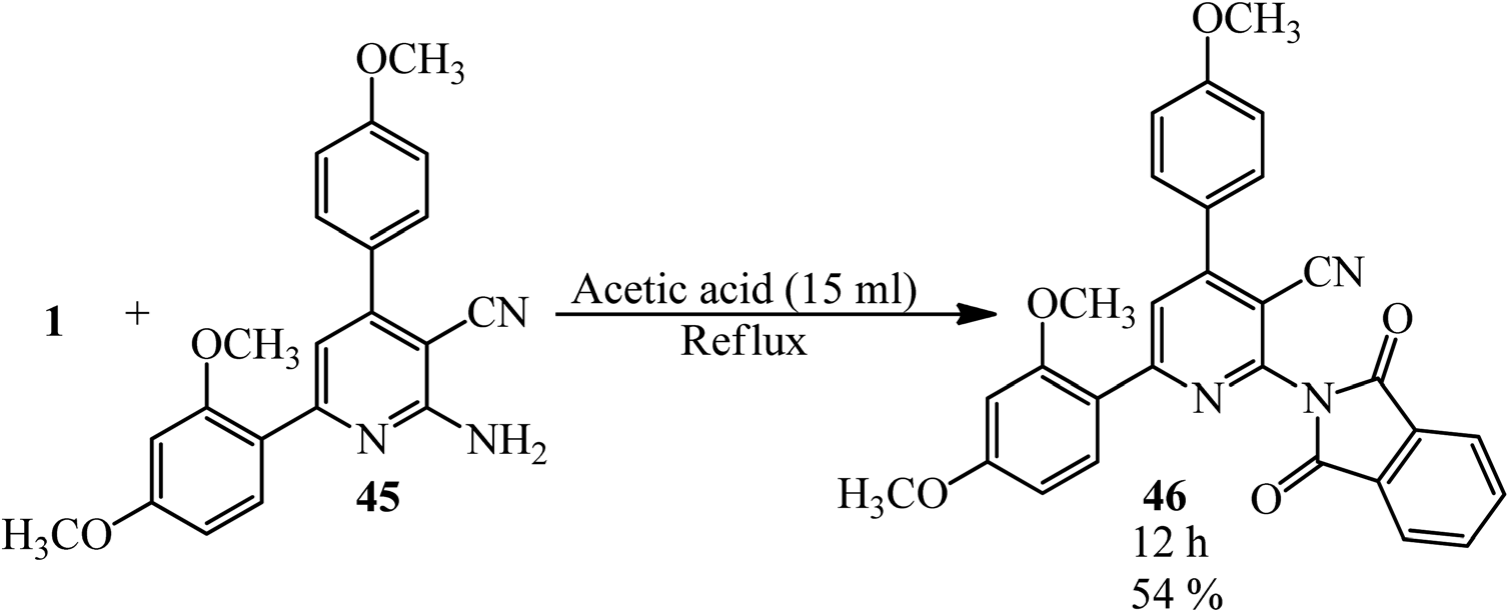
Synthesis of 6-(2,4-dimethoxyphenyl)-2-(1,3-dioxoisoindolin-2-yl)-4-(4-methoxyphenyl)nicotinonitrile.

Anwer *et al.* in 2019 manufactured nicotinonitriles attached to 1,3-dioxoisoindoline ring (46) *via* the reaction of PA (1) and 2-amino-6-(2,4-dimethoxyphenyl)-4-(4-methoxyphenyl)nicotinonitrile (45) in the presence of acetic acid that acted as the catalyst and solvent (Scheme 20).^123^

Shaban *et al.* in 2020 also synthesized a new derivative of (46) named 2-(1,3-dioxoisoindolin-2-yl)-4-(4-methoxyphenyl)-6-phenylnicotinonitrile *via* the rection of PA and 2-amino-4-(4-methoxyphenyl)-6-phenylnicotinonitrile in acetic acid under reflux conditions (22 h, 88%) and also microwave irradiation (3 min, 90.4%).^124^

**Scheme 21:**
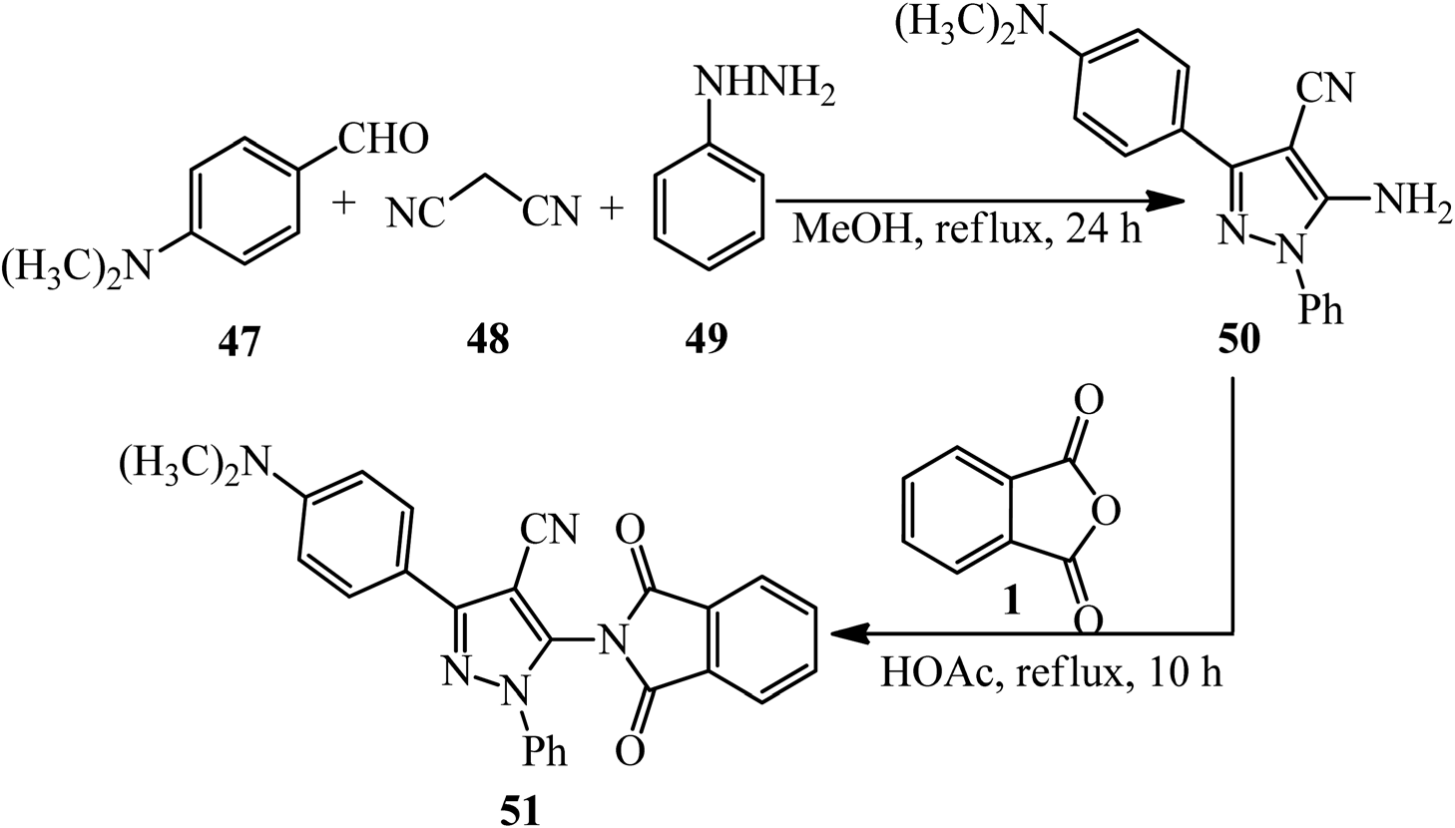
Synthesis of 3-(4-(dimethylamino)phenyl)-5-(1,3-dioxoisoindolin-2-yl)-1-phenyl-1*H*-pyrazole-4-carbonitrile.

Anwer and Sayed in 2020 reported the reaction of PA (1) and 5-amino-3-(4-(dimethylamino)phenyl)-1-phenyl-1*H*-pyrazole-4-carbonitrile (50) to obtain 3-(4-(dimethylamino)phenyl)-5-(1,3-dioxoisoindolin-2-yl)-1-phenyl-1*H*-pyrazole-4-carbonitrile (51). The adduct demonstrated high activities against Gram-negative and Gram-positive bacteria and high cytotoxic activity against *MCF7* and *HCT-116* (Scheme 21).^125^ The pyrazole-containing substrate (50) was achieved *via* the one-pot coupling reaction of *N*,*N*-dimethylamino benzaldehyde (47), malononitrile (48), and phenyl hydrazine (49).

**Scheme 22:**
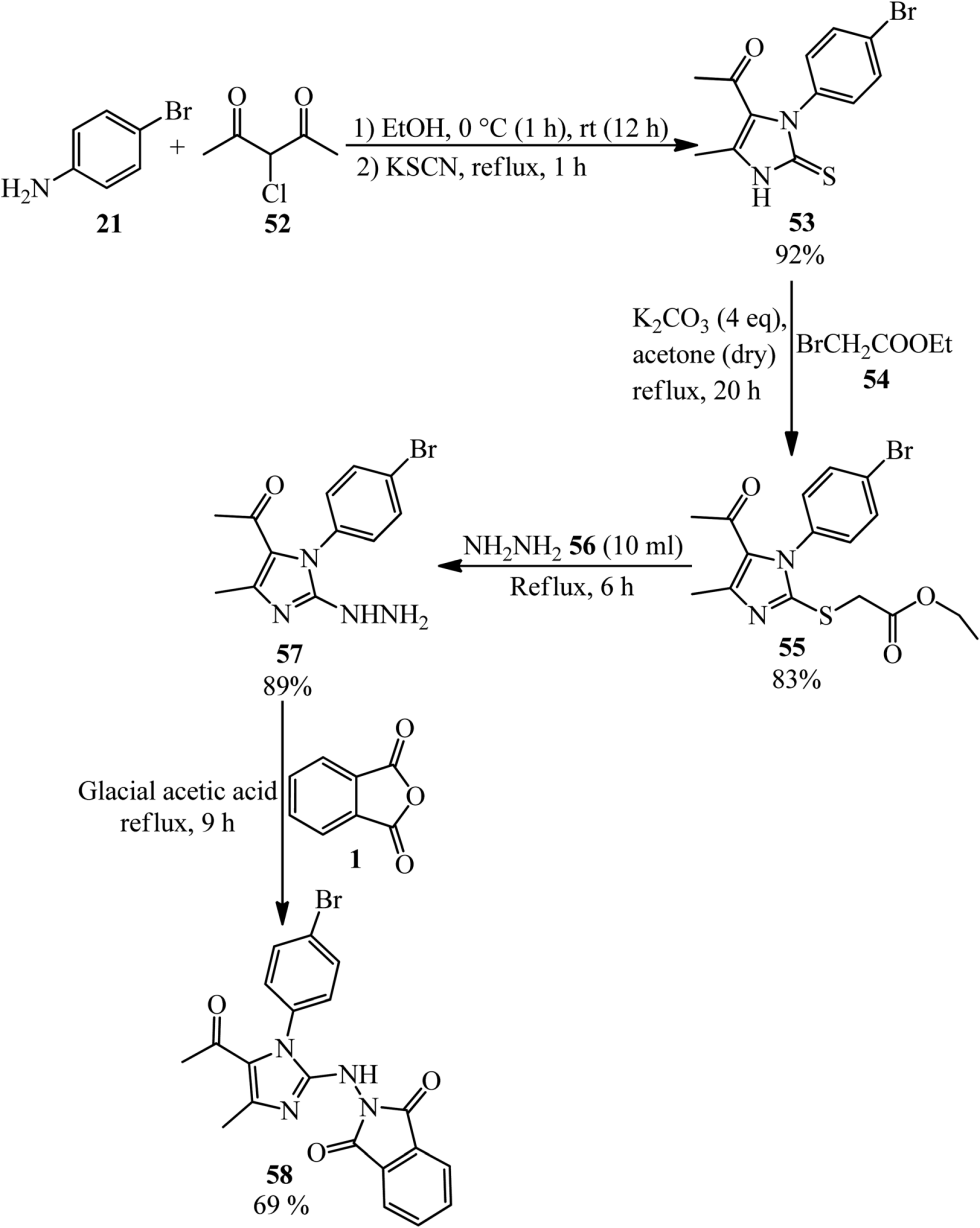
Synthesis of 2-((5-acetyl-1-(4-bromophenyl)-4-methyl-1*H*-midazole-2-yl) amino)isoindoline-1,3-dione.

El-Shahat and Hasanin group in 2021 fabricated novel imidazole isoindoline-1,3-dione (58) from the reaction of PA with 1-(1-(4-bromophenyl)-2-hydrazinyl-4-methyl-1*H*-imidazol-5-yl)ethenone (57) (Scheme 22).^126^ According to Scheme 21, 1-(3-(4-bromophenyl)-5-methyl-2-thioxo-2,3-dihydro-1*H*-imidazol-4-yl)ethan-1-one (53) was obtained through the dropwise addition of 3-chloro-2,4-pentanedione (52) to a solution of 4-bromoaniline (21) in ethanol, followed by the addition of equimolar amount of KSCN. Subsequent alkylation of (53) with ethyl bromoacetate (54) led to the corresponding ethyl ester (55), which fused with hydrazine hydrate (56) to yield (57).

**Scheme 23:**
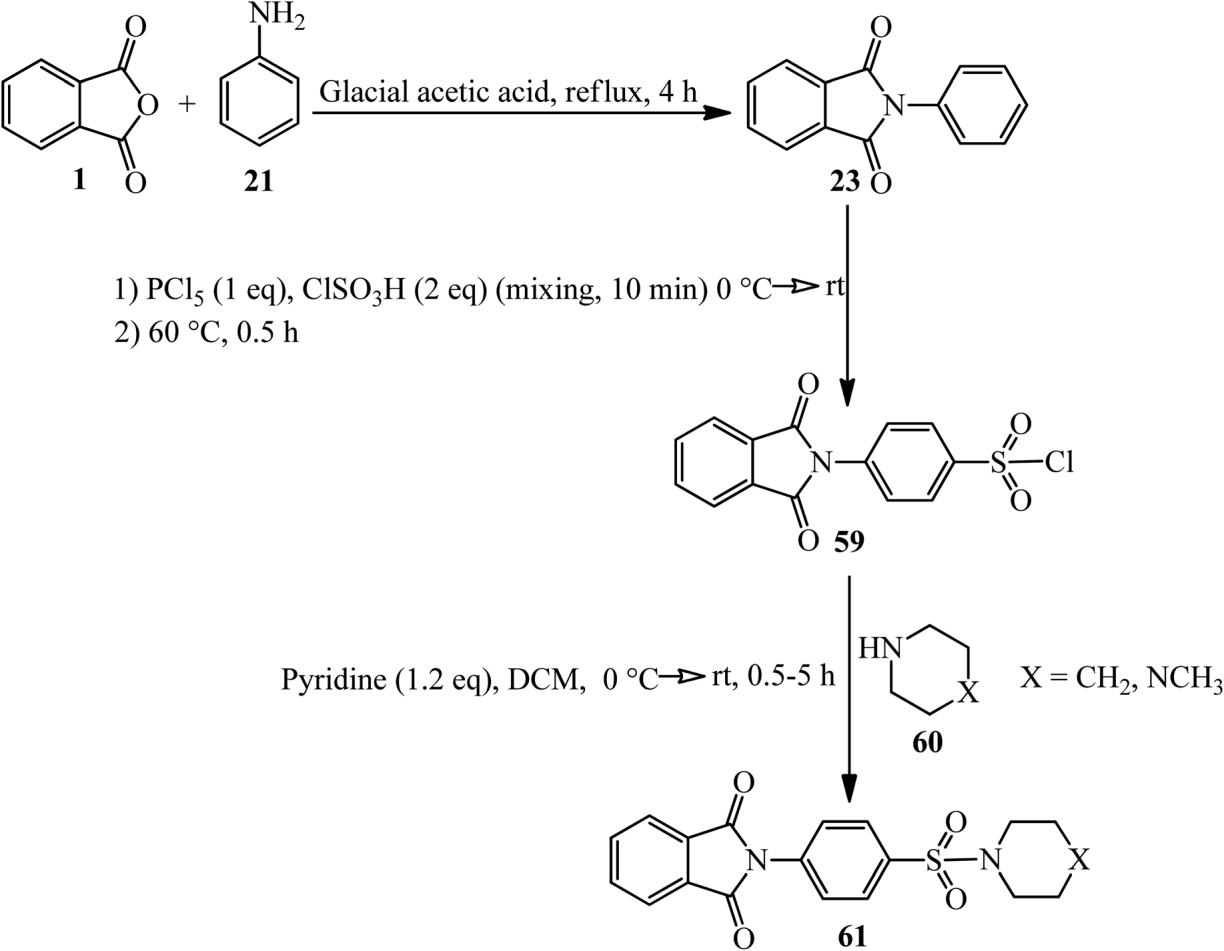
Synthesis of 4-(1,3-dioxo-2,3-dihydro-1*H*-isoindol-2-yl)benzene-1-sulphonamides.

The Jayaprakash group in 2015 constructed some novel 4-(1,3-dioxo-2,3-dihydro-1*H*-isoindol-2-yl)benzene-1-sulphonamides (61). As shown in Scheme 22, in the first step, the 2-phenyl-1,3-isoindolinedione (23) was obtained by the reaction of PA (4 mmol) and aniline (3.4 mmol) in glacial acetic under nitrogen atmosphere at 120 °C within 2 h. In the next step, the product reacted with a mixture of chlorosulfonic acid (2 eq.) and phosphorus pentachloride (1 eq.) at 60 °C for 30 min. These two steps were performed based on previously-reported procedures.^127^ The final products were obtained *via* the reaction of 4-(1,3-dioxoisoindolin-2-yl)benzene-1-sulfonyl chloride (59) and piperidine/1-methylpiperazine (60) in the presence of pyridine with 68–95% yield (Scheme 23).^128^ The authors carried out molecular modelling studies on the adducts as Dengue virus 2 *(DENV2) protease* inhibitors.

**Scheme 24:**
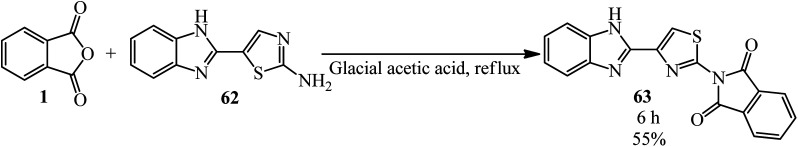
2-(4-(1*H*-Benzo[*d*]imidazol-2-yl)thiazol-2-yl)isoindoline-1,3-dione synthesis.

Nofal group in 2014 reacted the starting compound [4-(1*H*-benzo[*d*]imidazol-2-yl)thiazol-2-amine] (62) with PA in refluxing glacial acetic acid for 6 h to obtain 2-(4-(1*H*-benzo[*d*]imidazol-2-yl)thiazol-2-yl)isoindoline-1,3-dione (63) with 55% yield (Scheme 24).^129^

**Scheme 25:**
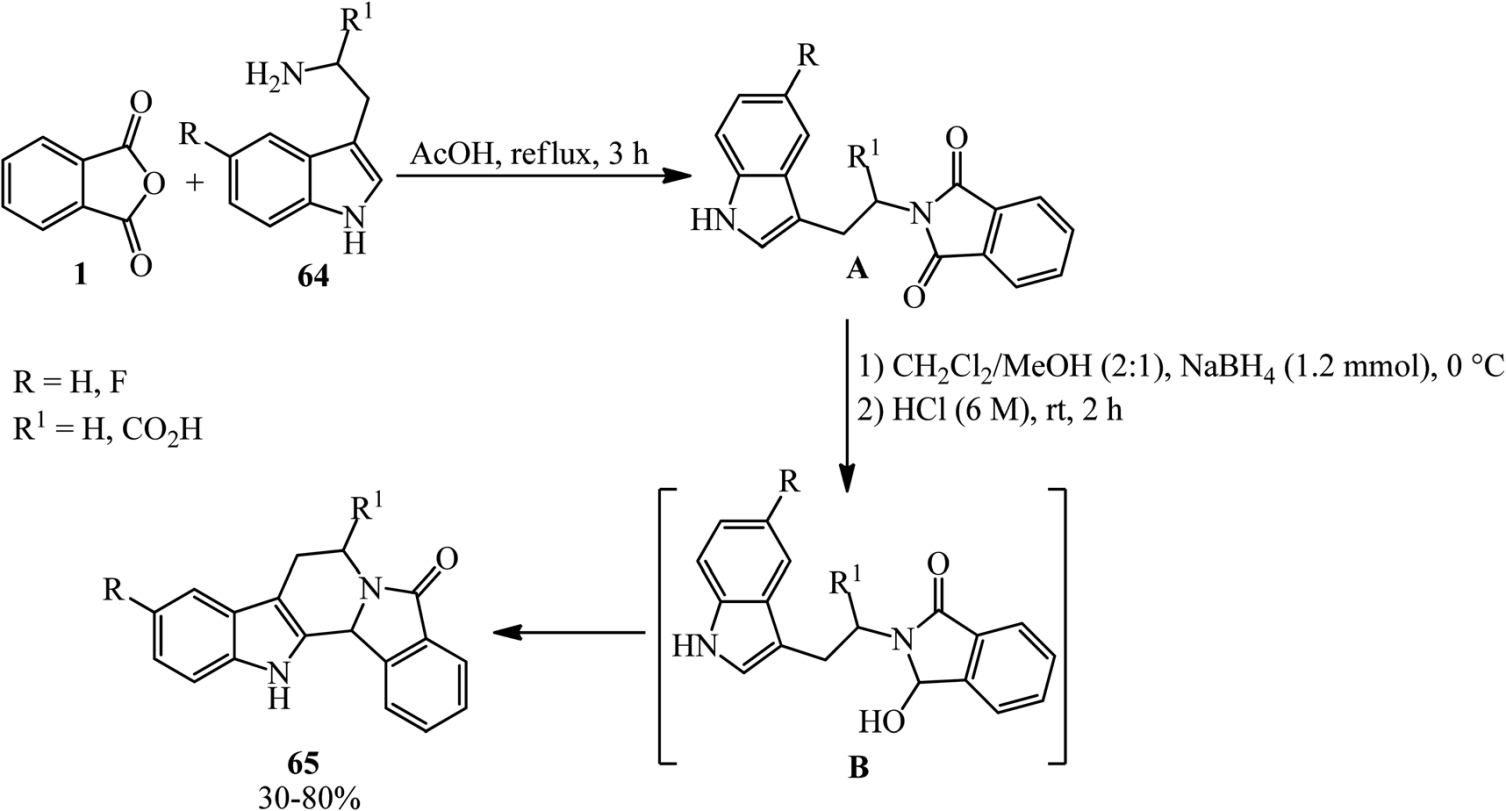
Synthesis of tetrahydro-β-carbolines.

Tetrahydro-β-carbolines (65), with strictosamide skeleton, were obtained by Liu in 2014 *via* intermolecular condensation, selective reduction, and intramolecular cyclization starting from PA (1) and tryptamines (64) in a 1 : 1.2 molar ratio (Scheme 25).^130^ The reaction proceeds *via* the intermediate (B), which has not been isolated.

**Scheme 26:**
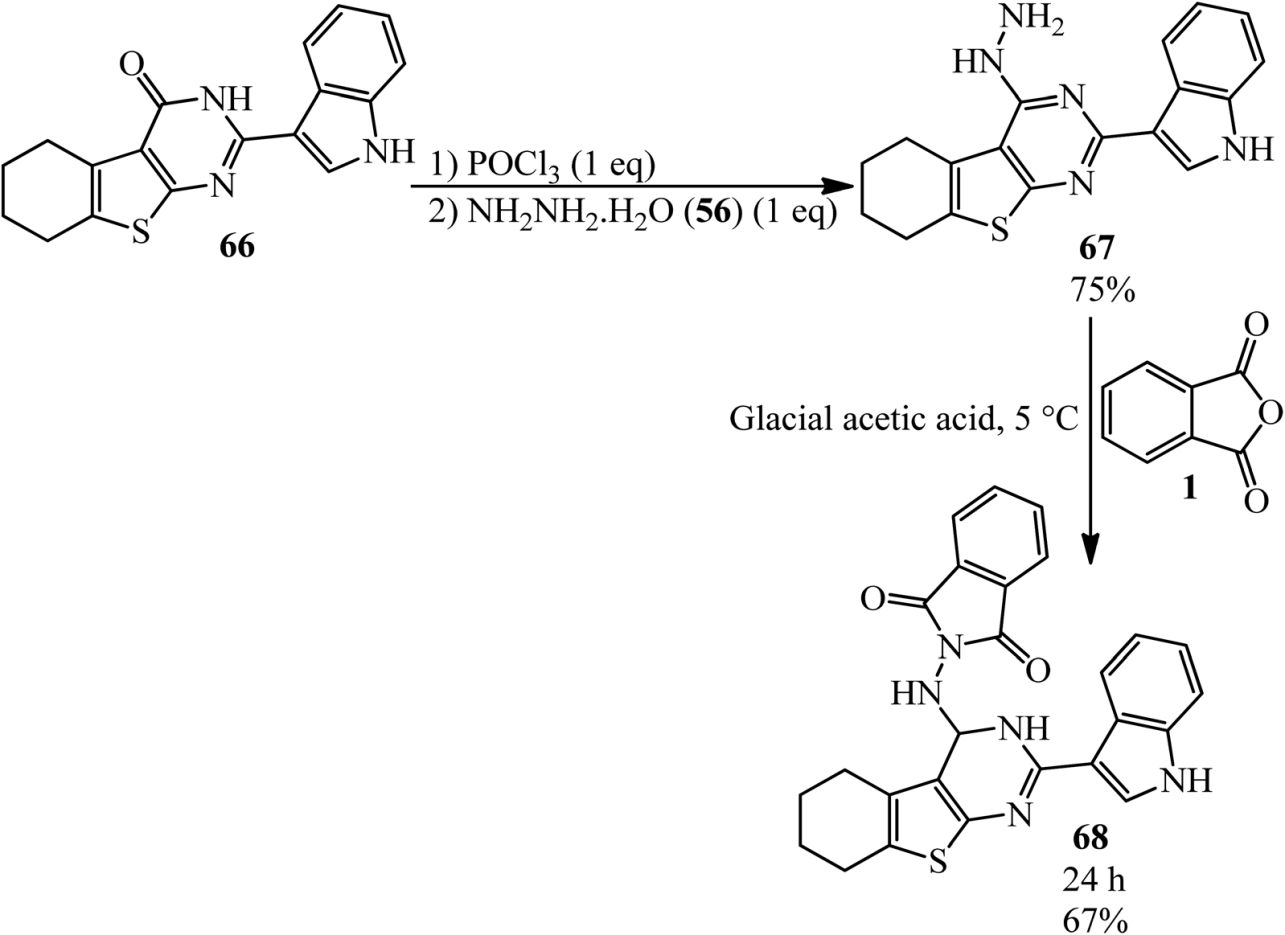
A novel isoindoline-1,3-dione synthetic procedure.

Abd El-All's research group in 2016 constructed 2-((2-(1*H*-indol-3-yl)-3,4,5,6,7,8-hexahydrobenzo[4,5]thieno[2,3-*d*]pyrimidin-4-yl)amino)isoindoline-1,3-dione (68) *via* the reaction of pyrimidinohydrazine (67) and PA. The target compound was tested for its activity against Influenza A Neuraminidase virus (*H3N2*), which was very potent (Scheme 26).^131^

**Scheme 27:**
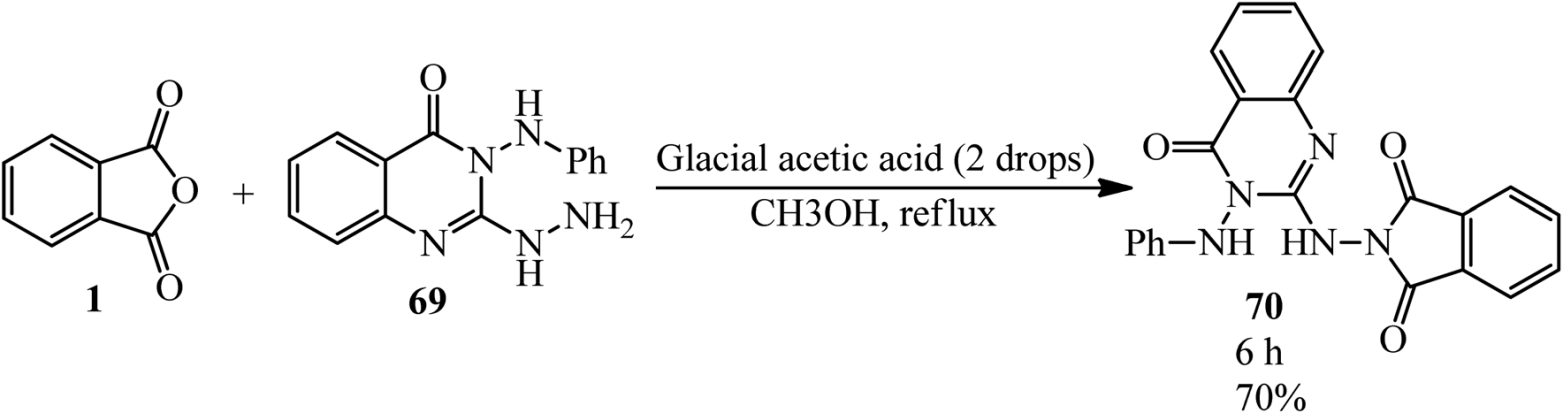
2-(4-Oxo-3-phenylamino-3,4-dihydroquinazolin-2-ylamino)-isoindole-1,3-dione synthesis.

In 2003, Saleh's group constructed 2-(4-oxo-3-phenylamino-3,4-dihydroquinazolin-2-ylamino)-isoindole-1,3-dione (70) by the reaction of PA with 2-hydrazinyl-3-(phenylamino)quinazolin-4(3*H*)-one (69) in refluxing methanol (Scheme 27).^132^

**Scheme 28:**
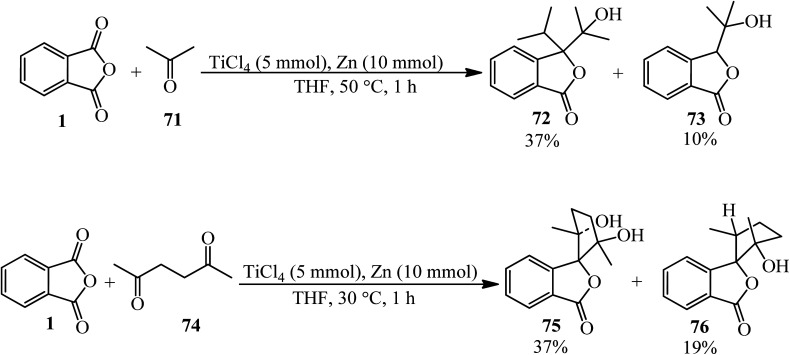
Reductive coupling of PA and ketones.

Kise *et al.* in 2020 presented the reductive coupling of PA (1) with acetone (71) in a 1 : 10 molar ratio by Zn–TiCl_4_ (in 2 : 1 molar ratio in THF) which, gave two-to-one (72) as the major and one-to-one (73) as the minor coupled products. The coupled products were transformed to 3,3-diisopropyl-, 3-isopropylidene-, and 3-isopropylphthalides. In addition, the reductive coupling of PA (1) with acetonylacetone (74) in 1 : 5 molar ratio by Zn–TiCl_4_ in THF gave 3-spirocyclopentanylphtalides as the selective *exo-cis* isomers (75, 76) (Scheme 28).^133^ Zn–TiCl_4_ was utilized as the source of low-valent titanium.

**Scheme 29:**
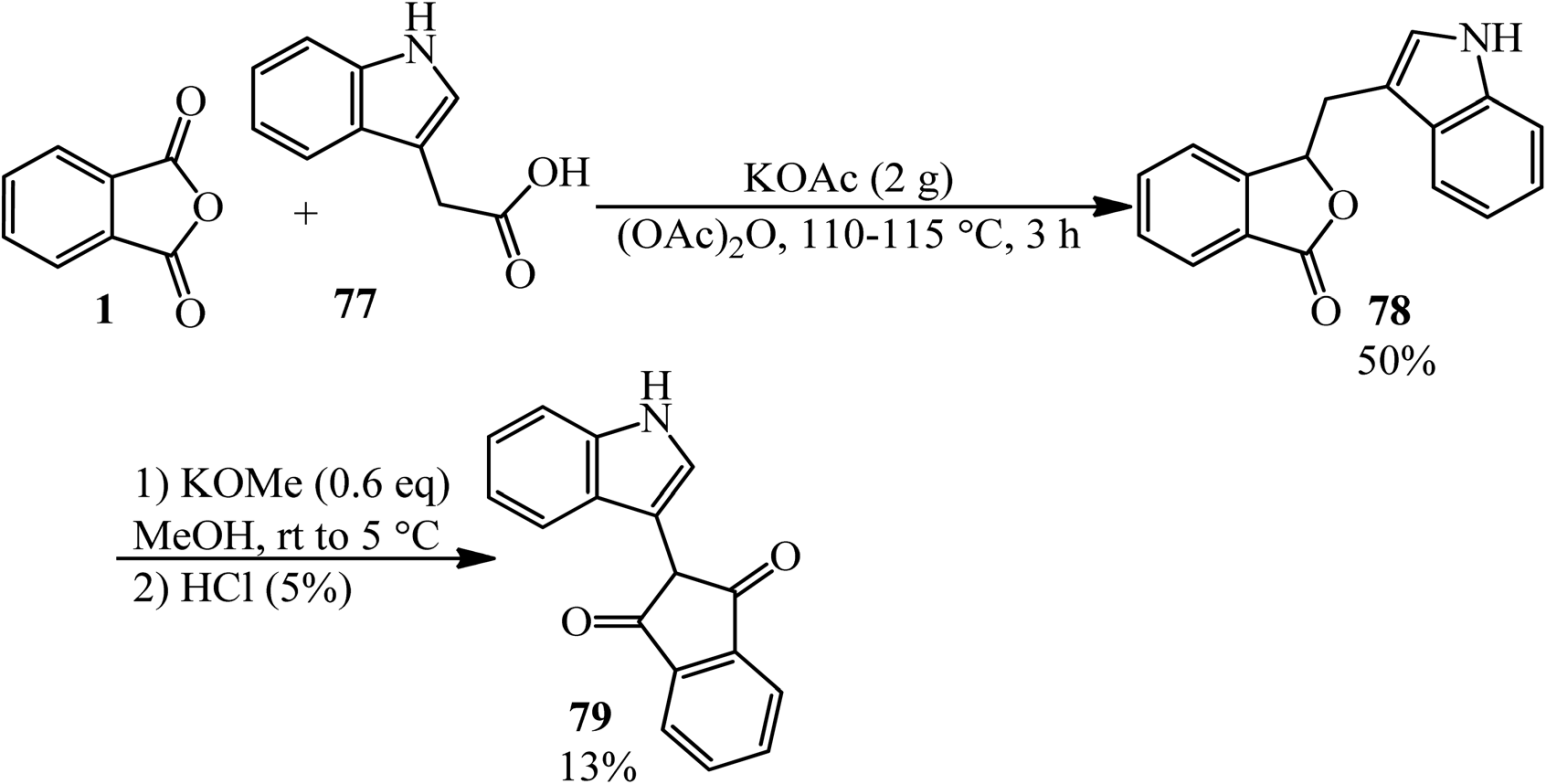
Perkin synthesis of 2-(3-indolyl)indane-1,3-dione.

Lácová in 1969 reacted indole-3-acetic acid (77) with PA, in a 0.56 : 1 molar ratio, under Perkin synthesis conditions (in the presence of potassium acetate and acetic anhydride) to form 3-(3-indolylmethine)phthalide (78), as yellow crystals, which underwent rearrangement in methanol in the presence of potassium methoxide to yield 2-(3-indolyl)indane-1,3-dione (79) (Scheme 29).^134^

**Scheme 30:**
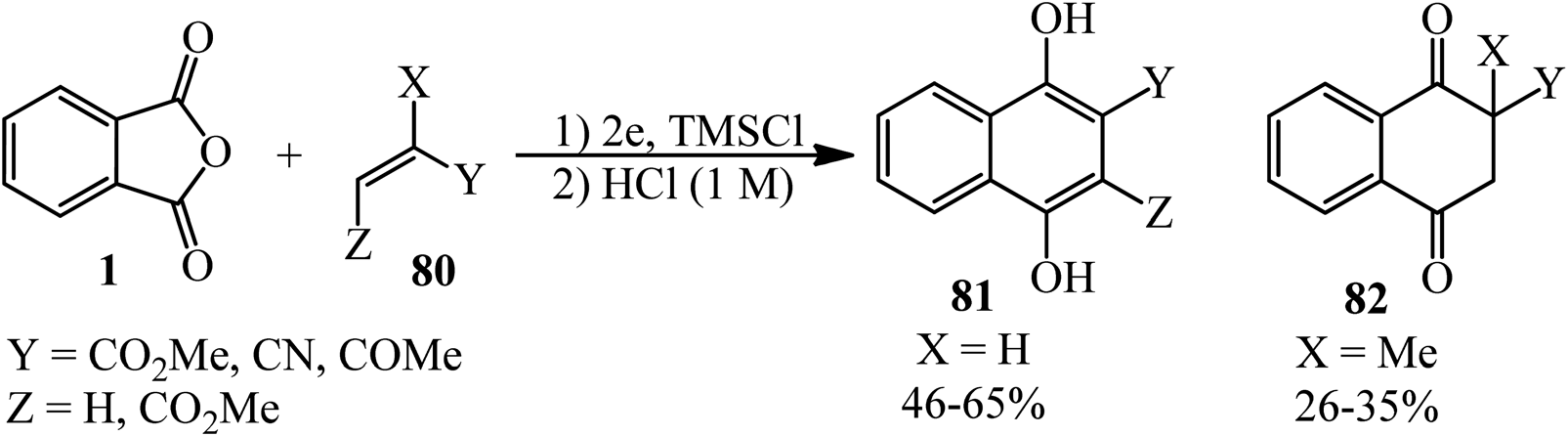
Synthesis of dihydroxynaphthalenes and 2,3-dihydronaphthalene-1,4-diones.

Kise and coworkers in 2020 demonstrated the electroreduction coupling of PA (1) with α,β-unsaturated carbonyl compounds (80) in a 1 : 3 molar ratio in the presence of TMSCl (5 mmol) and subsequent treatment with HCl (1 M), which gave 1,4-dihydroxynaphthalenes (81) and 2-methyl 2,3-dihydronaphthalene-1,4-diones (82) (Scheme 30).^135^

**Scheme 31:**
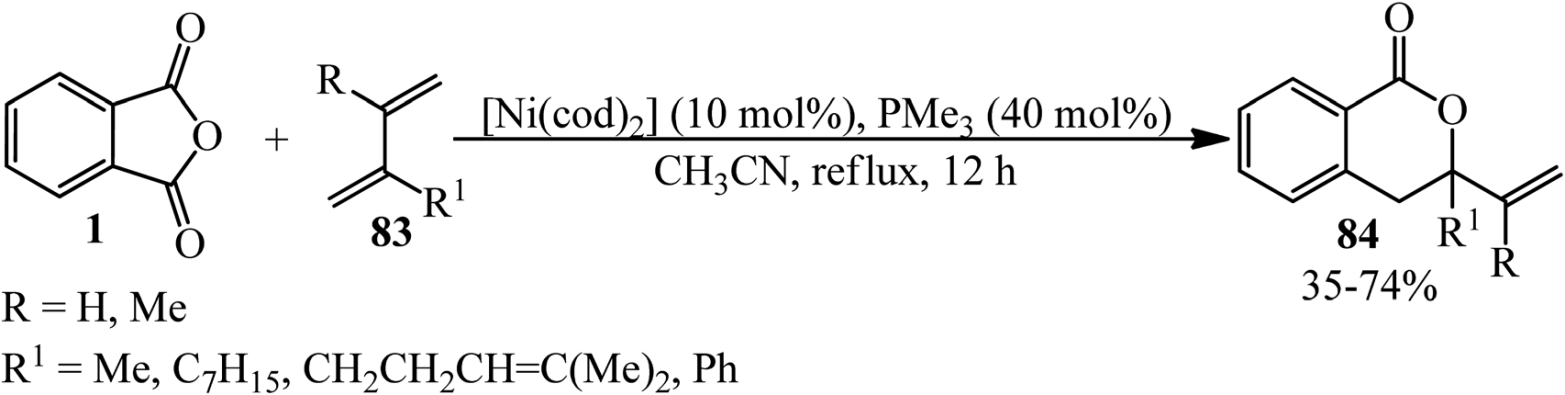
Substituted 3-vinyldihydroisocoumarins preparation.

An intermolecular nickel-catalyzed decarbonylative [4 + 2] cycloaddition between PA (1) with 1,3-dienes (83) in a 0.5 : 3 molar ratio in the presence of [Ni(cod)_2_] as a Ni(0) precursor to afford substituted 3-vinyldihydroisocoumarins (84) was studied by the Kurahashi group in 2011 (Scheme 31).^136^

**Scheme 32:**
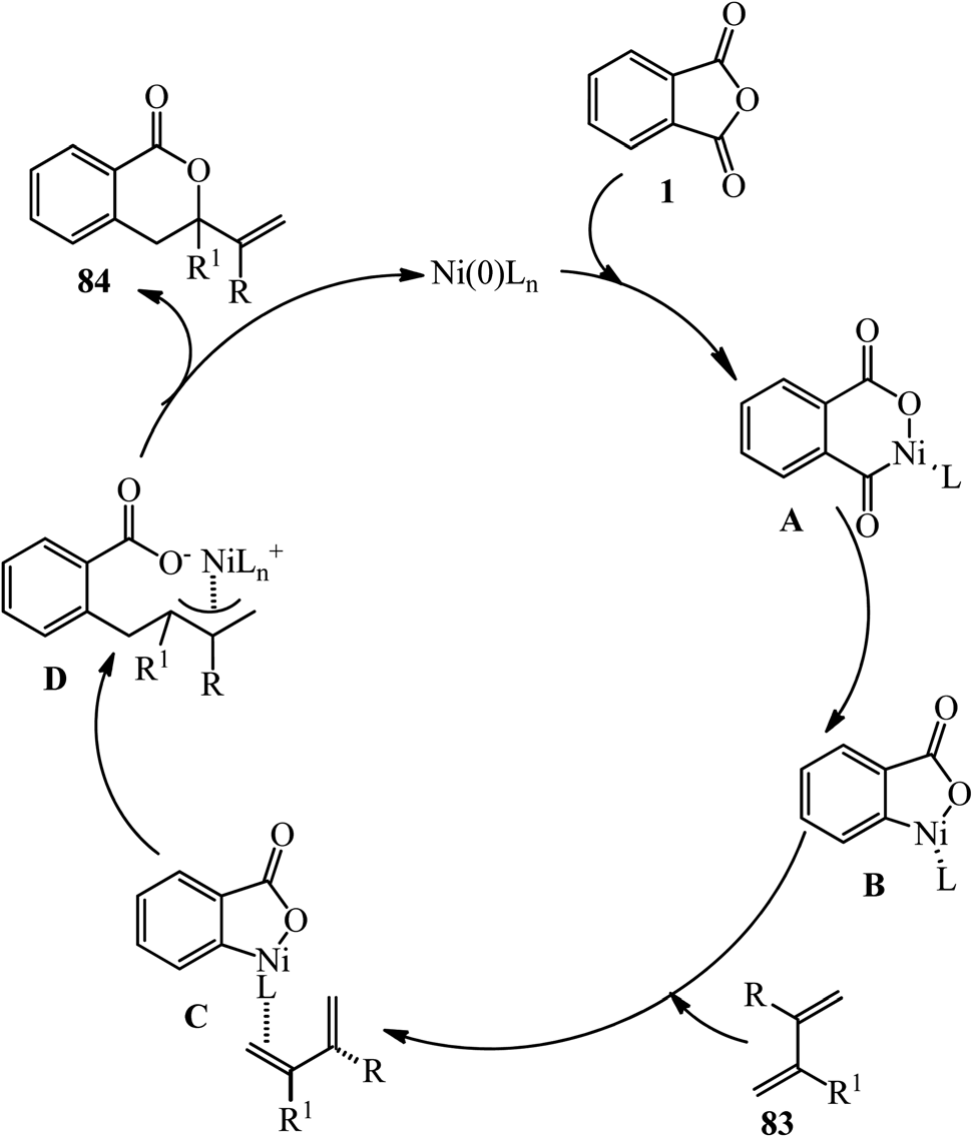
Plausible mechanism for the synthesis of substituted 3-vinyldihydroisocoumarins.

According to the proposed mechanism, the oxidative addition of (1) to Ni(0)-bearing electron-rich phosphine ligands gave the nickelacycle (A) (Scheme 32). 7,8 subsequent decarbonylation provides oxanickelacycle (B). The insertion of (83) through the more electron-rich C=C bond to C–Ni bond leads to the more stable acyclic^3^-allylnickel intermediate (D), which undergoes nucleophilic addition of oxygen to π-allylnickel at the more substituted carbon to give (84) and regenerates the starting Ni(0) complex.^136,137^

**Scheme 33:**
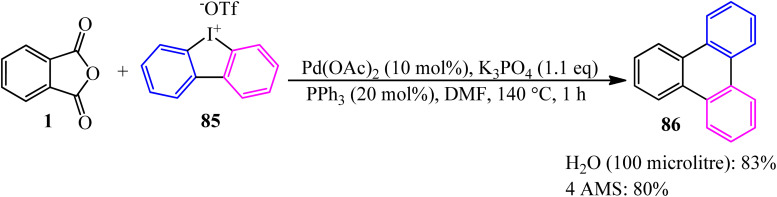
Synthesis of triphenylene.

In 2022, the palladium-catalyzed decarbonylative/decarboxylative [4 + 2] annulation of PA (1) with cyclic diaryliodonium salt (85) to synthesize triphenylene (86) developed by Li and coworkers (Scheme 33).^138^

**Scheme 34:**
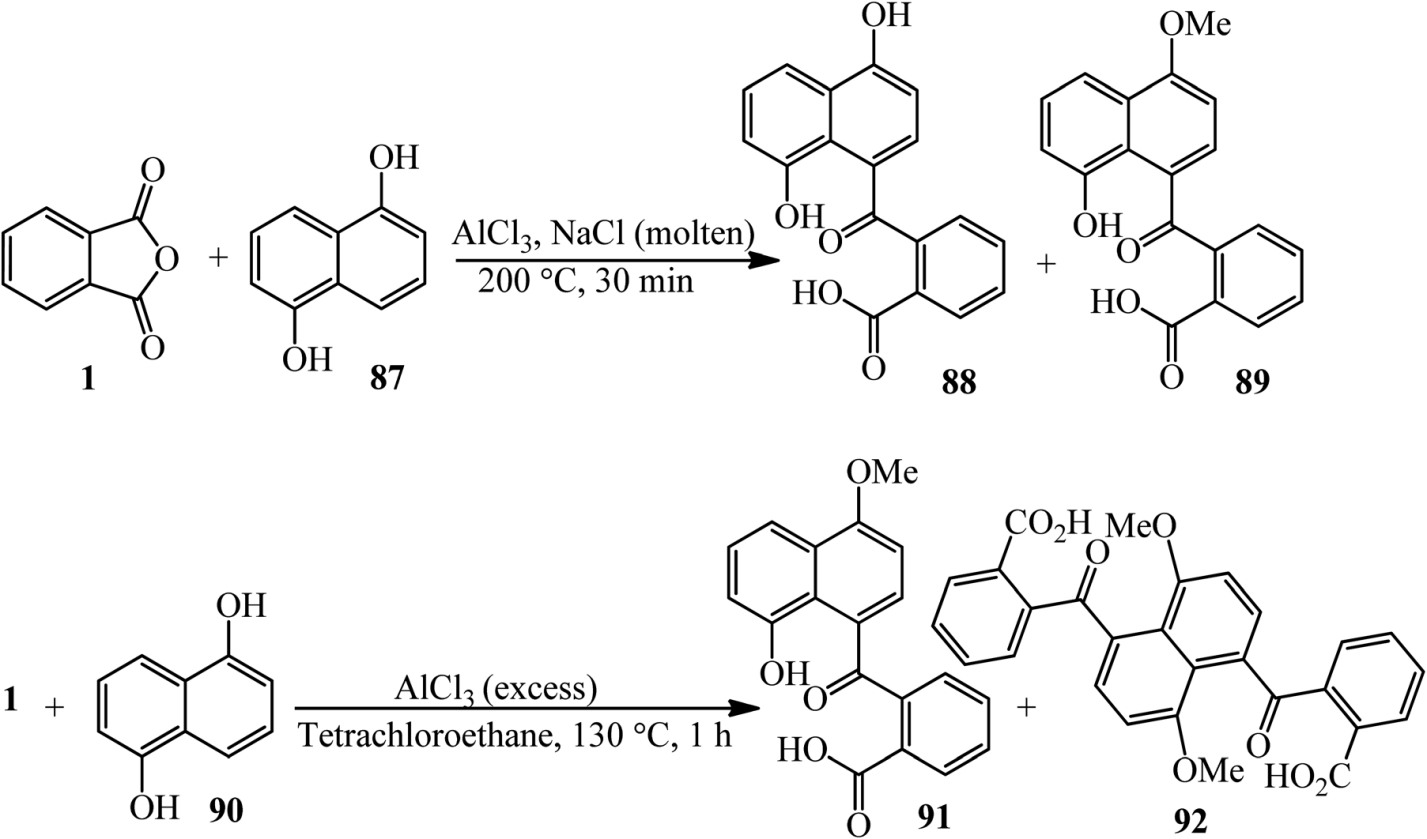
The Friedel–Crafts reaction of 1,5-dihydroxynaphthalene and 1,5-dimethoxynaphthalene with PA.

Satchel and Stacey in 1971 investigated some products [2-(4,8-dihydroxy-1-naphthoyl)benzoic acid and its methylated form] (88, 89) from a Friedel–Crafts reaction of PA (1) with 1,5-dihydroxynaphthalene (87). The reaction of 1,5-dimethoxynaphthalene (90) with PA and in the presence of excess aluminum chloride gave *o*-(8-hydroxy-4-methoxy-1-naphthoyl)benzoic acid (91) and *o*-(4,8-dihydroxy-1-naphthoyl) benzoic acid (92), while with an excess of phthalic anhydride, the main products are dimethoxy-acid and 4,8-bis-(2-carboxybenzoyl)-l,5-dimethoxynaphthalene (Scheme 34).^139^

**Scheme 35:**
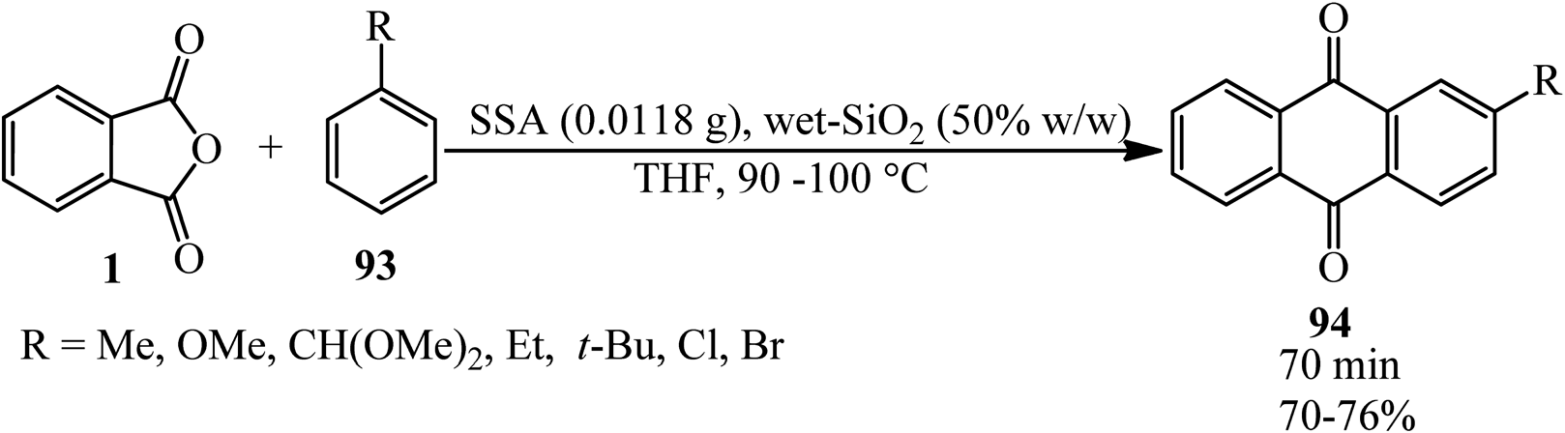
Synthesis of anthraquinones.

Naeimi and Shokrollah Brojerdi in 2014 gained anthraquinones (94) *via* the reaction of PA (1) and wide variety of substituted aromatic compounds such as (phenols, naphthols, alkyl benzenes, halo benzenes, and biphenyl) (93) using silica sulfuric acid (SSA) as a green, recyclable, and heterogeneous catalyst (Scheme 35).^140^ The catalyst could be reused seven times without significant activity loss.

Nie and Liu research group in 2022 provided 2-ethylanthraquinone (2-EAQ) (94) using PA (1) and ethylbenzene (93) as a feedstock by the combination of acylation and dehydration over a Sc-modified Hβ catalyst. Sc modification was used to create new strong Lewis acid and increased acid amount of Hβ.^141^

**Scheme 36:**
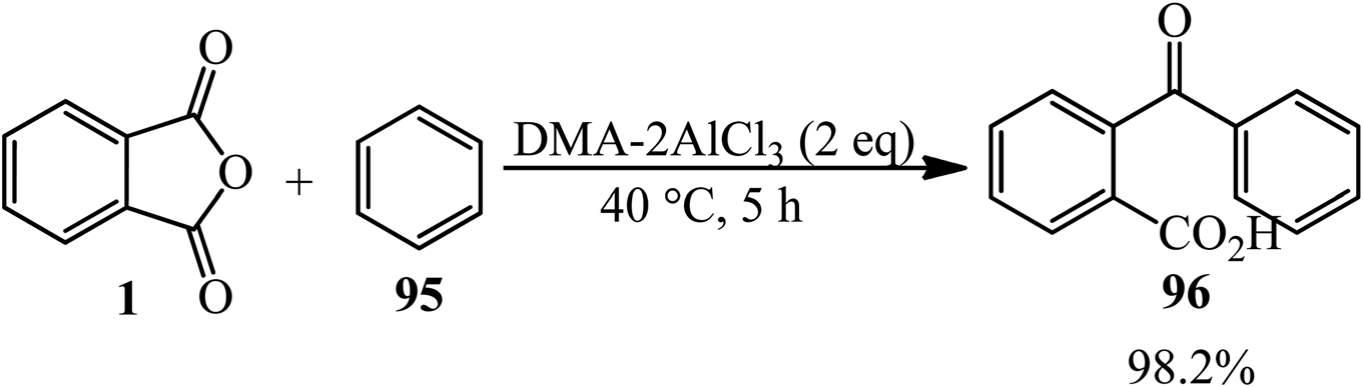
Synthesis of *o*-benzoylbenzoic acid (BBA).

The catalytic performance of amide-AlCl_3_ (DMA-2AlCl_3_) ionic liquid analogs in synthesizing *o*-benzoylbenzoic acid (BBA) (96) from PA and benzene (95) was demonstrated. The catalyst was obtained by mixing AlCl_3_ and DMA in 2 : 1 molar ratio at 100 °C for 3 h. Then, PA and benzene, in a 1 : 10 molar ratio were mixed at 40 °C within 5 h to obtain the product by 98.2% (Scheme 36).^142^ The authors proposed that Al_2_Cl_7_^−^, gained from DMA–2AlCl_3_, attacks the anhydride and formed (96) *via* an electrophilic substitution reaction.

**Scheme 37:**
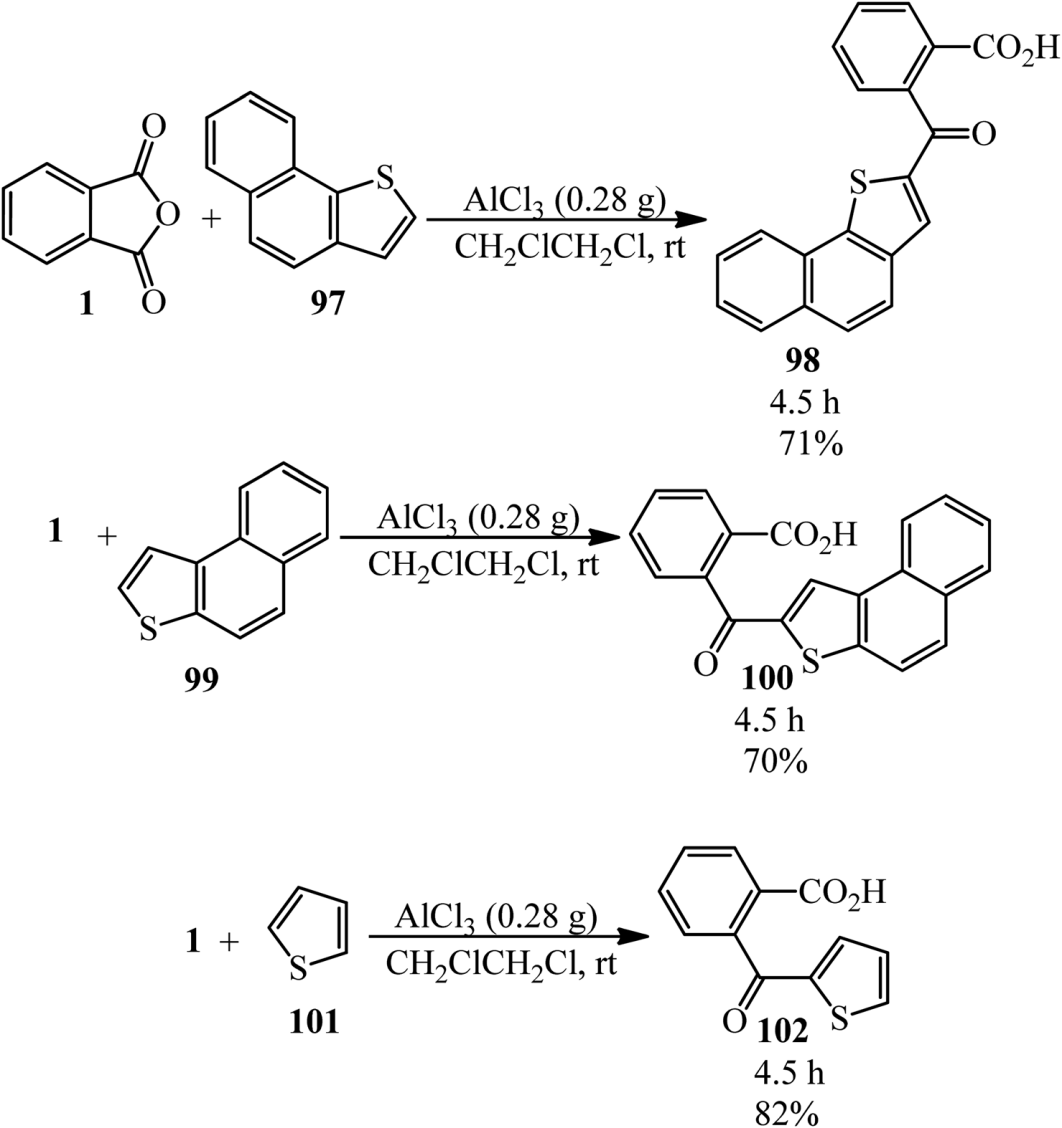
Reactions of naphto[1,2-*b*]thiophene, naphto[2,1-*b*]thiophene and thiophene with PA.

Naphto[1,2-*b*]thiophene (97) reacted with PA (1) to prepare ketocarboxilic acid (98). Naphto[2,1-*b*]thiophene (99) also reacted with PA to obtain 2-naphto[2,1-*b*]thienyl *o*-carboxyphenyl ketone (100) with 70% yield. The Friedel–Crafts reaction between PA and thiophene (101) yielded ketoacid (102) (Scheme 37).^143^

Naeimi and Namdari in 2009 described the direct preparation of anthraquinones (94) *via* the reaction of PA (1) and various benzene derivatives (93) in the presence of anhydrous AlCl_3_ (0.11 mmol)/methanesulfonic acid (0.01 mmol) (LAMA) at 95–100 °C within 15–65 min with 6–93% yield. Benzenes that contain electron withdrawing substituents (such as nitrobenzene and 1,3-dichlorobenzene) had the lowest reactivity in this reaction (8% and 6% yields, respectively). The compound 1,3-dinitrobenzene did not get the corresponding product even performing the reaction overnight.^144^

**Scheme 38:**
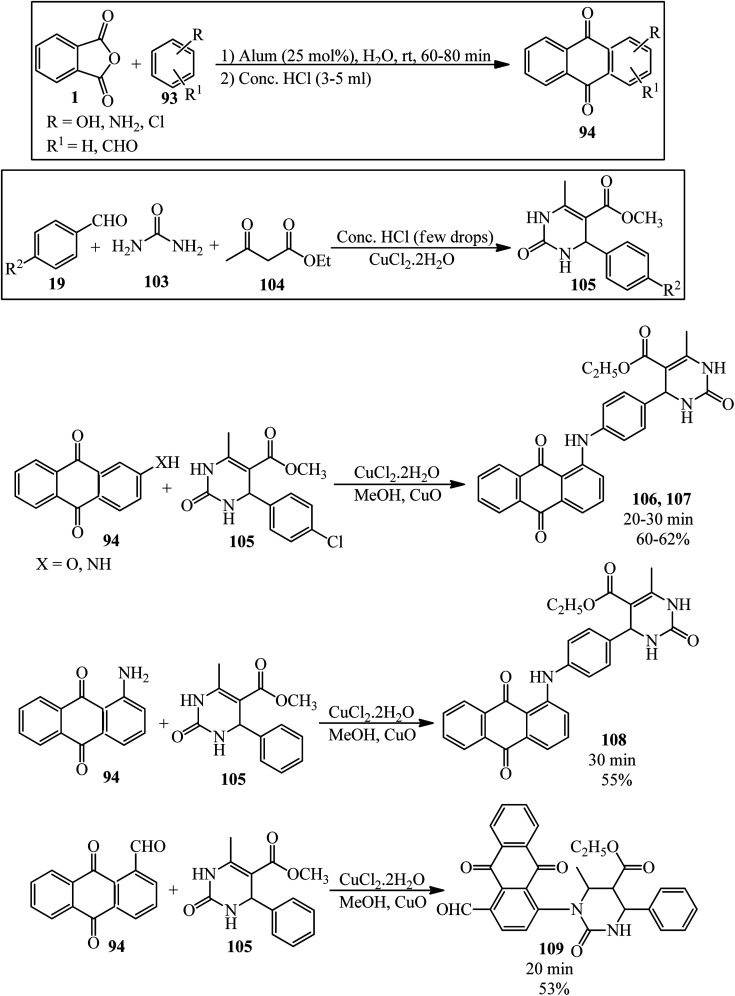
Synthesis of anthraquinone-based pyrimidines.

Shafiq group in 2021 reported newly synthesized anthraquinone-based pyrimidine derivatives. In the first step, the authors prepared the anthraquinone derivatives (94) *via* the modified procedure of Madje *et al.*^145^ in which the PA and benzene derivatives in a 1 : 1.1 molar ratio were mixed in aqueous media at room temperature in the presence of alum (KAl(SO_4_)_2_·12H_2_O), which was followed by the addition of concentrated HCl, washing with EtOAc, drying, and recrystallization from methanol. In the next step, the pyrimidine derivatives (105) (obtained by a procedure of Shafiq *et al.*^146^) were obtained *via* the three-component reaction of benzaldehydes (19), urea (103), and ethyl acetoacetate (104). In the third step, different derivatives of the precursors (94) and (105) were mixed in the presence of catalytic amount of copper chloride and cupric oxide in methanol for 20–30 minutes at ambient temperature to obtain the anthraquinone-based pyrimidines adducts (106–109) (Scheme 38).^147^ The antioxidant, antidiabetic, molecular docking, and QSAR studies of the products were also examined.

Phillips in 1927 reported the synthesis of alizarin through a multistep reaction. First, the condensation of PA, *o*-dichlorobenzene, and anhydrous aluminum chloride into 3′,4′-dichloro-2-benzoylbenzoic acid, and second, the conversion of this acid by means of sulfuric acid into 2,3-dichloro-anthraquinone. Upon fusion with alkali, the dichloro-anthraquinone (94) converted into alizarin.^148^ The authors confirmed the claim of Sprent and Dodd^149^ that 2-chloro-anthraquinone is obtained by the condensation of PA and o-dichlorobenzene could not be confirmed.

**Scheme 39:**
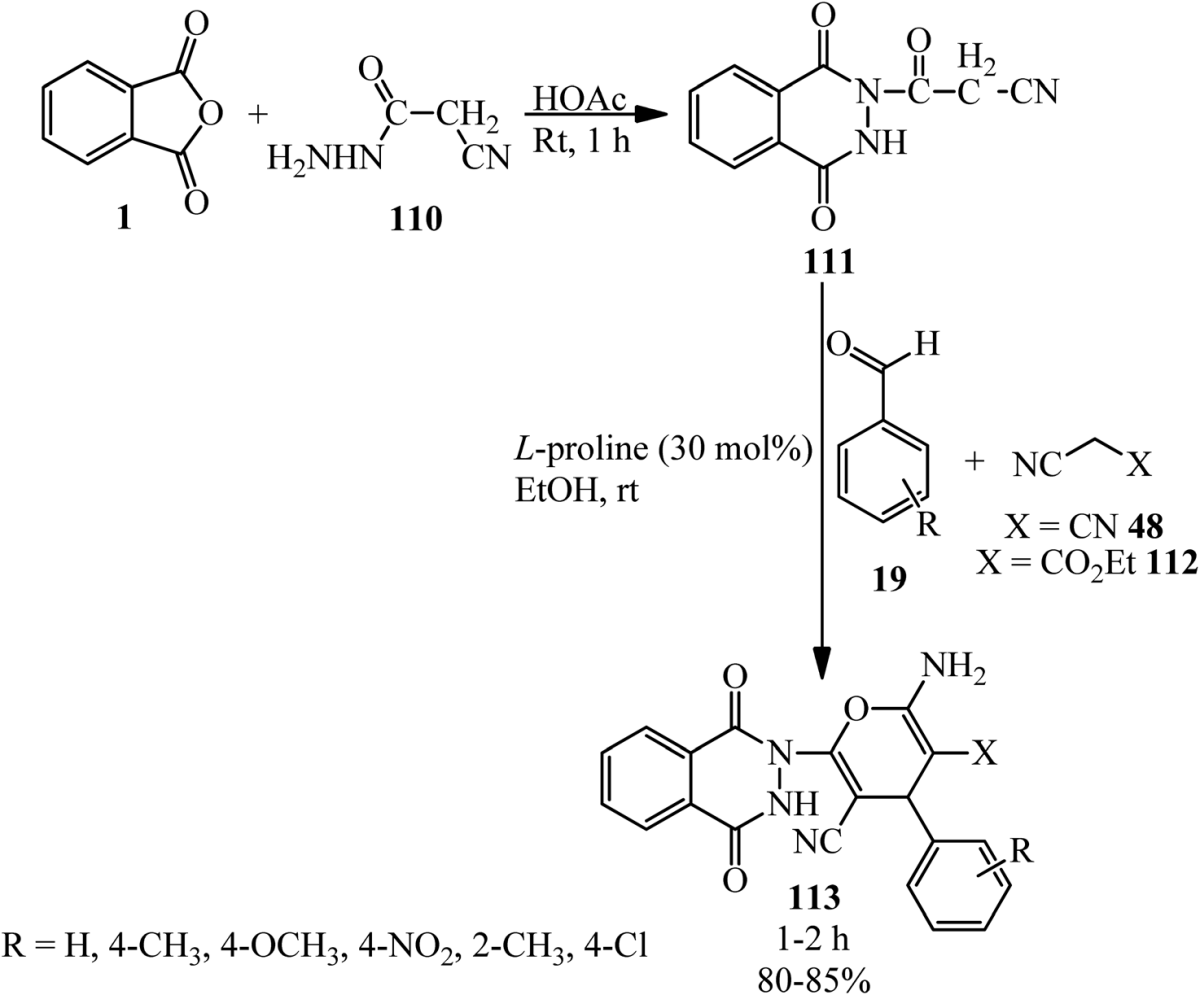
Synthesis of 2-amino-6-(1,4-dioxo-3,4-dihydrophthalazin)-2(1*H*)-yl-4-phenyl-4*H*-pyran-3,5-dicarbonitriles.

Kumar *et al.* in 2014 reported novel 2-amino-6-(1,4-dioxo-3,4-dihydrophthalazin)-2(1*H*)-yl-4-phenyl-4*H*-pyran-3,5-dicarbonitriles (113) through a two-step reaction. First, 3-(1,4-dioxo-3,4-dihydrophthalazin-(1*H*)-yl)-3-oxopropanenitrile (111) was obtained from rection of PA (1) with ethyl cyanohydrazide (110) in the presence of acetic acid. The adduct reacted with benzaldehydes (19) and active methylene compounds such as malononitrile (48) and ethyl cyanoacetate (112) using l-proline as the catalyst in EtOH at ambient temperature to afford (113) (Scheme 39).^150^

**Scheme 40:**
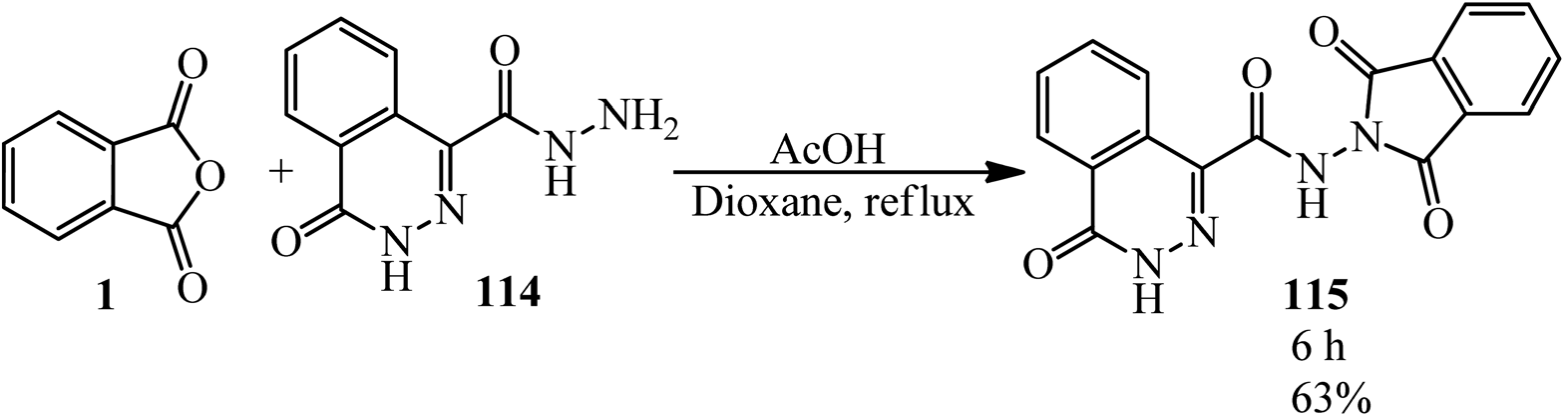
*N*-(1,3-Dioxoisoindolin-2-yl)-4-oxo-3,4-dihydrophthalazine-1-carboxamide preparation.

Abou-Elmagd group in 2012 achieved *N*-(1,3-dioxoisoindolin-2-yl)-4-oxo-3,4-dihydrophthalazine-1-carboxamide (115) *via* the reaction of PA and 4-oxo-3,4-dihydrophthalazine-1-carbohydrazide (114) in the presence of glacial acetic acid in refluxing dioxane (Scheme 40).^151^

**Scheme 41:**
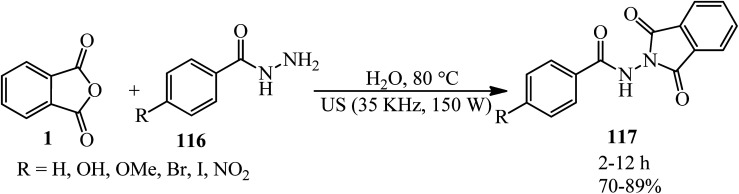
Synthesis of benzamide-dioxoisoindolines.

Simijonovic in 2020 described green aqua-mediated synthesis of benzamide–dioxoisoindoline derivatives (117) *via* the reaction of PA and benzoyl hydrazides (116) at 80 °C under ultrasonic irradiation. All compounds were subjected to experimental determination of their antioxidative potential. The DPPH test revealed that newly synthesized phenolic compounds are the best antioxidants (Scheme 41).^152^

**Scheme 42:**
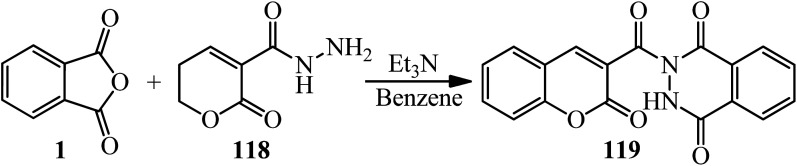
Synthesis of 2-(2-oxo-2*H*-chromene-3-carbonyl)-2,3-dihydrophthalazine-1,4-dione.

Aly's group in 2022 interpreted that the coumarin hydrazide (118) treated with PA (1) to furnish 2-(2-oxo-2*H*-chromene-3-carbonyl)-2,3-dihydrophthalazine-1,4-dione (119) by 76% yield (Scheme 42).^153^

**Scheme 43:**
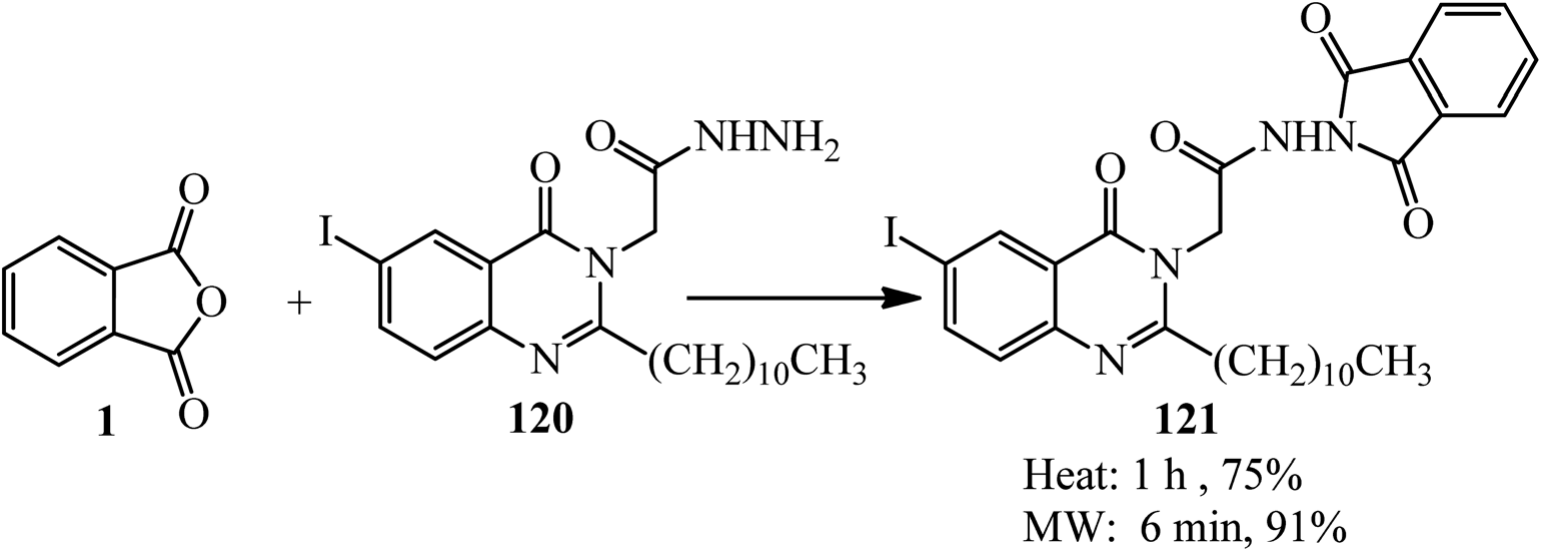
Production of 2-undecyl-4(3*H*)-quinazolinone.

In 2019, 2-(6-iodo-4-oxo-2-undecylquinazolin-3(4*H*)-yl)acetohydrazide (120) reacted with PA (1) to produce 2-undecyl-4(3*H*)-quinazolinone (121), which was obtained by either conventional method or by the microwave-assisted technique. The adduct was tested *in vitro* against a panel of three human tumor cell lines, namely, Hepatocellular Carcinoma (liver) *HepG2*, colon cancer *HCT-116*, and mammary gland breast *MCF-7*, which demonstrated satisfactory activity (Scheme 43).^154^

**Scheme 44:**
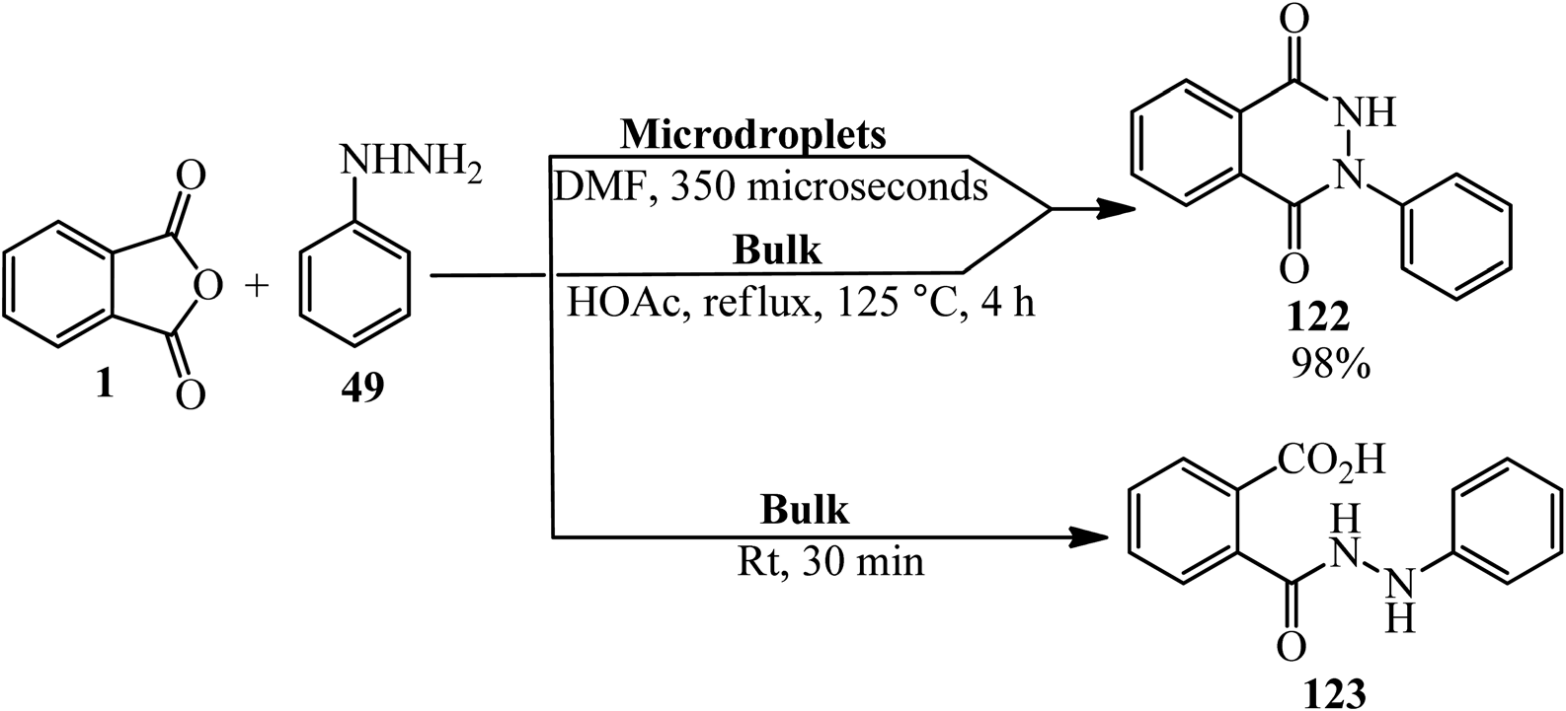
Investigating the reaction of PA and phenyl hydrazine.

Zare's group in 2019 performed the reaction PA (1) and phenyl hydrazine (49) in (A): microdroplets (the reaction condition: N_2_ atmosphere, DMF was demonstrated to be the best solvent for this microdroplet reaction, with a yield of product (122) up to 98% when the distance between the spray tip to the glass vial extended to 7 cm with reaction time of about 350 μs), and (B): bulk-phase without catalyst and without refluxing (the concentration of the both substrates is 10 mM). The microdroplet reaction led to significant formation of (122) with a small amount of product (123), whereas the bulk-phase reaction of PA and (49) in acetic acid catalyst under high-temperature refluxing led to product (122), but without catalyst at room temperature only (123) was formed. Moreover, this reaction in microdroplets showed excellent selectivity, yielding only the important six-membered heterocyclic product (122). This behavior indicated an extremely different reaction pathway in the confined microdroplets. The authors claimed that the reaction rate and selectivity enhancement was attributed to a surface reaction in the confined microdroplets having low pH values, which is enhanced by the positive charging of the microdroplets (Scheme 44).^155^

**Scheme 45:**
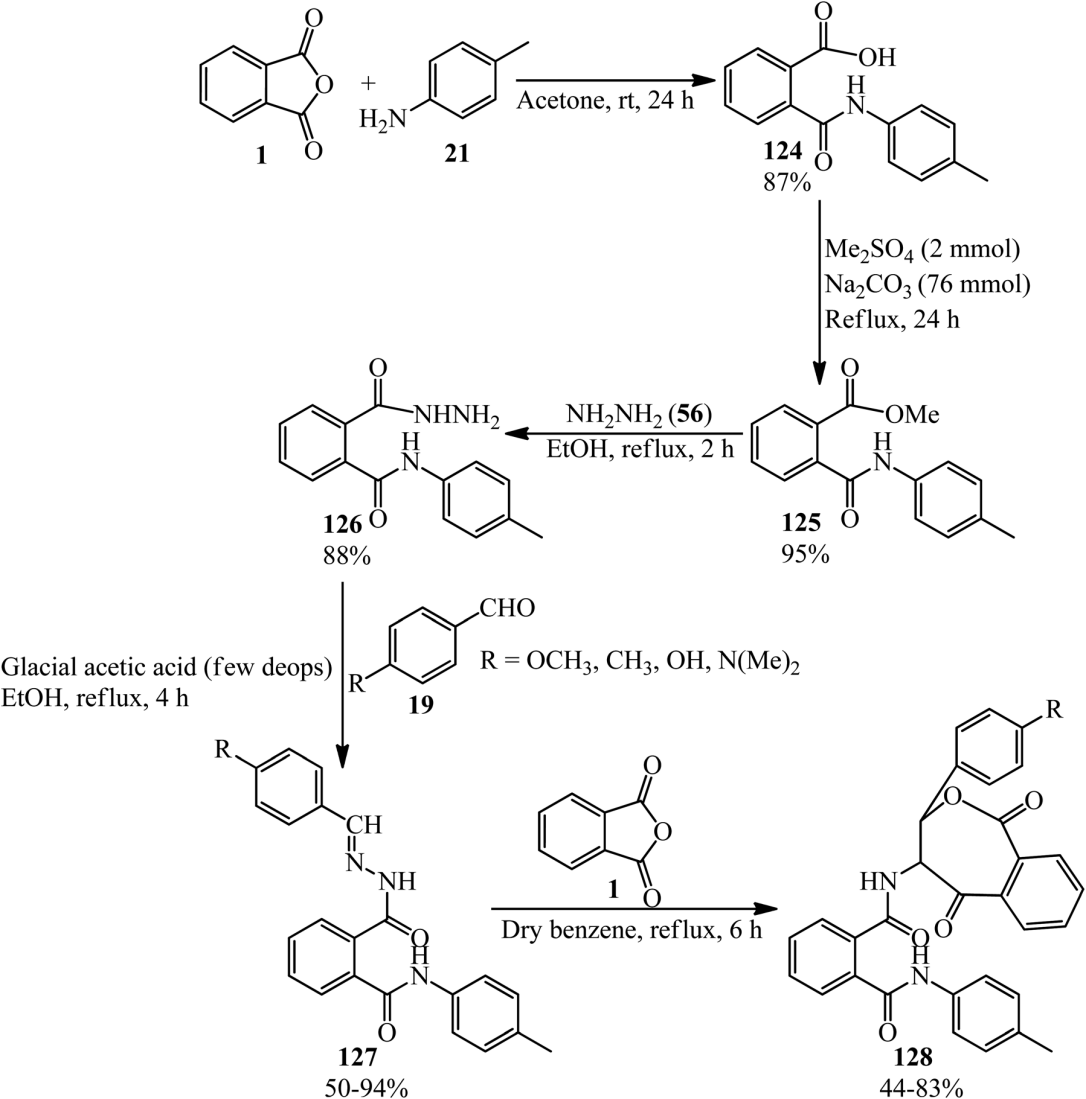
Preparation of Schiff bases and their 1,3-oxazepines derived from PA.

The Abbas group in 2021 achieved new Schiff bases and also their 1,3-oxazepines, derived from PA *via* a multistep reaction. In the first step, 2-[*N*-(4-methyl phenyl)]phthalamic acid (124) was prepared though the reaction of PA (1) and 4-methyl aniline (21) in a 1 : 1 molar ratio. In the next step, the methylation of (124) gave 2-[*N*-(4-methyl phenyl)]phthalamide acetate (125), which reacted with hydrazine (56) to get 2-[*N*-(4-methyl phenyl)]phthalamic acid hydrazide (126). The Schiff bases (127) was obtained in refluxing ethanol in the presence of glacial acetic acid as catalyst. Finally, the 1,3-oxazepine derivatives of the Schiff bases (128) gained from the consequent reaction of (127) with PA in refluxing benzene (Scheme 45).^156^

**Scheme 46:**
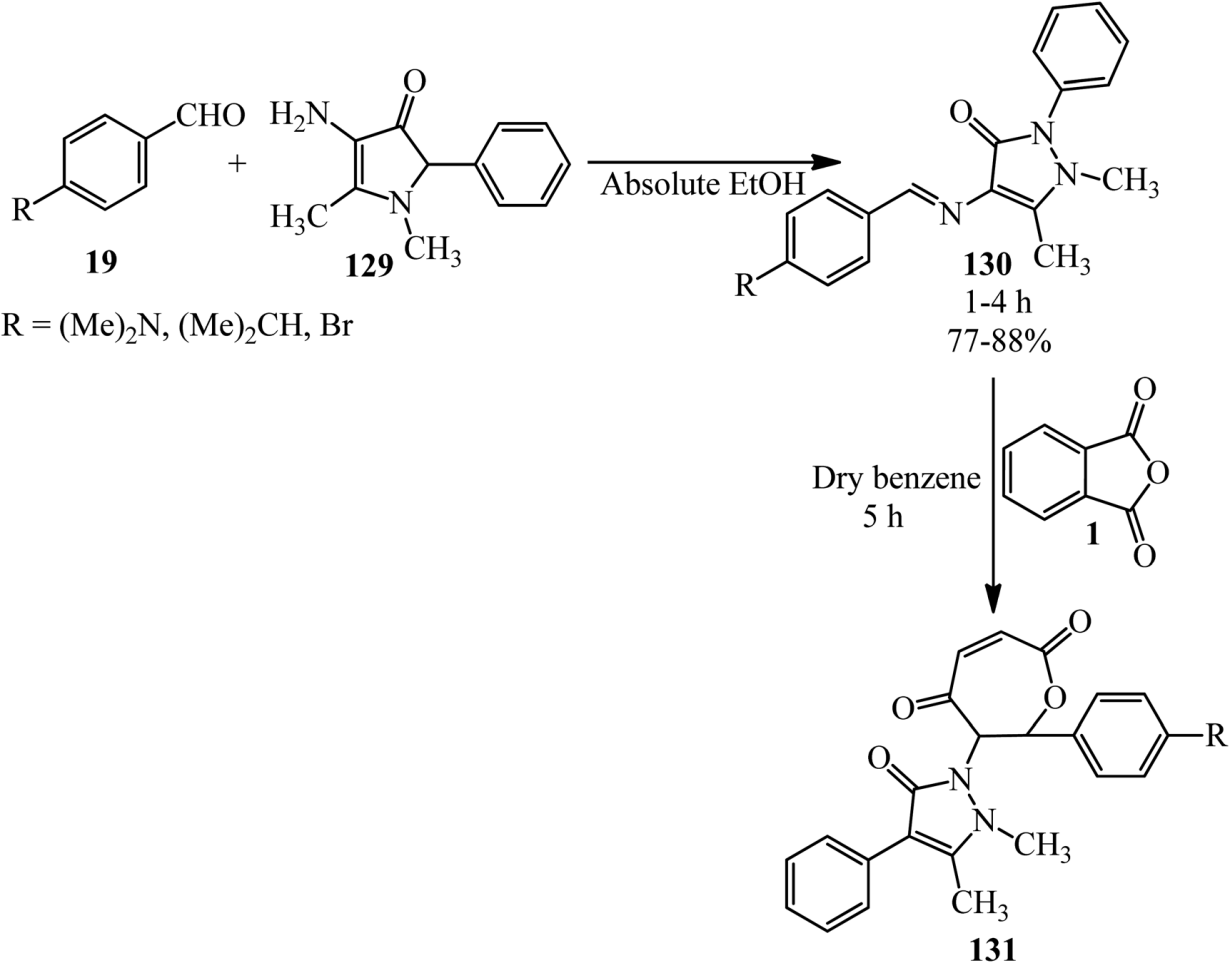
Synthesis of new 6-(2,3-dimethyl-5-oxo-4-phenyl-2,5-dihydro-1*H*-pyrazol-1-yl)-7-aryl-6,7-dihydrooxepine-2,5-diones.

Hanoon in 2011 reported new 6-(2,3-dimethyl-5-oxo-4-phenyl-2,5-dihydro-1*H*-pyrazol-1-yl)-7-aryl-6,7-dihydrooxepine-2,5-diones (131) *via* the reaction of PA (1) and Schiff bases (130) in dry benzene. The Schiff bases was obtained *via* the condensation of various aromatic aldehydes (19) with 4-aminophenazone (129) in the presence of glacial acetic acid as the catalyst (Scheme 46).^157^

**Scheme 47:**
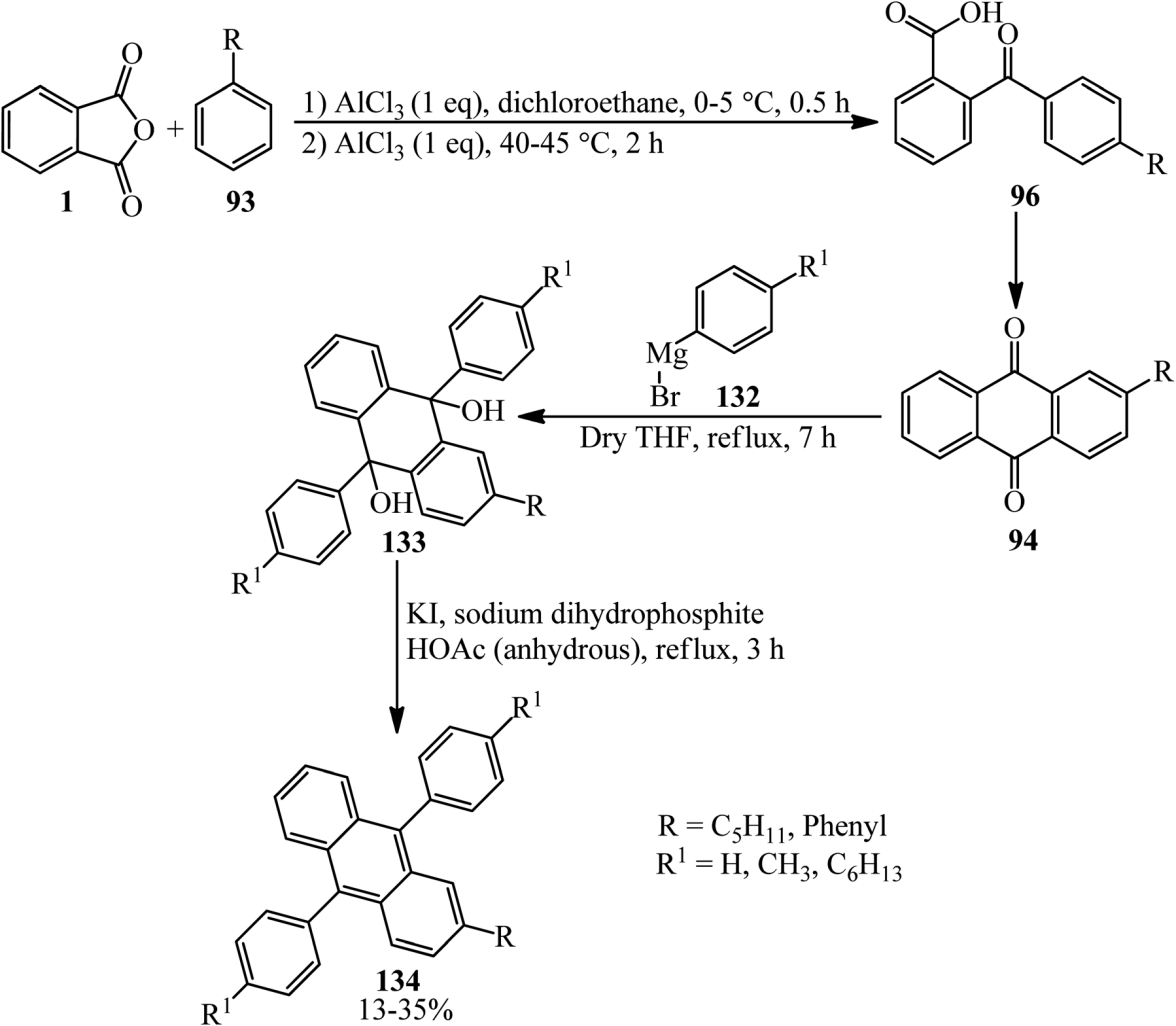
Synthesis of nonsymmetric 9,10-diphenylanthracenes (DPA).

In 2014, Serevičius group constructed a series of nonsymmetric 9,10-diphenylanthracenes (DPA) through a multistep reaction, which started from the acylation reaction of PA (1) and substituted benzenes (93) (including pentyl and phenyl benzenes) to obtain substituted benzoylbenzoic acid (96) that underwent an intramolecular cyclization in heated acidic media (such as poly phosphoric acid or oleum 5%), which led to the formation of 2-substituted anthraquinones (94), which reacted with arylmagnesium bromide (132), resulting in 9,10-diaryl-9,10-dihydroxydihydroanthracene (133), which was reduced to form 2,9,10-trisubstituted anthracenes (134) (Scheme 47).^158^ The authors investigations affirmed that DPA compounds are deep-blue emitters with enhanced charge transport properties.

**Scheme 48:**
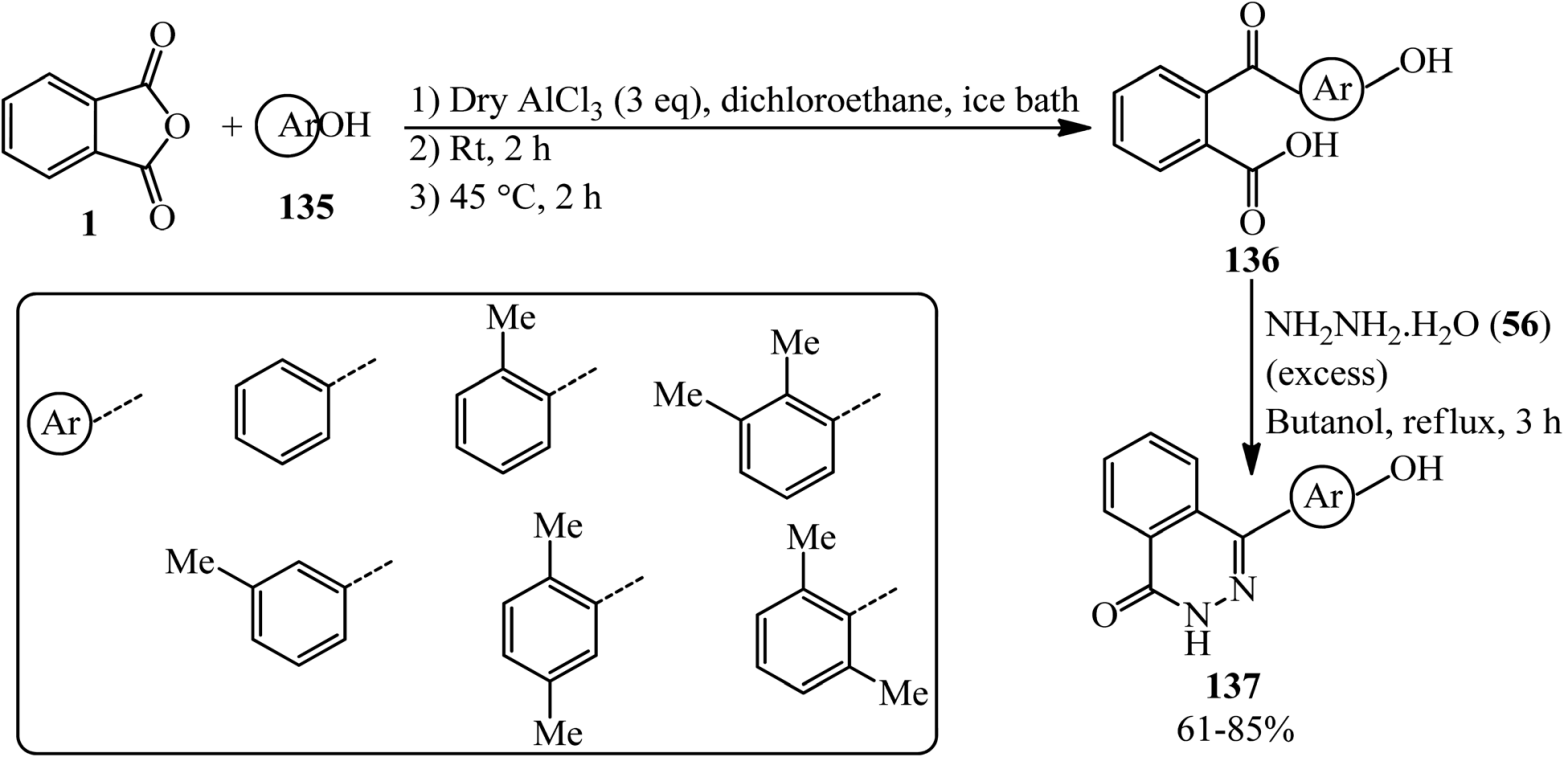
Synthesis of 4-(4′-hydroxyaryl)(2*H*)phthalazin-1-ones.

The synthesis of 4-(4′-hydroxyaryl)(2*H*)phthalazin-1-ones (137) was done by Cheng's group in 2007. The procedure consists of a two-step reaction, starting from the Friedel–Crafts acylation reaction of six phenols (135) with PA (1) to obtain α-keto acids (136), followed by cyclization with excess amount of hydrazine hydrate (56) in good to excellent yields with high regioselectivity to get products (137), which are methyl-substituted phthalazinone-containing bisphenol-like monomers; the adducts were utilized as monomers to obtain a number of novel heterocyclic poly(arylene ether ketone)s through the reaction with an activated difluoro monomer based on a novel N–C coupling reaction. The obtained polymers demonstrated polymers with high *T*_g_'s, good solubility, and excellent thermal stability (Scheme 48).^159^

**Scheme 49:**
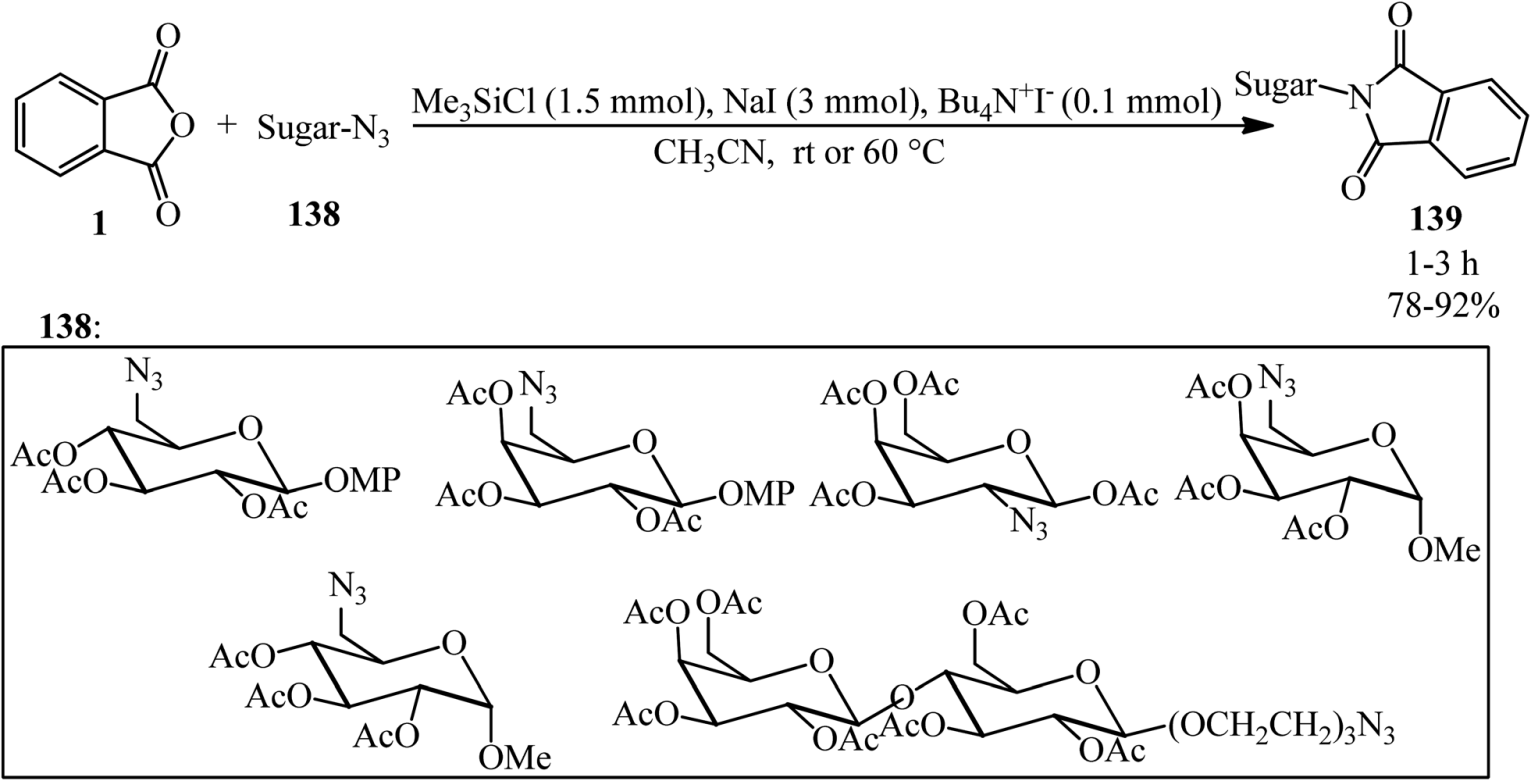
*N*-Sugar-substituted phthalimides preparation.

*N*-Sugar-substituted phthalimides (139) constructed in 2004 by Li's group *via* the reaction of PA (1) sugar azide (138) in a 15 : 1 molar ratio in the presence of tetrabutyl ammonium iodide (0.1 mmol, as catalyst) under essentially neutral conditions [which was performed by NaI (3 mmol) and Me_3_SiCl (1.5 mmol)] in dry acetonitrile at ambient temperature or 60 °C within 1–3 h with 78–92% yield (Scheme 49).^160^

Kamal in 1994 also reported *N*-substituted phthalimides (139) from the reaction of the corresponding azides (138) and PA (1) in a 1 : 1 molar ratio, employing chlorotrimethylsilane and sodium iodide (*in situ* generation of iodotrimethylsilane) in acetonitrile at ambient temperature within 15 min with 95% yield.^161^

**Scheme 50:**
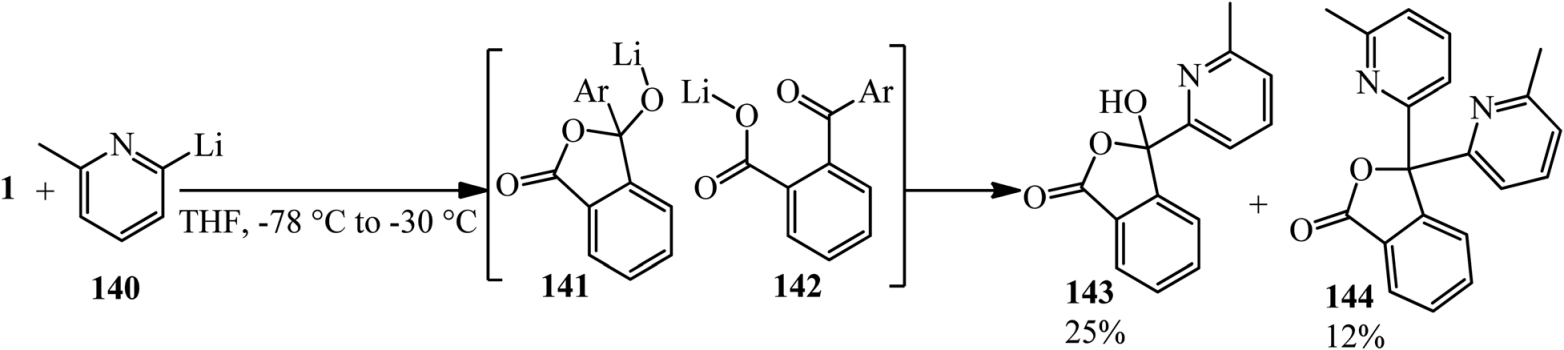
Addition of pyridinyl lithium to PA.

Nguyen and coworkers in 2012 performed the bisaddition of pyridinyl lithium (140) to PA, which yielded the monoaddition product 3-hydroxyisobenzofuranone (143) and 3,3-bis(6-methylpyridin-2-yl)isobenzofuran-1(3*H*)-one (144) as the byproduct. According to the mechanism, presumably, bisaddition arises from the ring opening of the initially formed alkoxy-isobenzofuranone (141), leading to diaryl ketone (142), which reacted with the organometallic reagent to give (144) (Scheme 50).^162^

**Scheme 51:**
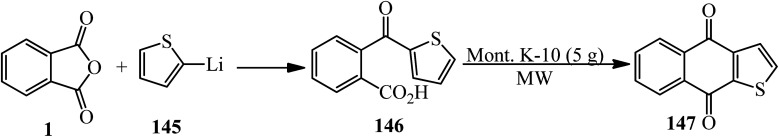
Synthesis of thieno[2,3-*b*]-l,4-naphthoquinone.

Acosta in 1995 synthesized 2-thenoylbenzoic acid (146) *via* the reaction of the PA (1) and 2-thienyllithium (145) by 80% yield. The product (146) cyclized in the presence of purified Mont K-10 (under microwave irradiation) to obtain thieno[2,3-*b*]-l,4-naphthoquinone (147) (Scheme 51).^163^

**Scheme 52:**
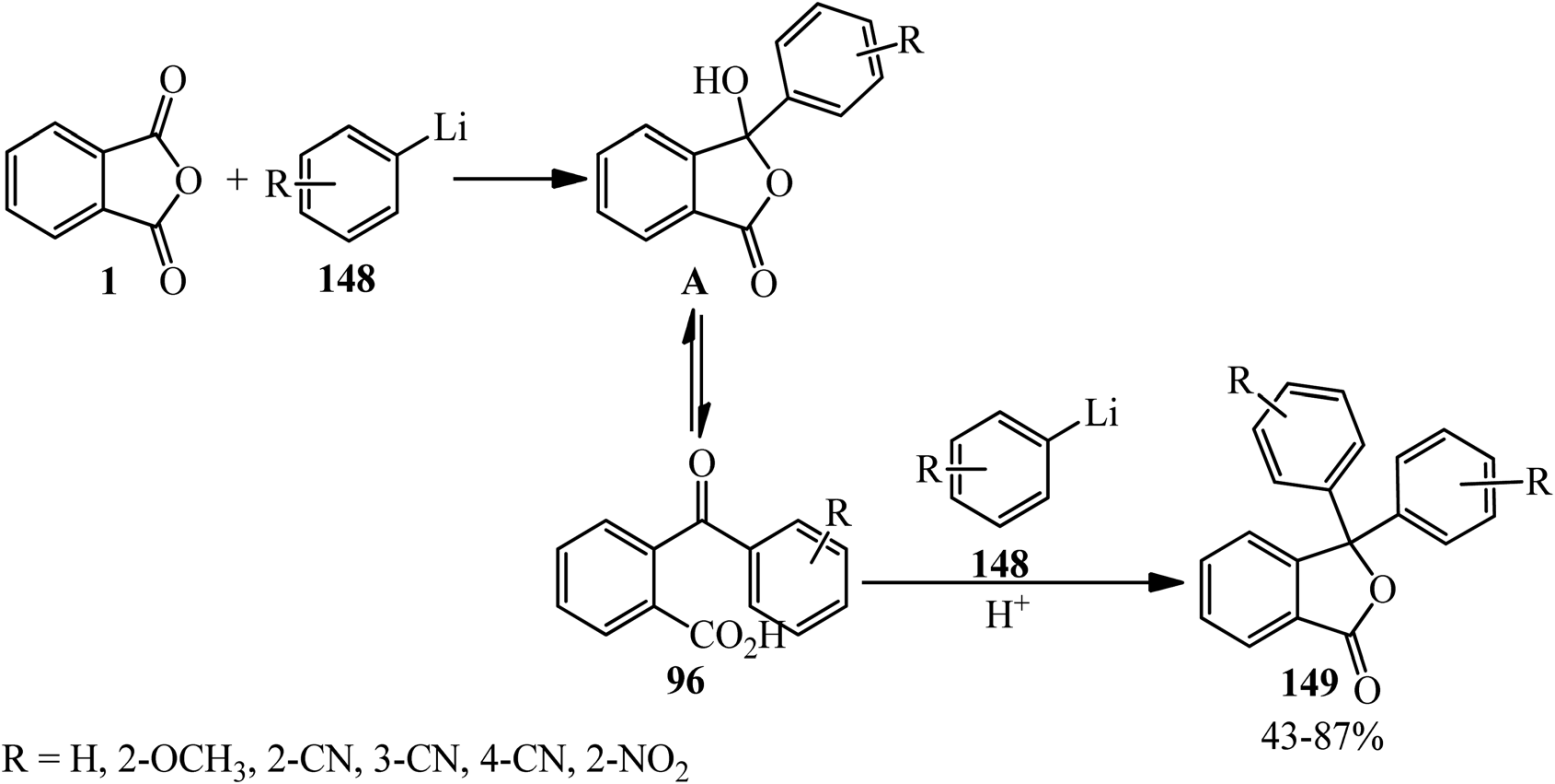
Investigation the reaction of PA and phenyllithium.

Parham and Piccirilli in 1976 claimed that when equimolar amounts of PA (1) and phenyllithium (148) were added at −78 °C, the yield of phthalide (149) was 78% based on phenyllithium. When the same ratios were maintained but the order of addition reversed, the yield of isolated (149) was 9%, while the yield of *o*-benzoylbenzoic acid (96) was 35%. Furthermore, the yield of *o*-benzoylbenzoic acid was further increased (55%) when excess (2 eq.) of PA was employed. In subsequent experiments, the aryllithium reagent was added rapidly to PA (2 eq.) in tetrahydrofuran at −100 °C, in which good yields of substituted benzoylbenzoic acids (96) were obtained (Scheme 52).^164^

**Scheme 53:**
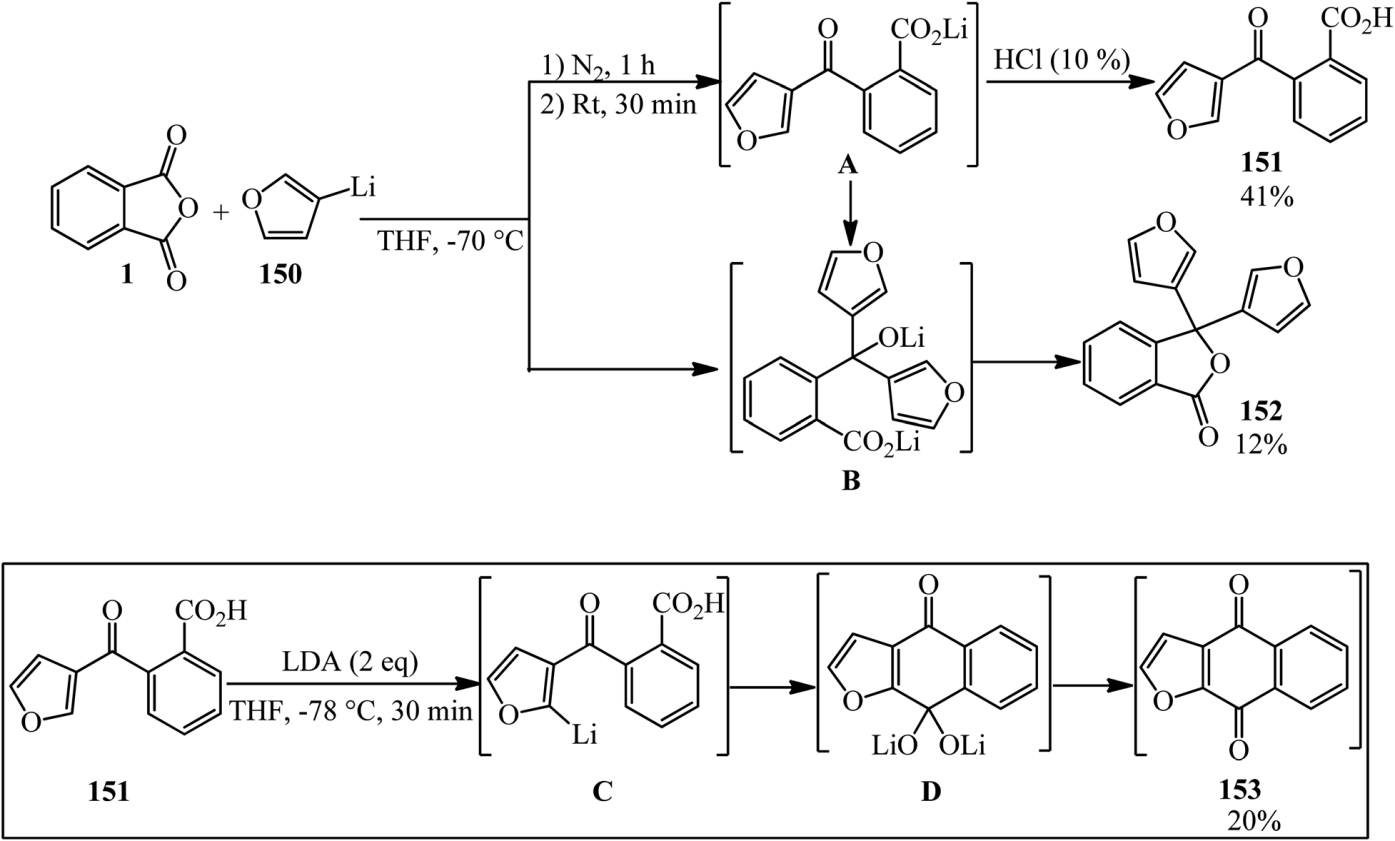
Identification of the PA and furan-3-yllithium reaction.

Tanaka's group in 1994 identified the reaction of PA (1) and furan-3-yllithium (150) to obtain 2-(3-furanoyl)benzoic acid (151) though the inverse addition method. On the other hand, the lactonization of (B) gave 3,3-di-(3-furyl)-1,3-dihydroisobenzofuran-1-one (152). The reaction of (151) with LDA gave naphtho[2,3-*b*]furan-4,9-dione (153) as yellow needles (Scheme 53).^165^ The authors attempted to obtain (153) *via* the chlorination of (151) with thionyl chloride or PCl_5_, which was not successful.

**Scheme 54:**
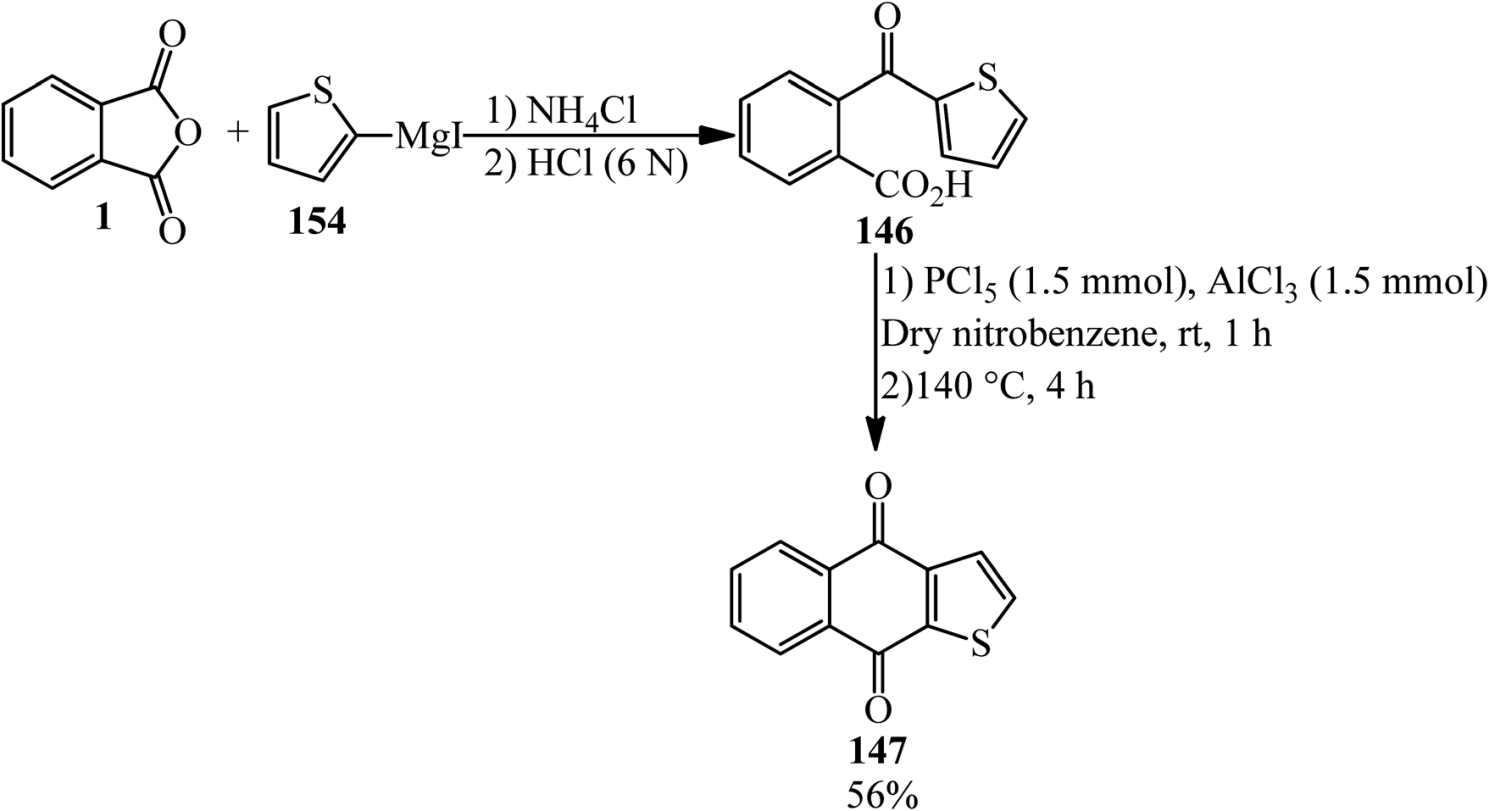
Reaction of PA and 2-thenoylmagnesium iodide.

Martin and Seoane in 1992 claimed that 2-thenoylbenzoic acid (146), obtained from PA (1) and 2-thenoylmagnesium iodide (154), yielded thieno[2,3-*b*]-l,4-naphthoquinone (147) (Scheme 54).^166^

**Scheme 55:**
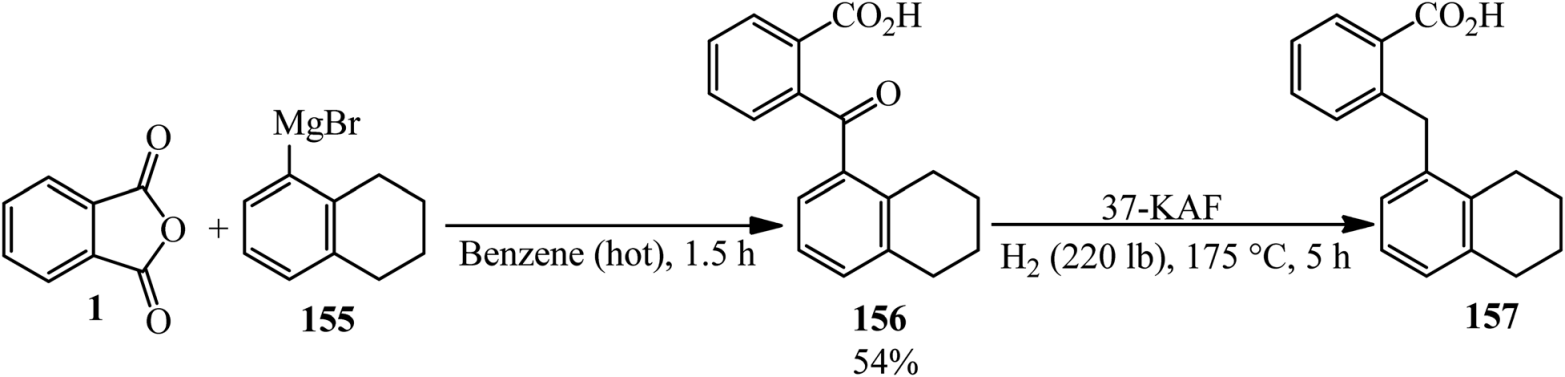
Reaction of PA and α-tetralylmagnesium bromide.

Fieser and Hershbe in 1937 reported the reaction of PA and α-tetralylmagnesium bromide (155) yielded the 2-(α-tetraloy1)-benzoic acid (156) as colorless prisms, which was reduced to 2-((5,6,7,8-tetrahydronaphthalen-1-yl)methyl)benzoic acid (157) by high pressure hydrogenation (Scheme 55).^167^

**Scheme 56:**
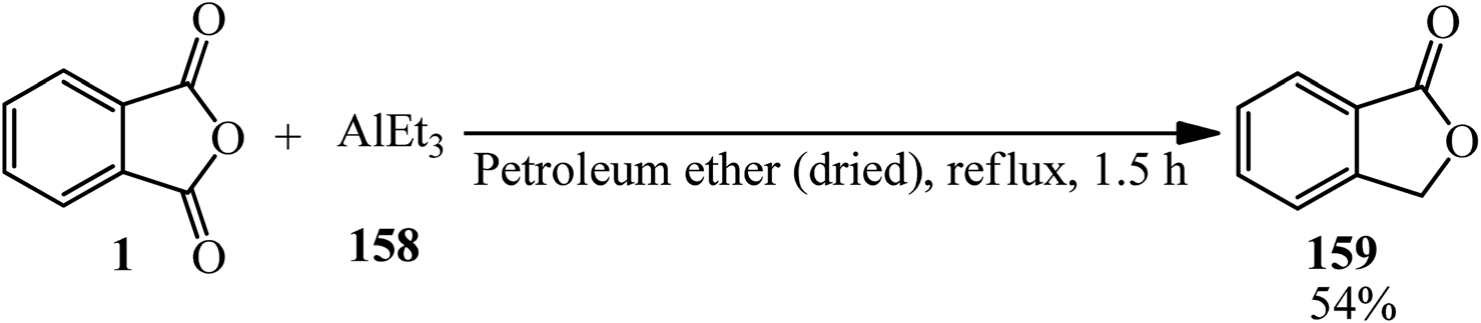
Reaction of PA with triethylaluminum.

Baba in 1968 mixed PA (1) with triethylaluminum (158), which surprisingly got the reduced isobenzofuran-1(3*H*)-one (159) instead of the common ketoacid corresponding to the Grignard reaction (Scheme 56).^168^

**Scheme 57:**
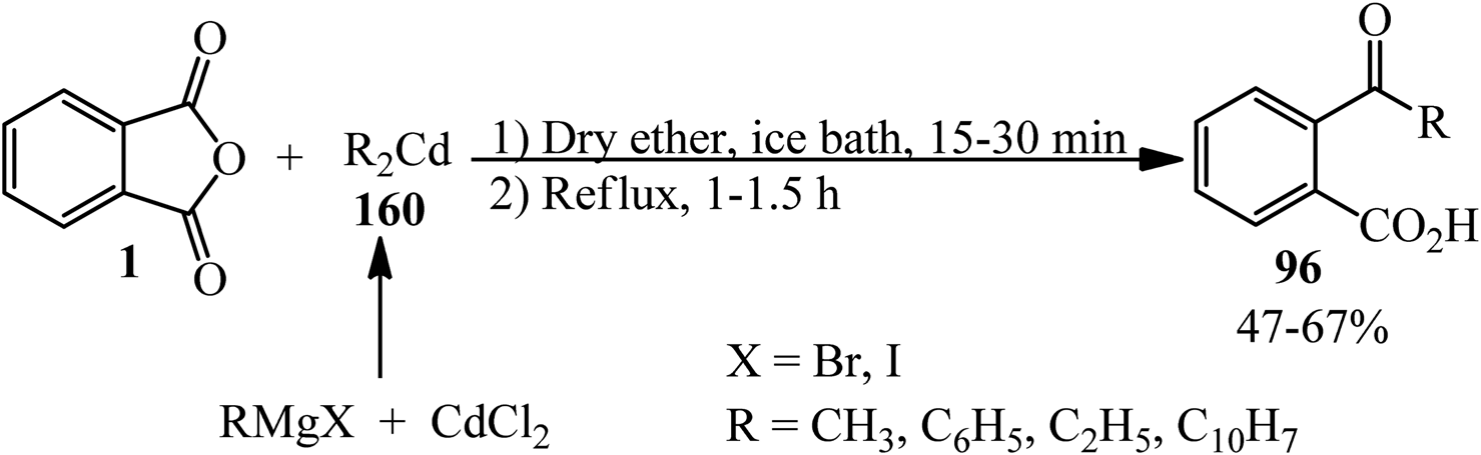
Reaction of PA and organocadmium compounds.

Benneville in 1941 demonstrated the reaction of PA (1) and organocadmium compounds (160) (including dialkyl- and diaryl-cadmium) to obtain keto acids (96). The method consists of more satisfactory yields than the ones achieved from the reaction of PA with the Grignard reagent and is more generally applicable than the Friedel–Crafts synthesis. At first, the organocadmium was obtained from mixing the Grignard reagent and anhydrous cadmium chloride by the method of Gilman and Nelson.^169^ Then, a solution of PA in dry ether was added to organocadmium within fifteen to thirty minutes in an ice-bath, followed by refluxing (Scheme 57).^170^

In addition, McMullen in 1922 studied the Friedel–Crafts reaction of PA with benzene in the presence of AlCl_3_ in detail.^171^

**Scheme 58:**
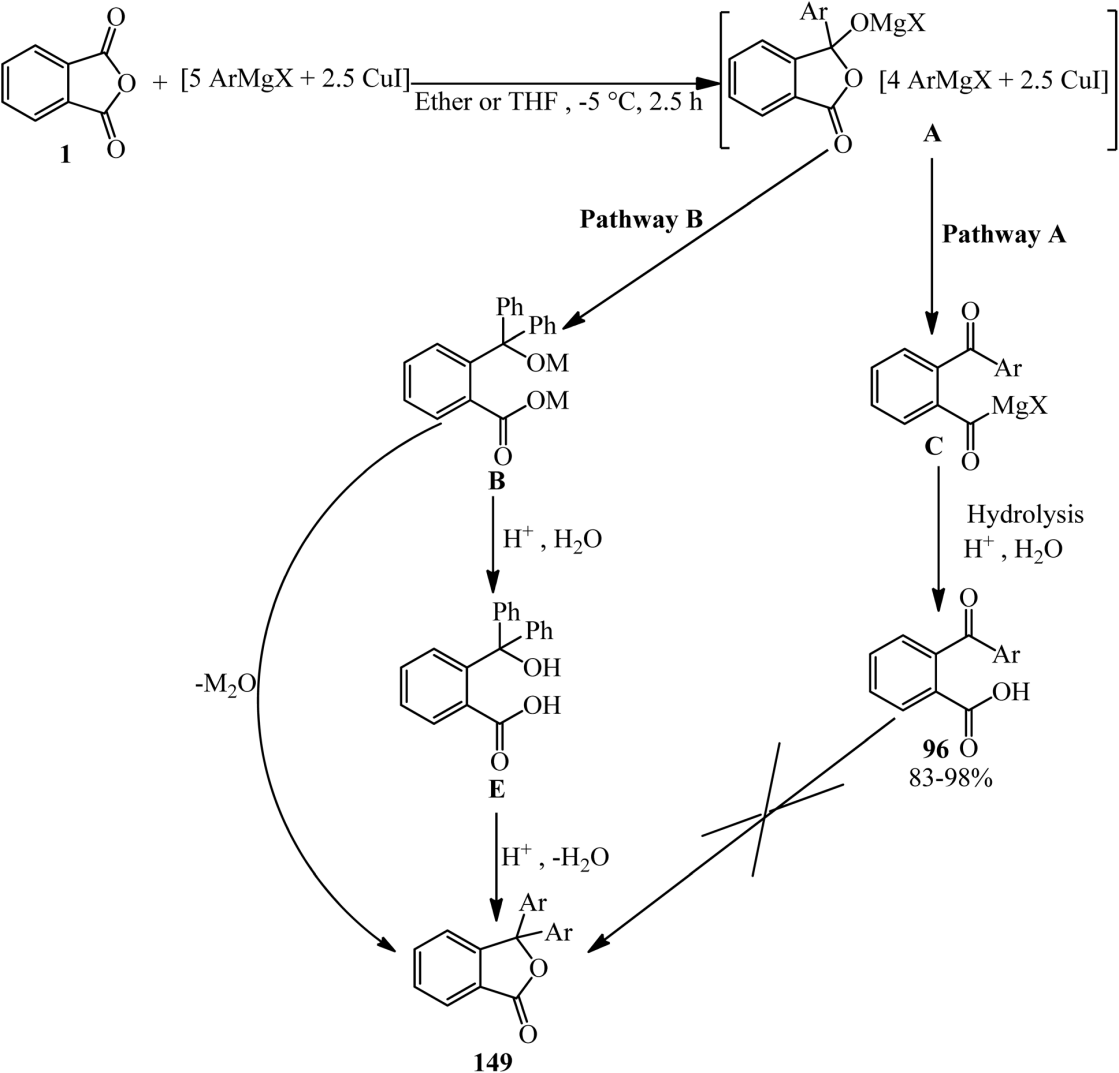
Investigating the reaction of arylcoppermagnesium reagents with PA.

Rahman and Nahar in 1992 studied the reaction of arylcoppermagnesium reagents (prepared from ArMgX and CuI) with one equivalent of PA. The phenylcoppermagnesium reagent (Ar = C_6_H_5_) gave 2-benzoylbenzoic acid (96) and 3,3-diphenylphthalide (149) in 40% and 42% yield, respectively. The yield of 2-benzoylbenzoic acid (96) increased to 93% in the presence of dimethyl sulphide. Under these conditions, no phthalide was formed. On the other hand, lithium diphenylcuprate reacted with PA in ether–hexane to give 2-benzoylbenzoic acid (96) and 3,3-diphenylphthalide (149) in 92% and 7% yields, respectively. The reaction of phenylcopper reagent (prepared from PhMgBr and CuI) and PA under similar conditions proceed slowly to get 2-benzoylbenzoic acid (96) in 15% yield. The reaction of phenylmagnesium bromide in the presence or absence of catalytic amounts of copper(i) iodide led to unsatisfactory results. The use of two equivalents of phenyllithium with one equivalent of PA, on the other hand, afforded 3,3-diphenylphthalide (149) in 77% yield. The authors believed that the species (A) is formed as the first step. If it is stable, it would be present in the system long enough to undergo an intramolecular ring opening (pathway A) to form 2-aroylbenzoate (C), which hydrolyzed to generate 2-aroylbenzoic acid (96). On the other hand, if (A) is very unstable, it may decompose intramolecularly by the transfer of an organic group and concomitant ring opening (pathway B) to produce (B), which converted into the lactone form, which is 3,3-diphenylphthalide (149) either during hydrolysis or by the elimination of metal oxide prior to hydrolysis. Compared with diethyl ether, THF and dimethyl sulphide are expected to have greater stabilizing effects on species (A), presumably by forming more stable complexes; thus, in their presence, the reaction of (A) directed to pathway A. Since dimethyl sulphide is the most efficient of the ligands examined, it is not surprising that the yields of 2-benzoylbenzoic acid are the highest in its presence (Scheme 58).^172^

**Scheme 59:**
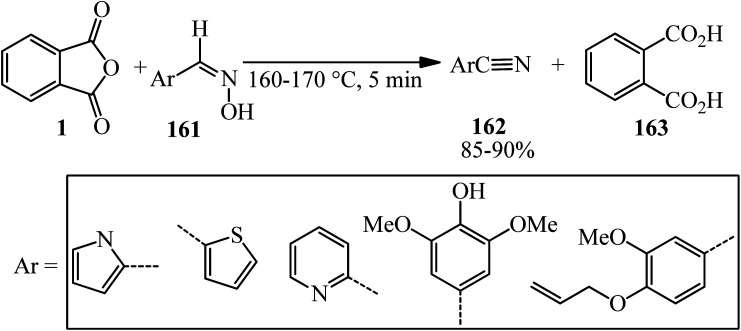
Reaction of PA and aldoximes.

Wang in 2004 accounted an efficient method for the conversion of aldoximes (161) to nitriles (162) *via* the reaction of PA and aldoximes, in 1.01 : 1 molar ratio, which gained phthalic acid (163) as byproduct (Scheme 59).^173^

**Scheme 60:**
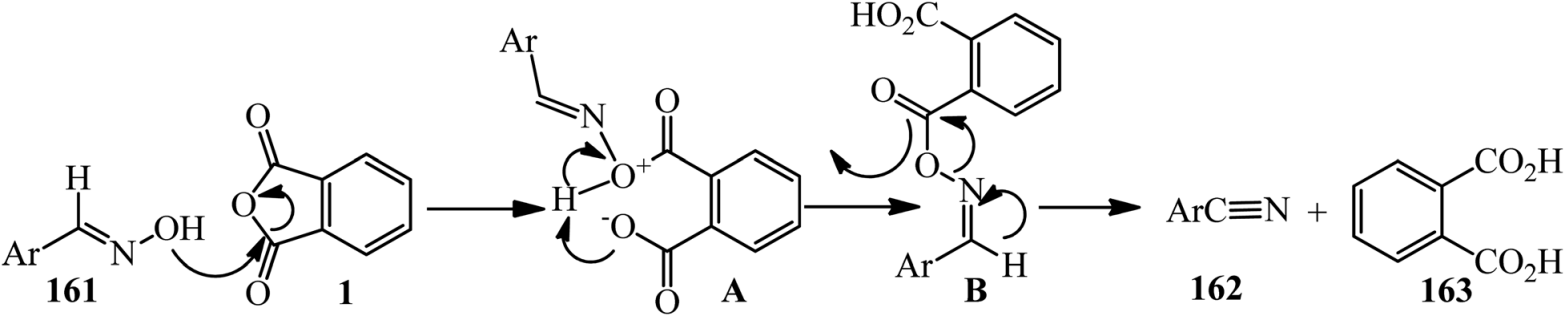
Proposed mechanism for the reaction of PA and aldoximes.

According to the proposed mechanism, using PA (1) led to formation of the acylated aldoxime intermediate (A), which efficiently underwent a feasible [3,3]-sigmatropic rearrangement as the reaction pathway *via* this proposed six-membered transition state (B), which gave nitriles (162) without utilizing an external base (Scheme 60).^173^

**Scheme 61:**
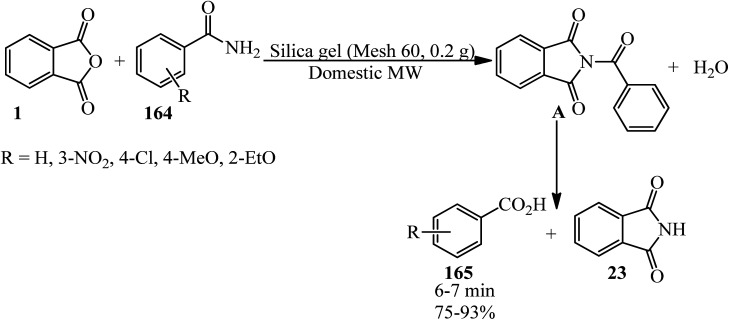
Transformation of amides to carboxylic acids.

Heravi group in 2005 transformed amides (164) to carboxylic acids (165) upon reaction with PA under microwave irradiation in the absence of solvent (Scheme 61).^174^ They utilized different amides (such as acetamide, acrylamide, and lactamide) successfully.

**Scheme 62:**
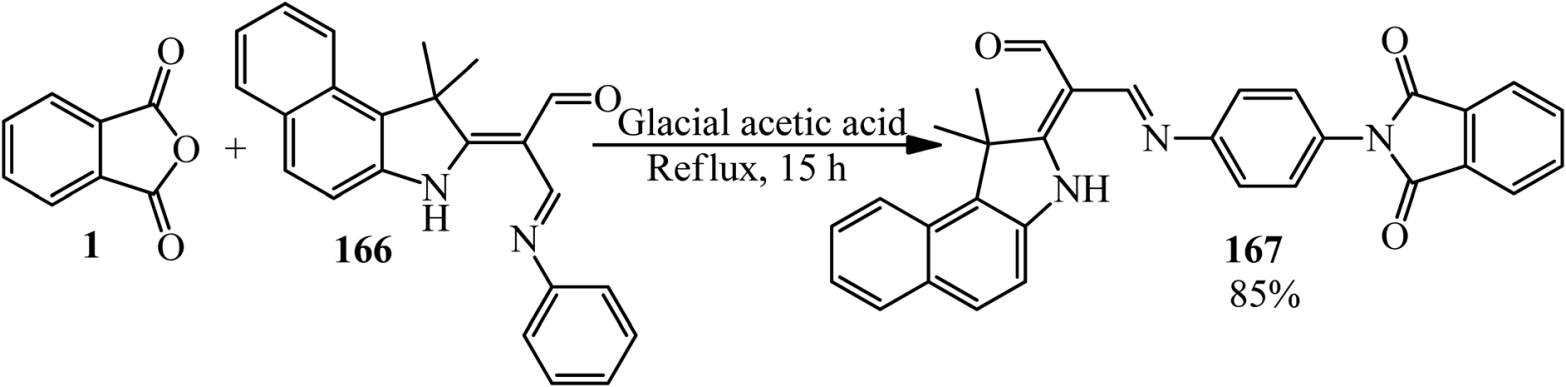
Synthesis of 2-(1,1-dimethyl-1*H*-benzo[*e*]indol-2(3*H*)-ylidene)-3-((4-(1,3-dioxoisoindolin-2-yl)phenyl)imino)propanal.

Kadhum group in 2022 supplied 2-(1,1-dimethyl-1*H*-benzo[*e*]indol-2(3*H*)-ylidene)-3-((4-(1,3-dioxoisoindolin-2-yl)phenyl)imino)propanal (167) *via* the reaction of PA (1) and Schiff base ((2*E*,3*E*)-2-(1,1-dimethyl-1*H*-benzo[*e*]indol-2(3*H*)-ylidene)-3-(phenylimino)propanal) (166) (Scheme 62).^175^

**Scheme 63:**
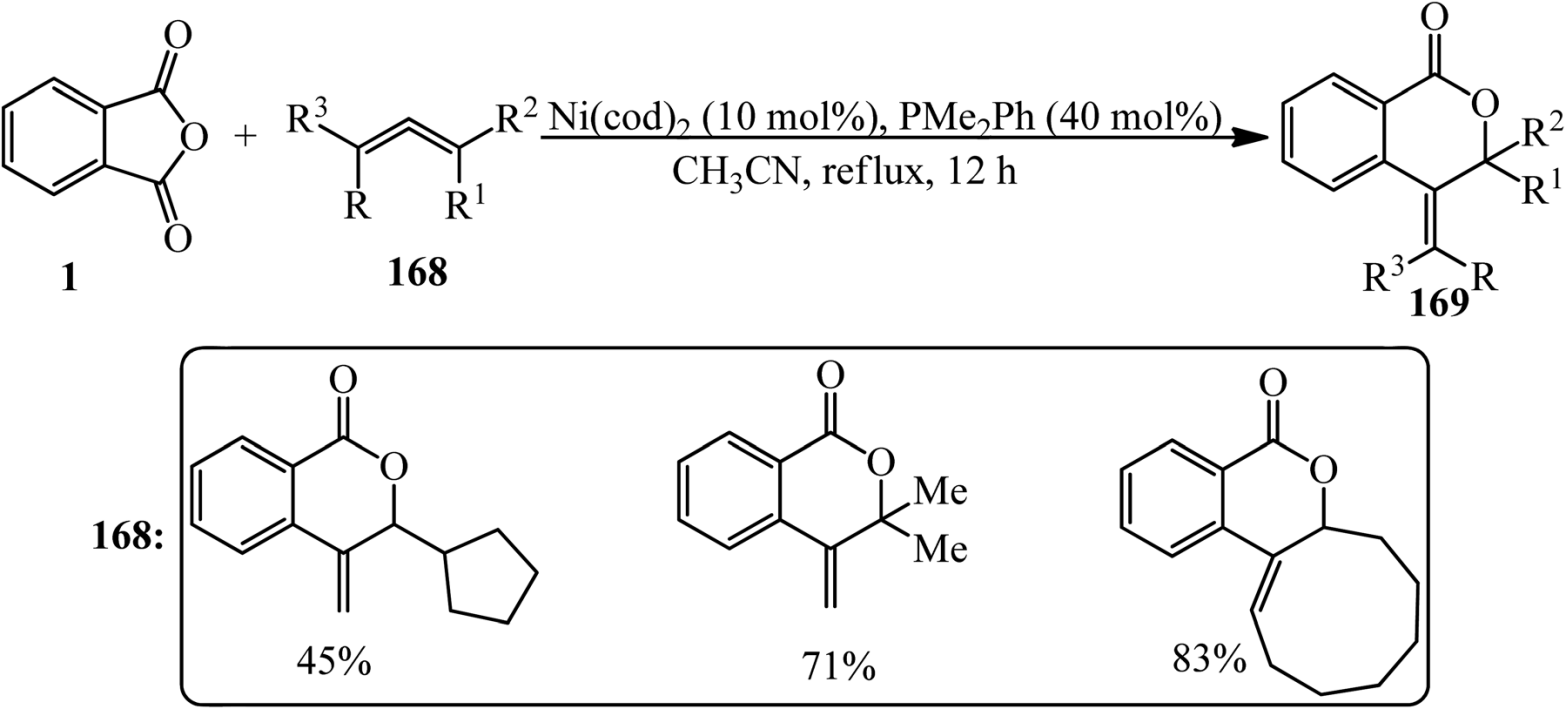
Decarbonylative cycloadditions of PA with allenes.

**Scheme 64:**
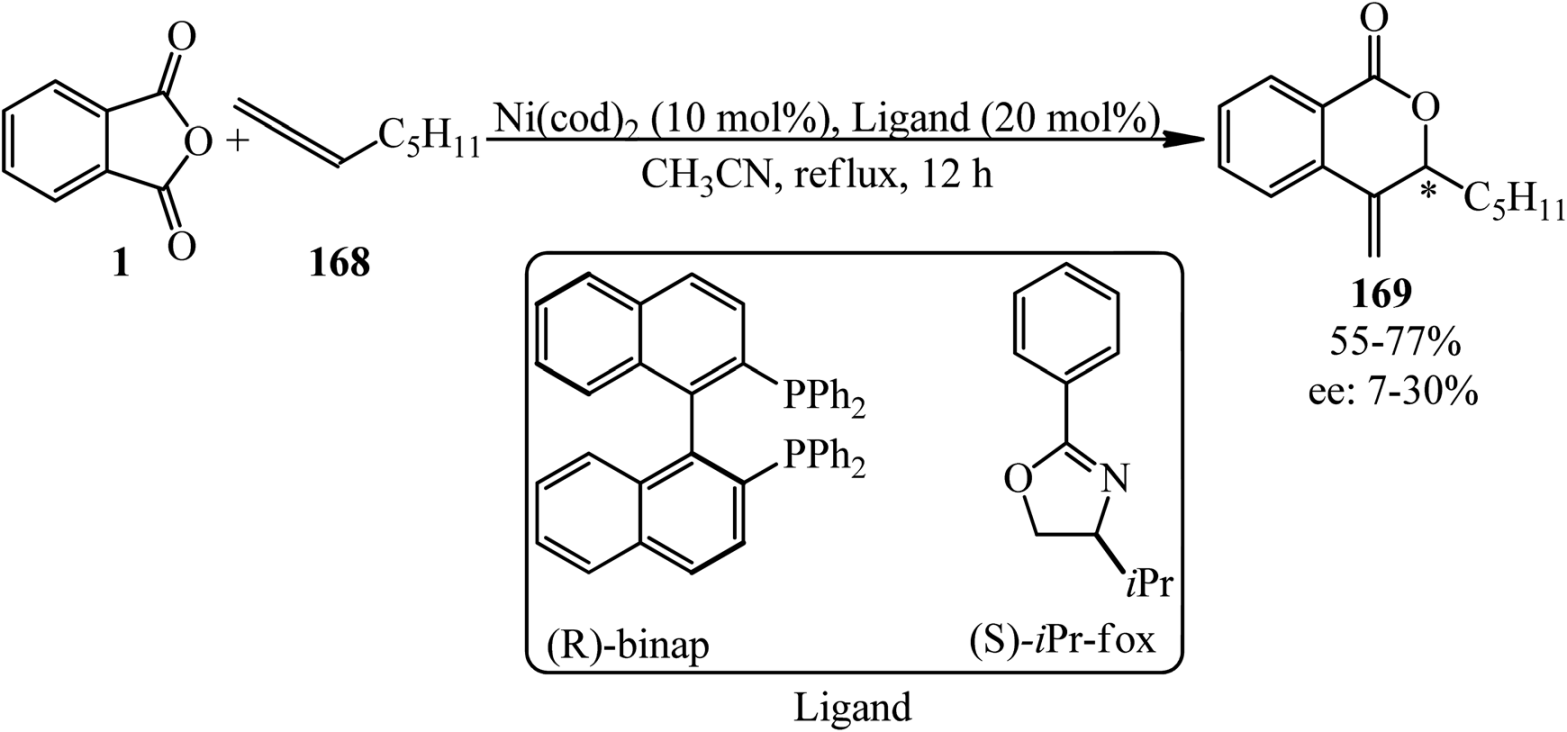
Enantioselective synthesis of δ-lactones using chiral phosphine ligands.

Matsubara group in 2011 dedicated the decarbonylative cycloadditions of PA (1) with allenes (168) in a 1 : 1.5 molar ratio using Ni(cod)_2_ as the Ni(0) precursor to give δ-lactones (169) in a single step. The reaction represented an unprecedented insertion reaction of a carbon–carbon double bond into a carbon–oxygen bond (Scheme 63).^176^ The asymmetric variant of the cycloaddition was also achieved using chiral phosphine ligands to provide δ-lactones enantioselectively (Scheme 64).^176^

**Scheme 65:**
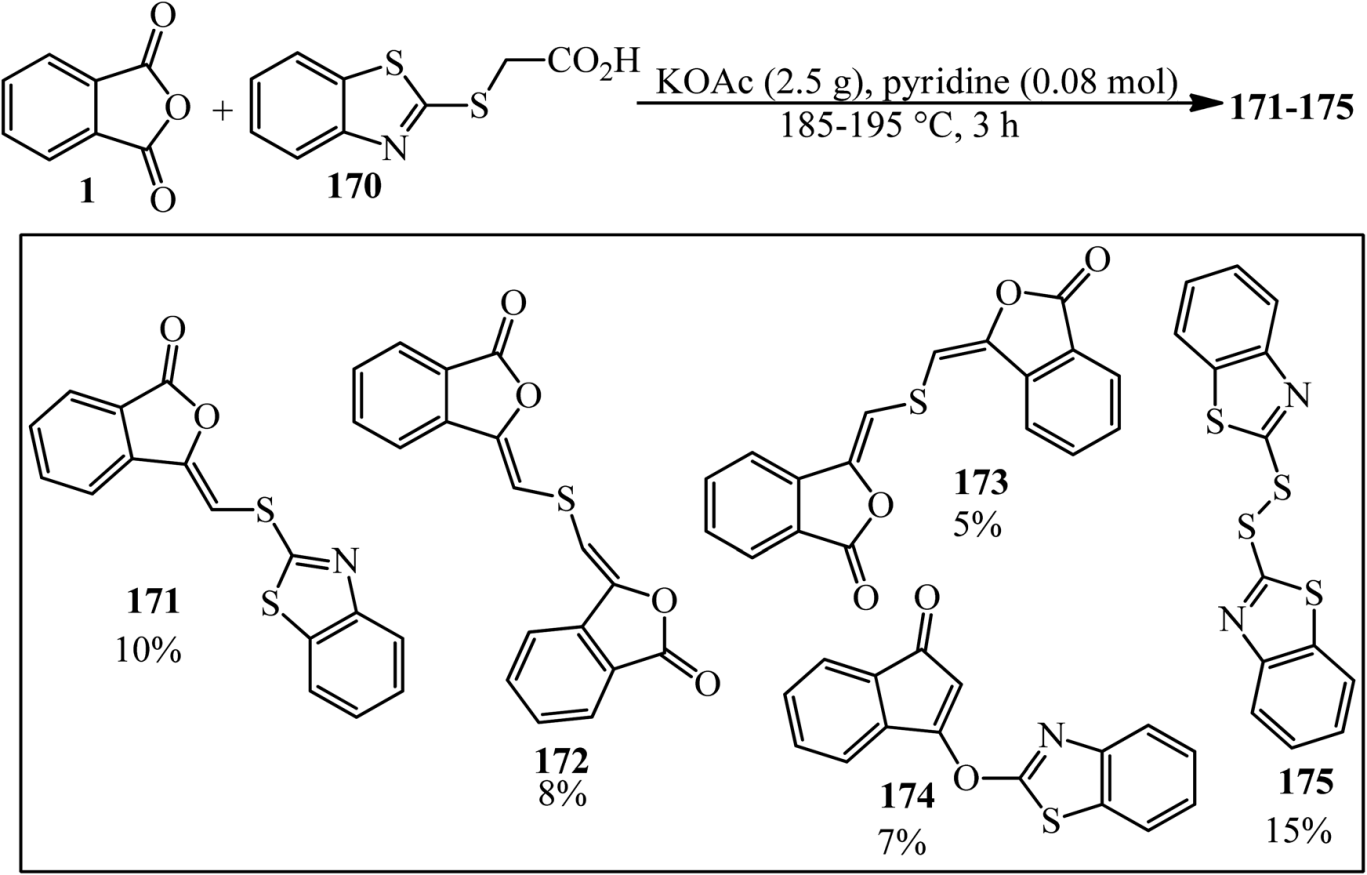
Reaction of 2-benzothiazolylthioethanoic acid with PA.

Lácová in 1986 considered the reaction of 2-benzothiazolylthioethanoic acid (170) (0.1 mol) with PA (0.2 mol) under conditions of Gabriel modification of Perkin synthesis. They claimed that in addition to the anticipated 3-(2-benzothiazolylthiomethylene)phthalide (171), four more compounds were identified as (*Z*,*Z*)-3,3′-thio-bis(methylenephthalide) (172), (*E*,*Z*)-3,3′-thio-bis(methylenephthalide) (173), l-(2-benzothiazolyloxy)-l-inden-3-one (174), and dibenzothiazolyl disulfide (175). The starting compounds did not react in the presence of acetic anhydride since 2-benzothiazolylthioethanoic acid (170) was preferentially acetylated to yield 4-methylthiazo[2,3-*b*]benzothiazolium 5-carboxylate by an intramolecular condensation. Only (*Z*)-3-(2-benzothiazolylthiomethylene)phthalide (171) corresponded to a normal course of aldol synthesis (Scheme 65).^177^

**Scheme 66:**
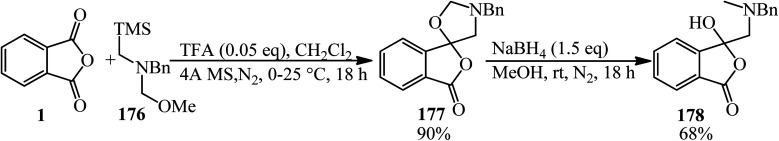
1,3-Dipolar cycloaddition of PA with *N*-benzylazomethine ylide.

Meyer and Ryan group in 2015 reported that PA (1 eq) underwent a 1,3-dipolar cycloaddition reaction with *N*-benzylazomethine ylide (that formed *in situ* from *N*-(methoxymethyl)-*N*-(trimethylsilylmethyl)benzylamine (176) (1.1 eq.) and a catalytic amount of trifluoroacetic acid) to produce unstable spiro(isobenzofuran-1,5′-oxazolidin)-3-ones (177), which was reduced with sodium borohydride to afford 1(3*H*)-isobenzofuranones (178) (Scheme 66).^178^

**Scheme 67:**
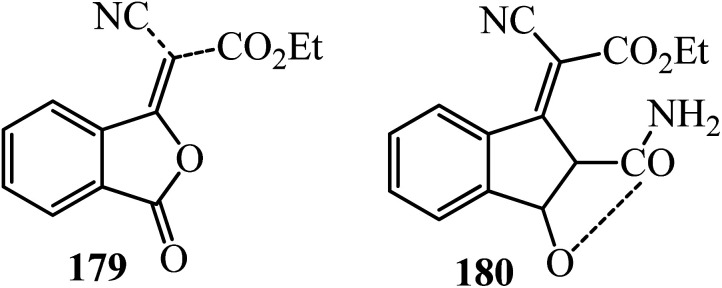
Ethyl cyano(phthalidy1idene)acetate and (2)-ethyl 2-carbamoyl-8-cyano-3-hydroxybenzofulvene-8-carboxylate.

Renfrew and Bostock in 1977 performed the Knoevenagel condensation of PA (1) with ethyl cyanoacetate (112) in the presence of sodium as the catalyst in benzene for 4 h to furnish ethyl cyano(phthalidy1idene)acetate (179) in 95% yield. Utilizing triethylamine as a base in toluene at 90 °C for 24 h, a yellow color was produced immediately, and upon refluxing, an intense orange color developed. On cooling, an orange oil separated, which on acidification gave a white solid in 64% yield, identified as (2)-ethyl 2-carbamoyl-8-cyano-3-hydroxybenzofulvene-8-carboxylate (180) (Scheme 67).^179^

**Scheme 68:**
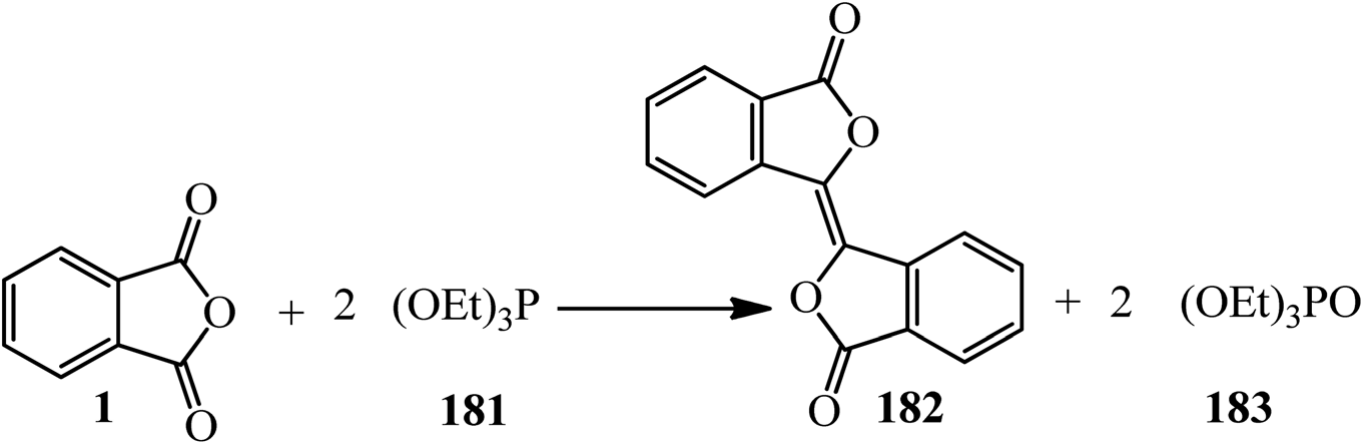
Synthesis of biphthalyl.

The Ramirez group in 1961 found that triethyl phosphite (181) effected the conversion of PA (1) in a 2 : 1 molar ratio into biphthalyl (182) in satisfactory yield (70%). The reaction was carried out in an excess of the phosphite as the solvent and the biphthalyl separated from the solution in nearly pure state. It was observed that most of the excess triethyl phosphite was isomerized to diethyl ethylphosphonate (183) during the reaction (Scheme 68).^180^

## 3. Applications of PA in three-component and pseudo three-component reactions

**Scheme 69:**
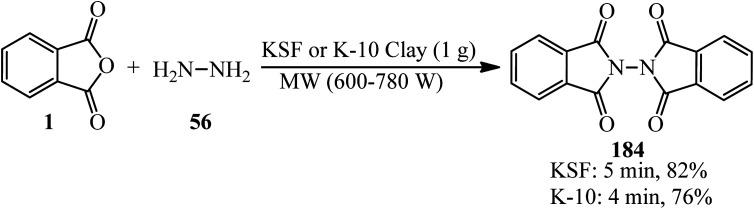
Synthesis of *N*-phthalimidophthalimide.

In 2006, Habibi and Marvi obtained *N*-phthalimidophthalimide (184) from the reaction of PA (1) and hydrazine hydrate (56) in 2 : 1 molar ratio in the presence of montmorillonite KSF and montmorillonite K-10 clays as natural heterogeneous catalysts with the help of microwave irradiation (600–780 W) under solvent-free conditions (Scheme 69).^181^ The pure product achieved through recrystallization by glacial acetic acid.

**Scheme 70:**
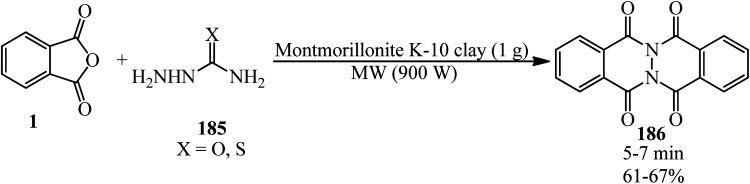
Synthesis of phthalazino[2,3-*b*]phthalazine-5,7,12,14-tetraone.

Habibi *et al.* in 2007 described a solvent-free and microwave-assisted procedure for the synthesis of phthalazino[2,3-*b*]phthalazine-5,7,12,14-tetraones (186) *via* the pseudo three-component reaction of PA (1) and (thio)semicarbazides (185) using montmorillonite K-10 clay. The catalyst demonstrated significant activity after recovering in another cycle (Scheme 70).^182^

Maccioni's group in 2003 also gained the commands (186) *via* the catalytic role of acetic acid in refluxing isopropyl alcohol media within 1 h with 61–75% yield.^183^ The antimicrobial properties of the products was also examined by the authors that was not satisfactory.

**Scheme 71:**
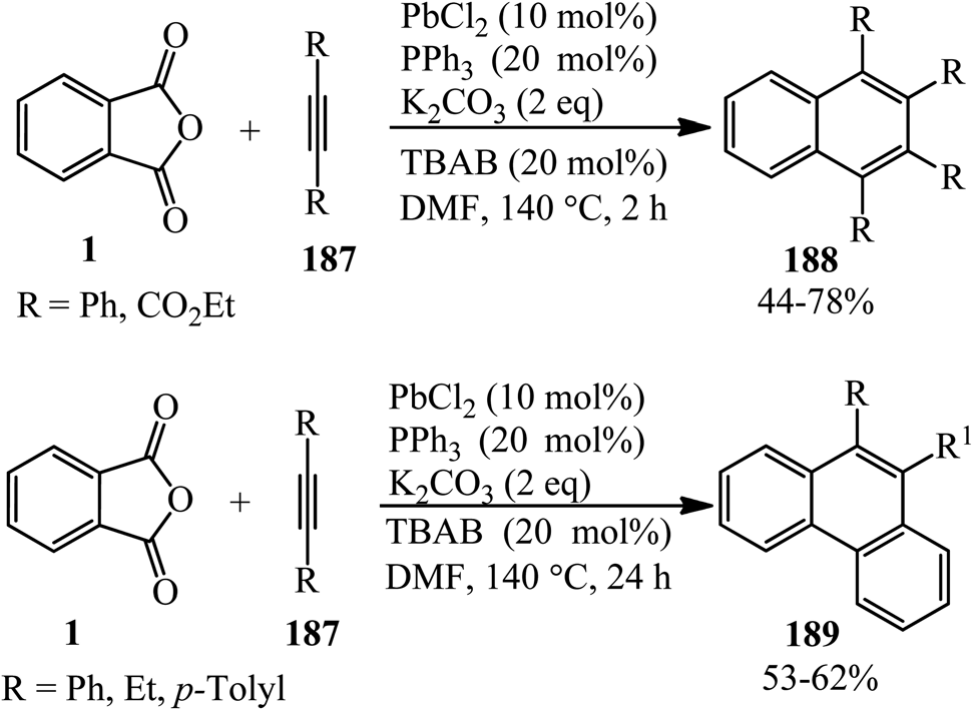
Synthesis of polyfunctionalized naphthalenes and phenanthrenes.

Jafarpour and coworkers in 2013 developed a new method for the decarboxylative and decarbonylative addition of cyclic anhydrides to alkynes. They performed the palladium-catalytic benzannulation of PA (1) with alkynes (187) in a 1 : 3 and 1 : 2 molar ratio to obtain the corresponding polyfunctionalized sterically condensed naphthalenes (188) and phenanthrenes (189), respectively. The sequential liberation of CO_2_ and CO occurred *via* the oxidative decomposition of anhydride (Scheme 71).^184^

**Scheme 72:**
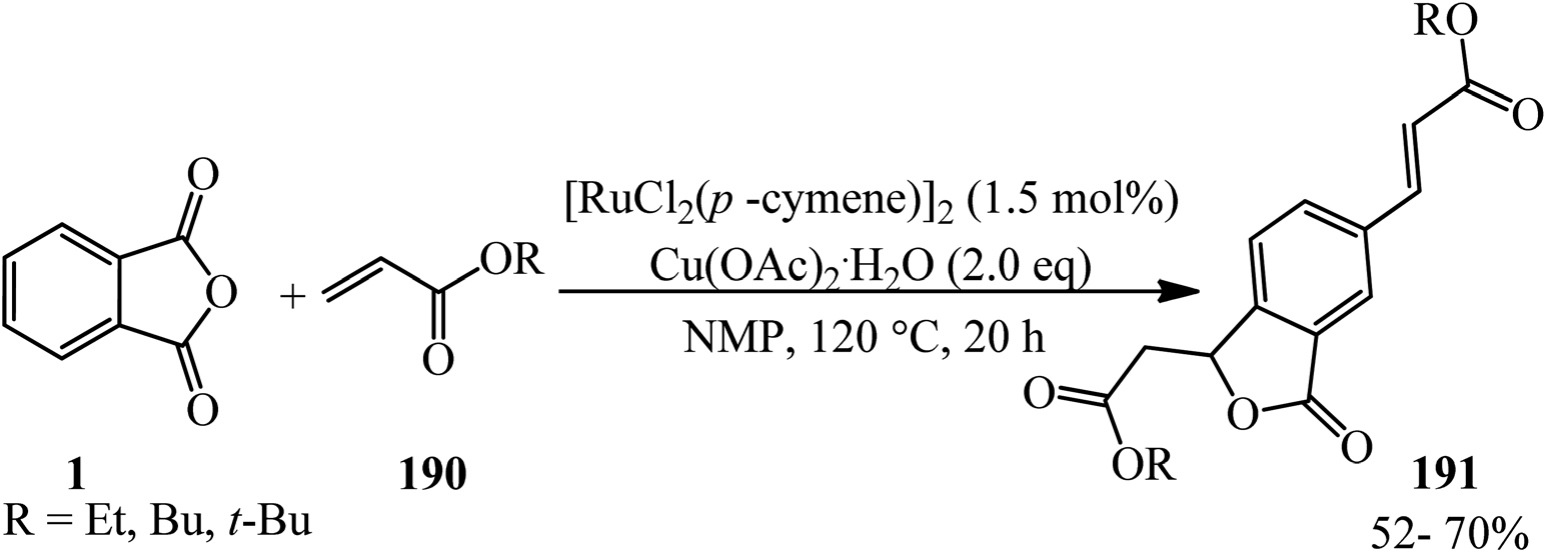
Synthesis of vinylated phthalides.

Fardpour *et al.* in 2019 constructed substituted vinylated phthalides (191) through a ruthenium-catalyzed cross-dehydrogenative coupling reaction of PA (1) with acrylates (190) in 1 : 2 molar ratio in the presence of Cu(OAc)_2_·H_2_O as the oxidant in *N*-methyl-2-pyrrolidone (NMP) solvent (Scheme 72).^185^ The reaction proceeded *via* C–H bond activation through a successive double vinylation accompanied by decarboxylation and annulation reaction.

**Scheme 73:**
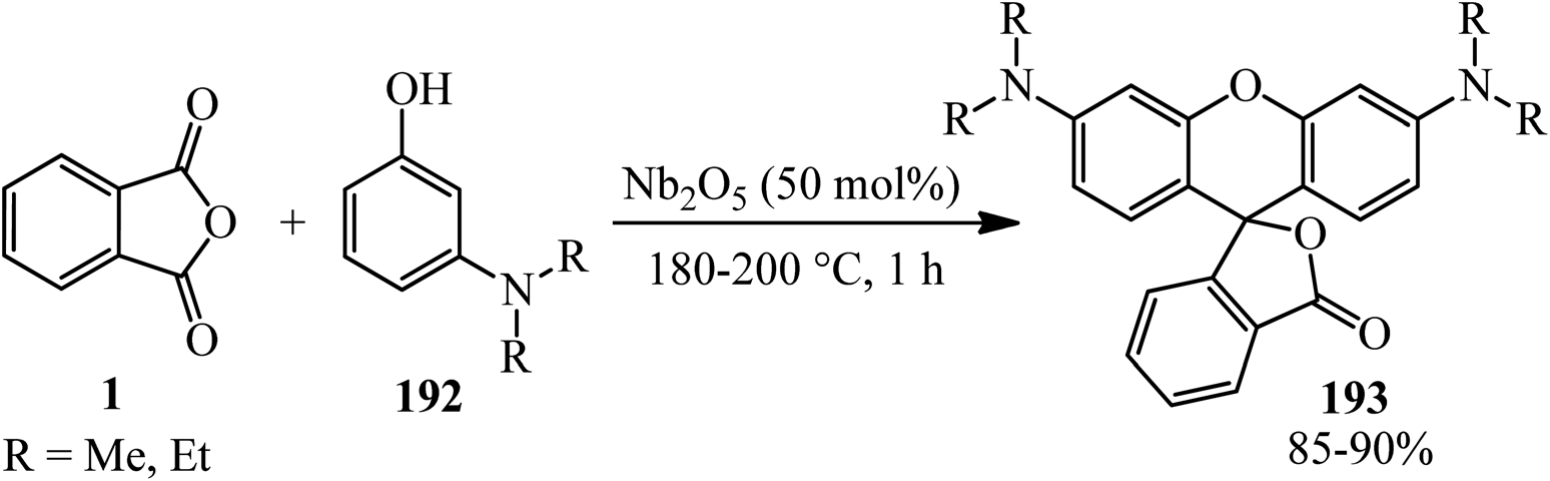
Synthesis of rhodamines.

Silva's research group in 2020 described the solvent-free synthesis of rhodamine dyes (193) *via* the reaction of PA and *m*-aminophenols (192) using Nb_2_O_5_ as the catalyst. The solvatochromic study of rhodamines was also performed (Scheme 73).^186^ Rhodamine dyes possessed various applications due to their properties, such as high molar absorptivity, high fluorescence quantum yield, photostability, and absorption and emission wavelengths in the visible region, which make them good candidates in electronic devices (such as lasers and OLEDs).

**Scheme 74:**
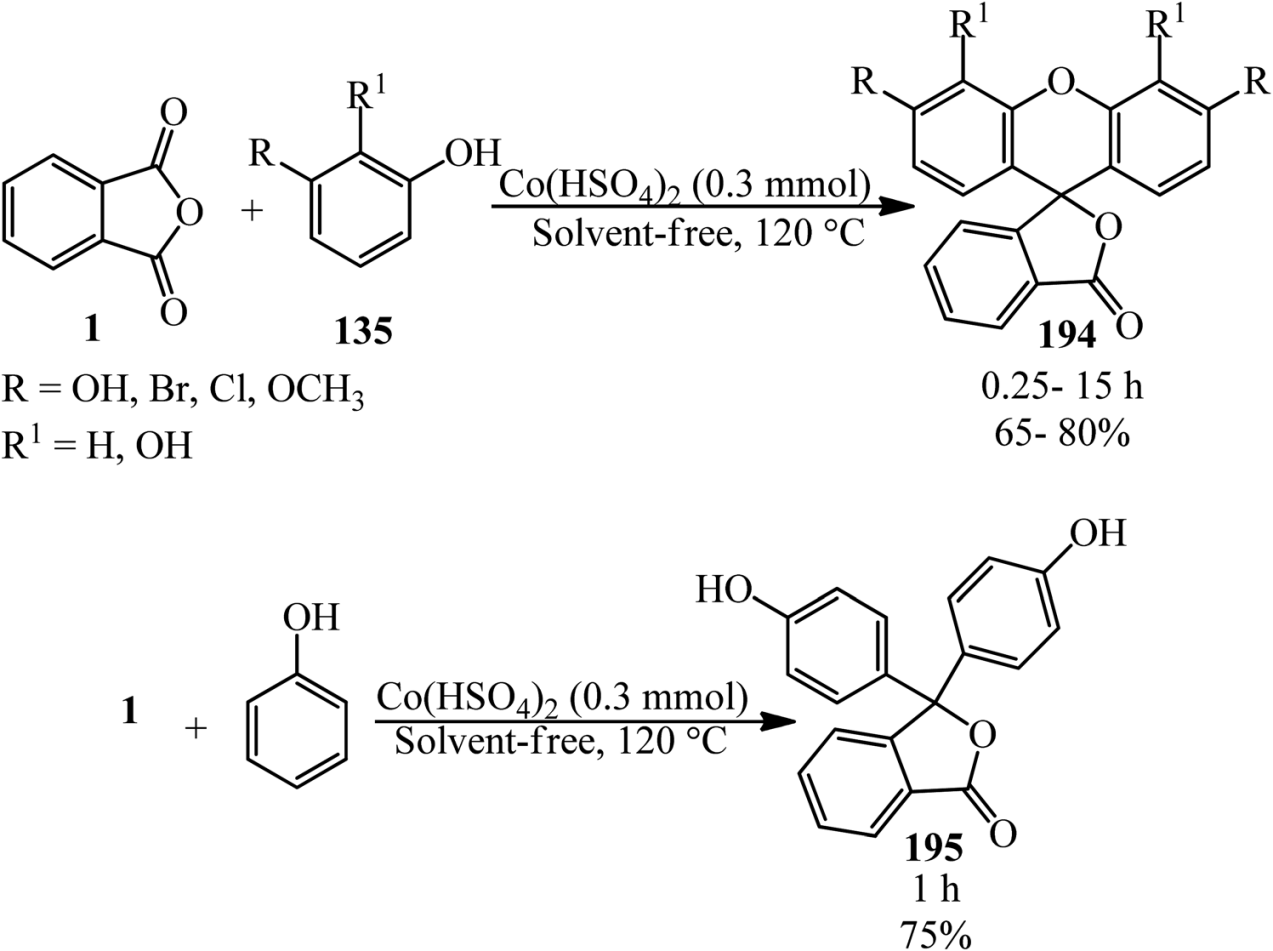
Synthesis of 3*H*-spiro[isobenzofuran-1,9′-xanthen]-3-ones and 3-bis(4-hydroxyphenyl)isobenzofuran-1(3*H*)-one.

Eshghi *et al.* in 2015 explained the condensation of PA (1) with substituted phenols (135) in the presence of cobalt hydrogen sulfate under melt conditions that gave 3*H*-spiro[isobenzofuran-1,9′-xanthen]-3-one (194). In the case of phenol, the product was 3,3-bis(4-hydroxyphenyl)isobenzofuran-1(3*H*)-one (195) (Scheme 74).^187^

**Scheme 75:**
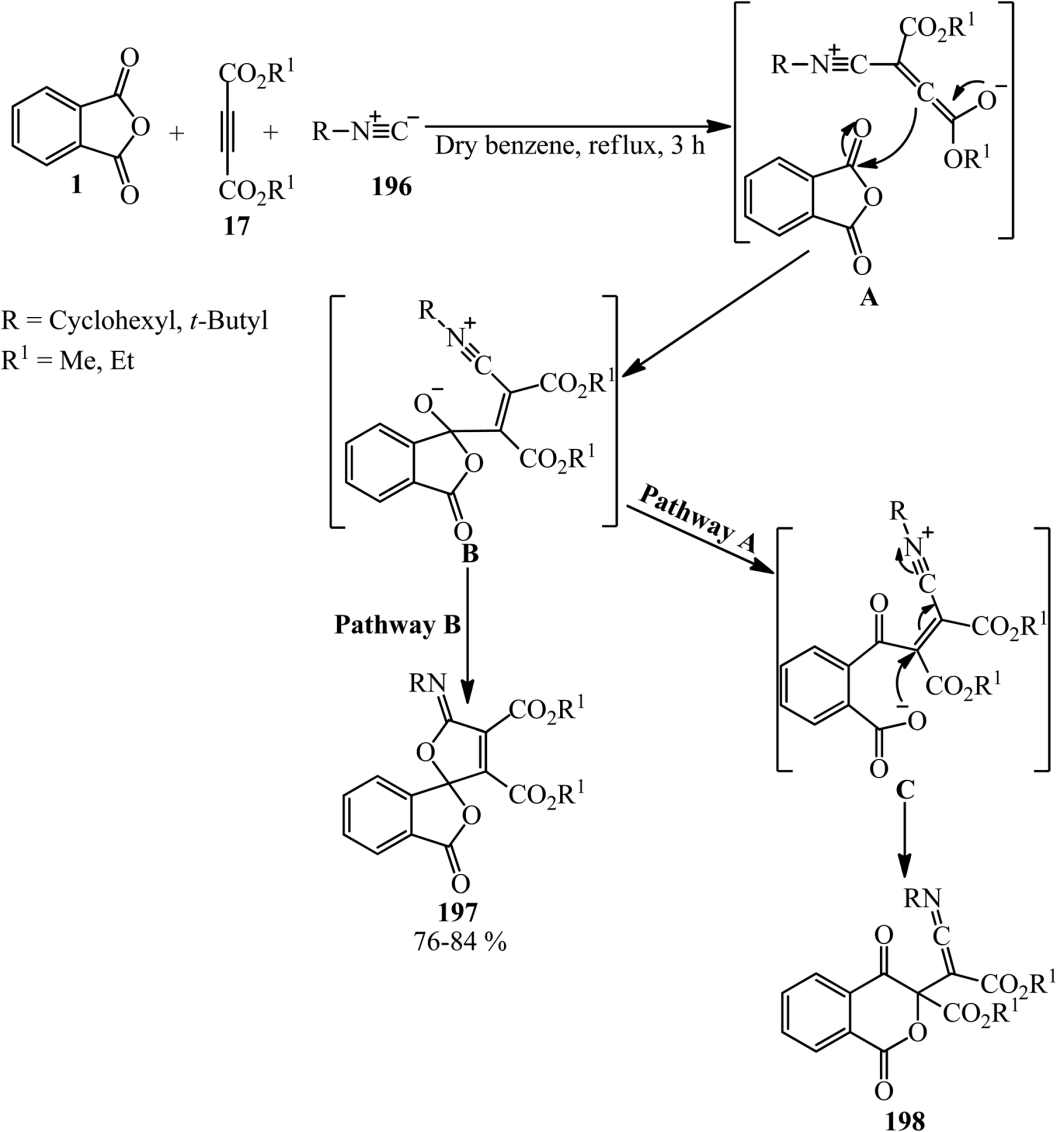
Synthesis of functionalized γ-spiroiminolactones.

Shaabani's research group in 2002 reported the addition of PA (1), dialkyl acetylenedicarboxylates (17) and alkyl isocyanides (196), leading to highly functionalized γ-spiroiminolactones (197). The authors purposed preparing highly functionalized ketenimines (198) (pathway A), but the unusual γ-spiroiminolactones (197) were obtained in high yields (pathway B) (Scheme 75).^188^

Shaabani *et al.* in 2009 also applied catalyst-free three-component method for the synthesis of a benzo-fused spirolacton (197) from the reaction of PA (1), dimethyl acetylenedicarboxylate (17), and cyclohexyl isocyanide (196) in dichloromethane at ambient temperature within 2 h with 82% yield. In fact, the zwitterion formed from isocyanide and dialkyl acetylenedicarboxylate reacted with PA to form the benzo-fused spirolactone (197).^189^

**Scheme 76:**
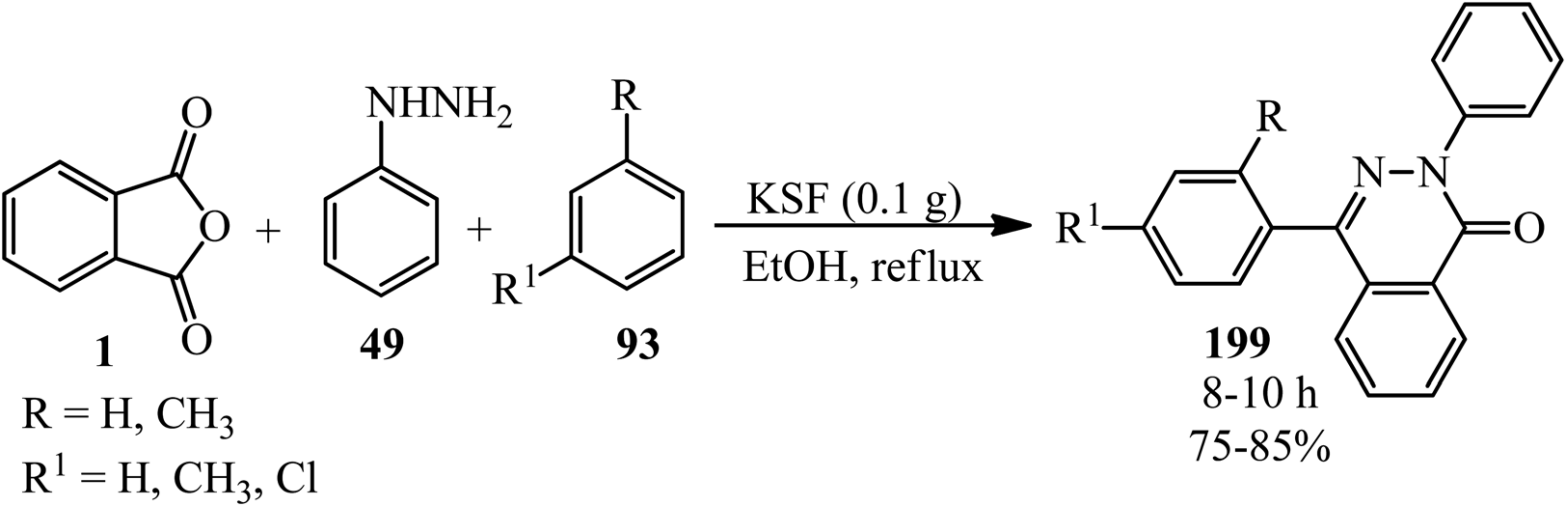
Synthesis of phthalazinones.

Mahmoodi's research group in 2010 demonstrated the regioselective synthesis of phthalazinones (199) from PA (1), phenyl hydrazine (49), and arenes (93) in the presence of efficient recyclable heterogeneous catalyst, montmorillonite-KSF, in high yields (Scheme 76).^190^

The same research group in 2012 also reported ultrasound-assisted (45 kHz) preparation of phthalazinones (199) in the presence of the recyclable catalyst [bmim]Br/AlCl_3_ (2 eq.) at 60 °C within 4–5 h by 65–75% yield.^191^

**Scheme 77:**
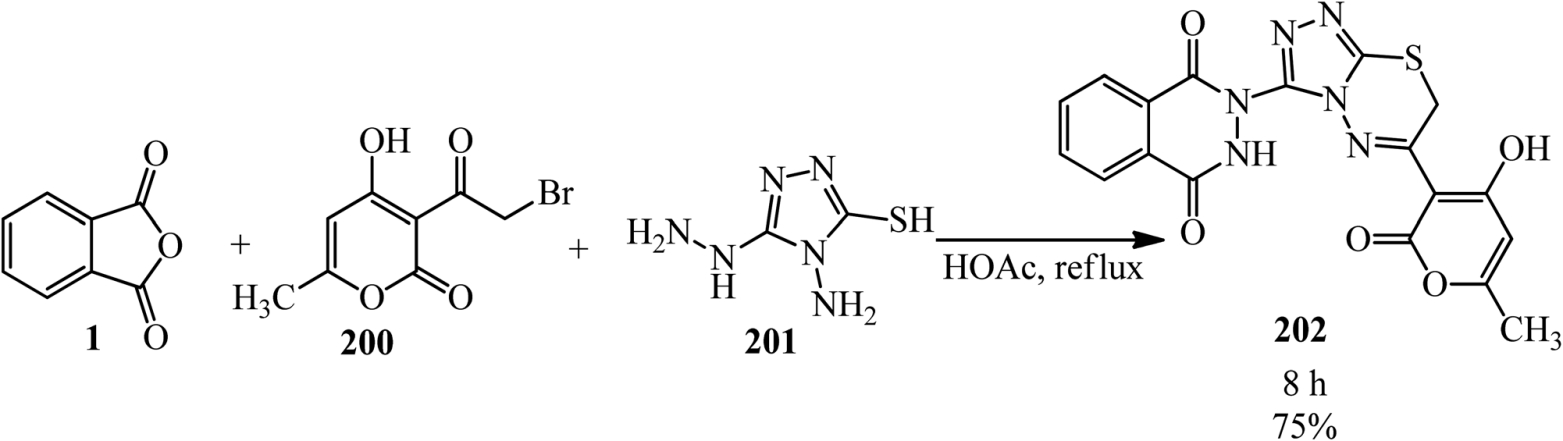
Synthesis of 2,3-dihydro-2-(6-(4-hydroxy-6-methyl-2-oxo-2*H*-pyran-3-yl)-7*H*-[1,2,4]-triazolo[3,4-*b*][1,3,4]thiadiazin-3-yl)phthalazine-1,4-dione.

Thirupaiah and Vedula in 2013 developed a facile and efficient one-pot, three-component protocol for the synthesis of novel 2,3-dihydro-2-(6-(4-hydroxy-6-methyl-2-oxo-2*H*-pyran-3-yl)-7*H*-[1,2,4]-triazolo[3,4-*b*][1,3,4]thiadiazin-3-yl)phthalazine-1,4-dione (202) from the reaction of PA (1), 3-(2-bromoacetyl)-4-hydroxy-6-methyl-2*H*-pyran-2-one (200), and 4-amino-5-hydrazino-4*H*-[1,2,4]triazole-3-thiol (201) in acetic acid medium (Scheme 77).^192^ The product was purified by simple recrystallization from ethanol.

**Scheme 78:**
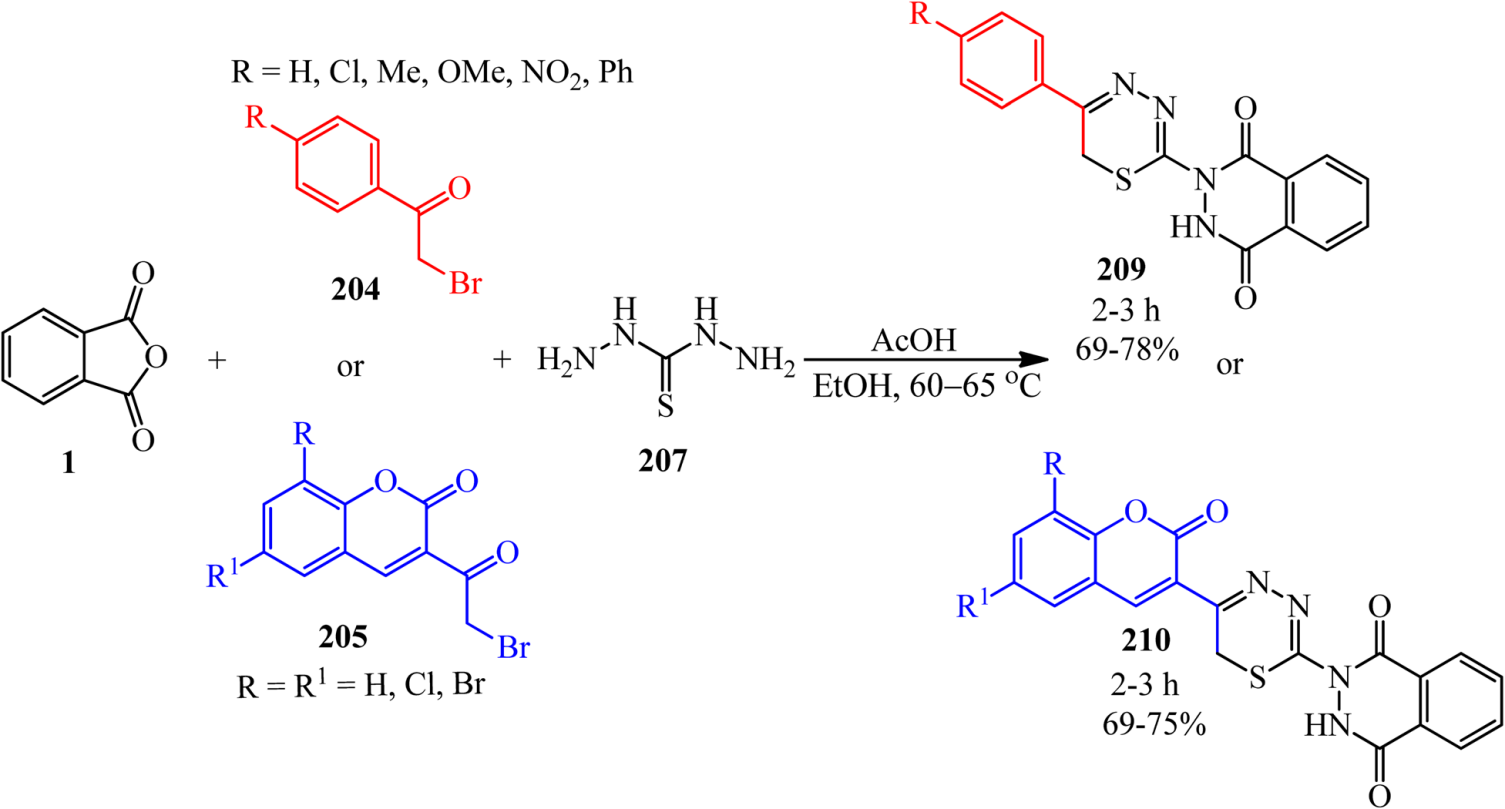
Synthesis of aryl(hetaryl)-substituted thiazolylphthalazine-1,4-diones.

Chunduru and Vedula in 2013 reported the synthesis of aryl(hetaryl)-substituted thiazolylphthalazine-1,4-diones (206, 207) *via* the reaction of PA (1), thiosemicarbazide (203), and phenacyl bromides (204)/3-(2-bromoacetyl)coumarins (205), respectively (Scheme 78).^193,194^

**Scheme 79:**
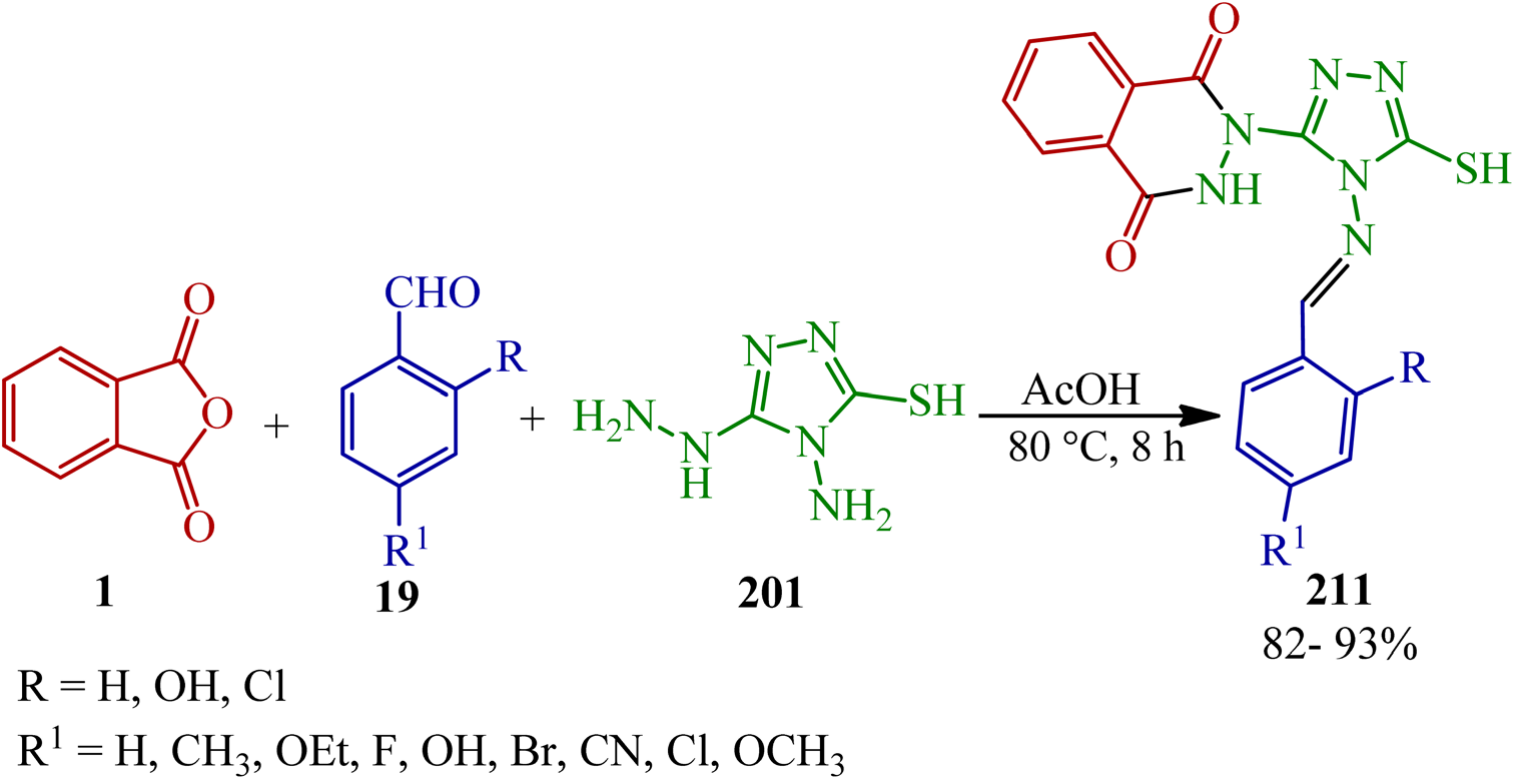
Synthesis of (*E*)-2-(benzylideneamino)-5-mercapto-4*H*-1,2,4-triazol-3-yl)-2,3 dihydrophthalazine-1,4-diones.

Sujatha and Vedula in 2019 presented a novel one-pot multicomponent method for the synthesis of (*E*)-2-((benzylideneamino)-5-mercapto-4*H*-1,2,4-triazol-3-yl)-2,3-dihydrophthalazine-1,4-diones (211) *via* the reaction of PA (1), aromatic aldehyde (19), and 4-amino-5-hydrazino-4*H*-1,2,4-triazole-3-thiol (201) (Scheme 79).^195^

**Scheme 80:**
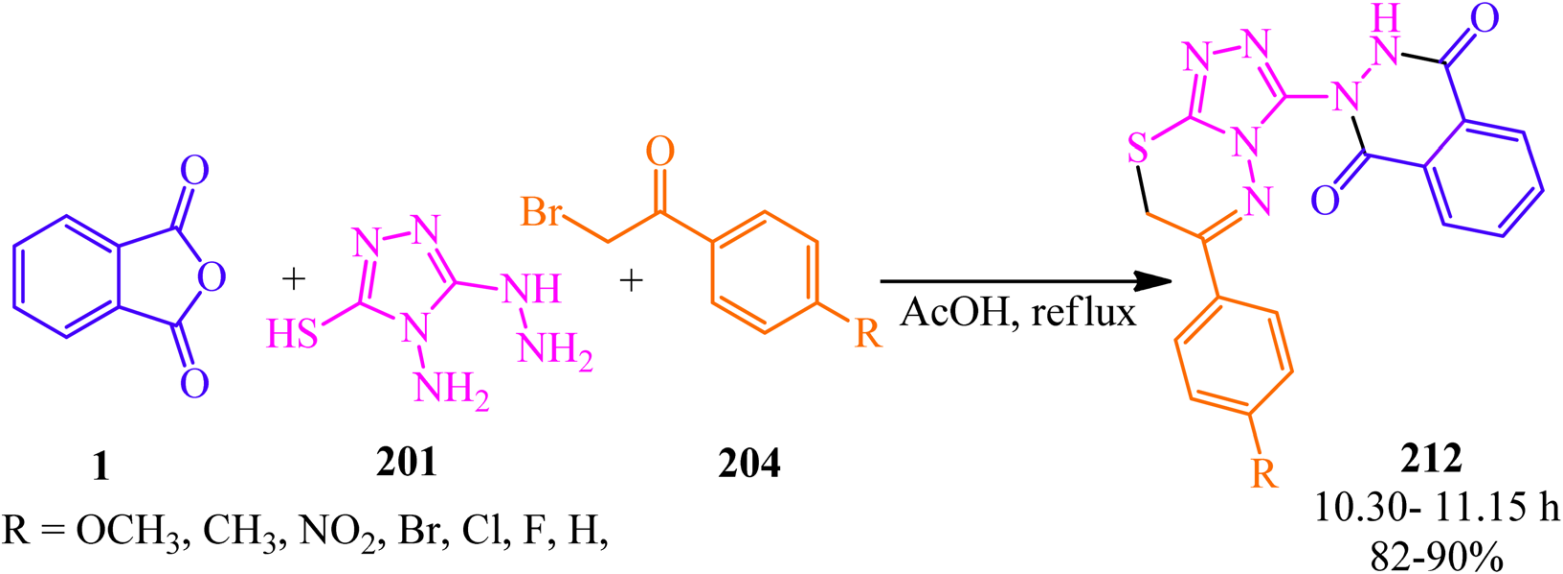
Synthesis of 2-(6-phenyl-7*H*-[1,2,4]triazolo[3,4-*b*][1,3,4]thiadiazin-3-yl)-2,3-dihydrophthalazine-1,4-diones.

Vedula *et al.* in 2020 developed the synthesis of a series of 2-(6-phenyl-7*H*-[1,2,4]triazolo[3,4-*b*][1,3,4]thiadiazin-3-yl)-2,3-dihydrophthalazine-1,4-diones (212) *via* a one-pot multicomponent reaction of PA (1), 4-amino-5-hydrazineyl-4*H*-1,2,4-triazole-3-thiol (201), and substituted 2-bromo-1-phenylethanones (204) in the presence of acetic acid under reflux conditions (Scheme 80).^196^

**Scheme 81:**
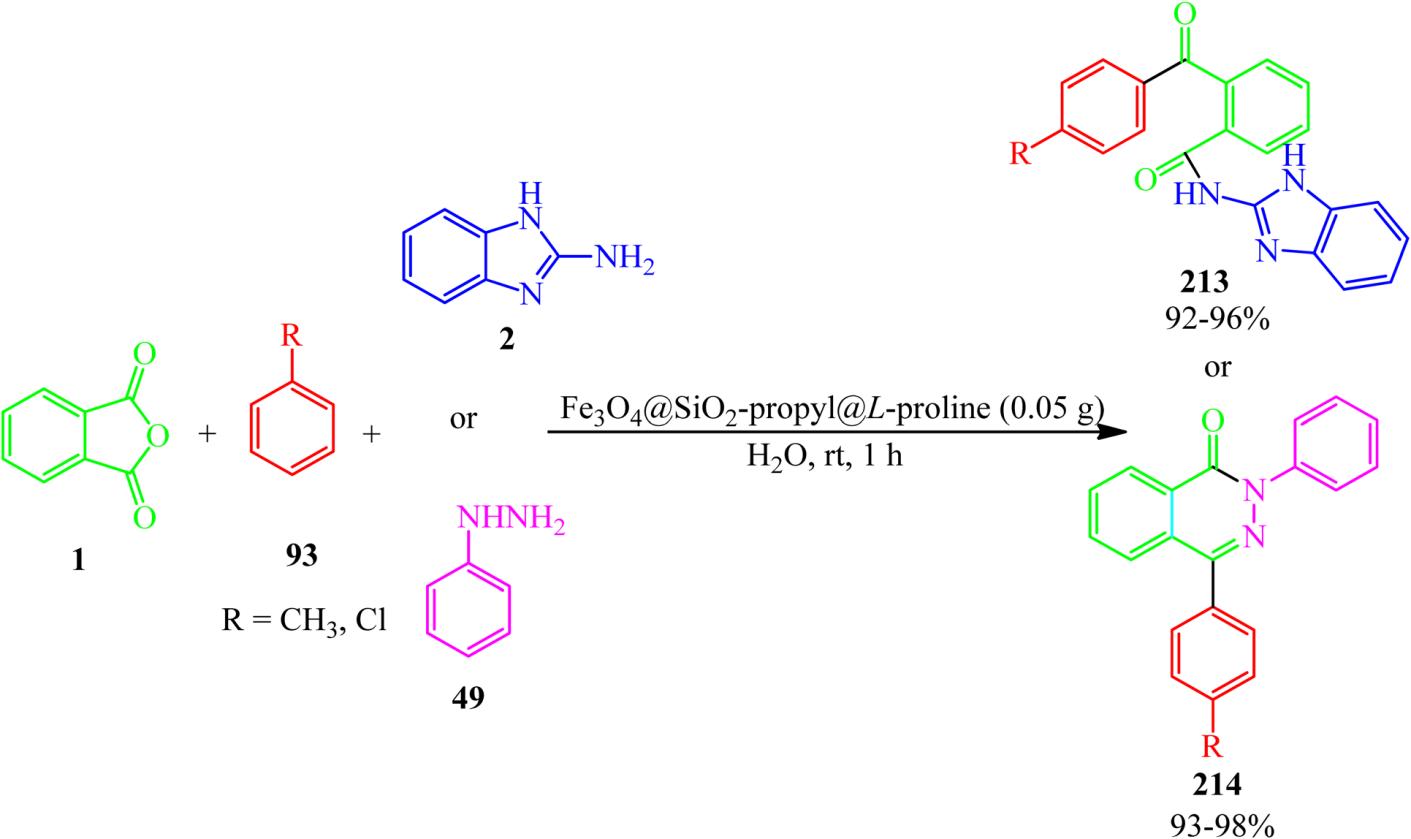
Synthesis of *N*-(1*H*-benzo[*d*]midazole-2-yl)-2-benzoylbenzamides and pyridazinones.

Zare Fekri and Farjood Feshalami in 2020 investigated the multicomponent reaction of PA (1) and 2-aminobenzimidazole (2), and arenes (93) to synthesize *N*-(1*H*-benzo[*d*]imidazol-2-yl)-2-benzoylbenzamides (213), and also, the multicomponent synthesis of pyridazinones (214) *via* the reaction of PA (1), phenyl hydrazine (49), and arenes (93) using l-proline-functionalized silicapropyl-modified nanomagnetic catalyst (Fe_3_O_4_@SiO_2_-propyl@l-proline) (Scheme 81).^197^

In 2021, they also obtained the amides (213) *via* the water-mediated the-component condensation of PA (1), 2-aminobenzimidazole (2), and (93) in the presence of nickel-ferrite silica-propyl supported glucosamine crystalline nanoparticles (NiFe_2_O_4_@SiO_2_-propyl@glucosamine) (0.05 g) as an efficient, reusable, and heterogeneous catalyst within 1 h with 92–96% yield.^198^

**Scheme 82:**
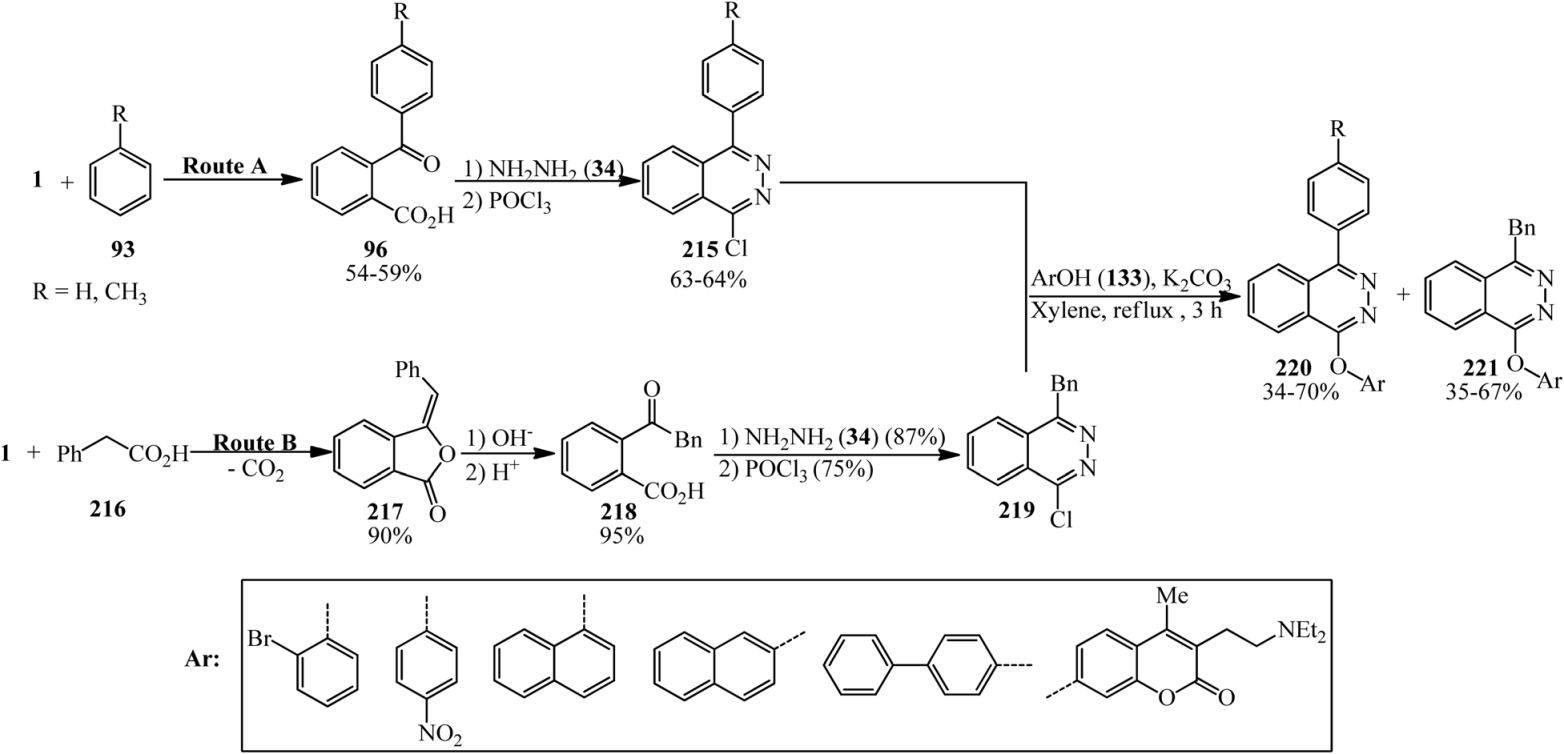
Multistep synthetic procedure of new phthalazines.

Bele and Darabantu in 2003 demonstrated the rapid synthesis of eighteen new 1,4-disubstituted phthalazines bearing an aryl or benzyl substituent at C-4 and a variety of aryloxy groups at C-l. The route A afforded the phthalazines (215) possessing a direct Ar–Ar linkage. Route B provided the l-chloro-4-benzylphthalazine (219). The neat condensation between (215, 219) and selected phenols (135) resulted in the decomposition of the reaction mixtures. Indeed, three series of new phthalazine derivatives were obtained in refluxing xylene (220, 221). All compounds were isolated simply by direct crystallization (Scheme 82).^199^

**Scheme 83:**
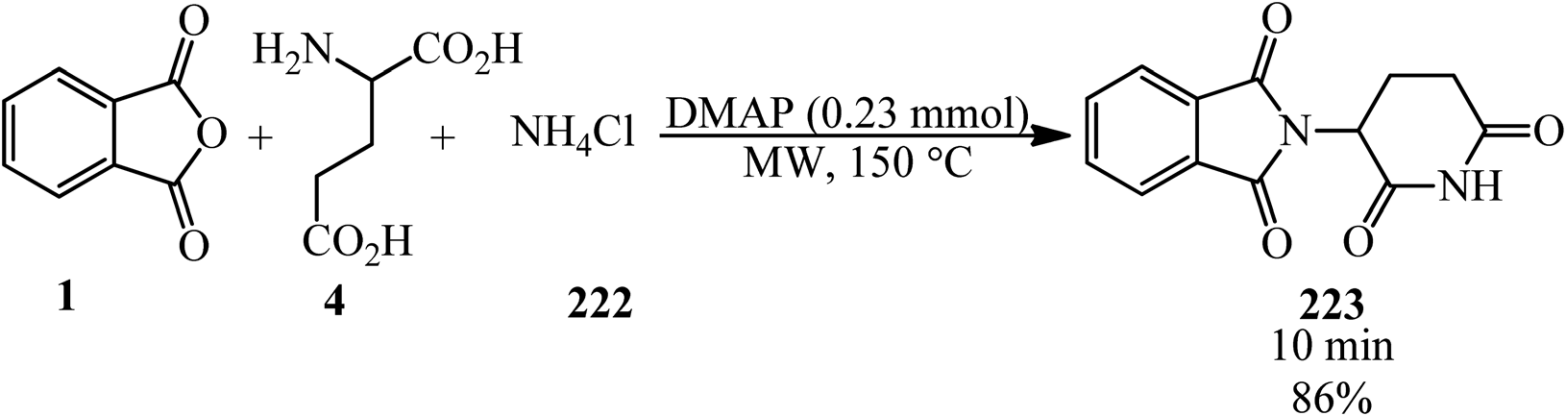
Synthesis of thalidomide.

Benjamin and Hijji in 2017 developed a novel green one-pot synthetic technique for the generation of thalidomide (223) *via* the reaction of PA (1), glutamic acid (4), and ammonium chloride (222) in a 1 : 1 : 1.1 molar ratio in the presence of catalytic amounts of 4-*N*,*N*-dimethylaminopyridine (DMAP) *via* microwave irradiation (Scheme 83).^200^

**Scheme 84:**
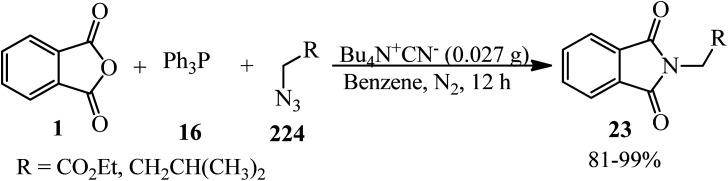
Synthesis of *N*-substituted phthalimides.

Garcia and Vilarrasa in 1986 prepared *N*-substituted phthalimides (23) *via* the reaction of PA (1), triphenylphosphine (16), and azides (224) in the presence of tetrabutyl ammonium cyanide as the catalyst and benzene or toluene as solvent (Scheme 84).^201^

**Scheme 85:**
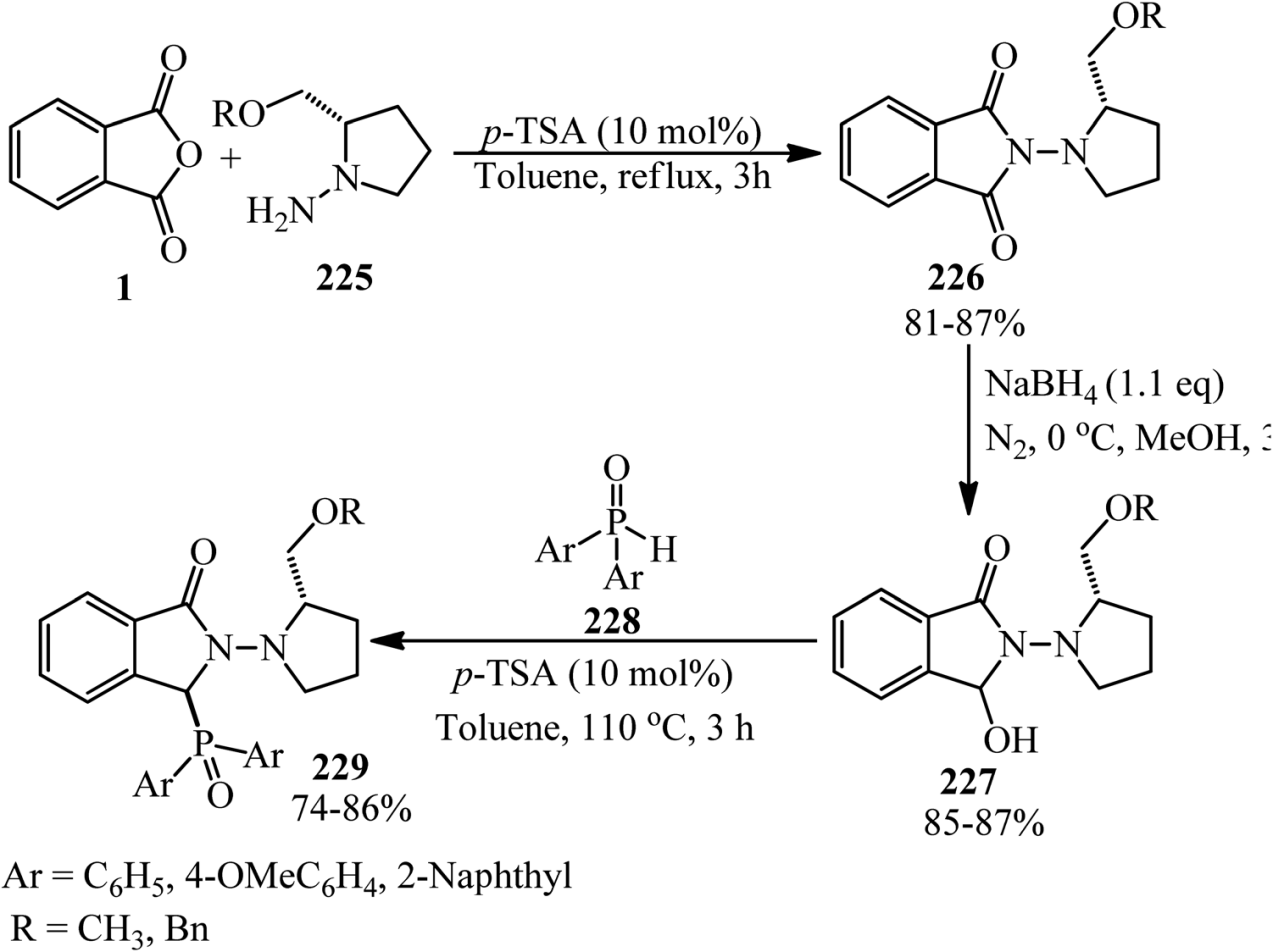
Synthesis of diarylphosphine oxide-substituted isoindolinones.

Deniau and coworkers in 2005 extended the asymmetric synthesis of diarylphosphine oxide-substituted isoindolinones (229) by a three-step reaction starting from PA (1) and (*S*)-1-amino-2-alkyloxymethylpyrrolidine (225) to prepare phthalhydrazides (226), which was reduced to 2-((*S*)-2-(alkoxymethyl)pyrrolidin-1-yl)-3-hydroxyisoindolin-1-one (227). In the second step, the final products (229) were obtained from the reaction of (227) with diarylphosphine oxides (228) (Scheme 85).^202,203^ The “de” of the products is more than 96% after recrystallization from hexane/toluene.

**Scheme 86:**
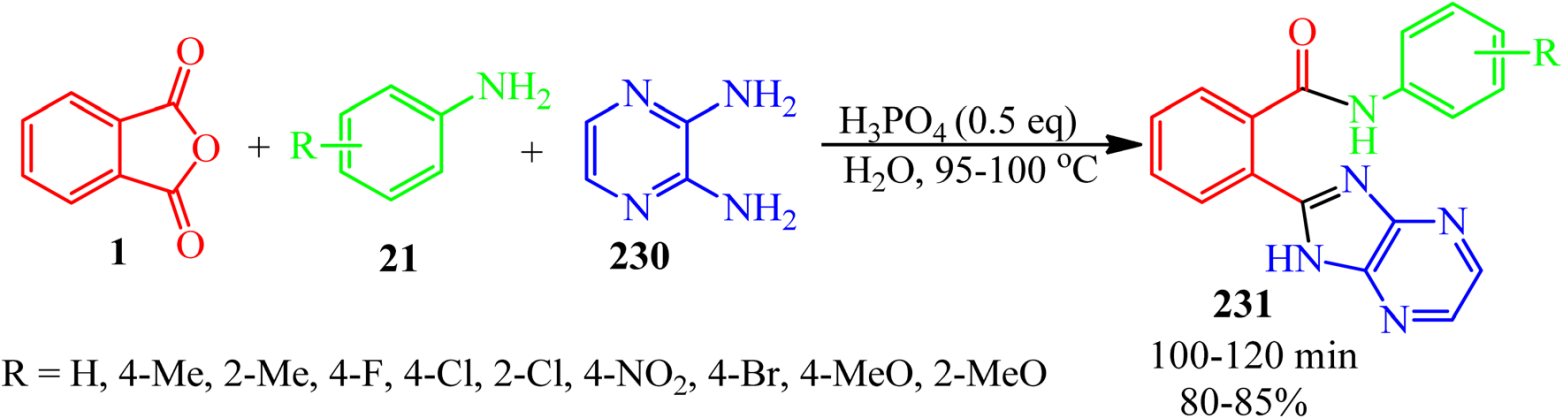
Synthesis of synthesis of imidazo[4,5-*b*]pyrazine-conjugated benzamides.

A green synthetic protocol was developed by Kalpana's group in 2021 for the synthesis of imidazo[4,5-*b*]pyrazine-conjugated benzamides (231) *via* the one-pot three-component reaction of PA (1), substituted anilines (21), and pyrazine-2,3-diamine (230) in the presence of phosphoric acid as the catalyst in heated water (Scheme 86).^204^ The products were evaluated for their anticancer activity against liver and ovarian cancer cell lines (HepG2 and HeLa), which demonstrated moderate to good activities. In addition, molecular modeling investigations affirmed the crucial binding interactions of the target protein and the synthesized ligands. In addition, the permeability and bioavailability properties were predicted along with molecular descriptors such as shape index, molecular complexity, and molecular flexibility.

**Scheme 87:**
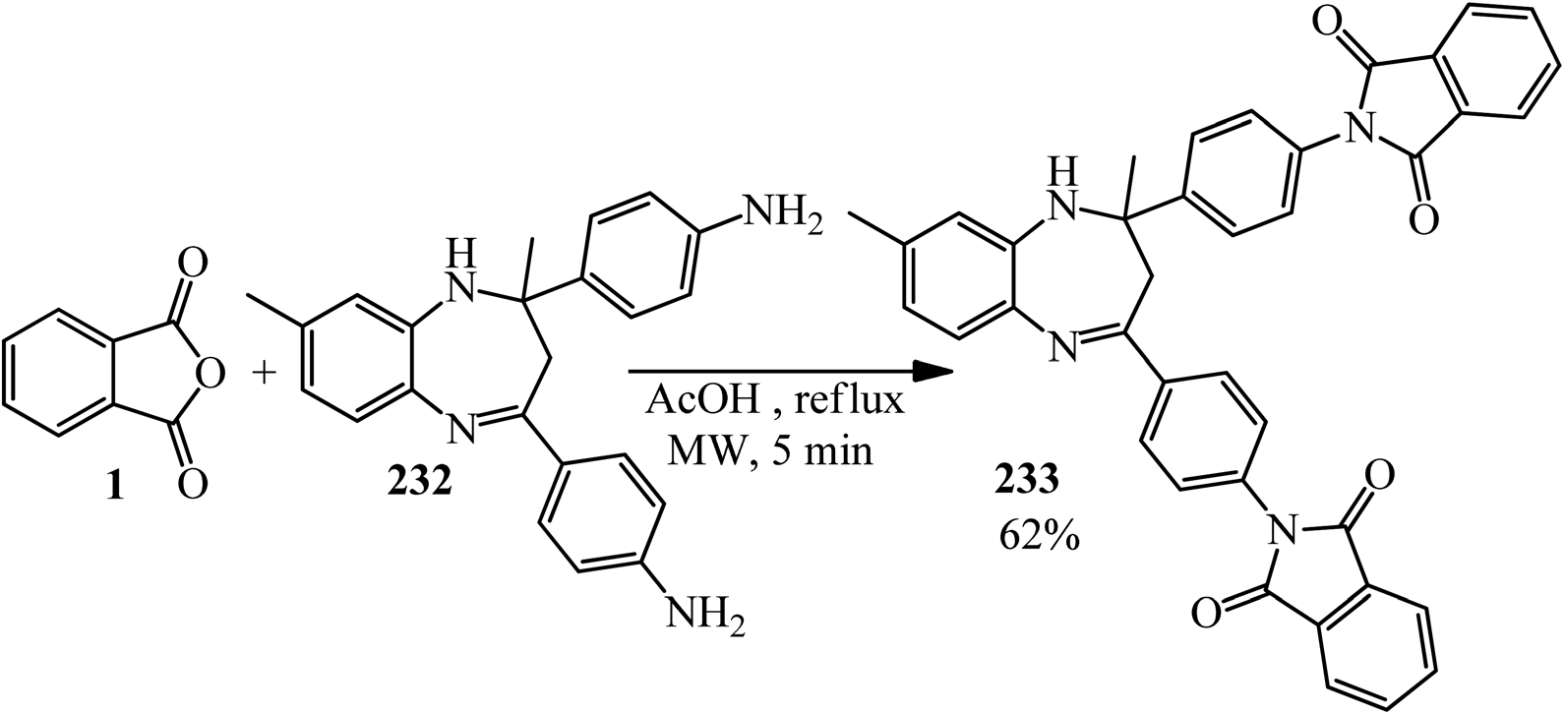
Synthesis of 2,2′-((2,8-dimethyl-2,3-dihydro-1*H*-benzo[*b*][1,5]diazepine-2,4-diyl)bis(4,1-phenylene))bis(isoindoline-1,3-dione).

Rasheed *et al.* in 2021 accomplished the reaction of PA (1) and 4,4′-(2,8-dimethyl-2,3-dihydro-1*H*-benzo[*b*][1,5]diazepine-2,4-diyl)dianiline (232) in 2 : 1 molar ratio in the refluxing acetic acid solvent under microwave irradiation to obtain 2,2′-((2,8-dimethyl-2,3-dihydro-1*H*-benzo[*b*][1,5]diazepine-2,4-diyl)bis(4,1-phenylene))bis(isoindoline-1,3-dione) (233) as a dark brown solid (Scheme 87).^205^

**Scheme 88:**
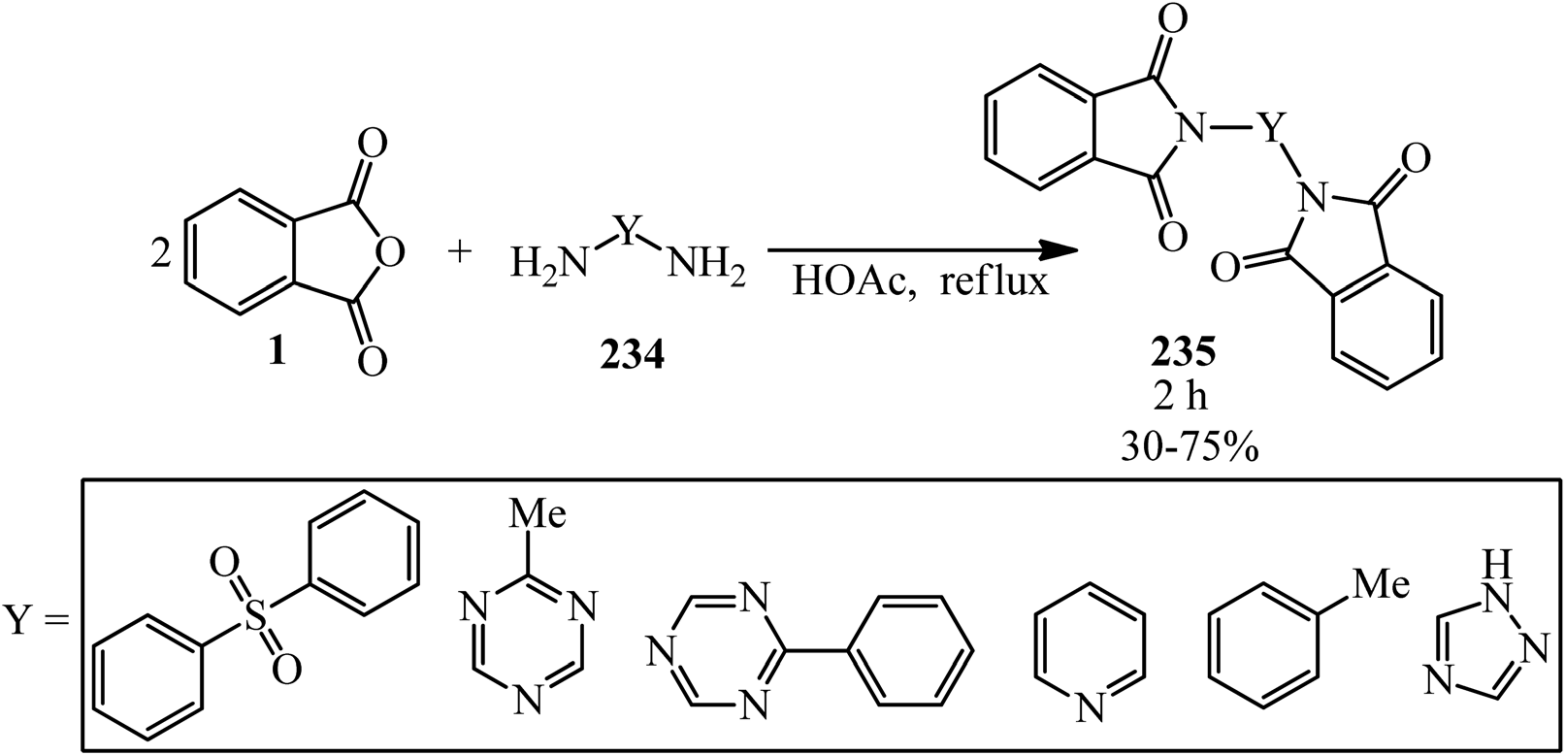
Preparation of *N*,*N*-substituted phthaldicarboximides.

Eissa in 2014 obtained six *N*,*N*-substituted phthaldicarboximides (235), which was synthesized from the reaction of PA (1) with diamines (234). The synthesized compounds were screened for their antibacterial activity against four microorganisms, namely, *Staphylococcus aureus*, *Bacillus subtilis*, *Escherichia coli*, and *Klebsiella pneumonia*, and they were found to exhibit good to moderate antibacterial activity (Scheme 88).^206^

**Scheme 89:**
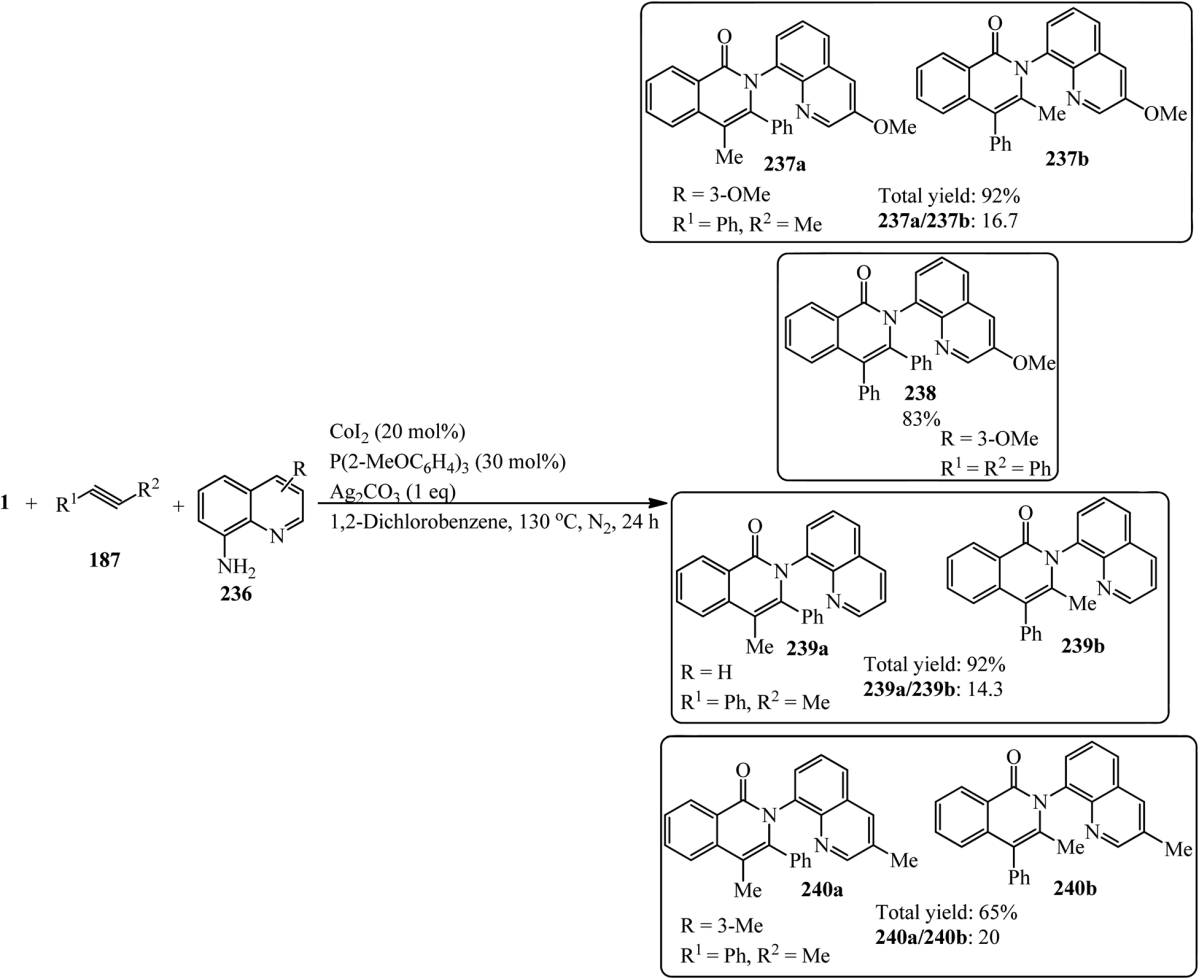
Regioselective synthesis of polysubstituted isoquinolones.

Chen's group in 2020 described an efficient cobalt-catalyzed intermolecular decarbonylative three-component carboamidation of alkynes (187) through the oxidative addition of cobalt into the N–C(O) bond of phthalimide and the subsequent decarbonylation. High regioselectivities achieved for unsymmetrical alkynes (including aryl-alkyl or aryl–aryl) to deliver polysubstituted isoquinolones (237–240) (Scheme 89).^207^

**Scheme 90:**
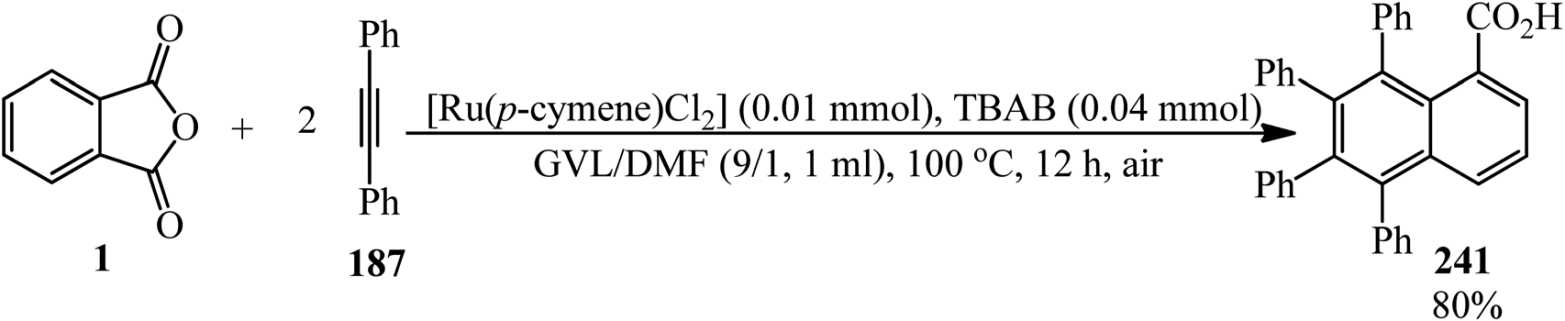
Benzannulation of PA with alkyne.

Zheng's group in 2019 described the efficient [2 + 2 + 2] benzannulation of PA (1) (or phthalic acid) with alkynes (187) in 1 : 1.33 molar ratio to prepare multisubstituted 1-naphthoic acids (241) *via* Ru-catalyzed C–H activation. The reaction proceeded well using atmospheric oxygen as the sole oxidant with high atom/step economies (Scheme 90).^208^

**Scheme 91:**
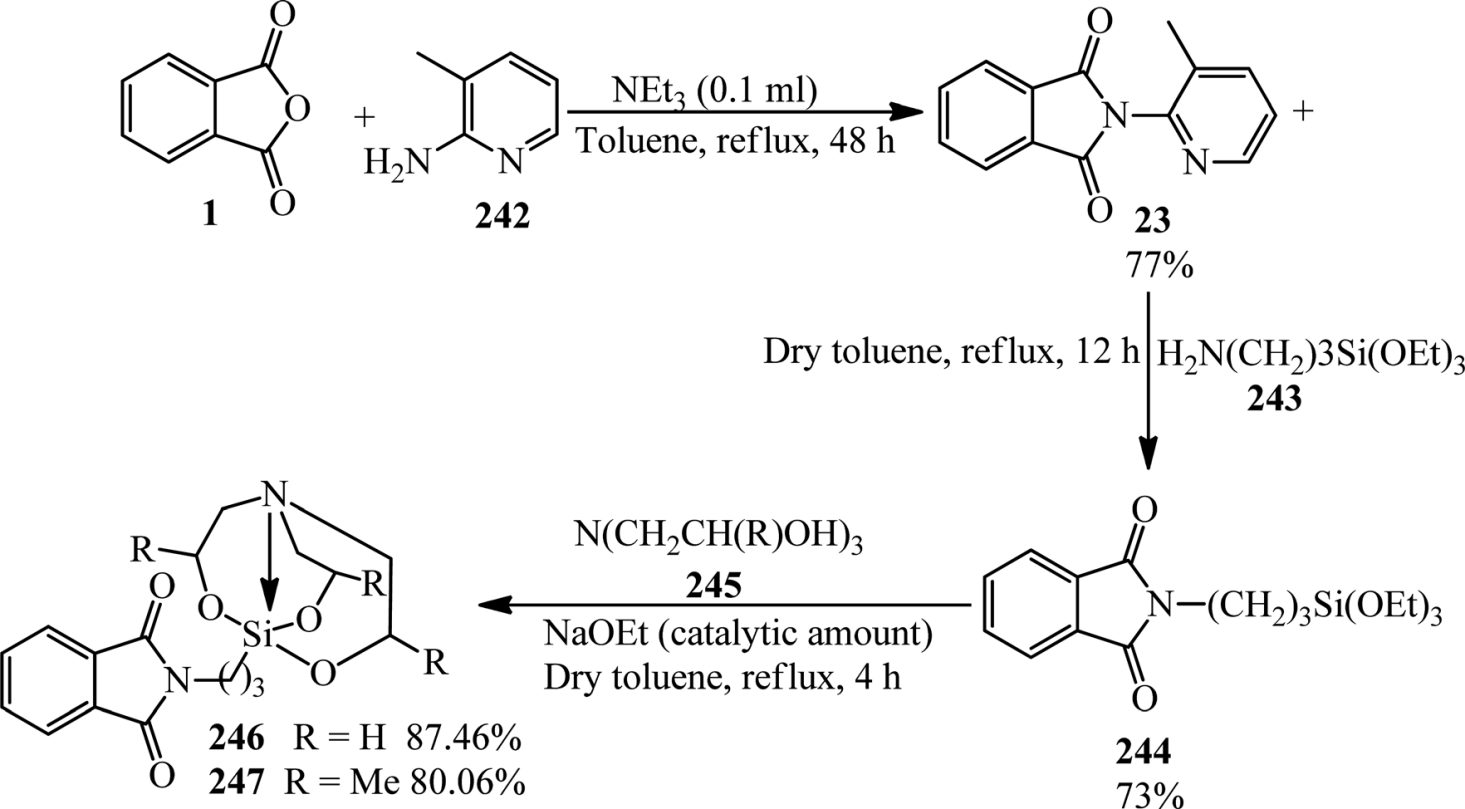
Transimidization reaction of PA, 2-amino-3-picoline, and 3-aminopropyl triethoxysilane.

Singh's group in 2015 demonstrated the transimidization reaction between PA (1), 2-amino-3-picoline (242), and 3-aminopropyl triethoxysilane (243) in a 1 : 1 : 1 : 1.125 molar ratio, which transited from 2-(3-methylpyridin-2-yl)isoindoline-1,3-dione (23) though a two-step reaction to synthesize *N*-(triethoxysilylpropyl)phthalimide (244). New silatranes (246, 247) containing phthalimide as the exocyclic group were prepared by the transesterification reactions of (244) with triethanolamine/trisisopropanolamine (245), respectively. *N*-(silatranylpropyl)phthalimides (246, 247) were evaluated for the preliminary antimicrobial activity using broth microdilution method, which shows that silatranes-possessing urea group exhibited good antimicrobial activity. The investigation of the UV-vis spectra of the silatranes proved fruitful for analyzing the hydrogen bonding of trifluroacetic acid with phthalimide heterocycle possessing silatranes. Hydrogen bonding has shown major influence upon n → π* transition, resulting in blueshift phenomenon (Scheme 91).^209^

**Scheme 92:**
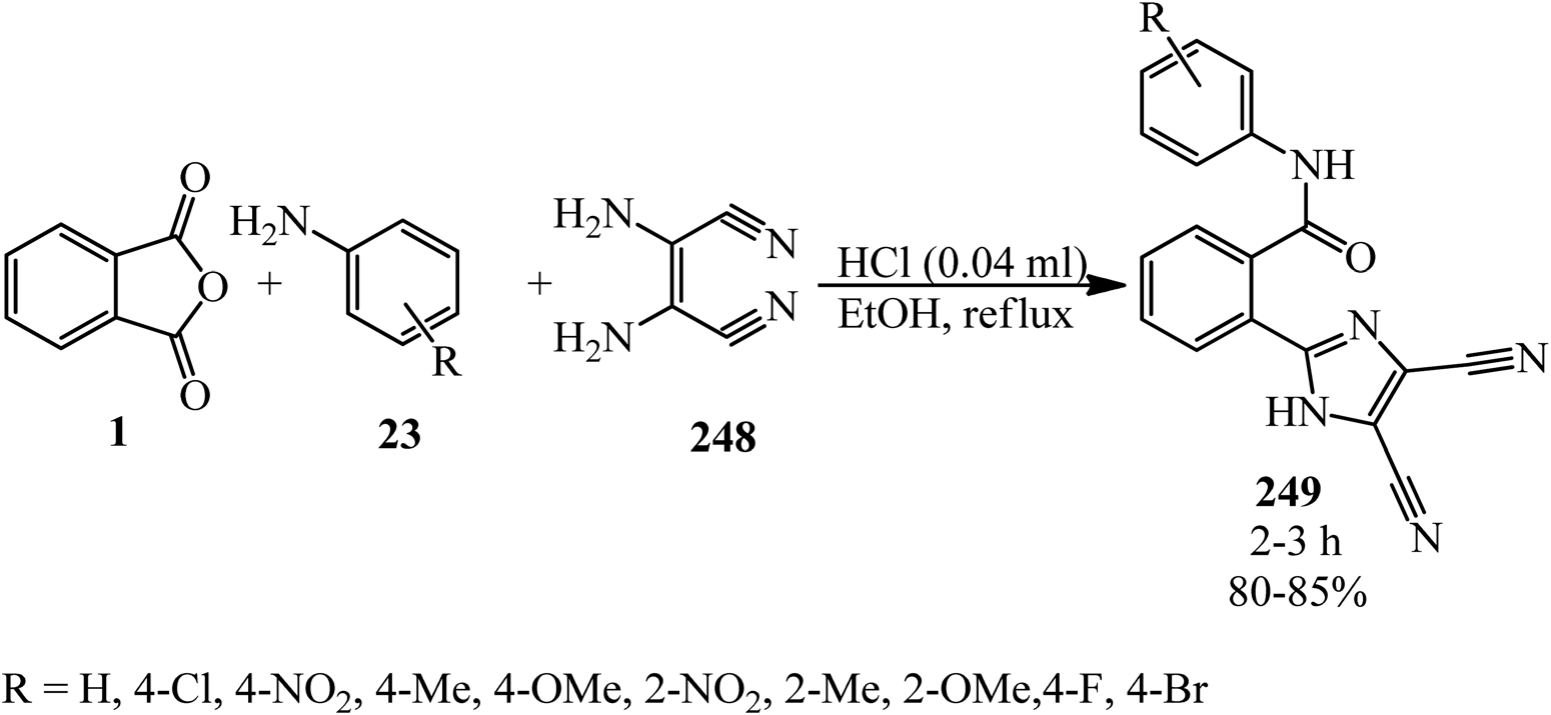
Synthesis of imidazole-based *N*-phenylbenzamides.

Malik's group in 2022 accessed new imidazole-based *N*-phenylbenzamides (249) from one-pot three-component reaction PA, substituted anilines (23), and 2,3-diaminomaleonitrile (248) in the presence of HCl in refluxing ethanol. The cytotoxic evaluation revealed that some derivatives (with para-fluorine and *para*-methoxy substituent) were the most active compounds, which exhibited good activity against the tested cancer cell lines (with single-digit IC_50_ values). Computational studies and molecular dynamic simulations were also investigated (Scheme 92).^210^

**Scheme 93:**
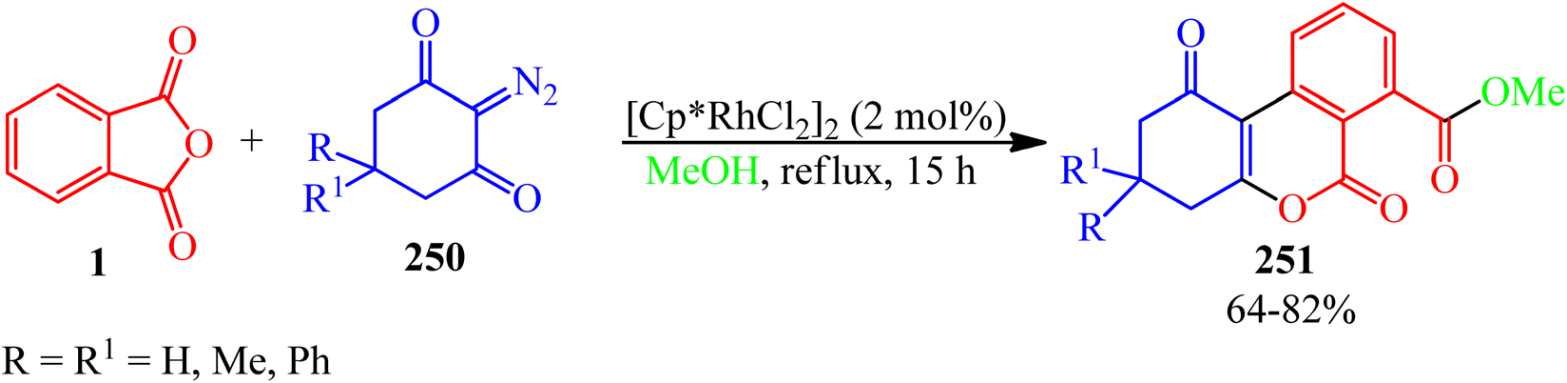
Preparation of cyclohexenone-fused isocoumarins.

Xinwei's group in 2022 developed a rhodium(iii)-catalyzed cascade reaction of phthalic anhydrides with cyclic 2-diazo-1,3-diketones (250) and methanol to obtain the esterified cyclohexenone-fused isocoumarins (251). The formation of C–C and two C–O bonds through C–H activation, transannulative coupling, and subsequent annulation process occurred through this strategy. Furthermore, there is no need for an additive or base. Surprisingly, the reaction proceeded well under air atmosphere with broad substrate and good functional group tolerance (Scheme 93).^211^

## 4. Applications of PA in four-component and pseudo four-component reactions

**Scheme 94:**
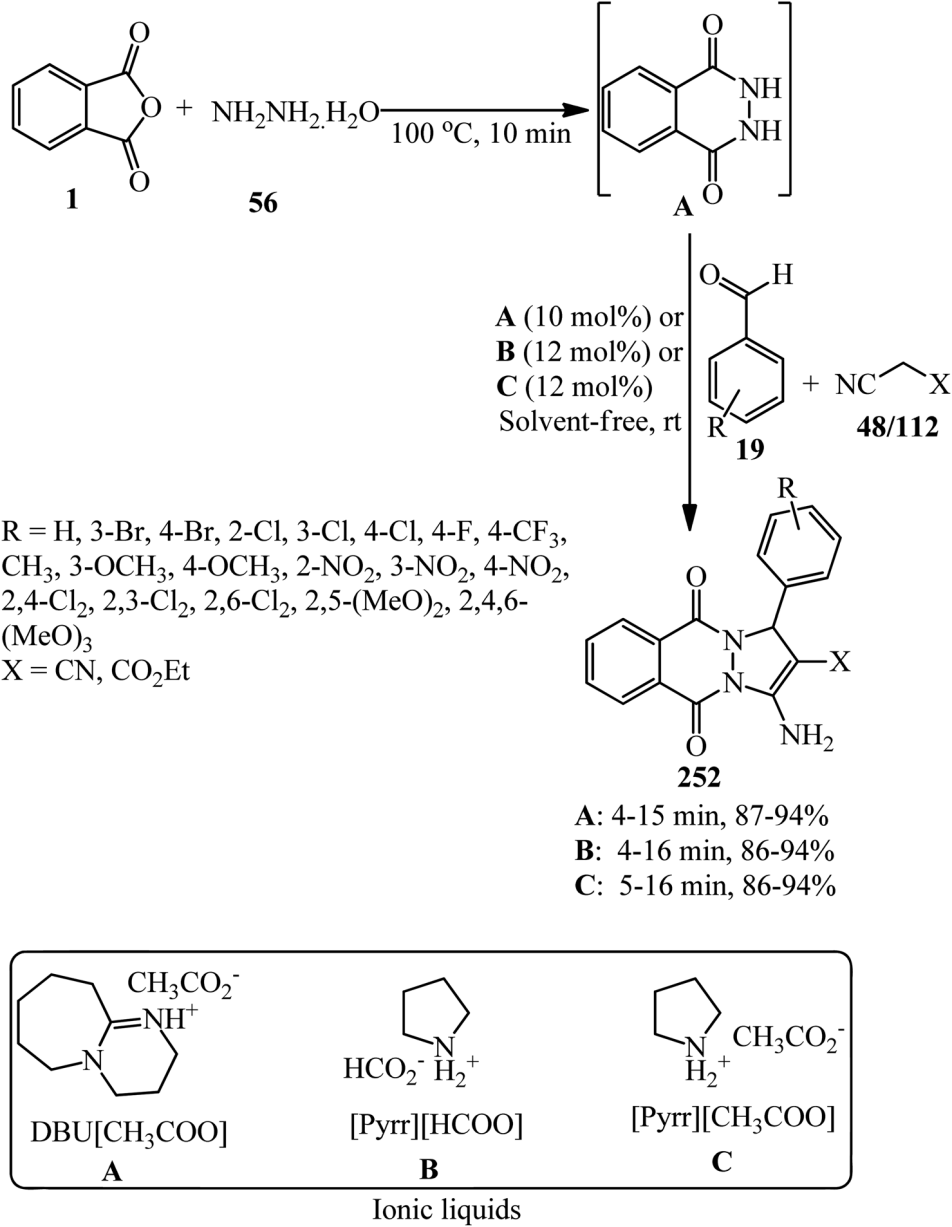
Synthesis of 1*H*-pyrazolo[1,2-*b*]phthalazine-5,10-diones.

Shaterian and Mohammadnia in 2012 prepared 1*H*-pyrazolo[1,2-*b*]phthalazine-5,10-diones (252) under ambient and solvent-free conditions in the presence of mild basic ionic liquids, which are 1,8-diazabicyclo[5.4.0]-undec-7-en-8-ium acetate (DBU[CH_3_COO]), pyrrolidinium formate ([Pyrr][HCOO]), and pyrrolidinium acetate ([Pyrr][CH_3_COO]) *via* the domino reaction of PA (1), aromatic aldehydes (19), malononitrile (48)/ethyl cyanoacetate (112), and hydrazine monohydrate (56) (Scheme 94).^212^ The PA and (56) were mixed at 100 °C for 10 min to obtain solid phthalhydrazide intermediate (A). The subsequent addition of (48)/(112) and aromatic aldehydes (19) in addition with 2appropriate ionic liquid at ambient temperature yielded the products (252).

In 2014, the same group also obtained 1*H*-pyrazolo[1,2-*b*]phthalazine-5,10-diones (252) *via* the four-component domino one-pot condensation reaction of PA (1), aromatic aldehydes (19), malononitrile(48)/ethyl cyanoacetate (112), and hydrazine monohydrate (56) in the presence of magnetic Fe_3_O_4_ nanoparticles coated with (3-aminopropyl)-triethoxysilane (APTES-MNP, 10 mol%) as the catalyst under solvent-free conditions within 4–15 min with 94–86% yield.^213^

Many other research groups studied the domino four-component reaction of PA (1), aromatic aldehydes (19), malononitrile(48)/ethyl cyanoacetate (112), and hydrazine monohydrate (56) to prepare the corresponding 1*H*-pyrazolo[1,2-*b*]phthalazine-5,10-diones (252) in the presence of various catalytic systems successfully. For example, Ghomi *et al.* in 2014 utilized CuI nanoparticles (10 mol%) within 25–30 min by 82–93% to accelerate this transformation.^214^

Some other researches utilized PA (1), aromatic aldehydes (19), malononitrile (48), and hydrazine monohydrate (56) to prepare 1*H*-pyrazolo[1,2-*b*]phthalazine-5,10-diones (252). They are as follows: (A) Ghorbani-Vaghei *et al.* in 2016 exploited piperidinium benzene-1,3-disulfonate nanomagnetic ionic liquid (NMIL, 20 mg) as a novel and reusable catalyst at 110 °C under solvent-free conditions within 40–80 min with 75–94% yield. The magnetized nanostructure was recycled and reused seven times without any loss of catalytic activity. In addition, they examined the four-component reaction in the presence of aliphatic aldehydes such as 3-phenylpropanal and fused aromatic candidates such as 2-naphthadehyde successfully.^215^ (B) Lashkari's group in 2018 reported zinc acetate dihydrate (Zn(OAc)·2H_2_O, 15 mol%) to accelerate the synthesis of 1*H*-pyrazolo[1,2*b*]phthalazine-5,10-dione derivatives (252) under solvent-free conditions at 70 °C with 93–83% yield within 2.5–4.5 h.^216^ (C) Mohamadpour in 2020 discussed an ecosafe green synthetic route to obtain (252) *via* the domino Knoevenagel–Michael cyclocondensation of PA (1), hydrazine monohydrate (56), aromatic aldehydes (19), and malononitrile (48) in the presence of carboxymethyl cellulose (CMC, 25 mol%) at 80 °C with a yield of 77–94% in 65–95 min period. Then, the catalyst was recovered after washing with ethyl acetate, filtering, and air drying, then reused for consecutive five runs with good yields and with insignificant CMC loss.^217^ (D) Maghsoodlou and coworkers in 2016 achieved (252) *via* the domino reaction of PA (1), hydrazine monohydrate (56), aromatic benzaldehyde (19), and malononitrile (48) (1 : 1 : 1 : 1 molar ratio) with copper(ii) acetate monohydrate (20 mol%) at 80 °C within 3–5 min with 73–89%.^218^ (E) Amini's group in 2021 prepared (251) through a one-step reaction of PA (1), aromatic aldehydes (19), malononitrile (48), and hydrazine monohydrate (56) (in a 1 : 1 : 1 : 1.2 molar ratio) in the presence of NiCl_2_·6H_2_O (10 mol%) in refluxing EtOH for 2–4 h within 81–96%. They also examined the procedure with ethanal (as an aliphatic candidate) to prepare the corresponding products successfully. The authors executed the α-glucosidase inhibitory activity of the synthesized compounds using a source of the α-glucosidase enzyme (EC3.2.1.20, *Saccharomyces cerevisiae* at 20 U mg^−1^ concentration). The results revealed that some of the products are efficient inhibitors of the α-glucosidase enzyme compared to the acarbose standard.^219^ (F) Lingampalle's group in 2019 constructed (252) though the domino reaction, in which, firstly, equal molar ratio of PA (1) and monohydrate hydrazine (56) were mixed at 80 °C for 15 min. Then, aromatic aldehydes (19) and malononitrile (48) were added and heated at 100 °C under microwave irradiation (300 W) for 10–16 min in the presence of boric acid (10 mol%) catalyst, which led to the corresponding adducts (252) with 86–94% yield.^220^ (G) 3-Amino-1-aryl-5,10-dioxo-1*H*-pyrazolo[1,2-*b*]phthalazine-2-carbonitriles (252) were also obtained by Abdesheikhi and Karimi-Jaberi in 2015 though the domino reaction of PA, hydrazine hydrate, benzaldehydes, and malononitrile in the presence of K_2_CO_3_ (0.1 g) in refluxing ethanol within 50–80 min by 86–96%.^221^ (H) Kalhor's group in 2022 obtained novel multifunctional nanocatalyst (Mn/4-MePy-IL@ZY), namely, 4-methylpyridinium chloride ionic liquid grafted on Mn@zeolite-Y, and scanned its catalytic performance (10 mol%) in the four-component synthesis of pyrazolo[1,2-*b*]phthalazines (252) under mild reaction conditions (H_2_O/EtOH, 1 : 1, 80 °C) within 8–12 min with 88–98% yields.^222^

Jonnalagadda *et al.* in 2020 utilized eggshell powder (ESP, 20 mg) as a biodegradable and recyclable catalyst for the synthesis of pyrazolo-phthalazine derivatives (252) in aqueous media within 28–45 min by 93–98% at 60 °C. They performed the reaction in the presence of the substrates PA, hydrazine hydrate, aldehydes, and activated methylene groups in a 1 : 1 : 1 : 1 molar ratio in a one-pot and one-step manner. They examined the method scope with different activated methylene compounds (malononitrile, ethyl cyanoacetate, and methyl cyanoacetate) and also several benzaldehydes and indole-3-carbaldehdye successfully.^223^

Roy's group in 2016 synthesized (252) through the domino reaction of PA (1), aromatic aldehyde (19), ethyl cyanoacetate (112), and hydrazine hydrate (56) in a 1 : 1 : 1 : 1.2 molar ratio in the presence of l-proline (10 mol%) and LiCl (5 mol%) in ethanol/water (1 : 1) media at 80 °C within 10–12 h by 87–90%. LiCl was used to elevate the yield of the reaction by elevating the electrophilicity of the aldehyde functionality.^224^

**Scheme 95:**
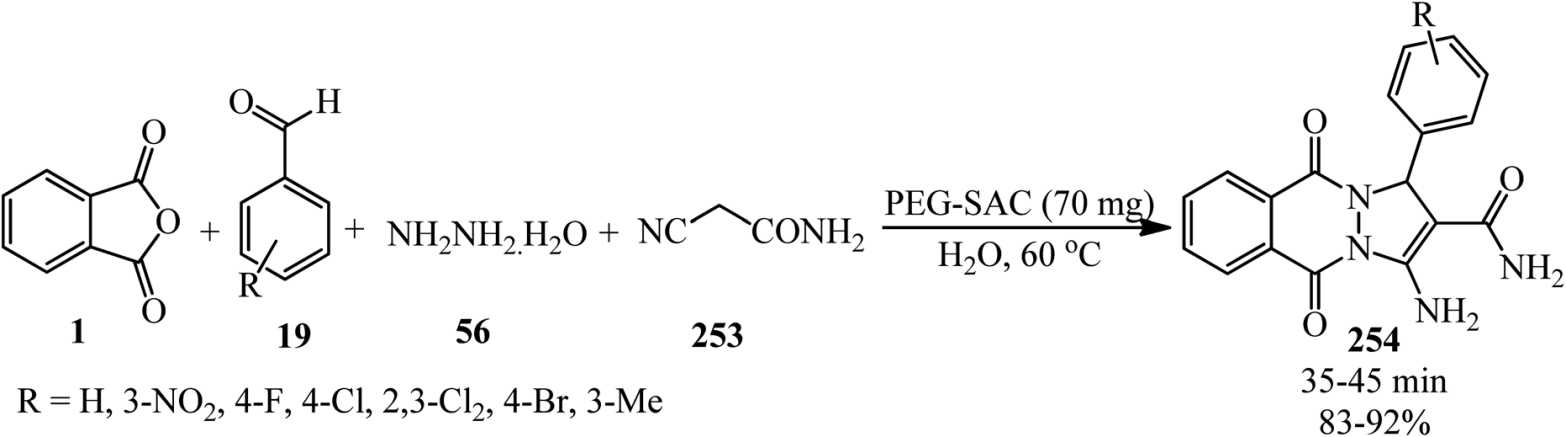
Preparation of multifunctionalized 1*H*-pyrazolo[1,2-*b*]phthalazine-5,10-dione carboxamides.

The Das group in 2014 prepared a novel biodegradable SO_3_H-bearing carbonaceous solid catalyst (PEG-SAC, 70 mg) to achieve 1*H*-pyrazolo[1,2-*b*]phthalazine-5,10-dione carboxamides (254) through a domino aqueous media reaction of PA (1), hydrazine hydrate (56), (hetero)aromatic aldehydes (19), and malononitrile (48)/2-cyanoacetamide (253) at 60 °C. They also utilized aliphatic aldehydes such as isobutyraldehyde successfully. To improve the applicability of the Brønsted acid PEG-SAC catalyst, the authors also examined the preparation of multifunctionalized 1*H*-pyrazolo[1,2-*b*]phthalazine-5,10-dione carboxamides in a regio-controlled manner (no 1*H*-pyrazolo[1,2-*b*]phthalazine-2-carbonitriles observed) (Scheme 95).^225^

**Scheme 96:**
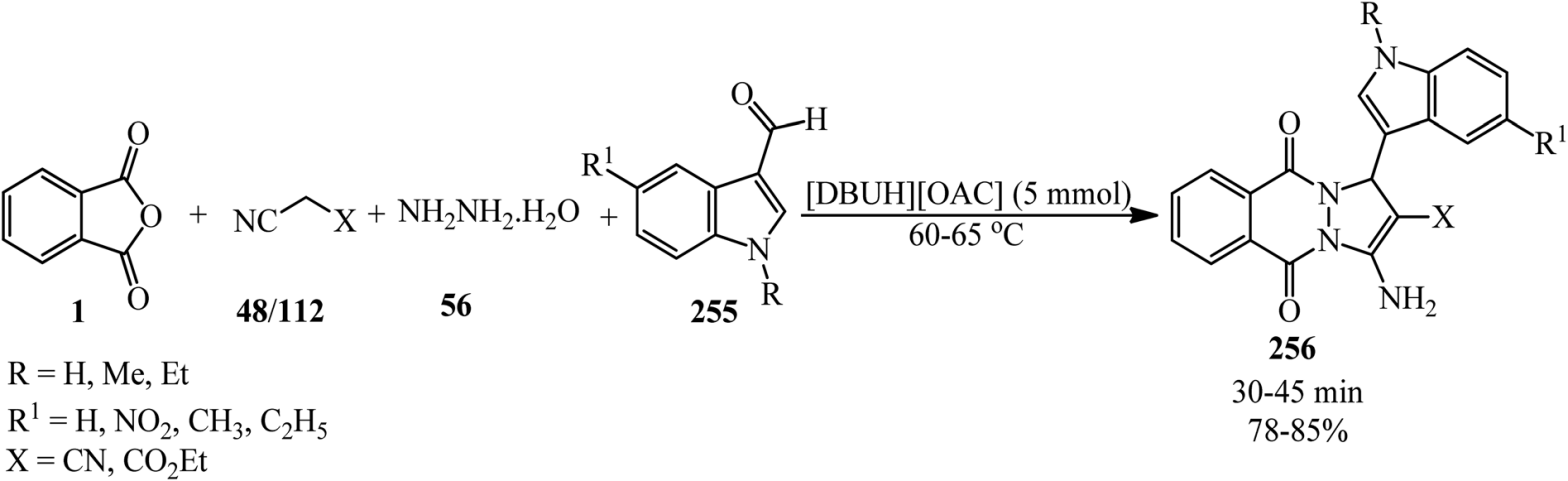
Synthesis of 3-amino-1-(1*H*-indol-2-yl)-5,10-dioxo-5,10-dihydro-1*H*-pyrazolo[1,2-*b*]phthalazines.

Raghavendra Siddaiah performed the domino synthesis of 3-amino-1-(1*H*-indol-2-yl)-5,10-dioxo-5,10-dihydro-1*H*-pyrazolo[1,2-*b*]phthalazine derivatives (256) *via* the reaction of PA (1) with hydrazine hydrate (56), malononitrile (48)/ethyl cyanoacetate (112), and indole-3-carboxaldehydes (255) in the presence of the ionic liquid ([DBUH][OAc]) at 60–65 °C (Scheme 96).^226^

Reddy's group in 2014 also obtained 3-amino-1-(1*H*-indol-2-yl)-5,10-dioxo-5,10-dihydro-1*H*-pyrazolo[1,2-*b*]phthalazines (256) in a four-component domino reaction between hydrazine hydrate, *N*-substituted-indole-3-carboxaldehydes, malononitrile/ethyl cyanoacetate, and dialkylphthalates (methyl and ethyl) in the presence of InCl_3_ (30 mol%) in refluxing ethanol within 1 h with 70–85% yield.^227^ Also, they utilized dialkylphthalates instead of PA; interestingly, the products (256) were obtained.

**Scheme 97:**
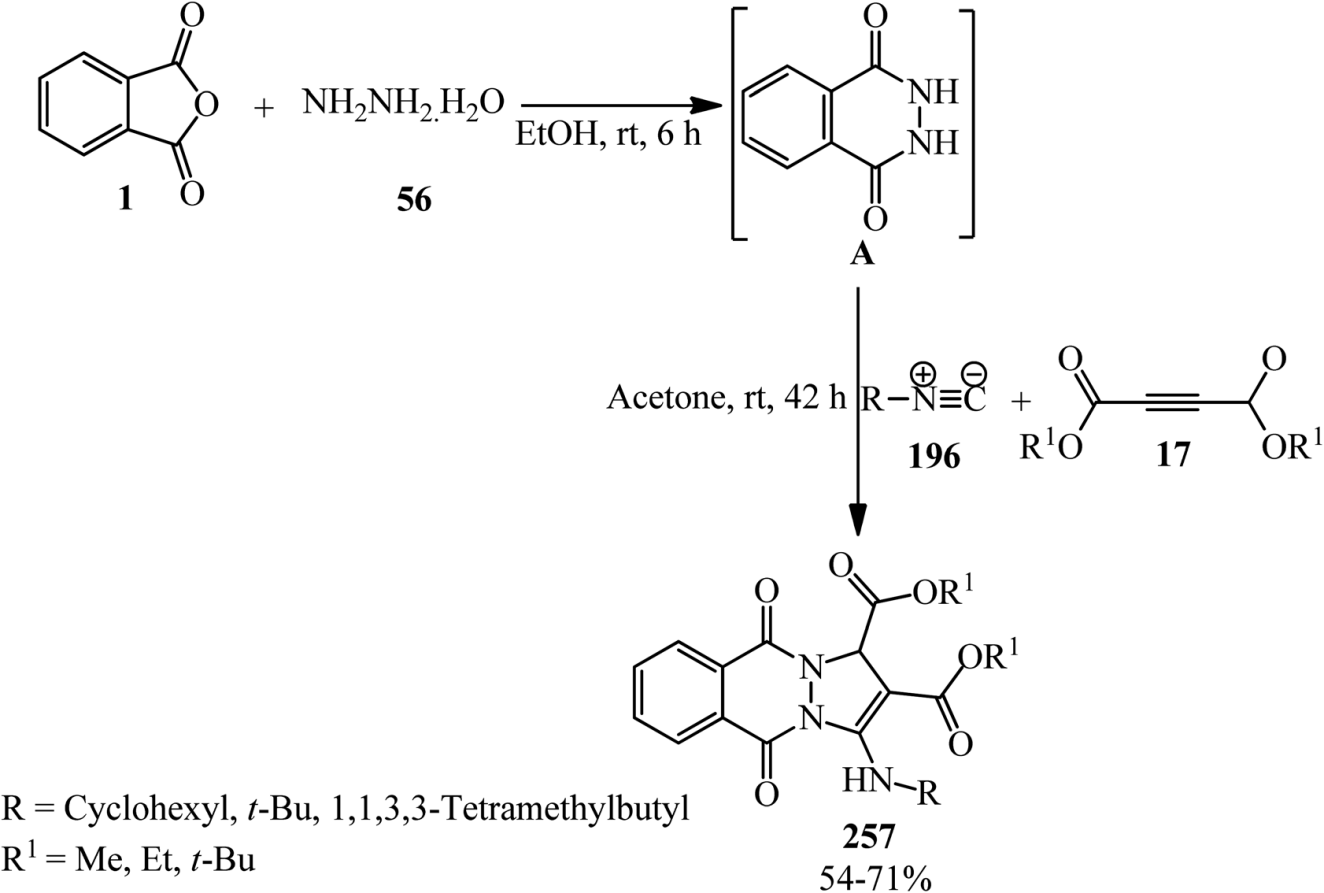
Synthesis of 1*H*-pyrazolo[1,2-*b*]phthalazine-1,2-dicarboxylates.

Shaabani's research group in 2012 developed the synthesis of structurally diverse 1*H*-pyrazolo[1,2-*b*]phthalazine-1,2-dicarboxylates (257) *via* a four-component reaction of PA (1), hydrazine hydrate (56), dialkyl acetylenedicarboxylates (17), and isocyanides (196) in ethanol/acetone (1 : 1) at room temperature in good to moderate yields (Scheme 97).^228^

**Scheme 98:**
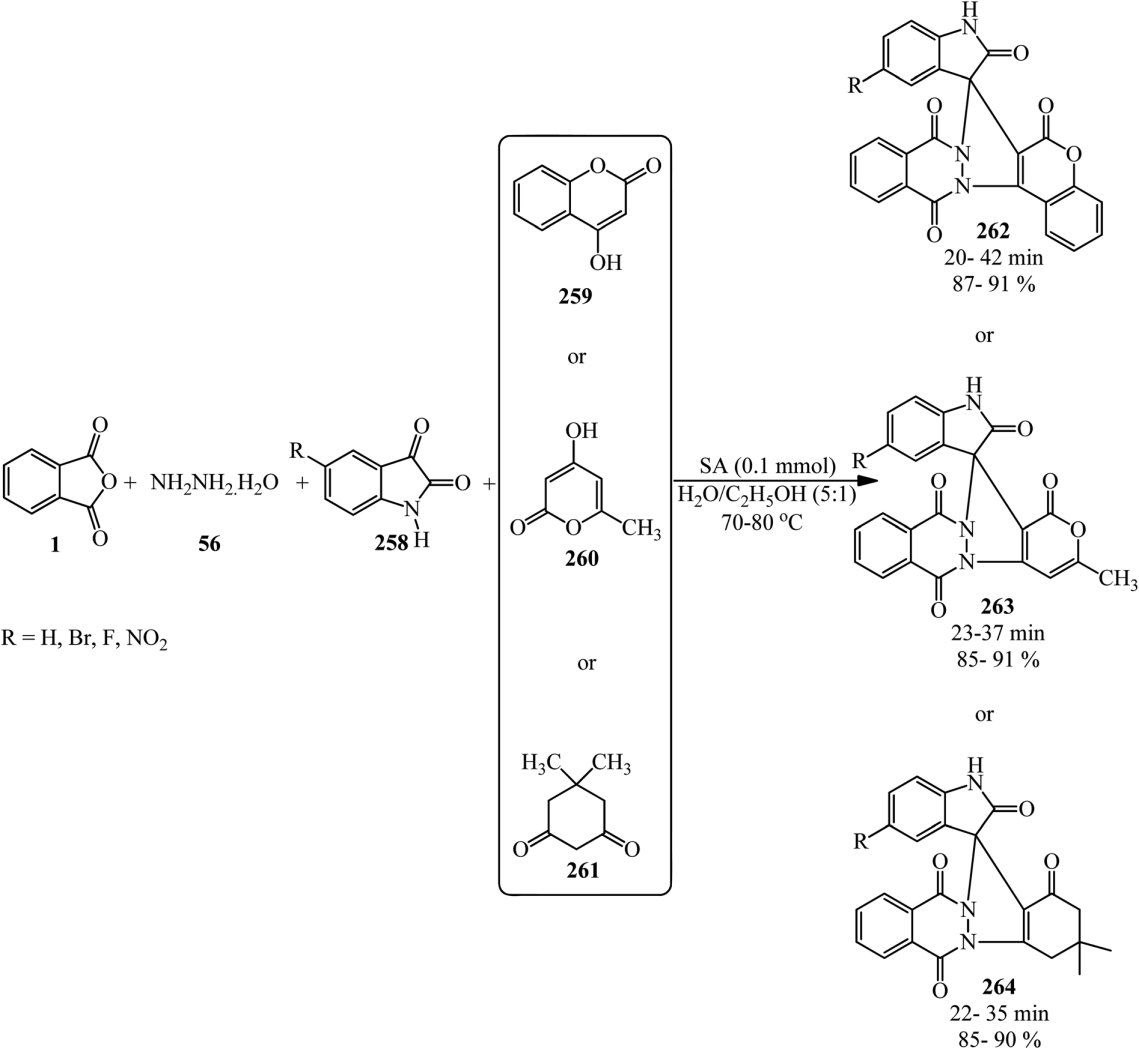
Synthesis of spirooxindoles spiroannulated with chromenopyrazolophthalazines/pyranopyrazolophthalazines/indazolophthalazine.

Kumar's research group in 2013 presented one-pot four-component protocol for the synthesis of structurally diverse spirooxindoles, spiroannulated with chromenopyrazolophthalazines/pyranopyrazolophthalazines/indazolophthalazine (262, 263, 264) from the reaction of PA (1), hydrazine hydrate (56), isatins (258), and cyclic ketones [4-hydroxy-2*H*-chromen-2-one (259), 4-hydroxy-6-methylpyran-2-one (260), dimedone (261)] in aqueous alcoholic medium (H_2_O/C_2_H_5_OH, 5 : 1) using catalytic amounts of sulphamic acid (SA) (Scheme 98).^229^ The reaction mechanism is considered to involve acid-catalyzed Knoevenagel condensation, Michael-type addition reaction, and consequent intramolecular dehydrative cyclization to obtain the pure products *via* recrystallization from ethanol.

**Scheme 99:**
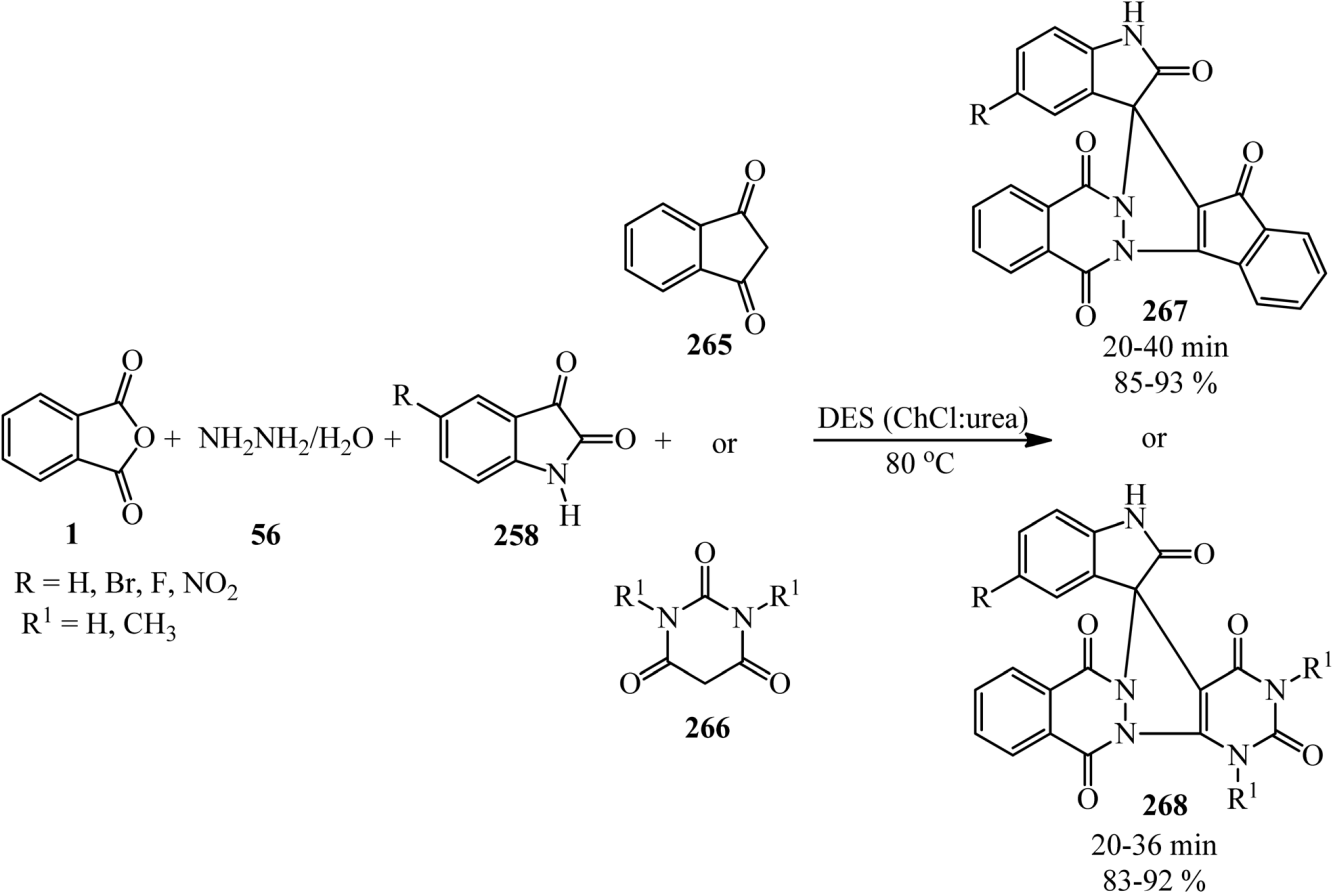
Construction of indenopyrazolophthalazines and pyrazolopyrimidophthalazines.

Kumar's group in 2014 constructed indenopyrazolophthalazines (266) and pyrazolopyrimidophthalazines (267, 268) *via* the reaction of PA (1), hydrazine hydrate (56), isatins (258), and 1,3-indandione (265)/barbituric acids (266) in the presence of deep eutectic solvent (DES, choline chloride : urea, 1 : 2) as a catalyst and reaction medium at 80 °C (Scheme 99).^230^ They also prepared chromenopyrazolophthalazines (262) and indazolophthalazine (264) in the same reaction conditions within 20–32 min with 88–90% and 86–93% yields, respectively.

Biabangard and Shaterian in 2015 obtained pyrazolopyrimidophthalazines (267) *via* the domino four-component reaction of PA (1), aromatic aldehydes (19), hydrazine hydrate (56), and barbituric acid (266), in a 1 : 1 : 1.2 : 1 molar ratio, using vitamin B1 supported on alumina (VB_1_-Al_2_O_3_, 5 mol%) as a heterogeneous catalyst under solvent-free conditions at 70 °C within 10–14 min with 88–92% yield.^231^ The recovered catalyst was reutilized for at least four runs without any loss of its activity.

**Scheme 100:**
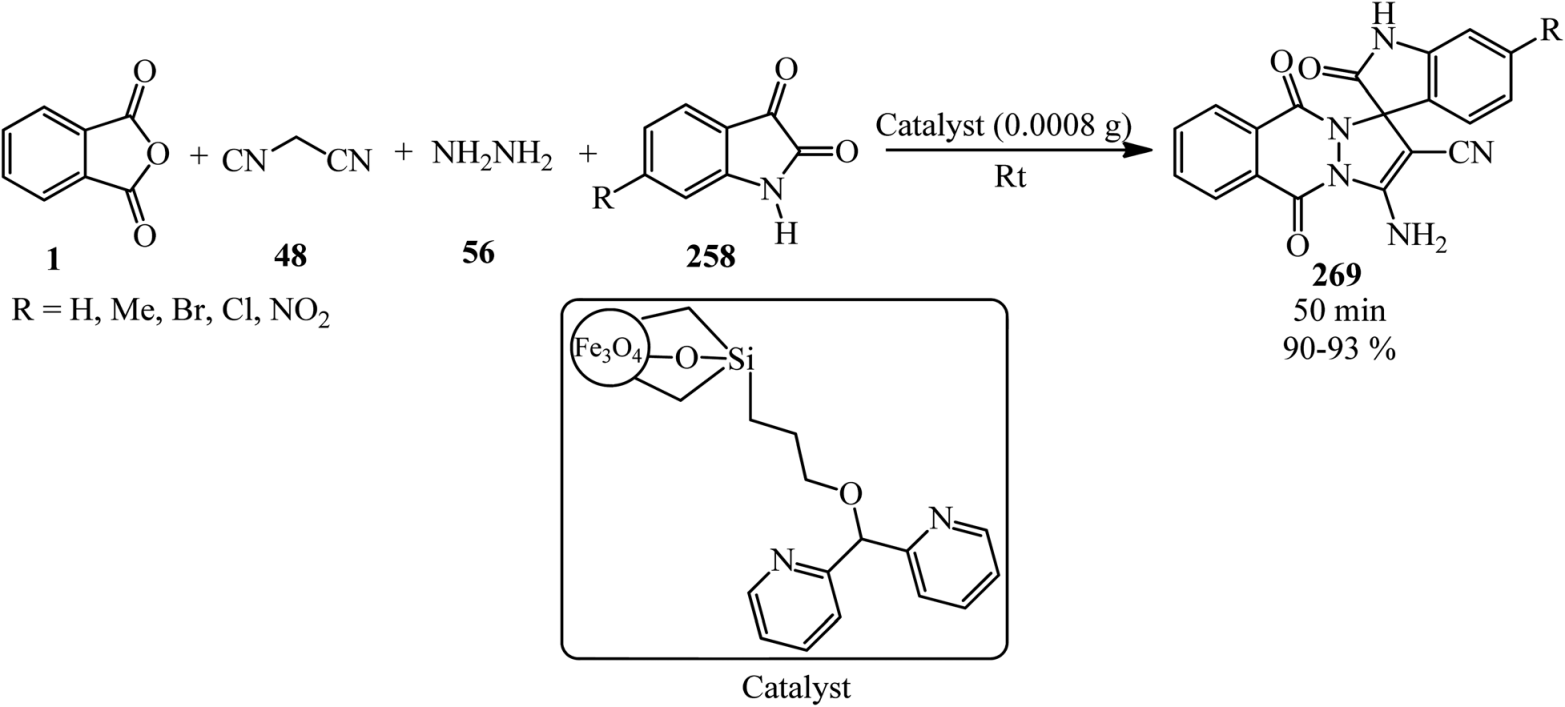
Preparation of pyrazolophthalazinyl spirooxindoles.

A novel catalyst obtained *via* the stabilization of methylene dipyridine nanoparticles on Fe_3_O_4_ (Fe_3_O_4_/SiO_2_/propyltriethoxysilane/methylene dipyridine nanoparticles) was prepared by Sadeghzadeh and Nasseri in 2013, which was utilized to prepare pyrazolophthalazinyl spirooxindoles (269) *via* a four-component solvent-free reaction at room temperature (Scheme 100).^232^

**Scheme 101:**
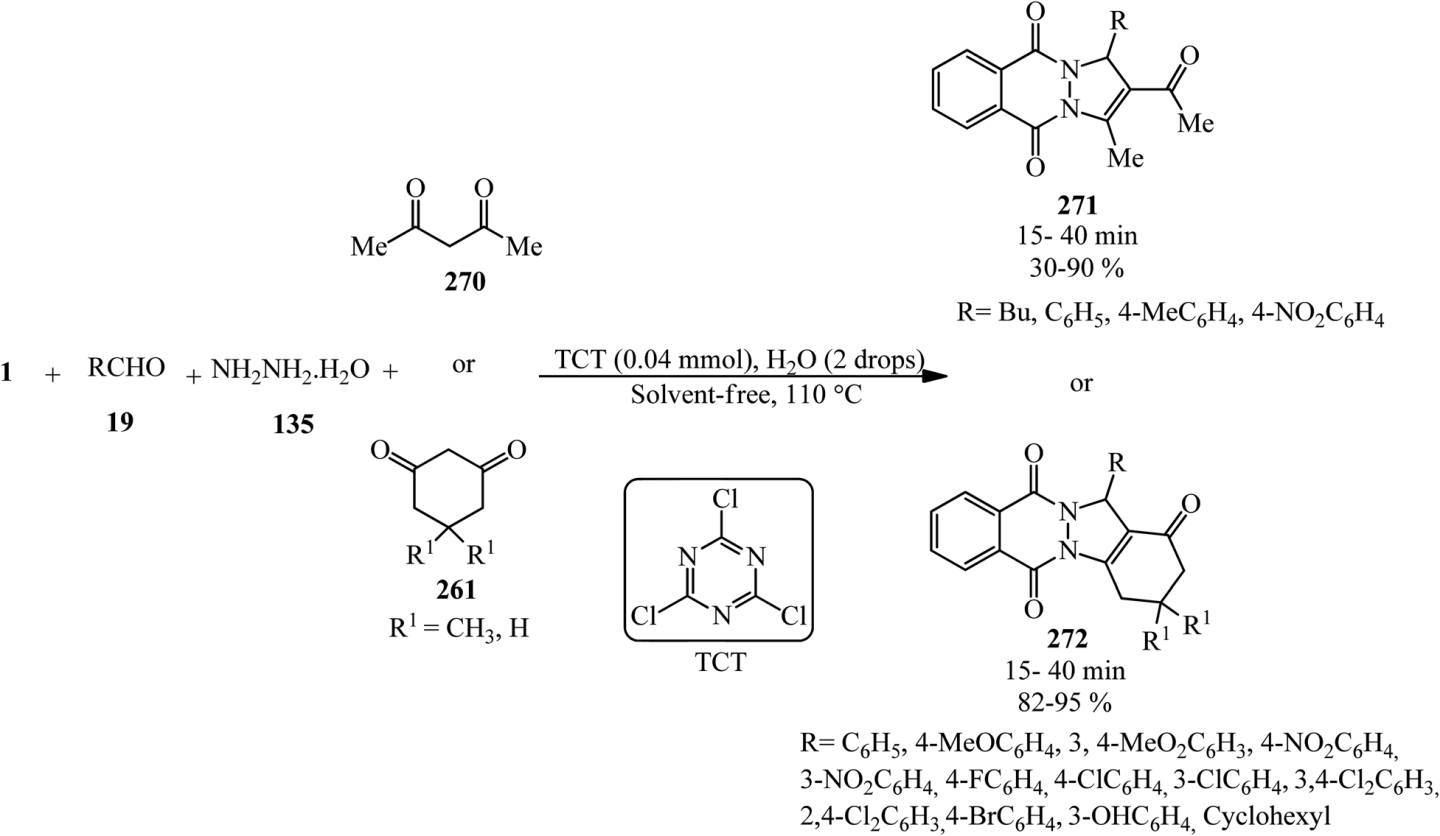
Synthesis of 1*H*-pyrazolo[1,2-*b*]phthalazine-5,10-diones and 1*H*-indazolo[1,2-*b*] phthalazine-1,6,11-triones.

Maleki and Sedigh Ashrafi in 2014 reported the synthesis of 1*H*-pyrazolo[1,2-*b*]phthalazine-5,10-diones (271) and 1*H*-indazolo[1,2-*b*] phthalazine-1,6,11-triones (272) *via* the multicomponent and one-pot reactions of PA (1), various aldehydes (19), hydrazinium hydroxide (56), and acyclic or cyclic 1,3-diketones (261, 270) using wet 2,4,6-trichlorotriazine (TCT) as a catalyst under solvent-free conditions (Scheme 101).^233^ Cyanuric acid was produced as the byproduct, which was removed by washing with water.

**Scheme 102:**
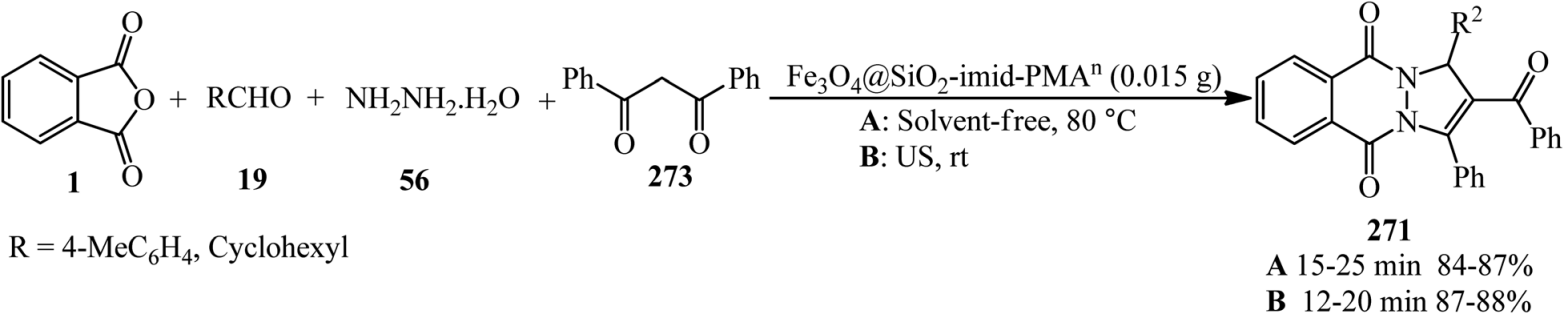
Pyrazolo[1,2-*b*]phthalazine-5,10-diones obtained from PA, aldehydes, hydrazine hydrate, and 1,3-diphenylpropanedione.

Esmaeilpour and Zahmatkesh *et al.* in 2017 investigated the synthesis of 1*H*-pyrazolo[1,2-*b*]phthalazine-diones (271) 2*H*-indazolo[2,1-*b*]phthalazine-triones (272) using Fe_3_O_4_@SiO_2_-imid-PMA^*n*^ nanoparticles (immobilization of H_3_PMo_12_O_40_ nanoparticles (PMA^*n*^) on an imidazole-functionalized Fe_3_O_4_@SiO_2_) as an ecofriendly magnetic catalyst from the four-component condensation, solvent-free reaction of PA (1), aldehydes (19), hydrazinium hydroxide (56), 1,3-diketones (dimedone (261), 1,3-pentandion), and in 1 : 1 : 1.2 : 1 molar ratio under conventional thermal conditions (80 °C) or ultrasound-assisted conditions at room temperature.^234^ Also, to enlarge the efficacy of the procedure, a vast range of aldehydes including aromatic, heteroaromatic, cyclic/acyclic aliphatic, and sterically-hindered candidates were used to perform the reaction successfully. They also examined the catalyst efficacy to prepare some new kinds of pyrazolo[1,2-*b*]phthalazine-5,10-diones (271) through the reaction of PA (1), aldehydes (19), hydrazine hydrate (56), and 1,3-diphenylpropanedione (273) (Scheme 102).

**Scheme 103:**
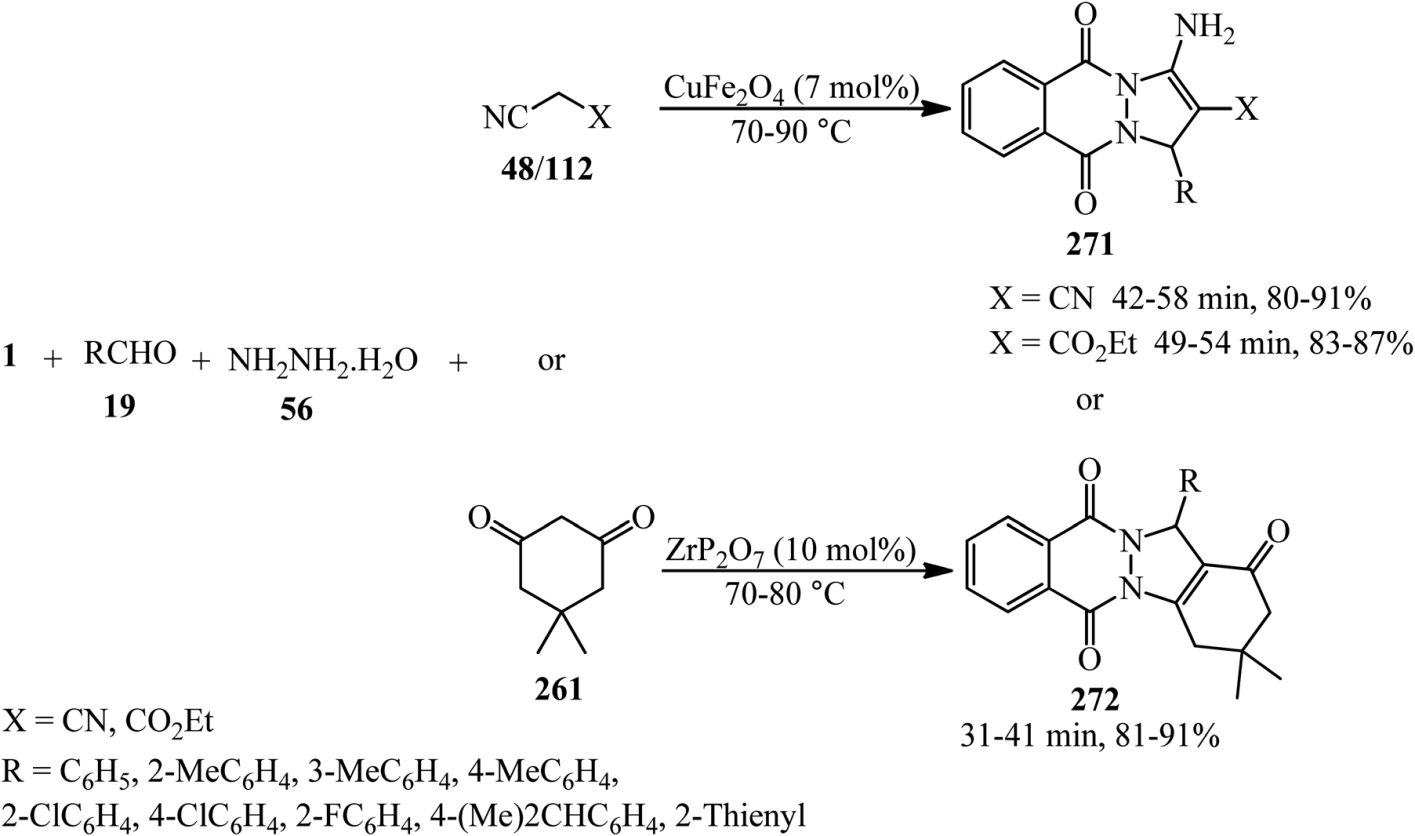
Synthesis of 1*H*-pyrazolo[1,2-*b*]phthalazine-diones and 2*H*-indazolo[2,1-*b*]phthalazine-triones.

Safaei-Ghomi *et al.* in 2016 described the synthesis of some 1*H*-pyrazolo[1,2-*b*]phthalazine-diones (271) and 2*H*-indazolo[2,1-*b*]phthalazine-triones (272) *via* the domino reaction of PA (1), aromatic aldehydes (19), hydrazine monohydrate (56), and malononitrile (48)/ethyl cyanoacetate (112)/dimedone (261), respectively. The reaction proceeded in the presence of CuFe_2_O_4_ and ZrP_2_O_7_ nanocatalysts under solvent-free conditions (Scheme 103).^235^

**Scheme 104:**
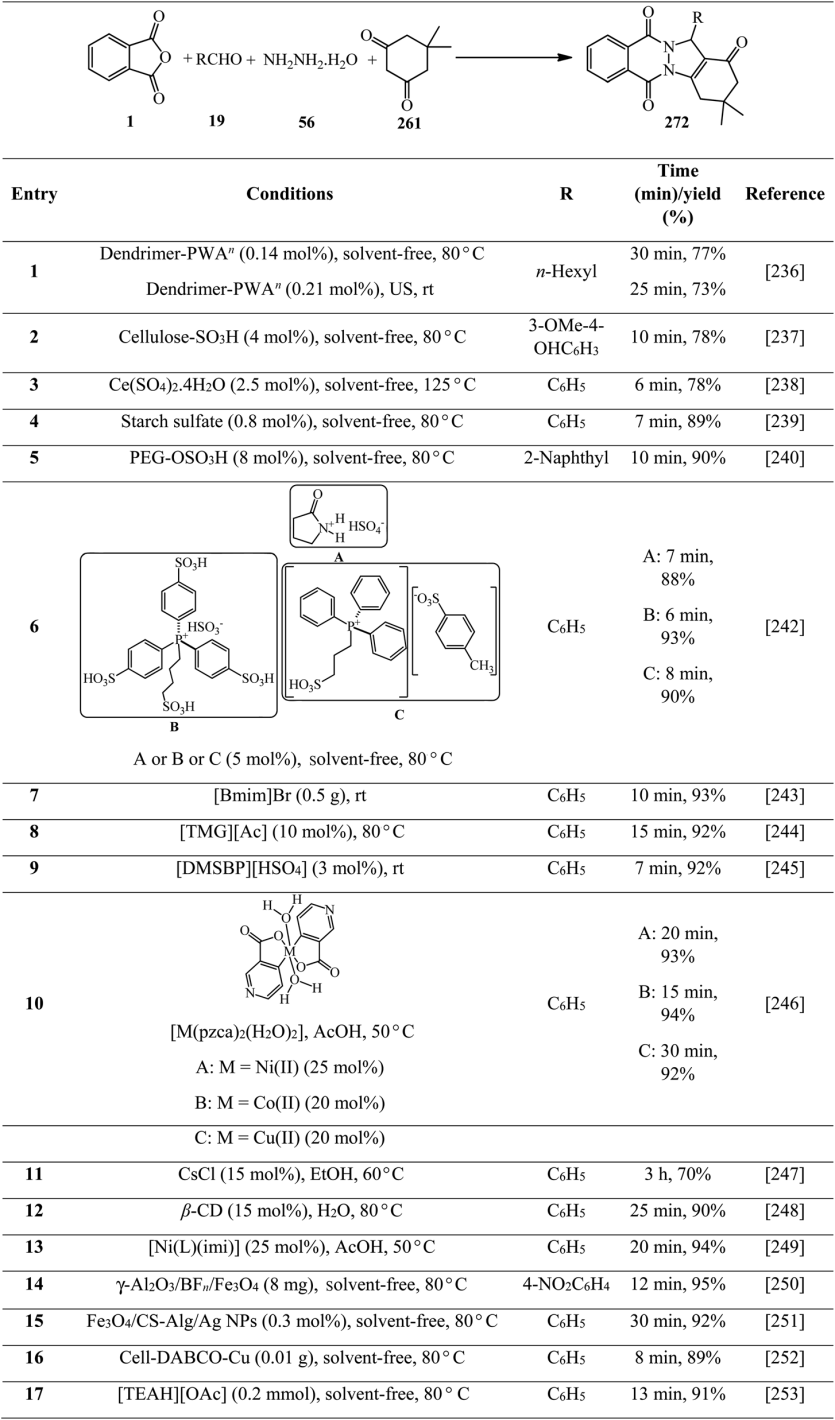
Investigation of the scope of the protocols to obtain 2*H*-indazolo[2,1-*b*]phthalazine-triones.

Besides the abovementioned examples, many other research groups prepared different kinds of 2*H*-indazolo[2,1-*b*]phthalazine-triones (272) *via* a four-component reaction of PA (1), different aldehydes (19), hydrazine hydrate (56), and dimedone (261). (A) The Javidi and Esmaeilpour group in 2016 synthesized dendrimer-encapsulated phosphotungstic acid nanoparticles immobilized on nanosilica with surface amino groups (dendrimer-PWA^*n*^) and utilized it as a reusable catalyst to accelerate the green synthesis of (272) *via* a one-pot, one-step, and four-component condensation reaction of PA (1), aldehydes (19), hydrazinium hydroxide (56), and dimedone (261) in a 1.2 : 1 : 1 : 1 molar ratio at both under solvent-free conditions at 80 °C and ultrasound irradiation at room temperature.^236^ The protocol was successful for different classes of aldehydes such as aromatic, cyclic/linear aliphatic, heteroaromatics, and sterically-hindered candidates. An example of their procedure has been demonstrated in Scheme 104 (entry 1). In addition, the catalyst could be successfully recycled and reused at least for six runs without a significant loss in activity. (B) Shaterian and Rigi in 2014 prepared (272) using cellulose-SO_3_H (4 mol%) as a solid-acidic reusable catalyst *via* the domino four-component condensation reaction under thermal (80 °C) solvent-free conditions (Scheme 104, entry 2).^237^ The protocol was unsuccessful in utilizing aliphatic aldehydes such as heptanal and octanal. (C) Mosaddegh and Hassankhani in 2011 described an efficient protocol for the one-pot, one-step synthesis of 2*H*-indazolo[2,1-*b*]phthalazine-1,6,11(13*H*)-triones (272) under thermal (125 °C) solvent-free conditions using PA (1), aromatic aldehydes (19), hydrazinium hydroxide (56), and dimedone (261) in a 1 : 1 : 1.2 : 1 molar ratio in the presence of Ce(SO_4_)_2_·4H_2_O (2.5 mol%) within 5–10 min (Scheme 104, entry 3).^238^ (D) Shaterian and Rigi in 2011 obtained (272) *via* the domino four-component condensation reaction from (1), aromatic aldehydes (19), (56), and dimedone (261) in a 1 : 1 : 1.2 : 1 molar ratio using 0.8 mol% starch sulfate as a bio-supported nonhygroscopic solid-acid catalyst under thermal (80 °C) solvent-free conditions within 4–15 min period with 72–92% yield (Scheme 104, entry 4).^239^ The recovered catalyst was reused four times without any loss of its activities. (E) The compounds (272) were achieved by Hasaninejed *et al.* in 2012 through the four-component one-step condensation reaction of PA (1), aromatic aldehydes (19), hydrazinium hydroxide (56), and 1,3-cyclohexanedione/dimedone in a 1 : 1 : 1.12 : 1 molar ratio at 80 °C using sulfuric acid-modified PEG-6000 (PEG-OSO_3_H, 8 mol%) as a green, recyclable, and biodegradable polymeric catalyst, with the yield of 80–93% in 10–20 min (Scheme 104, entry 5).^240^ The authors calculated the environmental impact factor (E-factor)^241^ to evaluate the minimizing environmental impacts of their method. (F) Shaterian and Aghakhanizadeh in 2012 obtained (272) from the domino four-component solvent-free reaction of PA (1), arylaldehydes (19), hydrazine monohydrate (56), and dimedone/1,3-cyclohexanedion in a 1.2 : 1 : 1.4 : 1 molar ratio using reusable ionic liquids [including A: 2-pyrrolidonium hydrogen sulfate ([Hnhp][HSO_4_]); B: (4-sulfobutyl)tris(4-sulfophenyl)phosphonium hydrogen sulfate; and C: triphenyl(propyl-3-sulphonyl)phosphonium toluenesulfonate] as acidic reusable catalysts (5 mol%). The catalysts recovered within 5 runs without significant activity loss. The time and yield of utilizing these 3 ILs at 80 °C are A: 8–11 min/70–90%; B: 4–8 min/83–92%; and C: 6–15 min/80–93%, respectively (Scheme 104, entry 6).^242^ (G) Shekouhy and Hasaninejad in 2012 obtained (272) *via* the one-pot and one-step four-component reaction of PA (1), (hetero)aromatic aldehydes (19), hydrazine monohydrate (56), and dimedone (261) in a 1 : 1 : 1.2 : 1 molar ratio with a yield of 89–95% in 3–15 min using 1-butyl-3-methyl imidazolium bromide ([Bmim]Br, 0.5 g) as a neutral IL under ultrasonic irradiation and catalyst-free conditions at room temperature (Scheme 104, entry 7).^243^ (H) Veisi and Aminimanesh *et al.* in 2014 synthesized (272) in a one-pot and one-step reaction of PA (1), aliphatic/aromatic aldehydes (19), hydrazine monohydrate (56), and dimedone (261) in a 1 : 1 : 1.1 : 1 molar ratio in the presence of mildly basic ionic liquid *N*,*N*,*N*,*N*-tetramethylguanidinium acetate ([TMG][Ac], 10 mol%) in high yields (55–98%) at 80 °C within 10–30 min (Scheme 104, entry 8).^244^ (I) Habibi and Shamsian in 2015 utilized new reusable acid–base bifunctional ionic liquid, 1,4-dimethyl(4-sulfobutyl)piperazinium hydrogen sulfate ([DMSBP][HSO_4_], 3 mol%), for the synthesis of (272) *via* the one-step four-component reaction of reaction of PA (1), aromatic aldehydes (19), hydrazine monohydrate (56), and dimedone (261) in a 1 : 1 : 1.2 : 1 molar ratio with 85–94% yield in 3–15 min (Scheme 104, entry 9).^245^ Examining the reaction with octanal as an aliphatic aldehyde, it did not form the corresponding product. The catalyst was recovered from the aqueous medium, dried under vacuum, and reused in five successive runs without a substantial loss of activity. (J) The compounds (272) were also prepared by Ebrahimipour *et al.* in 2015 *via* the one-pot and one-step, and four-component reaction in the presence of three octahedral complexes containing 2-pyrazinecarboxylate (pzca), including [Ni(pzca)_2_(H_2_O)_2_] (25 mol%), [Co(pzca)_2_(H_2_O)_2_] (20 mol%), and [Cu(pzca)_2_(H_2_O)_2_] (20 mol%) in acetic acid solvent at 50 °C within 10–40 min with 80–94% yields (Scheme 104, entry 10).^246^ (K) Bhosale *et al.* in 2017 developed the synthesis of (272) in the presence cesium chloride (15 mol%) in refluxing ethanol at 60 °C *via* the equimolar amounts of the substrates in a one-step and one-pot manner, in 3–4 h duration with a yield of 70–82% (Scheme 104, entry 11).^247^ (L) Gill's research group in 2017 demonstrated a synthetic route for 2*H*-indazolo[2,1-*b*]pthalazinetrione derivatives (272) through a one-pot, one-step four-component protocol in the presence of β-cyclodextrin as a supramolecular, biodegradable, and reusable catalyst in 80 °C aqueous media within 94–79% yield during 35–25 min (Scheme 104, entry 12).^248^ (M) Ebrahimipour and coworkers in 2017 reported the synthesis of (272) with a yield of 84–94% in 15–25 min in the presence of imidazole 2-acetamido-*N*′-(3-methoxy-2-oxidobenzylidene)benzohydrazonate–nickel(ii) [Ni(L)(imi)], which is a tridentate Schiff base ligand (Scheme 104, entry 13).^249^ The Ni(ii) complex showed promising antimicrobial activities against some Gram-negative and Gram-positive bacteria such as *E. coli*, *S. aureus*, *P. aeruginosa*, and *B. cereus*. The catalyst was recovered by centrifugation and recrystallization from a mixture of ethanol and water and reused at least three times with satisfactory results. (N) Bamoniri *et al.* in 2020 described a method for the synthesis of 2*H*-Indazolo[2,1-*b*]phthalazinetrione derivatives (272) using nano γ-Al_2_O_3_/BF_*n*_/Fe_3_O_4_ (8 mg) *via* the one-step reaction of equimolar of four substrates under solvent-free conditions, which yielded the corresponding adducts with 90–97% yield in 10–17 min (Scheme 104, entry 14).^250^ The catalyst was recycled by washing with CH_2_Cl_2_, drying at 50 °C under vacuum for 1 h, and reused within 5 runs successfully. (O) Liu and Li *et al.* in 2020 prepared a novel modified core–shell magnetic nanocomposite by anchoring Ag NPs on magnetite core that was coated with chitosan-alginate dual bio-polysaccharide (Fe_3_O_4_/CS-Alg/Ag NPs). The novel nanocatalyst was utilized for the synthesis of (272) *via* one-step and one-pot reaction of the corresponding substrates at 80 °C within 0.2–1.5 h with 50–95% yield. The catalyst demonstrated human lung protective effects against α-Guttiferin. These events revealed that the catalyst suppressed Guttiferin-induced cell death in a dose-dependent manner in lung MRC-5, CCD-19Lu, WI-38, and BEAS-2B cell lines. The catalyst was recovered easily using an external magnet and recycled for 10 successive times with minimal reduction in activity (Scheme 104, entry 15).^251^ (P) In 2022, Naeimi and Zahedifar reported an immobilized copper(ii) complex on microcellulose (cell-DABCO-Cu) as a novel catalyst to promote the preparation of (272) at 80 °C under solvent-free conditions in a one-step and one-pot manner (Scheme 104, entry 16).^252^ In addition, the CuO nanoparticles with an average size of 40 nm were obtained by the direct calcination of Cell-DABCO-Cu. (Q) Mahmoodi *et al.* in 2020 obtained (272) *via* the one-pot, one-step, four-component, and solvent-free condensation of PA (1), aromatic aldehydes (19), hydrazine hydrate (56), and dimedone (261), which was accelerated with triethanolammonium acetate ([TEAH][OAc]) at 80 °C within 10–35 min with 89–95% yield (Scheme 104, entry 17).^253^ The authors examined the efficacy of the protocol through utilizing succinic anhydride instead of PA. They also prepared phthalazine-diones (271) by the reaction of PA (1), aromatic aldehydes (19), hydrazine hydrate (56), and alkyl cyanoacetates (112) (methyl and ethyl) successfully in the same reaction conditions within 22–37 min with 80–95%. (R) Tigote's group in 2017 used ZnFe_2_O_4_ nanoparticles (1.5 mol%) as a catalyst for this transformation *via* the one-pot and two-step reaction of aromatic aldehyde (19) and the dimedone (261), which was added into the mixture of PA (1) and hydrazine hydrate (56) in a 1 : 1 : 1 : 1.2 molar ratio at room temperature within 30–90 min with 76–89% yield.^254^ (S) Mahmoodi's group in 2020 used tetrabutyl phosphonium sulfate ([TBP]_2_SO_4_, 5 mol%) as a novel room-temperature ionic liquid (RTIL) to prepare (272) *via* the domino reaction of the mentioned four substrates at room temperature within 10–20 min with 89–92% yield. The authors examined the scope of the protocol utilizing succinic anhydride instead of PA. They also achieved phthalazine-diones through the reaction of phthalazine-diones (271) and the reaction of PA (1), aromatic aldehydes (19), hydrazine hydrate (56), and alkyl cyanoacetates (112) (methyl and ethyl) successfully in the same reaction conditions within 15–45 min with 65–92% yield.^255^

**Scheme 105:**
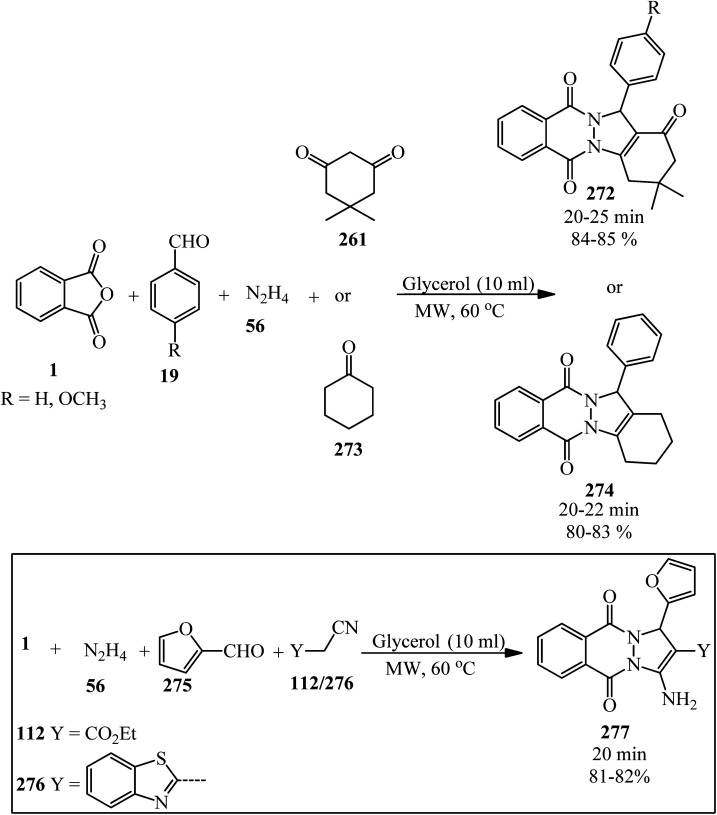
Synthesis of 2*H*-indazolo[2,1-*b*]phthalazine-triones, 3,4-dihydro-1*H*-indazolo[1,2-*b*]phthalazine-6,11(2*H*,13*H*)-diones, and 3-amino-2-(benzo[*d*]thiazol-2-yl)-1-(furan-2-yl)-1*H*-pyrazolo[1,2-*b*]phthalazine-5,10-diones.

Some research groups utilized 1,3-cyclohexanedione (273) (in addition with dimedone) to prepare (272) and 3,4-dihydro-1*H*-indazolo[1,2-*b*]phthalazine-6,11(2*H*,13*H*)-diones (274), which consist of (A). Sadek *et al.* in 2019 developed a highly efficient, catalyst-free, one-pot, multicomponent synthesis of various indazolophthalazines (272, 274) in glycerol as a cheap, biodegradable, and commercially available promoting solvent and catalyst under controlled microwave heating (Scheme 105).^256^ The reaction mixture in glycerol was heated under reflux in a Milestone microwave lab station at 60 °C. They also synthesized (277) *via* the same reaction conditions.

(B) Pal's group in 2016 prepared the compounds (272, 274) *via* the one-pot four-component reaction of PA (1), variety of aldehydes (19) (including aliphatic and (hetero)aromatic aldehydes), hydrazine hydrate (56), and active methylene compounds (dimedone (261)/1,3-cyxlohexandione (273)) in the presence of magnetic Fe_3_O_4_–glutathione core–shell (nano-FGT, 10 mg) at 80 °C under solvent-free conditions within 20–30 min with 87–97%. The magnetic catalyst could be separated easily by an external magnet and reused in five more consecutive runs without much decrease in the catalytic activities. The authors also examined the efficacy of the catalyst in the synthesis of two kinds of phthalazine-diones (271) *via* the reaction of PA (1), aldehydes (19) (which are 4-chloro-3-nitrobenzaldehyde and pentanal), aminonitriles/ethyl cyanoacetate (112), and hydrazine hydrate (56) in a 1 : 1 : 1 : 1.2 molar ratio in the same reaction conditions within 20–25 min with 92–97%.^257^

**Scheme 106:**
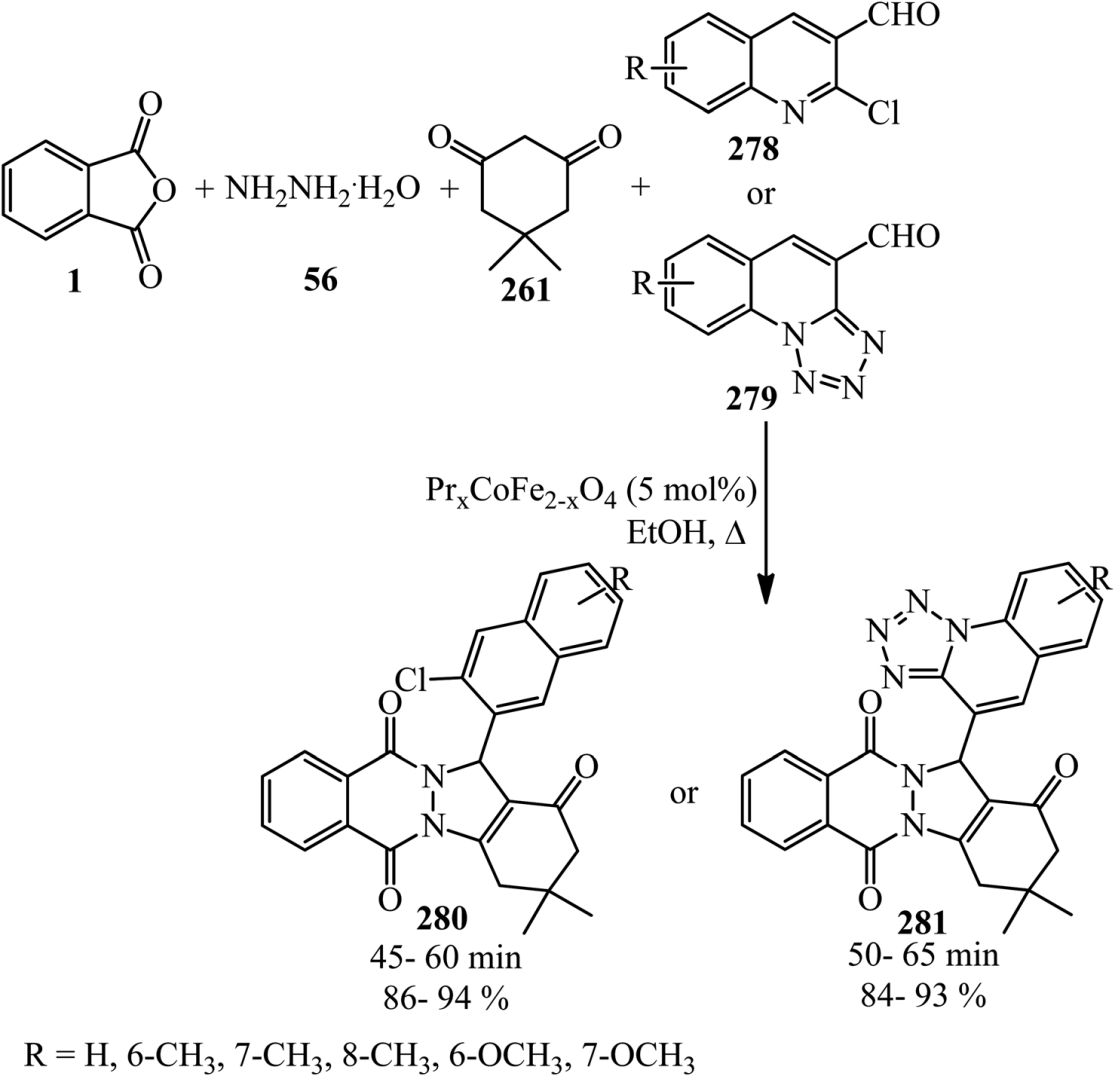
Synthesis of phthalazine-quinolines.

Zaheer *et al.* in 2016 exploited Pr_*x*_CoFe_2−*x*_O_4_ (*x* = 0.1) nanoparticles to catalyze the efficient one-pot four-component reaction of PA (1), hydrazine hydrate (56), dimedone (261), and various quinoline aldehydes (278, 279) to achieve phthalazine quinoline derivatives (280, 281) (Scheme 106).^258^ The synthesized adducts were evaluated for antibiofilm activity against *P. aeruginosa* and *C. albicans*.

**Scheme 107:**
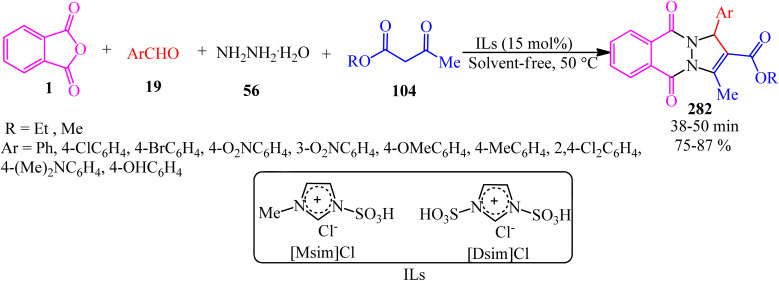
Synthesis of pyrazolo[1,2-*b*]phthalazine-2-carboxylates.

Zahedifar's research group in 2018 constructed a series of novel pyrazolo[1,2-*b*]phthalazine-2-carboxylates (282) *via* the one-pot four-component reaction of PA (1), aromatic aldehydes (19), hydrazine hydrate (56), and alkyl acetoacetates (104) in the presence of acidic ionic liquids such as 3-methyl-1-sulfo-1*H*-imidazol-3-ium chloride ([Msim]Cl), 1,3-disulfo-1*H*-imidazol-3-ium chloride ([Dsim]Cl), and triethyl(sulfo)ammonium chloride ([Et_3_NSO_3_H]Cl) as the catalyst (Scheme 107).^259^

**Scheme 108:**
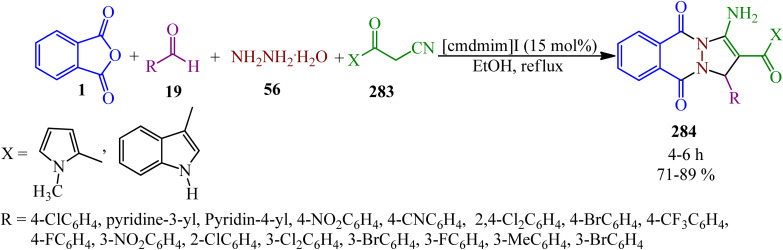
Synthesis of novel 1*H*-pyrazolo[1,2-*b*]phthalazine-5,10-dioness by [cmdmim]I.

Jahanshahi and Mamaghani in 2019 described the synthesis of a wide range of novel 1*H*-pyrazolo[1,2-*b*]phthalazine-5,10-dioness (284) in the presence of acetic acid-functionalized imidazolium salt (1-carboxymethyl-2,3-dimethylimidazolium iodide, [cmdmim]I), as a newly synthesized Brønsted acid catalyst. The reaction occurred *via* tandem Knoevenagel cyclocondensation of PA (1), aromatic aldehydes (19), hydrazine hydrate (56), and 3-(1-methyl-1Hpyrrol-2-yl)-3-oxopropanenitrile (or 3-(1*H*-indol-3-yl)-3-oxopropanenitrile) (283) in the presence of [cmdmim]I in ethanol under reflux conditions (Scheme 108).^260^ The reusability of the catalyst was examined in 5 consecutive runs without any appreciable decrease in the activity.

**Scheme 109:**
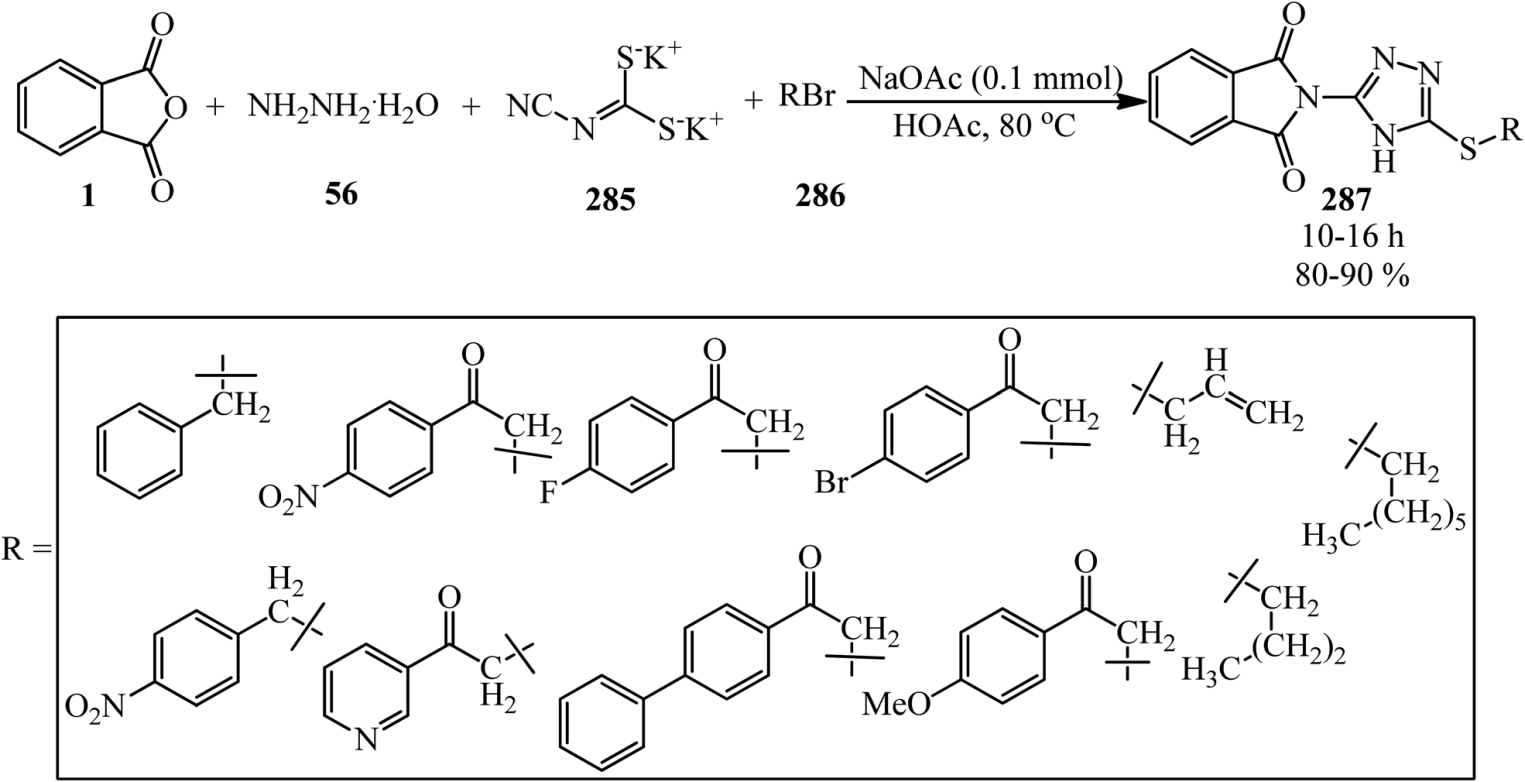
Synthesis of alkyl/aralkyl/phenacyl thiotriazolyl isoindoline-1,3-diones.

In 2022, Vedula and coworkers provided alkyl/aralkyl/phenacyl thiotriazolyl isoindoline-1,3-diones (287) *via* the reaction of PA (1), hydrazine hydrate (56), dipotassium cyanodithioimidocarbonate salt (285), and alkyl/aralkyl/phenacyl bromides (286) in a 1 : 1.5 : 1 : 1 molar ratio using acetic acid and sodium acetate (0.1 mmol) *via* a one-pot four-component reaction at 80 °C (Scheme 109). The *in vitro* anticancer activity of the products revealed that some of them demonstrated cytotoxic assay against HeLa cancer cell lines. The compounds also subjected to their docking analysis and DFT calculations.^261^

## 5. Applications of PA in five-component and higher-component reactions

**Scheme 110:**
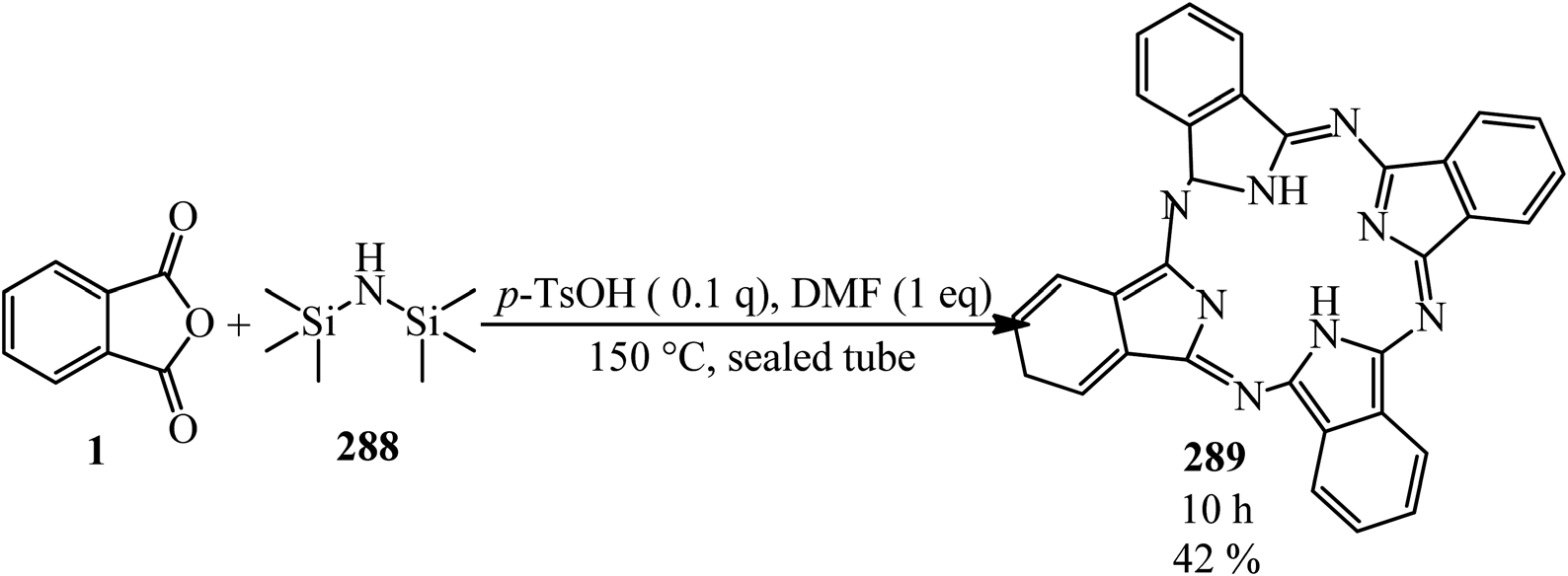
Synthesis of phthalocyanines.

Toru's group in 2003 reported the preparation of metal-free phthalocyanines from PA (1) with hexamethyldisilazane (HMDS) (288) in a 1 : 5 molar ratio (Scheme 110).^262^ The transformation is actually a pseudo eight-component reaction.

**Scheme 111:**
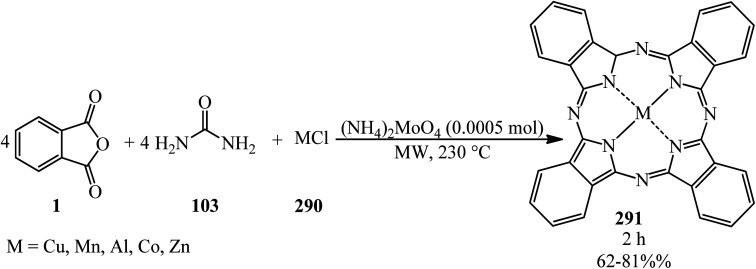
Synthesis of metal phthalocyanines.

Park *et al.* in 2004 developed a green procedure for the synthesis of metal phthalocyanines (291) (MPc: M = Cu, Mn, Al, Co, and Zn) through the condensation reaction of PA (1) (0.283 mol) and urea (103) (0.816 mol) with various metal chlorides (290) (7 g) in the presence of ammonium molybdate (5 × 10^−4^ mol) as a catalyst under microwave-assisted solvent-free conditions (Scheme 111).^263^ The procedure actually is a pseudo nine-component reaction.

## 6. Applications of PA in esterification

**Scheme 112:**
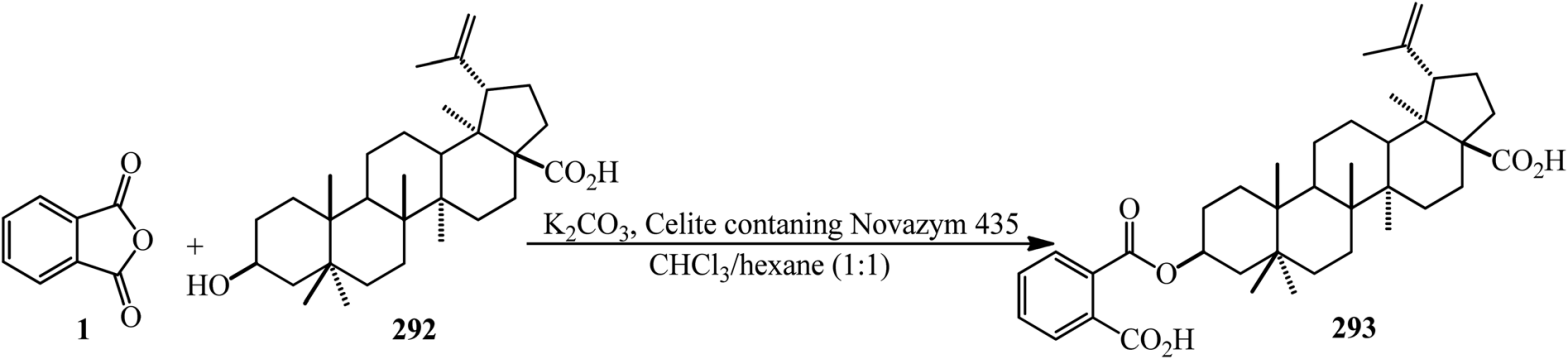
Synthesis of 3-*O*-phthalyl betulinic acid.

Ahmad *et al.* in 2010 reported immobilized Candida Antarctica lipase, Novozym 435, as a biocatalyst to catalyze the esterification reaction of PA (1) and betulinic acid (292) to obtain 3-*O*-phthalyl betulinic acid (293) in *n*-hexane/chloroform. The effect of different parameters in the reaction process was predicted by the “response surface methodology” (RSM) technique. The comparison of the results of this model and experimental values revealed a good correspondence. The effect of enzyme amount was found to be less, while the reaction temperature, reaction time, and molar ratio strongly affected the ester yield. According to the “central composite rotatable design” (CCRD) optimization, in the presence of a betulinic acid to phthalic anhydride in a 1 : 1.11 molar ratio, the maximal yield of the ester (64.7%) was obtained using 145.6 mg enzyme at 53.9 °C in 20.3 h. This predicted that the optimum conditions are in close correlation with the experimental results (Scheme 112).^264^

Ahmad's research group in 2010 also considered the reaction parameters of lipase-catalyzed esterification of betulinic acid (292) using PA (1) in organic solvent media.^265^ The lipase from *Candida antarctica* immobilized on an acrylic resin (Novozym 435) was employed for esterification. The influence of different reaction parameters, such as effect of single and mixed solvents, substrate molar ratio, reaction time, temperature, amount of enzyme, effect of inorganic bases, and effect of substrate support were investigated and optimized. The optimum conditions to obtain 3-*O*-phthalyl-betulinic acid (293) (61.8%) are: substrate molar ratio (betulinic acid : phthalic anhydride, 1 : 1), within 24 h at 55 °C, enzyme (176 mg), and Celite (170 mg) in 1 : 1 mixture of chloroform and *n*-hexane as a solvent in the presence of K_2_CO_3_ (as an inorganic base).

**Scheme 113:**
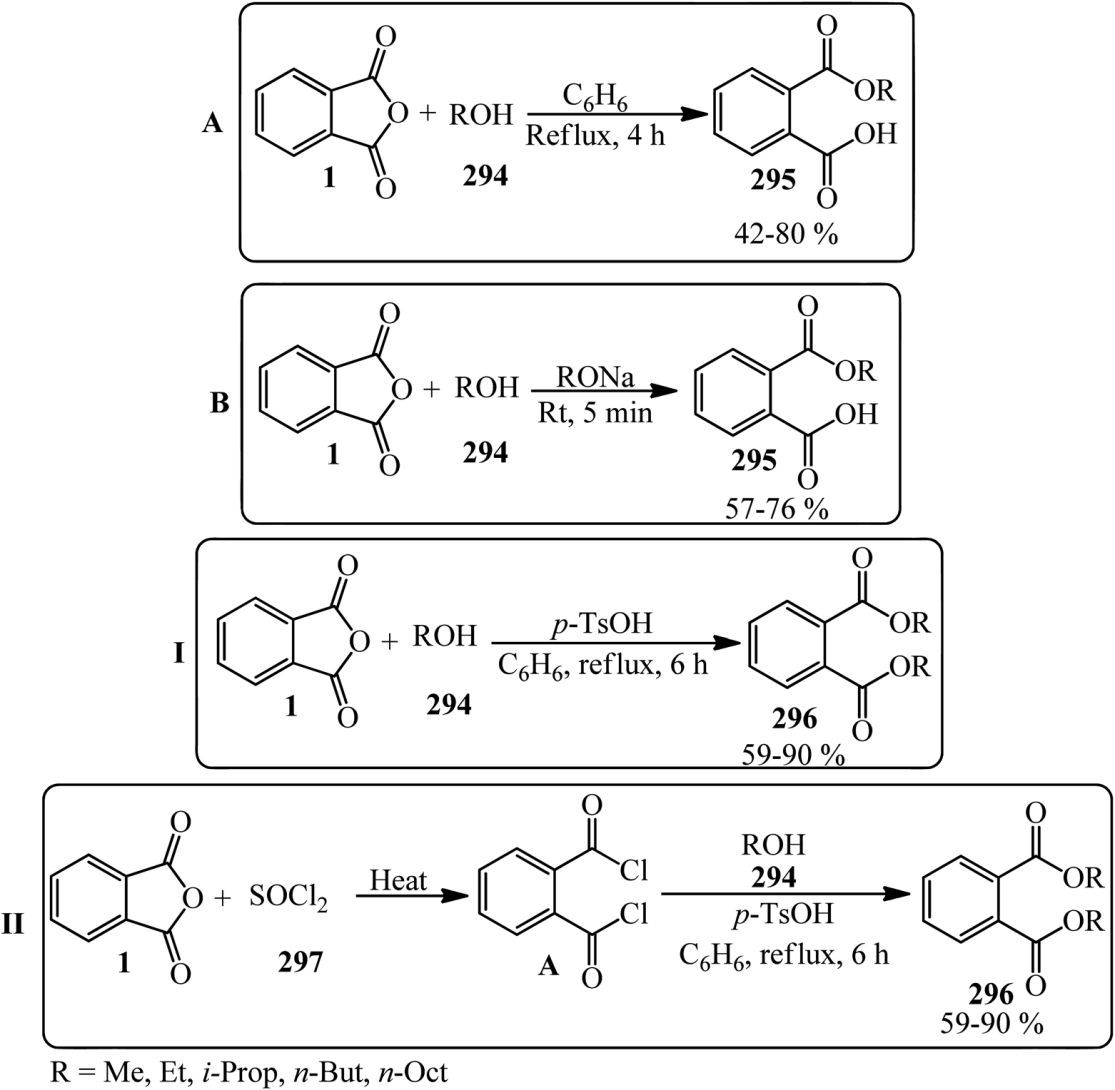
Synthesis of monoesters and diesters.

Dubey's research group in 1997 converted PA (1) to its monoesters through the reaction with simple alcohols (294) under a variety of conditions (Scheme 113).^266^ The monoesters (295) could be prepared *via* two procedures: (A) reaction of PA and alcohols in a 1 : 2.2 molar ratio, in refluxing benzene, (B) reaction of PA with alkyl alcohols in 1 : 3.8 molar ratio in the presence of sodium alkoxide (0.02 g) at room temperature with 5 min. The diesters (296) were also obtained *via* various procedures: (I) reaction of PA and alcohols in 1 : 3.2 molar ratio in benzene solvent in the presence of *p*-TSA (5 mg) under reflux conditions in a dean-stark apparatus within 6 h; (II) the reaction of PA with thionyl chloride (297) for 1 h under reflux conditions, which was followed by separating thionyl chloride, and consequent addition of pyridine and an appropriate alcohol and refluxing for 3 h.

Fareghi-Alamdari and coworkers in 2017 manufactured two functionalized diacidic ionic liquids (FDAILs) including hydroxyl functionalized diacidic IL [3,3′-(2,2-bis (hydroxymethyl)propane-1,3-diyl)bis(1-methyl-1*H*-imidazole-3-ium)hydrogen sulfate, HFDAIL] and sulfonated diacidic IL [3,3′-(2,2-bis((sulfoxy)methyl)propane-1,3-diyl)bis(1-methyl-1*H*-imidazole-3-ium)bromide, SFDAIL] by a simple method in high yields, which served for the esterification reaction of anhydrides (PA, succinic anhydride, and maleic anhydride) with some alcohols in a 1 : 5 molar ratio to give corresponding dialkyl esters (296) as plasticizers under solvent-free conditions.^267^ HFDAIL showed higher catalytic performance in comparison with other reported catalysts, suggesting its high acidity and hydrophilic property. Phthalates, which are a class of neutral plasticizers. Are widely used in nonmilitary industries. Recycling experiments suggested that ILs could be reused seven times without a remarkable loss in their catalytic activity. According to the proposed mechanism, HFDAIL has two hydroxyl groups, which can absorb the produced water by hydrogen bonding. Thus, the esterification reaction equilibrium will shift to the right and the product yield will increase.

Fareghi-Alamdari's group also introduced two highly acidic, imidazolium-based, functionalized dicationic ionic liquids (FDCILs), which used (in 0.2 eq.) as efficient and green catalysts in the synthesis of phthalate plasticizers through the esterification of PA with alcohols (such as ethanol, *n*-propanol and *n*-butanol) at 110 °C. Among these two FDCILs, (FDCIL 1: [(dimethyl-4-sulfobutyl-ammonium)-1,2-ethane-1*H*-imidazolium sulfonic acid]hydrogen sulfate and FDCIL 2: 3,3′-(1,2-ethanediyl)bis[1-(4-sulfobutyl)-1*H*-imidazolium sulfonic acid]hydrogen sulfate), the first one performed better. The catalytic activity of FDCIL is related to the density of acidic groups on it (higher acidity) and the length of the carbon chain (low lipophilic character) in the cationic part. The influences of the reaction temperature, catalyst dosage, and molar ratio of PA to alcohol on the esterification reaction were investigated. The reusability of the catalyst in these reactions was also studied. The yields were estimated by GC analysis.^268^ Phthalate plasticizers are the main plasticizers used as softening agents in various industrial applications. These compounds are mainly used as plasticizers for cellulosic resins and some vinyl ester resins, PVC, and nitrocellulose lacquers.^269^

Fareghi-Alamdari in 2018 also prepared supported diacidic ionic liquid on magnetic silica nanoparticles (SDAIL@magnetic nano SiO_2_) and investigated its catalytic activity (10 mol%) for the selective diesterification of alcohols (2-methoxy ethanol, allyl alcohol, 2-ethoxyhexanol, butanol, propanol, ethanol, and methanol) with PA in a 5 : 1 molar ratio to afford the corresponding dialkyl plasticizers (296) under solvent-free conditions (65–180 °C) within 1–10 h. Under the optimized conditions, the conversion of PA was 100%, and the diester plasticizers were obtained with excellent yields (80–100%). The SDAIL@magnetic nano-SiO_2_ catalyst showed good reusability and could be easily separated from the reaction mixture using an external magnet, washed with dichloromethane, and reused for the next runs for up to 8 runs without significant activity loss.^270^

Shahedi and Mansoori in 2018 studied the esterification reaction of PA (1) with various aliphatic and cycloaliphatic alcohols (such as propanol and butanol) in the presence of a Fe_3_O_4_@SiO_2_–SO_3_H (5 mol%) nanocatalyst. The two mentioned alcohols gave the corresponding diesters (296) in duration of 8–12 h with a yield of 71–97%.^271^

Kulawska's group in 2011 considered the kinetics of the syntheses of higher aliphatic alcohols (C7, C9, C11) phthalates in an isothermal, semi-batch reactor. In the first stage of the process, the formation of monoester (295) was very fast and irreversible. In the second stage, the esterification of monoester toward diester (296) in the presence sulfuric acid catalyst progressed slowly. These reactions appeared to be first order with respect to the monoester and did not depend on the concentration of the alcohol.^272^

Mohammadpour Amini in 2003 evaluated the esterification of PA with 2-ethylhexanol and 1-butanol and ester decomposition of dioctyl phthalate (DOP) in the presence of different kinds of heteropolyacids such as (I) Keggin H_3_PW_12_O_40_, H_4_SiW_12_O_40_, and H_4_SiMo_12_O_40_; (II) Wells–Dawson H_6_P_2_W_18_O_62_, H_6_P_2_W_17_MoO_62_; (III) Preyssler H_14_[NaP_5_W_29_MoO_110_], H_14_[NaP_5_W_30_O_110_]. The heteropolyacids with Preyssler and Wells–Dawson structures (due to the higher number of acidic protons) and their molybdenum substituted derivatives (attributed to the reduction of Mo(vi) to Mo(v) and enhanced acidity) demonstrated higher activity in esterification and ester decomposition reactions than Keggin type heteropolyacids. A complete conversion of PA to dioctyl phthalate and dibutyl phthalate are achieved in 2 h in the presence of molybdenum-substituted Preyssler heteropolyacid. In the decomposition of dioctyl phthalate in the presence of Preyssler heteropolyacid, 2-ethylhexene was formed in quantitative yield.^273^

The kinetics of esterification of PA with 2-ethylhexanol was studied by Bhutada and Pangarkar in 1986 using three kinds of catalysts such as tetrabutyl titanate (TBT), tetrabutyl zirconate (TBZ), and *p*-TSA. In each case, the kinetic parameters such as rate constant (order with respect to various reactants and catalyst), activation energy, and collision frequency were determined. The titanates and zirconates were found to be better than *p*-TSA in rate and rate/product quality, respectively. The reaction shows general first order in the monoester and alcohol. Bimolecular acyl oxygen fission satisfactorily explains the kinetics.^274^

Gharibe in 2020 utilized the novel ZnAl_2_O_4_/SiO_2_ (5 mol%) for the esterification of PA with 2-ethylhexanol to obtain dioctyl phthalate (DOP) in 99.2% yield in 45 min.^275^

Brennecke's group in 1994 surveyed the pressure effect on the bimolecular rate constants for the esterification of PA with methanol in supercritical carbon dioxide (SC CO_2_) at 40 °C and 50 °C. They observed a 25-fold decrease in the bimolecular rate constant based on bulk concentrations when increasing the pressure from 97.5 to 166.5 bar.^276^

Khodadadi Moghaddam and Gholami in 2006 inspected the esterification of PA with 2-octanol in the presence of sulfated titania (SO_4_^2−^/TiO_2_ prepared by immersing titania powder in 1 N solution of sulfuric acid). The conversion reached an equilibrium composition in 20 min for dioctyl phthalate without water removal from the reactor. The linear dependence of conversion to the catalyst amount exhibited that there are no mass transfer limitations in the reaction conditions.^277^

Yadav's group in 1992 represented the efficacy of several solid superacidic catalysts [such as phosphate, borated, and sulfated zirconia, sulphated iron oxide, phosphotungstic acid (P_2_O_5_·24WO_3_·*n*H_2_O), and dodecatungstophosphoric acid (H_3_PO_4_·12WO_3_·*n*H_2_O)] in the preparation of the industrially important plasticizer dioctyl phthalate (DOP) from 2-ethylhexanol and PA.^278^

**Scheme 114:**
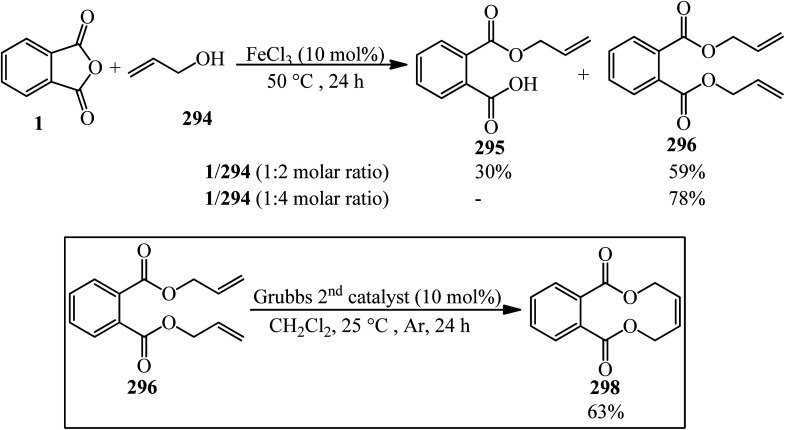
Synthesis of allylic mono- and diesters of PA and macrolide.

Bajracharya *et al.* in 2021 prepared phthalate monoesters (295) and diesters (296) *via* the reaction of PA (1) and alcohols (allyl, isoamyl, *n*-butyl, and benzyl alcohols) (294) through a one-pot two-step procedure in the presence of FeCl_3_ as a catalyst. Utilizing the reactants in a 1 : 2 molar ratio at 50 °C achieved both the phthalate esters (295, 296). Addition of excess amounts of alcohol at 50 °C or 100 °C yielded the selective preparation of diesters (296). The authors claimed that the synthesis of the adducts (295) was performed through a facile addition displacement pathway, which was continued by Lewis acid catalysis providing phthalate diesters (296). The ring-closing metathesis of diallyl phthalate using the Grubb's 2nd generation catalyst led to macrolide (298) (Scheme 114).^279^

**Scheme 115:**
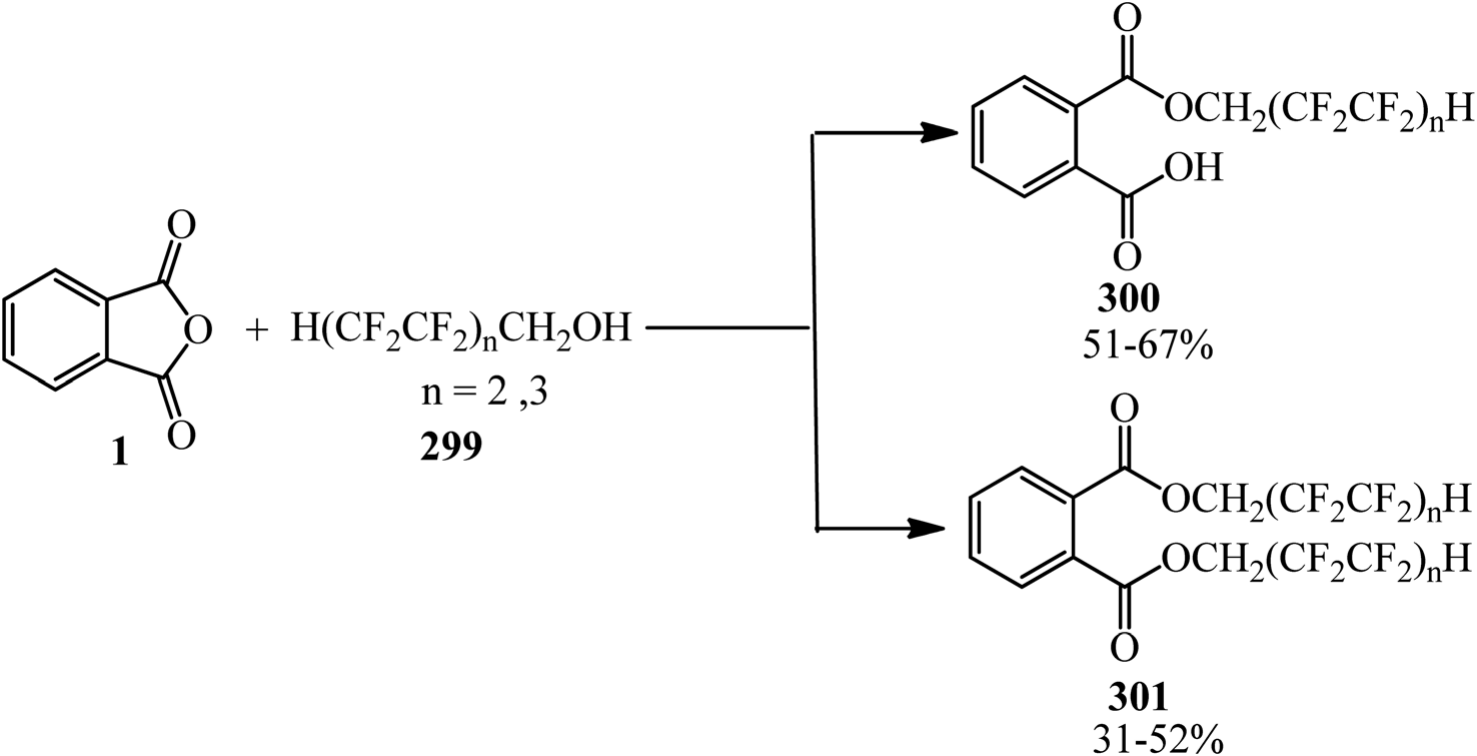
Synthesis of polyifluoroalkyl mono- and diesters.

Polyifluoroalkyl mono- and diesters of PA were obtained by Rakhimova and Kudashev in 2011 through the reaction of PA (1) with polyfluorinated alcohols (299). The monesters (300) were achieved *via* the ultrasonic-assisted (40 kHz) reaction of equimolar amounts of PA and polyfluorinated alcohol in cyclohexanone for 2 h at 70 °C, which was followed by heating at 130 °C for 2 h to complete the transition of phthalic anhydride. The diesters (301) were also prepared as the same procedure in the presence of PA : alcohols in a 1 : 2 molar ratio (Scheme 115).^280^

Karimi Alavijeh and Amini in 2019 demonstrated that mesoporous MIL-101(Cr)-SO_3_H and [Cr_3_O(BDC-SO_3_H)_2.4_(BDC-SO_3_NH_3_Bu)_0.6_]_*n*_ (20% BuNH_2_) acted as potent and robust catalysts in the esterification of PA (1) with various alcohols in a 1 : 5 molar ratio under solvent-free conditions.^281^ After the completion of the reaction, the catalyst was separated by centrifugation, washing with dichloromethane, drying at 50 °C, and reusing in the next reaction for 6 runs without activity loss.

Fieser in 1931 discussed about the condensation of β-naphthol with phthalic anhydride in detail.^282^

## 7. Some examples of PA utilization in multistep applicable products

PA could function as a key substrate in multicomponent sequential reactions, which led to applicable products in various fields such as drugs, natural products, and industry. Some examples have been discussed below.

**Scheme 116:**
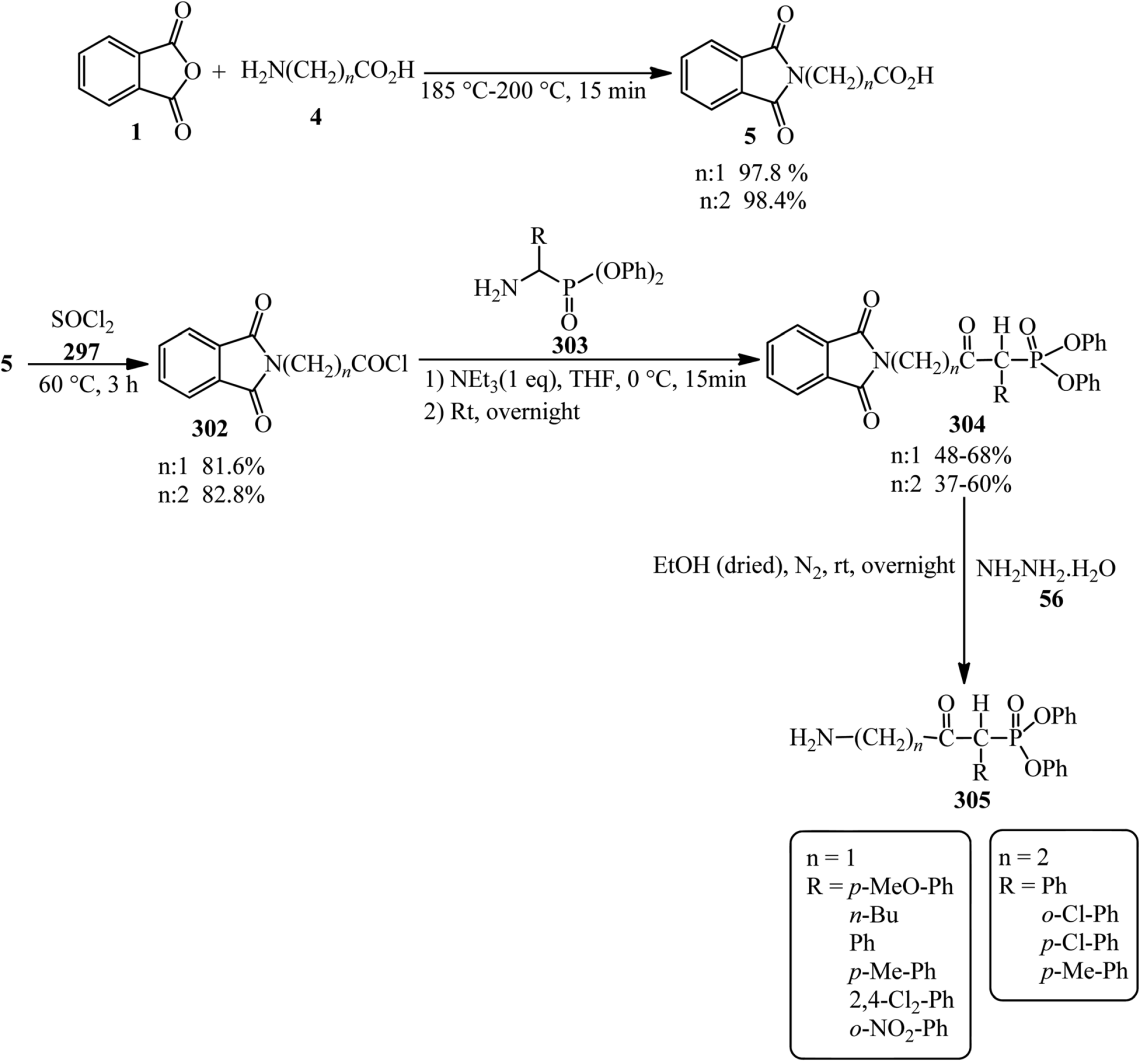
Phosphonodipeptides and homolog preparation procedure.

**Scheme 117:**
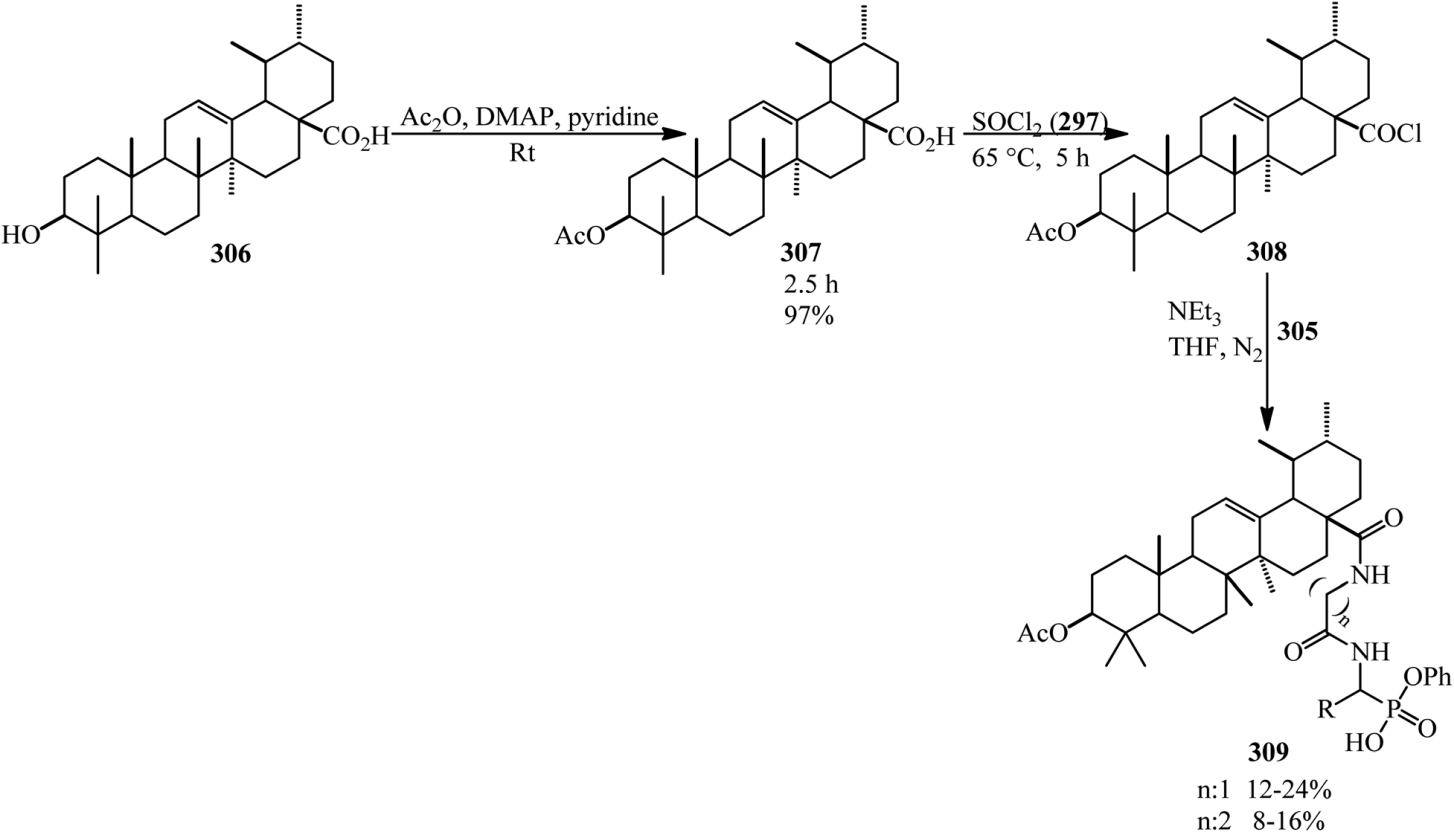
Novel phosphonodipeptide conjugates of ursolic acid and their homologs.

Cavé *et al.* in 2008 prepared novel phosphonodipeptide conjugates of ursolic acid (3β-hydroxy-urs-12-en-28-oic acid) and their homologs *via* a series of reactions. First, the preparation of a series of α-phosphonodipeptides and homologs has been reported (Scheme 117). According to Scheme 116, the 1,3-dihydro-1,3-dioxo-2*H*-isoindole-2-acetic (or propanoic) acids (5) were obtained by mixing the grounded forms of PA (1) and amnio acids (4) (glycine and β-alanine), which was converted to acyl chloride by SOCl_2_ (297) to phthalimidoacetyl chloride (302). The subsequent reaction of (302) with α-aminophosphonates (303) yielded *N*-blocked phosphonodipeptides and homologs (304). The phosphonodipeptides and homologs (305) were obtained from hydrazinolysis with hydrazine hydrate (56). In the second step (Scheme 117), the introduction of phosphonodipeptides and their homologs (305) to naturally bioactive ursolic acid (UA) (306) at C-28 moiety afforded new classes of phosphonodipeptide conjugates of ursolic acid and their homologs (309), which was performed by (i) its acylation to 3β-acetoy-urs-12-en-28-oic acid (307); (ii) chlorination to 3β-acetoy-urs-12-en-28-oyl chloride (308), and (iii) final reaction with (305) to afford the (309) adducts.^283^ The phosphonodipeptide conjugates of UA and their homologs (309) are potent for anti-HT29 (human colon cancer cell line) and anti-HIV properties.

**Scheme 118:**
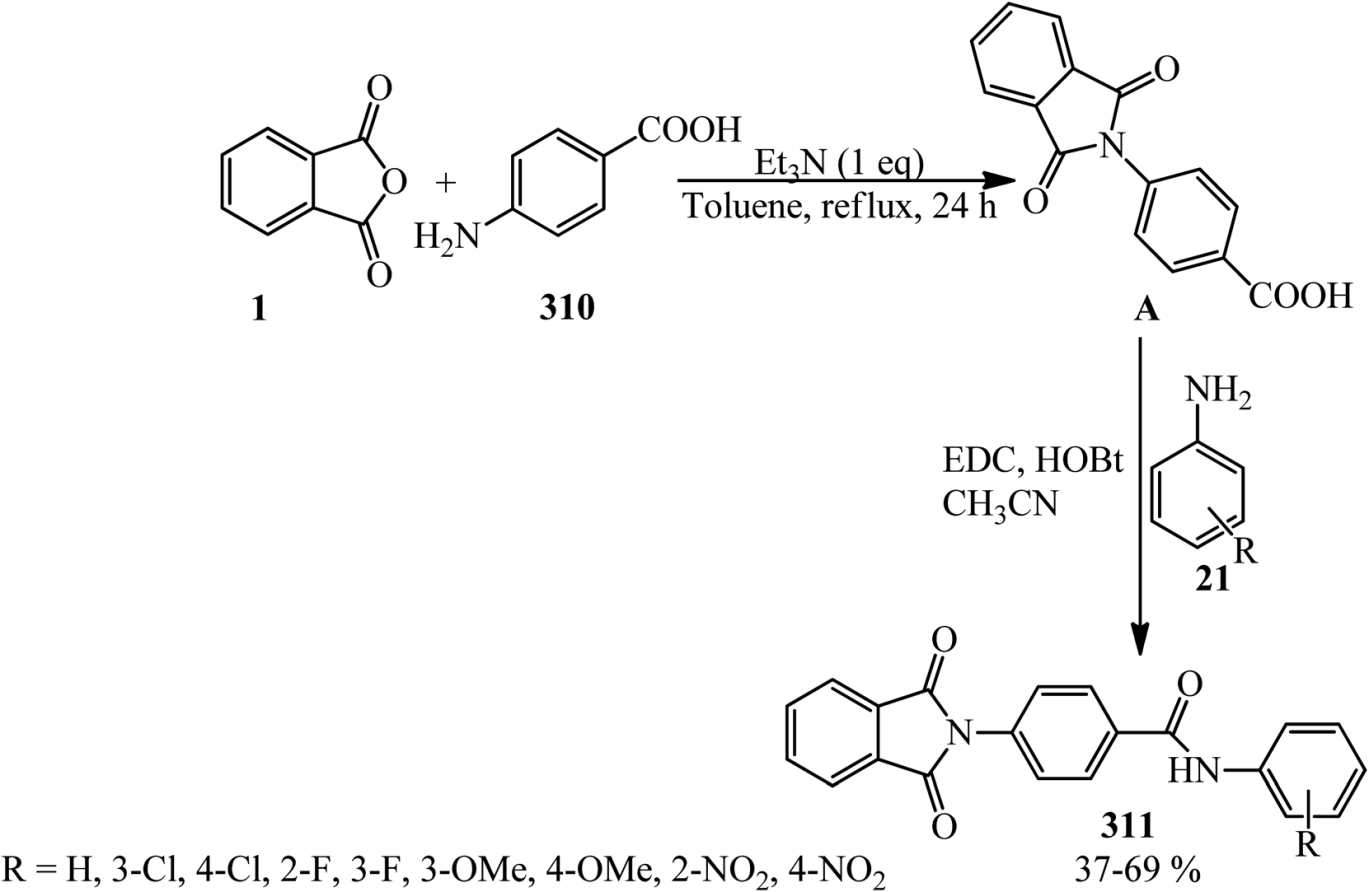
Synthesis of 4-(1,3-dioxoisoindolin-2-yl)-*N*-phenylbenzamides.

Aliabadi and coworkers in 2016 synthesized a new series of phthalimides named 4-(1,3-dioxoisoindolin-2-yl)-*N*-phenylbenzamides (311) *via* the reaction of PA (1) with 4-aminobenzoic acid (310). The first step consisted of Gabriel-like reaction in the presence of triethylamine (as a proton acceptor) under reflux conditions to obtain 4-(1,3-dioxoisoindolin-2-yl)benzoic acid (A), which was mixed with equimolar quantities of *N*-ethyl-*N*-dimethyl aminopropyl carbodiimide (EDC, as a carbodiimide coupling agent) and hydroxyl benzotriazole (HOBt, additive agent) in acetonitrile solvent and was stirred for 30 min at room temperature. Then, an equimolar quantity of appropriate anilines (21) was added to the reaction medium. On continuous stirring for 24 h, an amidation reaction through a carbodiimide coupling reaction yielded the adducts (311). The potential of the products as anti-Alzheimer agents was examined. The anti-acetylcholinesterase activity of the synthesized derivatives was assessed by Ellman’s test. The product, with ortho nitro moiety, in this series exhibited the highest inhibitory potency (IC_50_ = 1.1 ± 0.25 μM) compared to donepezil (IC_50_ = 0.41 ± 0.12 μM) as the reference drug (Scheme 118).^284^

**Scheme 119:**
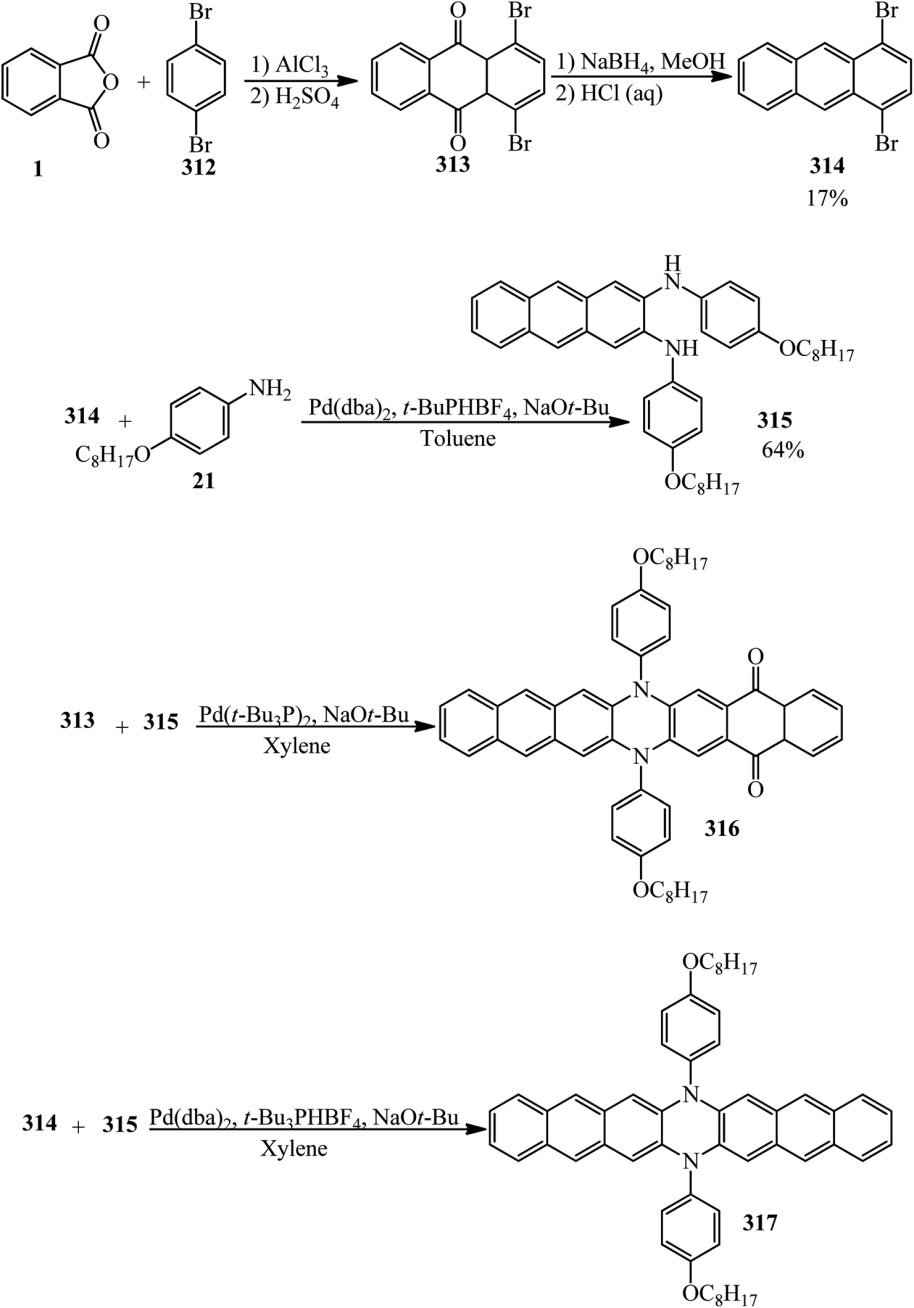
Synthetic procedure of 7,16-bis(4-(octyloxy)phenyl)-7,16-dihydrodinaphtho[2,3-*b*:2′,3′-*i*]phenazine and 7,16-bis(4-(octyloxy)phenyl)-16,18*a*-dihydrodinaphtho[2,3-*b*:2′,3′-*i*]phenazine-5,18(4*aH*,7*H*)-dione.

Iwanaga in 2019 prepared dinaphtho[2,3-*b*:2′,3′-*i*]dihydrophenazine derivatives *via* a multistep reaction starting from PA. First, Friedel–Crafts reaction of PA (1) with 1,4-dibromobenzene (312), followed by dehydration, afforded 2,3-dibromoanthraquinone (313), which reduced stepwise to give 2,3-dibromoanthracene (314) with 17% overall yield. Pseudo three-component conventional Buchwald-Hartwig coupling of (314) with 4-octyloxyaniline (21)^285^ gave compound (315) as a yellow solid. The reaction of (315) with (313) and (314) gave 7,16-bis(4-(octyloxy)phenyl)-16,18*a*-dihydrodinaphtho[2,3-*b*:2′,3′-*i*]phenazine-5,18(4*aH*,7*H*)-dione (316) and 7,16-bis(4-(octyloxy)phenyl)-7,16-dihydrodinaphtho[2,3-*b*:2′,3′-*i*]phenazine (317), respectively. The X-ray crystallographic analysis of (317) indicated a planar structure. The photophysical properties were influenced by nitrogen atoms, resulting in extended π-conjugation for (317) and intramolecular donor–acceptor interactions for (316). The oxidation potentials of (316, 317) were similar due to the independence of the 2,3-diaminoanthracene unit (Scheme 119).^286^

**Scheme 120:**
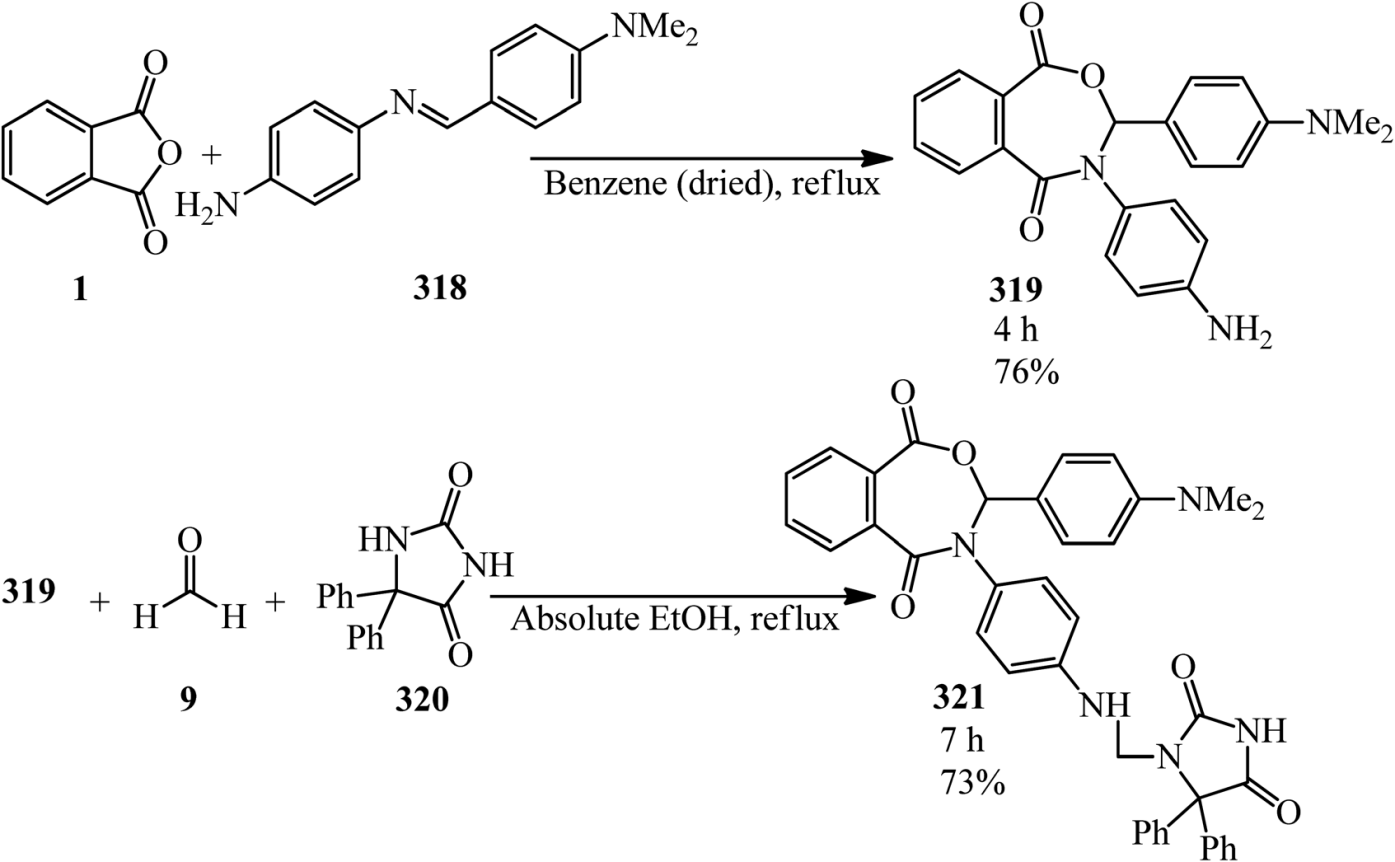
3-(4-(Dimethylamino)phenyl)-4-(4-(((2,4-dioxo-5,5-diphenylimidazolidin-1-yl)methyl)amino)phenyl)-3,4-dihydrobenzo[*e*][1,3]xazepane-1,5-dione preparation.

4-(4-Aminophenyl)-3-(4-(dimethylamino)phenyl)-3,4-dihydrobenzo[*e*][1,3]oxazepine-1,5-dione (319) was constructed through the reaction of PA (1) and Schiff base (318). The Mannich reaction occurred between (319), paraformaldehyde (9), and phenytoin (320) in equimolar ratio to obtain 3-(4-(dimethylamino)phenyl)-4-(4-(((2,4-dioxo-5,5-diphenylimidazolidin-1-yl)methyl)amino)phenyl)-3,4-dihydrobenzo[*e*][1,3]oxazepine-1,5-dione (321). The authors investigated the preparation of some other phenytoin derivatives containing diazepines and quinazoline rings through the reaction of (318) with malic anhydride and phthalic imide. The anticancer and cytotoxicity properties of the products was tested through the effects of treating the *L20B* cell line (Scheme 120).^287^

**Scheme 121:**
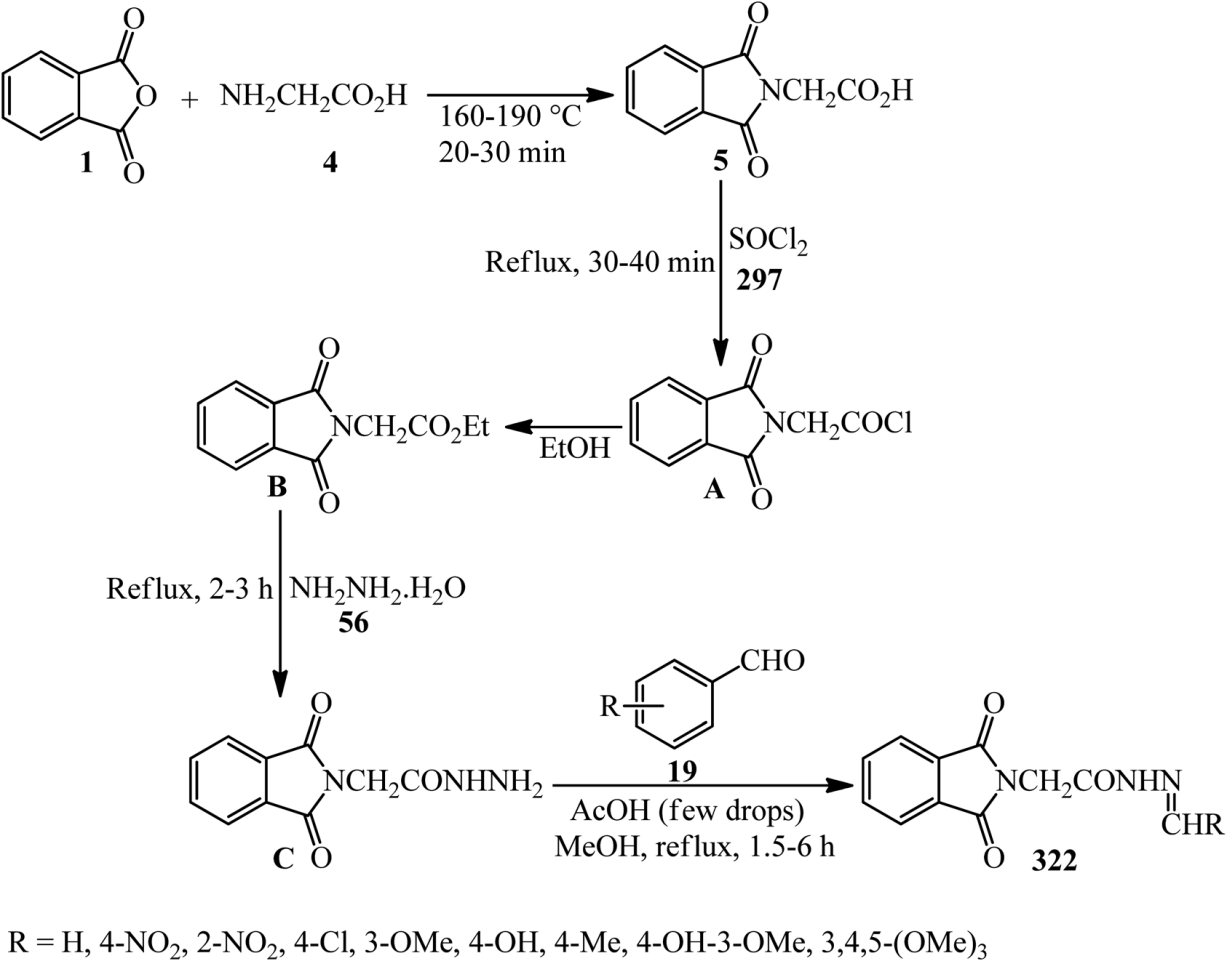
Synthesis of 2-(1,3-dioxo-1,3-dihydro-2*H*-isoindol-2-yl)-*N*′-(substituted benzylidene)acetohydrazides.

Bala's group in 2014 introduced phthalic anhydride-based benzylidene-hydrazides (322) as novel, potential antiinflammatory agents. The synthetic route started from PA (1) and glycine (4) to give phthaloylglycine (5), which was subjected to chlorination by thionyl chloride to form (A). Subsequent esterification, followed by reaction with hydrazine hydrate (56), and the final reaction of intermediate (C) with benzaldehydes (19) yielded 2-(1,3-dioxo-1,3-dihydro-2*H*-isoindol-2-yl)-*N*′-(substituted benzylidene)acetohydrazides (322). The research consists of computational studies. The products were screened for *in vivo* antiinflammatory and analgesic activities by carrageenan-induced Rat Paw Oedema and tail immersion methods, respectively, using diclofenac sodium as a standard drug (Scheme 121).^288^

## 8. Conclusion

In this literature survey, the authors focused on applications of phthalic anhydride (PA), a cyclic fused heterocyclic anhydride, as a versatile substrate in organic reactions. Actually, PA is an efficient substrate and/or intermediate in several organic transformations due to its bifunctional cyclic anhydride moiety, which could be opened *via* the attack of various nucleophilic groups. In addition to the introduction part, which identified the compound and its overall utilities in various respects, the review has been subdivided into some parts, centralizing on the organic two- and multicomponent reactions containing PA as the substrate. The importance and applicability of PA in esterification are also discussed with examples. There is also a final part that discusses the preparation of heterocycles starting from PA *via* multistep protocols, which possess various applications in pharmacology, treatment, natural products, industry, and some other fields of operative aspects of science and technology. In all the abovementioned parts, PA plays a crucial role as a substrate that cannot be changed with other heterocycles, which could be due to the intrinsic activity of the anhydride functional group. The authors hope that this review would be helpful and effectual for future research by chemists on PA and its analogs.

## Conflicts of interest

There are no conflicts to declare.

## Supplementary Material
